# Iron/N-doped graphene nano-structured catalysts for general cyclopropanation of olefins†
†Electronic supplementary information (ESI) available: Experimental details for catalyst preparation and catalytic reactions, additional catalytic results, catalyst characterisation, and cyclopropane characterisation. See DOI: 10.1039/d0sc01650k


**DOI:** 10.1039/d0sc01650k

**Published:** 2020-06-08

**Authors:** Abhijnan Sarkar, Dario Formenti, Francesco Ferretti, Carsten Kreyenschulte, Stephan Bartling, Kathrin Junge, Matthias Beller, Fabio Ragaini

**Affiliations:** a Dipartimento di Chimica – Università degli Studi di Milano , Via C. Golgi 19 , 20133 Milano , Italy . Email: fabio.ragaini@unimi.it; b Leibniz-Institut für Katalyse e.V. , Albert-Einstein-Straße 29a , 18059 Rostock , Germany . Email: matthias.beller@catalysis.de

## Abstract

The first examples of heterogeneous Fe-catalysed cyclopropanation reactions are presented. Pyrolysis of *in situ*-generated iron/phenanthroline complexes in the presence of a carbonaceous material leads to specific supported nanosized iron particles, which are effective catalysts for carbene transfer reactions. Using olefins as substrates, cyclopropanes are obtained in high yields and moderate diastereoselectivities. The developed protocol is scalable and the activity of the recycled catalyst after deactivation can be effectively restored using an oxidative reactivation protocol under mild conditions.

## Abstract 2

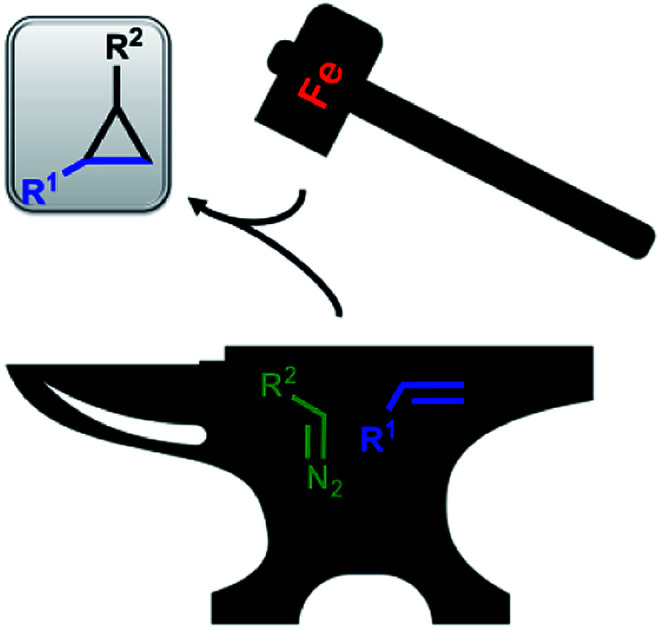
An Fe-based heterogeneous catalyst allows for the synthesis of cyclopropanes *via* a carbene transfer reaction, a transformation usually belonging to the homogeneous domain.

## Introduction

**Scheme 1:**
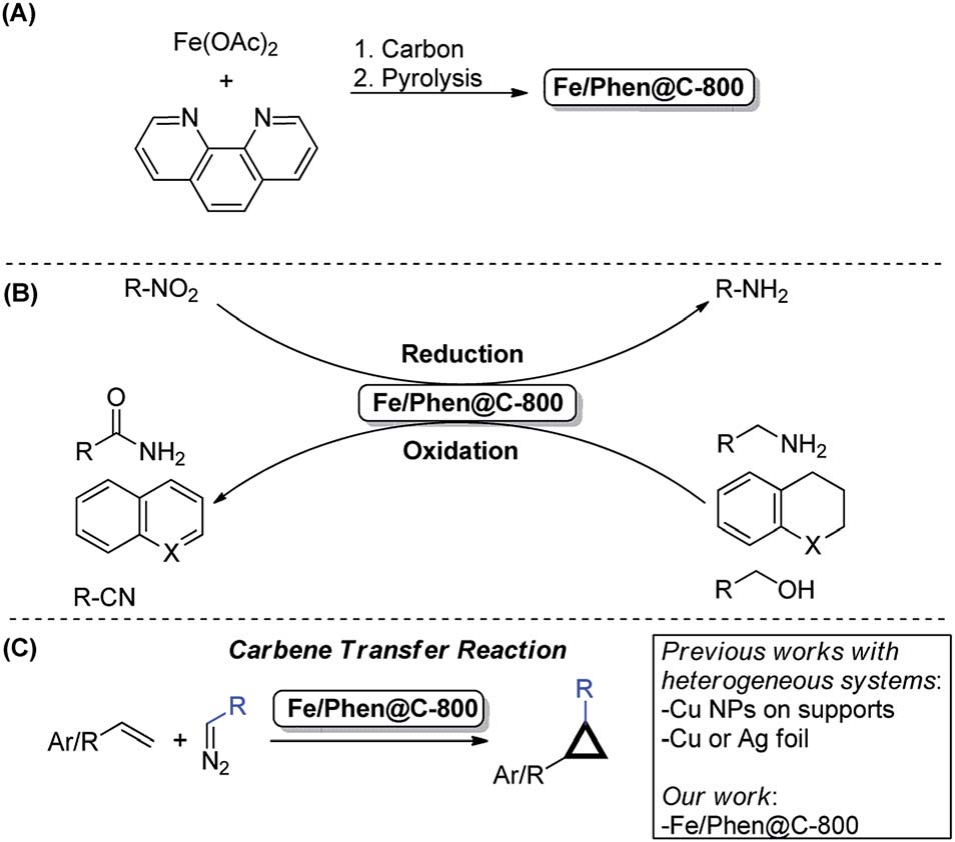
(A) Preparation of Fe/Phen@C-800, (B) selected known catalytic applications and (C) the present work: cyclopropanation reactions using Fe/Phen@C-800 as the catalyst.

The use of available, environmentally compatible and inexpensive 3d-metals in catalysis is an hot topic in modern chemistry.^1^–^4^ Among the various transition metals, particularly Fe is a privileged element due to its abundance, very low price and negligible toxicity.^5^–^7^ In the past decade, a remarkable number of reports were disclosed concerning the use of specific well-defined iron homogeneous complexes, supported or unsupported Fe-based nanoparticles, or other kinds of aggregates (*e.g.* nanoclusters) in various catalytic transformations.^6^,^8^–^15^ In 2013, inspired by electrocatalysis, some of us reported the development of an iron-based heterogeneous catalyst prepared by the pyrolysis of Fe(OAc)_2_/Phen (Phen = 1,10-phenanthroline) impregnated onto Vulcan carbon.^16^ According to the most recent and accurate characterization, the active material Fe/Phen@C-800 is composed of Fe-based nanoparticles (NPs), small Fe clusters, and even single Fe atoms embedded in a nitrogen doped carbon matrix (see below for a more detailed description). Based on this work, a whole family of catalytically interesting materials has been prepared using different approaches.^17^,^18^ So far, they have been mainly used in reduction/oxidation reactions and no application in other advanced organic transformations has been reported.^19^ Clearly, the discovery of new reactivity for iron-based heterogeneous catalysts will give new impetus for 3d-metal catalysis in general, and probably facilitate their actual implementation in industry (Scheme 1).

Inspired by the availability of different Fe oxidation states in such materials,^20^,^21^ we considered them also to be promising candidates for processes known to be catalysed by iron species in higher oxidation states. To prove this idea, the cyclopropanation of olefins using diazo compounds as carbene sources was chosen as a model reaction. Indeed, this transformation is efficiently catalysed by homogeneous Fe(ii/iii) complexes based on nitrogen-containing ligands such as porphyrins, phthalocyanines or other multidentate N- and N,O-ligands.^22^

Cyclopropanes constitute an important scaffold in natural products and many biologically active molecules including several pharmaceuticals and agrochemicals.^23^ Furthermore, their highly strained structure provides a surprising reactivity making them versatile reagents in synthetic organic chemistry.^24^ Thus, many homogeneous complexes based on Fe, Ru, Co, and Cu have been developed, which successfully catalyse this transformation.^25^,^26^ Along with homogeneous catalysts, immobilized metal complexes (mainly based on Cu and, to a less extent, on Ru and Rh) are also known to be active in cyclopropanations. However to date, very few reports describe the use of truly heterogeneous catalysts. After the pioneering work of Mayoral in 1997,^27^ Cu NPs on TiO_2_–Al_2_O_3_,^28^ Al_2_O_3_ (ref. 29) and core–shell Fe–Fe_*x*_O_*y*_ (ref. 30) were reported as catalysts. More recently, the groups of Coleman and Mack described the use of silver foil as a recyclable catalyst under mechanochemical conditions.^31^,^32^

Here, we present for the first time a heterogeneous iron catalyst for such reactions, which can be conveniently used and professionally recycled.

## Results and discussion

### Optimization and scope of olefin cyclopropanation using the Fe/Phen@C-800 catalyst

**Scheme 2:**
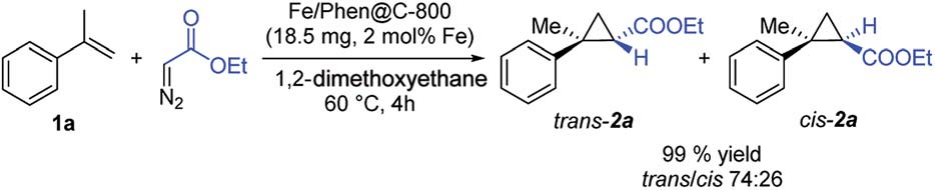
Cyclopropanation of **1a** using Fe/Phen@C-800 as the catalyst.

Initially, we explored the activity of Fe/Phen@C-800 in the reaction of α-methylstyrene **1a** with ethyl diazoacetate (EDA) as a model transformation (Scheme 2). To our delight, the Fe/Phen@C-800 catalyst is able to efficiently catalyse this reaction affording the cyclopropane **2a** in very high to excellent yields with a moderate diastereoselectivity in favour of the *trans* isomer. Control experiments using materials prepared by the same procedure employed for Fe/Phen@C-800, but omitting either Fe(OAc)_2_ or Phen, or by using Fe(OAc)_2_ and Phen as such resulted in no detectable formation of the desired product (Table S2, entries 6–8†).

Notably, the nature of the solvent has a minimal influence both on the reaction yield and diastereoselectivity making this reaction versatile in terms of the media profile (Table S2†). Applying 1,2-dimethoxyethane (DME) as solvent, 60 °C was identified as the optimal reaction temperature (Table S3†). Even though in most optimization experiments an excess of the olefin (5-fold amount) with respect to the diazo compound has been used, it is possible to decrease the amount of **1a** (1.5 eq.) and still a very good yield of the cyclopropanes can be achieved (Table S4, entry 3†). This demonstrates the applicability of the procedure even to more expensive olefins. Under the latter conditions, diethyl fumarate and diethyl maleate derived from EDA homocoupling were also observed as side products. Advantageously, the catalyst is water tolerant and only a slightly decreased yield was obtained using a “wet” solvent (Table S4, entry 4†).

**Scheme 3:**
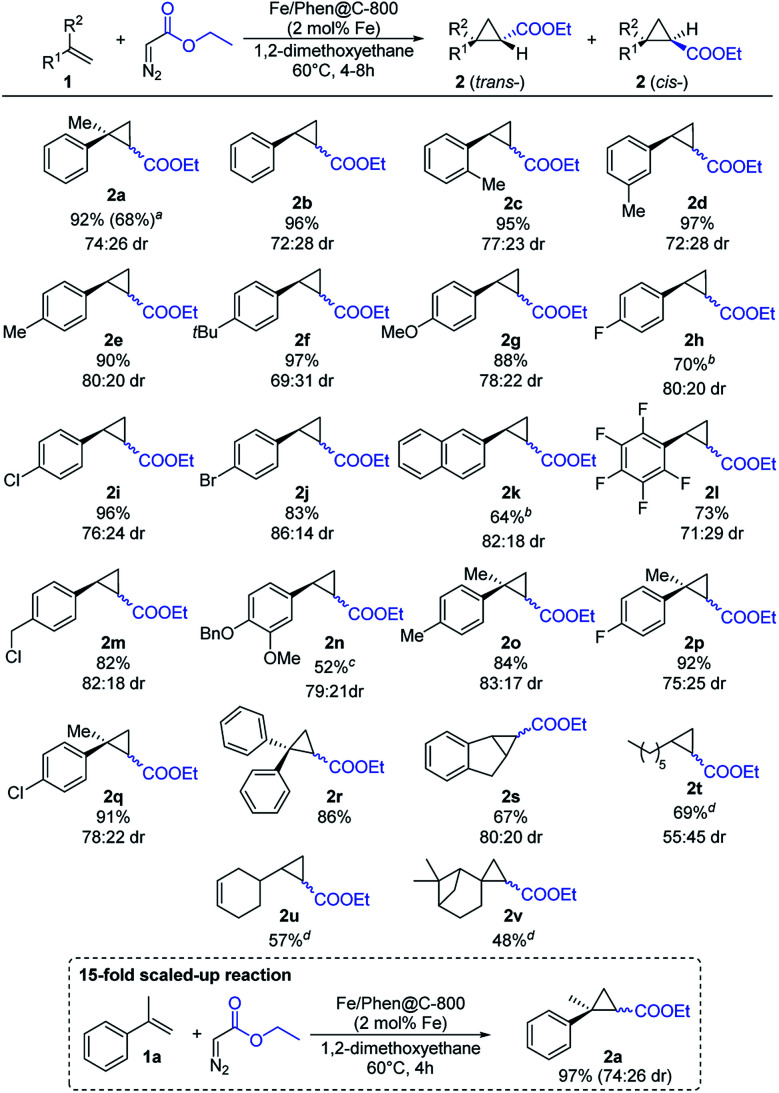
Substrate scope with respect to olefins. Reaction conditions: 0.50 mmol of EDA, 2.50 mmol of alkene, 18.5 mg of Fe/Phen@C-800 (corresponding to 2 mol% Fe), and 3 mL of DME at 60 °C for 4 h. Yields are based on the starting EDA and refer to the isolated compounds (sum of the *trans* and *cis* diastereoisomers). Diastereomeric *trans*/*cis* ratios (dr) are based on separated diastereomers. ^*a*^Isolated yield of the major isomer in parentheses, dr measured by ^1^H NMR. ^*b*^Only major isomer isolated, dr measured by ^1^H NMR. ^*c*^1.5 mmol of alkene was employed. ^*d*^Reaction time: 8 h. Isolated as an isomeric mixture. Where possible, dr are measured by ^1^H NMR.

Given the good results obtained in the benchmark reaction and the apparent robustness of the catalyst, the substrate scope was investigated (Scheme 3).

Styrene and related derivatives (**1b–1e**) gave the corresponding cyclopropanes in high yields regardless of the position of the substituent on the arene ring. Similarly, substrates with electron-donating groups (**1f–1g**) afforded the corresponding cyclopropanes in very high yields. Also halogen-containing substrates (**1h–1j**, **1m**, **1p**, **1q**) were well tolerated, maintaining intact the C–X bond, which can allow further functionalization. To our surprise, pentafluoro styrene (**1l**) as an example of an electron-poor substrate afforded the corresponding product in a good yield. Notably, more challenging aliphatic substrates such as 1-octene (**1t**) and β-pinene (**1v**) gave the products in satisfactory yields. Using vinyl cyclohexene (**1u**) the cyclopropanation reaction occurred regioselectively on the terminal olefinic bond maintaining intact the internal one. The selectivity for the terminal double bond in **1u** is explained by the lack of activity of the catalyst in the case of internal olefins (Fig. S1†), most likely due to a hindered approach of the substrate to the carbene formed on the surface of the catalyst.

**Scheme 4:**
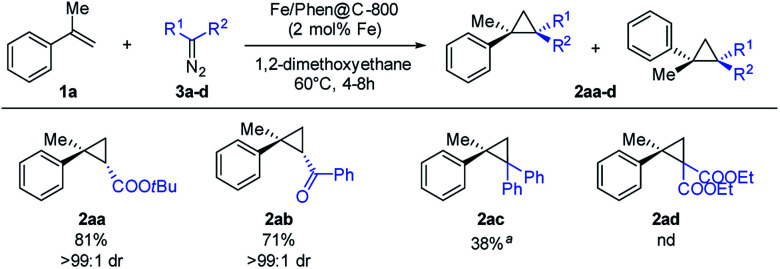
Substrate scope with respect to diazo compounds. Reaction conditions: 0.50 mmol of **3a–d**, 2.50 mmol of alkene, 18.5 mg of Fe/Phen@C-800 (2 mol% Fe), and 3 mL of DME, at 60 °C for 4 h. Isolated yields are reported, based on the starting EDA; dr were measured by ^1^H NMR. ^*a*^Reaction conditions: 100 °C in toluene for 8 h.

Under the optimized conditions, the scope of the diazo compound was also examined (Scheme 4). Mono-substituted diazo compounds (ester or ketone) gave the corresponding cyclopropanes **2aa–2ab** in excellent yields. Interestingly, the use of more sterically demanding diazo compounds has a dramatic positive effect on the diasteroselection, furnishing the *trans* isomer with >99 : 1 dr. The use of di-substituted diazomethanes proved to be more challenging. Nevertheless, diphenyldiazomethane yielded the product **2ac** in a moderate yield (100 °C, 8 h in toluene).

### Catalyst reactivation and recycling

**Fig. 1:**
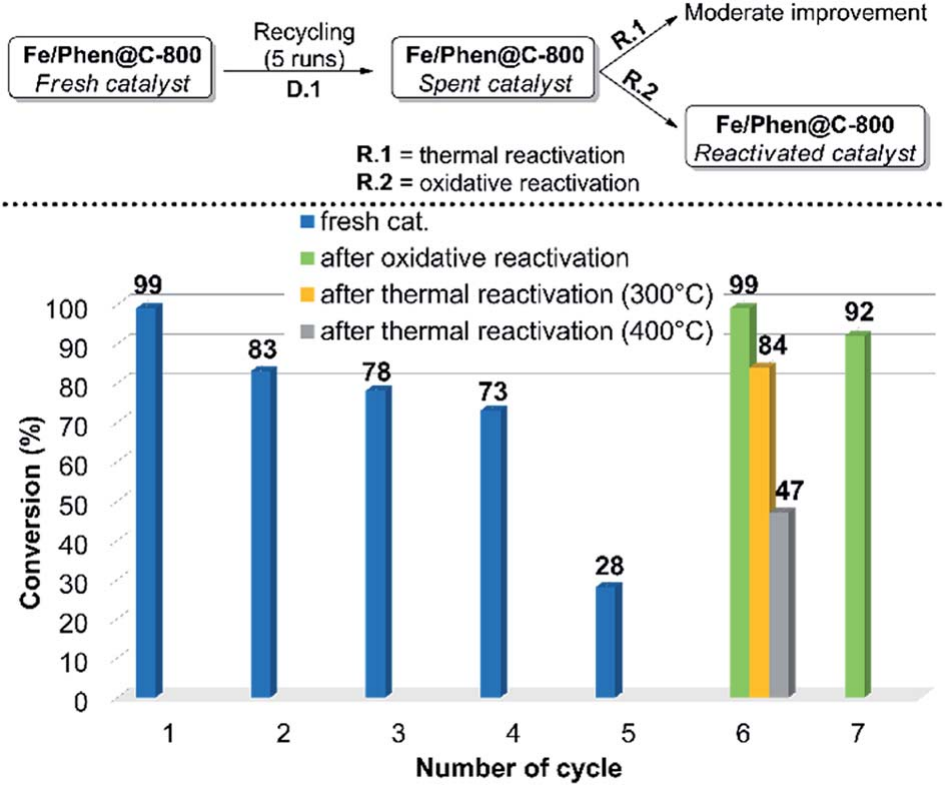
Recycling and reactivation steps. For experimental details regarding the recycling runs and the reactivation procedures see the ESI.†

In order to demonstrate the practical utility of this protocol, the scalability and recyclability of the system were studied. The model reaction was successfully scaled-up to 15-fold without significant variations of yield and diasteroisomeric ratio compared to the small-scale run. Regarding the recyclability, a significant decrease of the activity was observed after the 4^th^ run (D.1 in Fig. 1, cycles 1–5). ICP analysis of the solution showed a negligible (*ca.* 0.1%) loss of iron from the catalyst, so this cannot be the reason for deactivation.

In order to make the whole process both efficient and effective, two routes of re-activation of the spent catalyst were explored. The material obtained after 5 runs (Fe/Phen@C-800_S) was thermally treated under an inert atmosphere (R.1 in Fig. 1). However, despite a partial reactivation was gained, still complete conversion of EDA could not be achieved. In contrast, by treating Fe/Phen@C-800_S with an aqueous solution of 3 v/v% H_2_O_2_, the initial catalyst activity was restored (R.2 in Fig. 1)! The reactivated catalyst, Fe/Phen@C-800_R, could be further recycled as the fresh one (Fig. 1, cycle 7). Such an oxidative regeneration is typical for catalysts that suffer from physicochemical deactivation (*e.g.* fouling or poisoning).^33^,^34^ Apparently, the olefinic substrates and in particular styrenes underwent to a small extent oligo- and/or polymerization, which deactivates the iron catalyst. Indeed, we verified that complete deactivation of the catalyst occurred even by treating the parent material only with styrene under the reaction conditions. Also in this case, the activity was restored by oxidative treatment (see the ESI† for experimental details).

Note that most oxidative reactivation processes are based on the use of air at high temperatures, but such a treatment would result in the complete burning of the carbon support of our catalyst. Hydrogen peroxide has been reported as a milder alternative in a few cases.^35^–^37^ Moreover, the Fe^*n*+^/H_2_O_2_ system (known as Fenton's reagent) is also effective in these processes thanks to the generated HO˙ radicals.^38^ Thus, it appears that in our case both the direct action of H_2_O_2_ and related Fenton-like reactions are responsible for the removal of the oligomers/polymers from the spent catalyst.

### Characterisation of fresh, spent and reactivated catalysts

To further elucidate the reason for the declined activity, the fresh, spent and regenerated catalysts were characterized by X-ray photoelectron spectroscopy (XPS) and scanning transmission electron microscopy (STEM). XPS N 1s analysis showed common peaks at around 399 eV, 400 eV, and 401 eV for all the materials (Fig. S2–S6†). These results are in agreement with three different nitrogen bonding situations, namely N bonded in residual organic matrices and/or Fe–N_*x*_ centers, pyrrolic-N, and graphitic-N, respectively.^16^,^39^,^40^ A minor peak at 398.1 eV ascribed to pyridinic-N has been detected in Fe/Phen@C-800_S. C 1s core levels displayed an analogous pattern for all the materials (Fig. S2†). Indeed, signals corresponding to C=C/C–H (284.8 eV), C=N or C–O (approximately at 285 eV), and C–N or C=O (285–291 eV) functionalities can be detected.^41^ Deconvolution of the Fe 2p_3/2_ region showed four pairs of peaks which can be attributed to various Fe states. Peaks at around 708 eV, 711 eV, and 714 eV in the region correspond to Fe(0), Fe(ii) and Fe(iii), respectively.^42^ Additionally, the peak at 710 eV suggests the presence of Fe–N_*x*_ bonds which is in agreement with the previous results from N 1s spectra.^43^

**Fig. 2:**
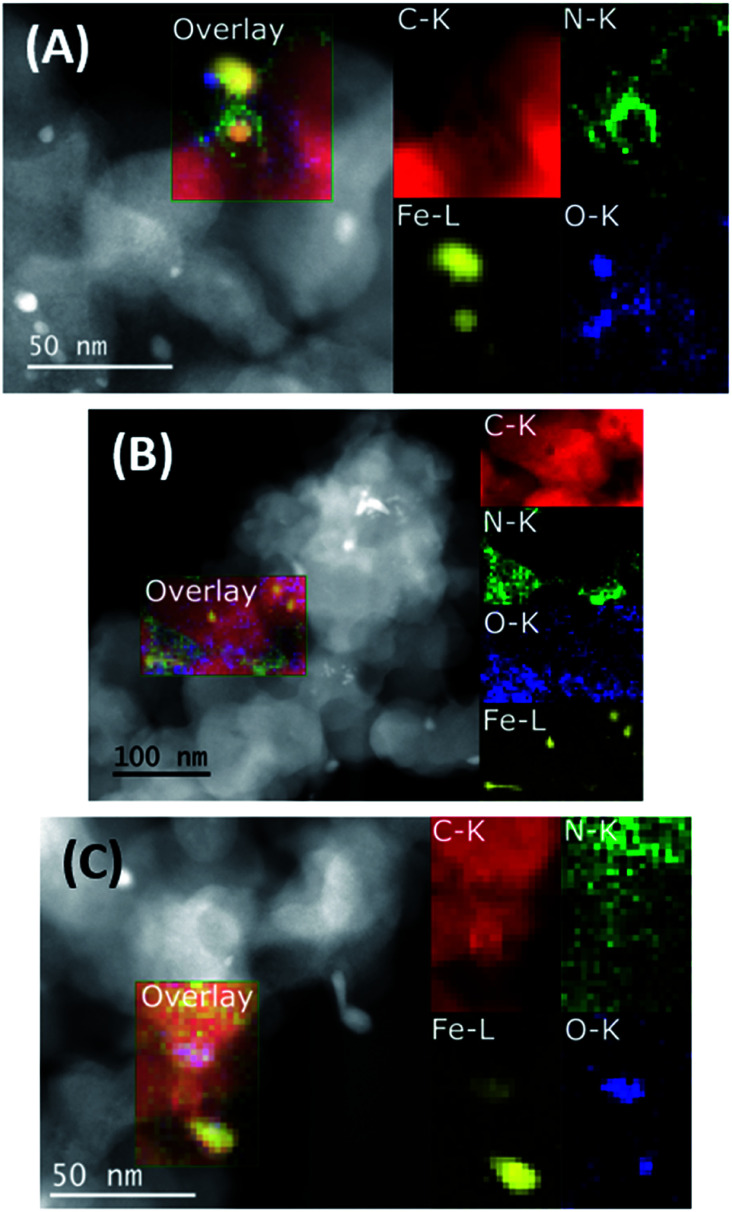
ADF-STEM images and EELS elemental maps of Fe/Phen@C-800 (A), Fe/Phen@C-800_S (B) and Fe/Phen@C-800_R (C).

Finally, the interpretation of the O 1s region is not trivial because of the large amount of oxygen functionalities both derived from oxygen groups present in the carbonaceous matrix^44^ and iron oxides.^45^ For this reason, unambiguous assignments are not possible here. In general, only small changes in the XPS data can be observed when comparing the fresh, spent, and regenerated catalysts. STEM images and analytical data of the three materials were then acquired. As depicted in Fig. 2, the annular dark field (ADF) STEM images and related elemental maps show the presence of Fe, N, and O on the carbon support. Nitrogen is mainly distributed in a phase also containing C, O and Fe on the surface of the support alongside Fe-based particles. In addition to the elemental maps, the different contrast visible in both the annular bright field (ABF) and high angle annular dark field (HAADF) images (Fig. S7†) confirmed the predominant presence of probably metallic Fe NPs in addition to Fe oxide NPs. Whereas layers of graphene covered the former, the latter are not covered thus indicating a possible protecting role of graphene towards oxidation. The nitrogen-doped amorphous carbonaceous matrix attached to the support showed the presence of dispersed Fe clusters. Their identity is also confirmed by electron energy loss spectra (see Fig. S10B†). The complex pattern revealed by Fe 2p XPS spectra reflects the variety of iron functionalities (metallic Fe, FeO_*x*_, and FeN_*x*_ centres) in the material. The spent catalyst (Fe/Phen@C-800_S) showed similar structures: still defined Fe-based NPs (mainly metallic and to a less extent oxidic) enveloped in carbon shells and dispersed iron clusters can be clearly observed (Fig. 2B, S8 and S10C†). Finally, the reactivated catalyst Fe/Phen@C-800_R generally showed more extended oxidic particles, while maintaining intact small iron clusters (Fig. 2C, S9 and S10D†).

The general morphology and distribution of Fe, N, O and C remained similar between the three different states of the catalyst as shown by the STEM data. Combined with the small changes in the electronic structure revealed by XPS, the data are consistent with the deactivation of the catalyst being due to fouling, which is removed by the H_2_O_2_ treatment, rather than by a structural change of the catalyst itself.

## Conclusions

In conclusion, here we report the first iron-based heterogeneous catalyst for cyclopropanation reactions. While in the past, this class of non-noble metal catalysts was generally employed for reduction or oxidation reactions, they are indeed effective catalysts also for carbene transfer reactions. The developed protocol allows obtaining several cyclopropanes from aromatic and aliphatic olefins and different diazo compounds in a practical and efficient manner. The heterogeneous nature of the catalyst and its robustness make it also an ideal candidate for flow chemistry applications. The deactivation of the catalyst has also been studied and an oxidative regeneration protocol was effectively developed, which may be of more general use even for other reactions.

## Conflicts of interest

There are no conflicts to declare.

## Supplementary Material

Supplementary information
