# Supplementary material for: Iron/N-doped graphene nano-structured catalysts for general cyclopropanation of olefins
Source: Chem Sci. 2020 Jun 8;11(24):6217–21. doi: 10.1039/d0sc01650k (PMC7480268; doi:10.1039/d0sc01650k)
Supplement: Supplementary file 1 [file SC-011-D0SC01650K-s001.pdf]

Electronic Supplementary Material (ESI) for Chemical Science.
This journal is © The Royal Society of Chemistry 2020

Electronic Supplementary Information for:

Iron/N-doped Graphene nano-structured Catalysts for General Cyclopropanation of Olefins

Abhijnan Sarkar, Francesco Ferretti, Fabio Ragaini

Dipartimento di Chimica - Università degli Studi di Milano, Via C. Golgi 19, 20133 Milano (Italy)

Dario Formenti, Carsten Kreyenschulte, Stephan Bartling, Kathrin Junge, Matthias Beller

Leibniz-Institut für Katalyse, Albert-Einstein-Straße 29a, 18059 Rostock (Germany)

Corresponding authors:

Fabio Ragaini – E-Mail: fabio.ragaini@unimi.it

Matthias Beller – E-Mail: matthias.beller@catalysis.de

Table of content

1. **General aspects** 3
2. **Analytical details** 3
3. **Procedure for catalyst preparation** 3
4. **Procedure for catalytic reactions and product isolation** 4
5. **Procedure for catalyst recycling and regeneration** 4
6. **Catalytic data: reaction optimisation** 5
7. **Catalysts characterisation** 7
8. **Characterisation of the reaction products** 14
9. **References** 73

1. General aspects

Unless otherwise stated, all the catalytic reactions were performed under N_2 atmosphere using distilled solvents. Ethyl diazoacetate (EDA, Sigma Aldrich) was stored at 4-8 °C (purity 97%, as indicated by the certificate of analysis for the specific lot). α-Methyl styrene was purchased from Acros Organics and purified by distillation over calcium hydride prior of its use in catalysis. All other substrates were purchased from various suppliers and used without any purification. Employed solvents were purified using standard techniques.

For the preparation of the catalytic materials, absolute ethanol was purchased from Alfa Aesar, $Fe(OAc)_2$ was purchased from Sigma Aldrich (trace metal basis >99.999%) and 1,10-phenanthroline (Phen) was purchased as monohydrate from Alfa Aesar. However, before use, it was dissolved in distilled CH_2Cl_2, dried over Na_2SO_4 followed by filtration under a dinitrogen atmosphere and evaporation of the solvent in vacuo. Both $Fe(OAc)_2$ and Phen were weighed in the air but stored under Ar to avoid oxidation and water uptake. Vulcan XC 72R, Aerosil OX 50 and MgO (nanopowder, > 99% trace metals basis) were purchased from Cabot Corporation, Evonik Industries and Alfa Aesar, respectively.

Diazo compounds **3b**,[1] **3c**,[2] **3d**[3] were prepared according to literature procedures.

2. Analytical details

^{1}H NMR and ^{13}C NMR spectra were recorded at room temperature (at frequencies of 300 MHz for the proton and 75 MHz for the carbon) on a Bruker AC 300 FT or on a Bruker Avance DRX 400 (at frequencies of 400 MHz for the proton and 100 MHz for the carbon). Chemical shifts are reported in ppm relative to TMS; the data are reported as follows: proton multiplicities (s = singlet, d = doublet, t = triplet, q = quartet, m = multiplet and br = broad), coupling constants and finally integration.

IR spectra were registered on a Varian Scimitar FTS-1000.

The scanning transmission electron microscopy (STEM) measurements were performed at 200kV with a probe aberration-corrected JEM-ARM200F (JEOL, Corrector: CEOS). The microscope is equipped with a JED-2300 (JEOL) energy-dispersive x-ray-spectrometer (EDXS) and an Enfinium ER (GATAN) electron energy loss spectrometer (EELS) with Dual EELS for chemical analysis. The solid samples were deposed without any pretreatment on holey carbon supported by a Cu-grid (mesh 300) and transferred to the microscope.

The XPS (X-ray Photoelectron Spectroscopy) measurements were performed on an ESCALAB 220iXL (Thermo Fisher Scientific) with monochromated Al Kα radiation (E = 1486.6 eV). Samples are prepared on a stainless steel holder with conductive double sided adhesive carbon tape. The electron binding energies were obtained with charge compensation using a flood electron source and referenced to the C1s core level of carbon at 284.8 eV (C-C and C-H bonds). For quantitative analysis, the peaks were deconvoluted with Gaussian-Lorentzian curves using the software Unifit 2017. The peak areas were normalized by the transmission function of the spectrometer and the element specific sensitivity factor of Scofield.[4]

3. Procedure for catalyst preparation

For the catalyst preparation we followed a procedure published by some of us.[5] In order to obtain 1 g of the final catalytically active material, 94 mg of $Fe(OAc)_2$ (0.5 mmol) were dissolved in 20 mL of absolute ethanol. Then, 291 mg of Phen (1.5 mmol) were added and the as obtained brown solution was stirred at 60 °C for 2 hours. Subsequently, the support (Vulcan® XC-72R, Aerosil OX 50 or MgO, 615 mg) was added portionwise. The heterogeneous mixture was maintained under stirring for 18 h at room temperature (23 °C). Then, ethanol was carefully removed by rotary evaporation and the solid material was dried for at least 4 hours under vacuum. The as obtained solid was transferred into a crucible equipped with lid and placed in the pyrolysis chamber of an AUSTROMAT 264 pyrolytic oven. The oven was heated to 800 °C for 2 h (temperature ramp of 25 K/min) flushing 10 mL·min^{-1} of Ar. Once the heating was stopped, the chamber was allowed to cool down to room temperature (Ar was flushed until the temperature reached 50 °C). The as

obtained catalysts are referred as Fe/Phen@C-800, Fe/Phen@SiO_2-800, and Fe/Phen@MgO-800 respectively in the whole text. A ~3% mass loss was observed after the thermolysis step.

By using the same procedure, catalyst without the addition of either Phen or $Fe(OAc)_2$ were prepared (they are referred to as Fe@C-800 and Phen@C-800, respectively).

4. Procedure for catalytic reactions and product isolation

In a typical catalytic reaction, the catalyst (18.5 mg) was placed in an oven dried Schlenk tube which was evacuated and backfilled with nitrogen. Solvent (2 mL) was added followed by α-methyl styrene (2.50 mmol) and a stock solution of EDA (0.5 mmol, 1 mL, [EDA] = 0.5 M,). The mixture was then heated at 60 ^{o}C and stirred for 4 hours under a N_2 atmosphere. Once the reaction was completed, the internal standard (2,4-dinitrotoluene) was added the catalyst was removed by filtration over Celite. The solvent was evaporated, the crude mixture dissolved in $CDCl_3$ and analysed by means of NMR and IR spectroscopy to determine conversion, yield and diastereomeric ratio. Isolated compounds were obtained by purification by column chromatography on silica gel. In the following, all compounds have been drawn as a single enantiomer for simplicity, but they are obviously racemic mixtures. The 15-fold scaled-up experiment was performed under exactly the same conditions, simply scaling all reagents and solvent (1,2-dimethoxyethane) by the same ratio and performing the reaction in a 100 mL Schlenk flask instead of a tube. All reported yields are calculated based on the initial amount of EDA.

5. Procedure for catalyst recycling and regeneration

After completion of the reaction, the reaction mixture was completely transferred to a centrifuge tube and the spent catalyst was separated by centrifugation at 6000 rpm for 5-10 minutes. Isolated solid material was washed with dichloromethane (3x10 mL), separated by centrifugation again, dried under vacuum (8 h) and used for another cycle under the reaction conditions described in Paragraph 4.

The thermal reactivation of the spent catalyst was performed under dinitrogen for 3 h at 300 or 400 °C.

Oxidative reactivation was performed using H_2O_2. Specifically, the spent catalyst was stirred with an aqueous solution of H_2O_2 (3 %) at room temperature for 16 h. Afterwards, the mixture was centrifuged (6000 rpm for 5-10 minutes) and the isolated solid was washed with distilled water (3x10 mL), dried for 8 h under vacuum and reused in a catalytic run.

The spent and regenerated catalysts are referred as Fe/Phen@C-800_S and Fe/Phen@C-800_R, respectively.

Table S1. Elemental compositions of the fresh, deactivated and reactivated Fe-based catalysts based on CHN and AAS Fe analysis.

	**C [wt.%]**	**H [wt.%]**	**N [wt.%]**	**Fe [wt.%]**
**Fe/Phen@C-800**	64.77	0.45	3.85	2.95
**Fe/Phen@C-800_S**	61.31	3.23	1.35	2.07
**Fe/Phen@C-800_R**	50.65	3.02	0.76	2.27
**Fe/Phen@MgO-800**	19.03	0.13	0.84	3.31
**Fe/Phen@SiO_2-800**	20.97	0.08	1.09	3.77

6. Catalytic data: reaction optimization

Table S2. Cyclopropanation of **1a** with Fe/Phen@C-800 as catalyst: effect of solvent and control experiments.[a]

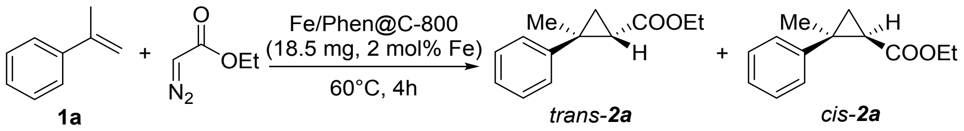

Entry	Solvent	Diethylfumarate yield [%][b]	Diethylmaleate yield [%][b]	2a yield [%][b]	*trans*:*cis* ratio[b]
1	Benzene	traces	3	96	72:28
2	1,2-Dichloroethane	-	1	96	79:21
3	*n*-Hexane	1	5	84	74:26
4	CH_3CN	-	1	89	75:25
**5**	**1,2-Dimethoxyethane**	**-**	**>1**	**99**	**74:26**
6[c]	1,2-Dimethoxyethane	<1	<1	<1	-
7[d]	1,2-Dimethoxyethane	<1	<1	<1	-
8[e]	1,2-Dimethoxyethane	<1	<1	<1	-

[a] Reaction conditions: 0.50 mmol EDA, 2.50 mmol **1a**, 18.5 mg Fe/Phen@C-800 (corresponding to 2 mol% Fe), 3 mL solvent, at 60 °C for 4h. [b] Determined by ^{1}H NMR spectroscopy (2,4-dinitrotoluene as an internal standard). [c] The reaction was conducted using Fe@C-800 as catalyst. [d] The reaction was conducted using Phen@C-800 as catalyst. [e] The reaction was conducted using $Fe(OAc)_2$ (1.7 mg, 1,0 10^{-2} mmol) + Phen (3.8 mg, 2.1 10^{-2} mmol) as catalyst.

Table S3. Cyclopropanation of **1a** with Fe/Phen@C-800 as catalyst: effect of temperature.[a]

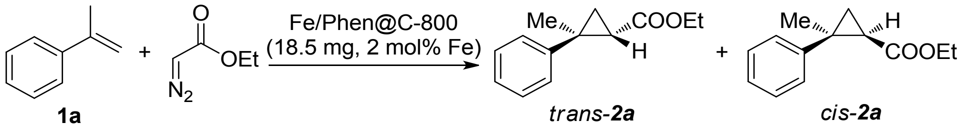

Entry	Temperature [° C]	Time [h]	Diethylfumarate yield [%][b]	Diethylmaleate yield [%][b]	2a yield [%]	*trans*:*cis* ratio
1	50	4	-	1	98	73:27
2	40	4	-	2	92	75:25
3	40	6	-	3	97	74:26
4	70	4	-	2	97	71:29

[a] Reaction conditions: 0.50 mmol EDA, 2.50 mmol **1a**, 18.5 mg Fe/Phen@C-800 (corresponding to 2 mol% Fe), 3 mL DME. [b] Determined by ^{1}H NMR spectroscopy (2,4-dinitrotoluene as an internal standard).

Table S4. Cyclopropanation of **1a** with Fe/Phen@C-800 as catalyst: effect of catalyst and olefin loading.[a]

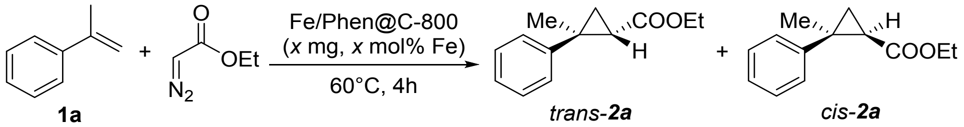

Entry	Cat. loading [mg]	Olefin loading (eq)	Diethylfumarate yield [%][b]	Diethylmaleate yield [%][b]	2a yield [%]	***trans*:*cis* ratio**
1	14.9	5	-	>1	85	73:27
2	9.8	5	-	1	71	74:26
3	18.5	1.5	2	10	86	70:30
4[c]	18.5	5	-	-	71	73:27

[a] Reaction conditions: 0.50 mmol EDA, 2.50 mmol **1a**, x mg Fe/Phen@C-800 (corresponding to x(2/18.5) mol% Fe), 3 mL DME. [b] Determined by ^{1}H NMR spectroscopy (2,4-dinitrotoluene as internal standard). [c] Water was added (10 v/v%)

Scheme S1 Cyclopropanation of **1a** with different Fe-catalysts under optimised conditions: effect of the support.[a]

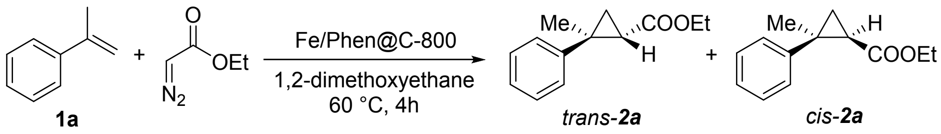

	yield	-	*trans*/*cis*
Fe/Phen@C-800	99%	-	74:26
Fe/Phen@MgO-800	98%	-	76:24
Fe/Phen@SiO_2-800	97%	-	76:24

[a] Reaction conditions: 0.50 mmol EDA, 2.50 mmol **1a**, 18.5 mg Fe/Phen@support-800, 3 mL solvent, at 60 °C for 4h. Yields and d.r. determined by ^{1}H NMR spectroscopy (2,4-dinitrotoluene as an internal standard).

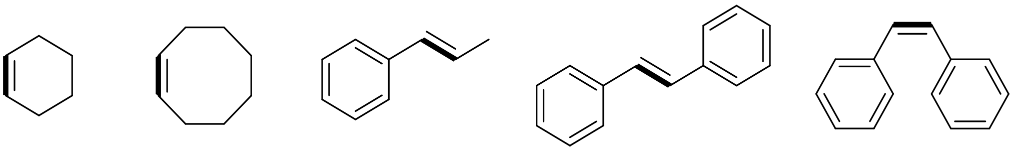

[a] Reaction conditions: 0.50 mmol EDA, 2.50 mmol **1a**, 18.5 mg Fe/Phen@C-800 (corresponding to 2 mol% Fe), 3 mL 1,2-dimethoxyethane, at 60 °C for 8h.

Figure S1. Examples of internal olefins unreactive under optimised reaction conditions[a]

7. Catalysts characterisation

a. XPS

Table S5. Elemental compositions (near-surface region) for the fresh (Fe/Phen@C-800), spent (Fe/Phen@C-800_S) and reactivated (Fe/Phen@C-800_R) Fe-based catalysts based on XPS quantitative analysis.

	**C [at.%]**	**O [at.%]**	**N [at.%]**	**Fe [at.%]**
**Fe/Phen@C-800**	87.4	3.5	7.6	0.56
**Fe/Phen@C-800_S**	85.6	3.4	9.9	0.54
**Fe/Phen@C-800_R**	85.6	3.8	9.2	0.79

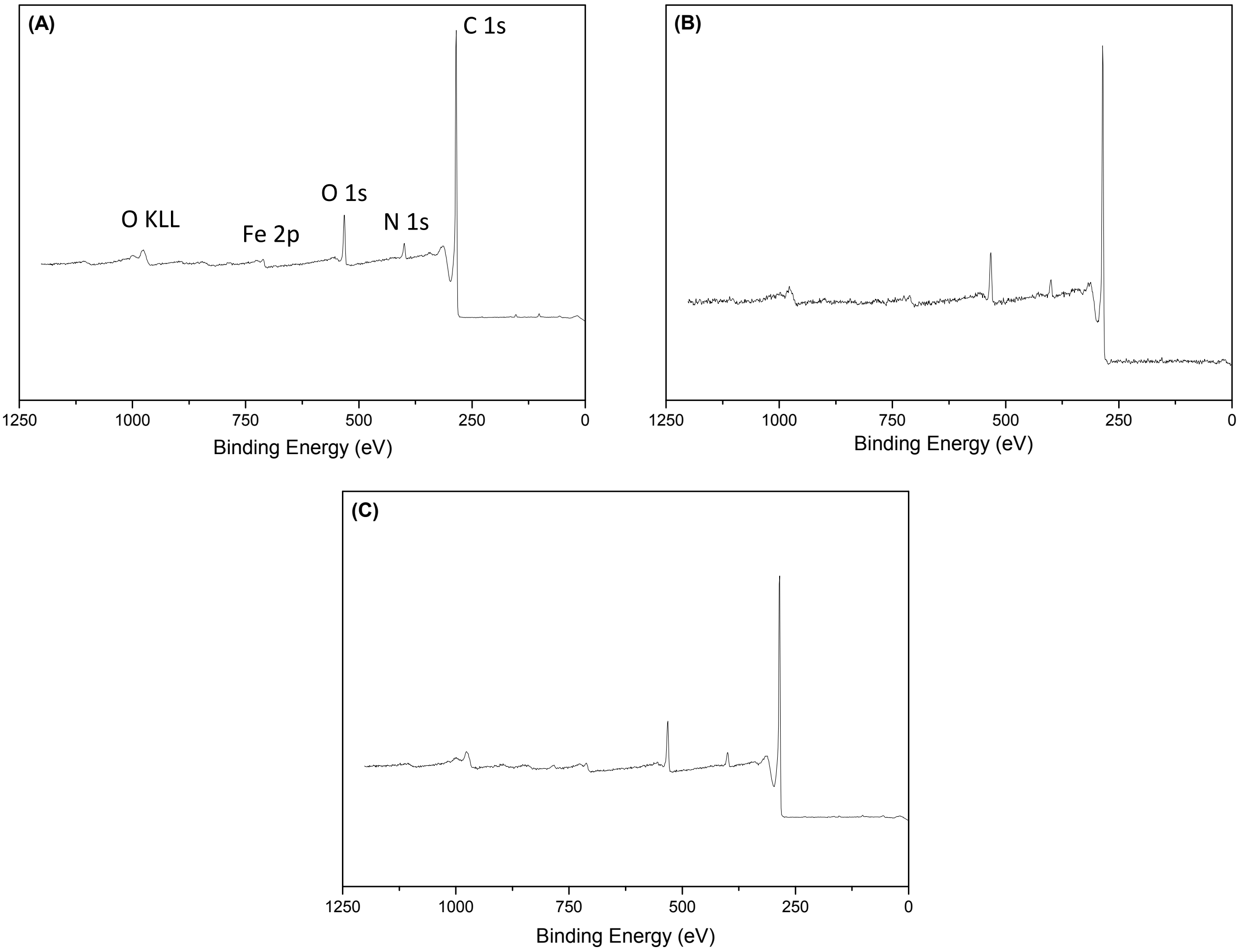

Figure S2. XPS analyses (survey) of Fe/Phen@C-800 (**A**), Fe/Phen@C-800_S (**B**) and Fe/Phen@C-800_R (**C**).

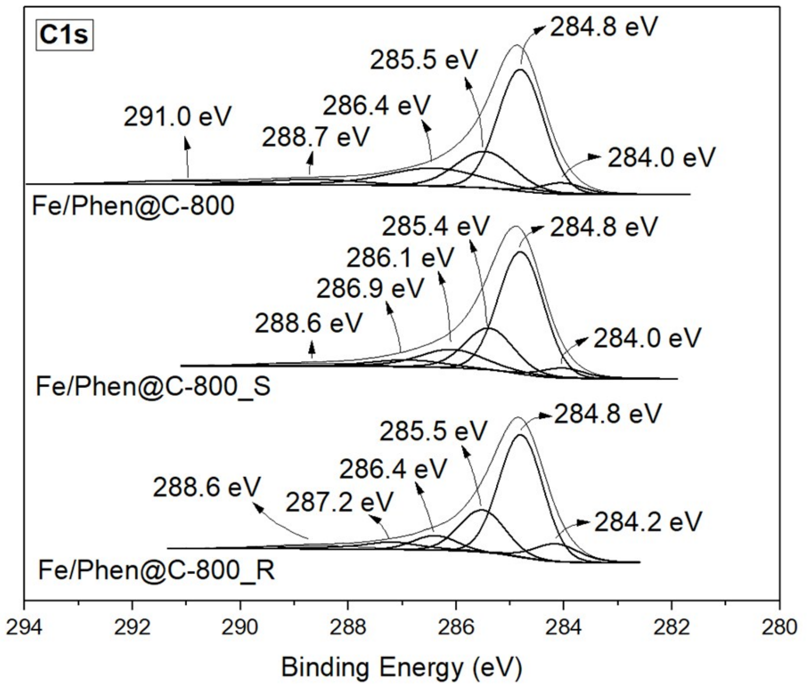

Figure S3. XPS analyses (C1s) of Fe/Phen@C-800, Fe/Phen@C-800_S and Fe/Phen@C-800_R.

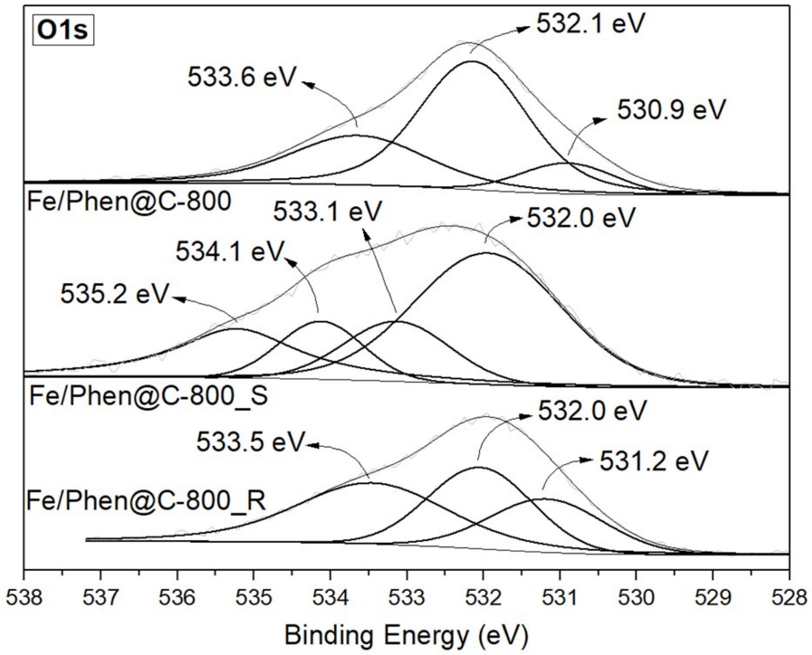

Figure S4. XPS analyses (O1s) of Fe/Phen@C-800, Fe/Phen@C-800_S and Fe/Phen@C-800_R.

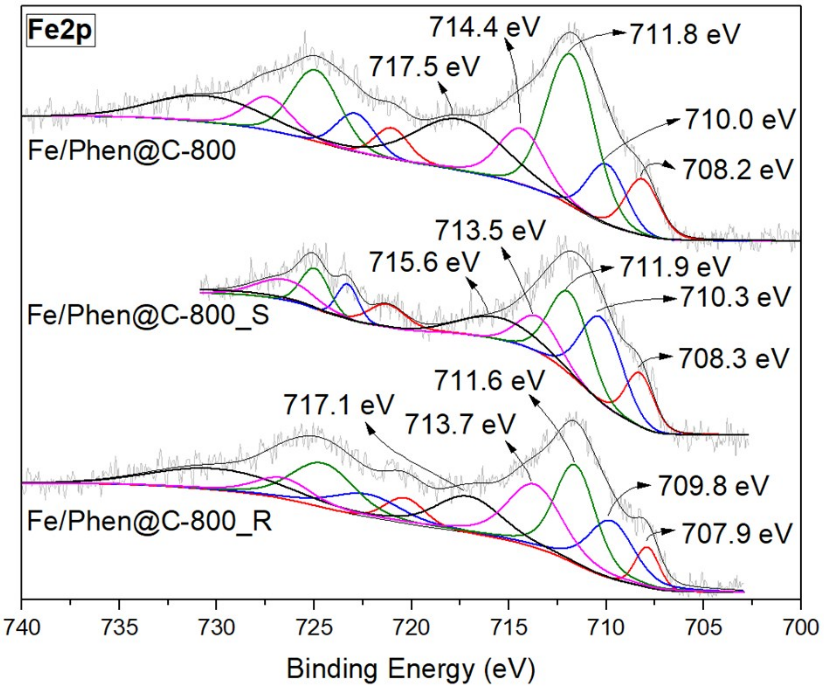

Figure S5. XPS analyses (Fe2p) of Fe/Phen@C-800, Fe/Phen@C-800_S and Fe/Phen@C-800_R.

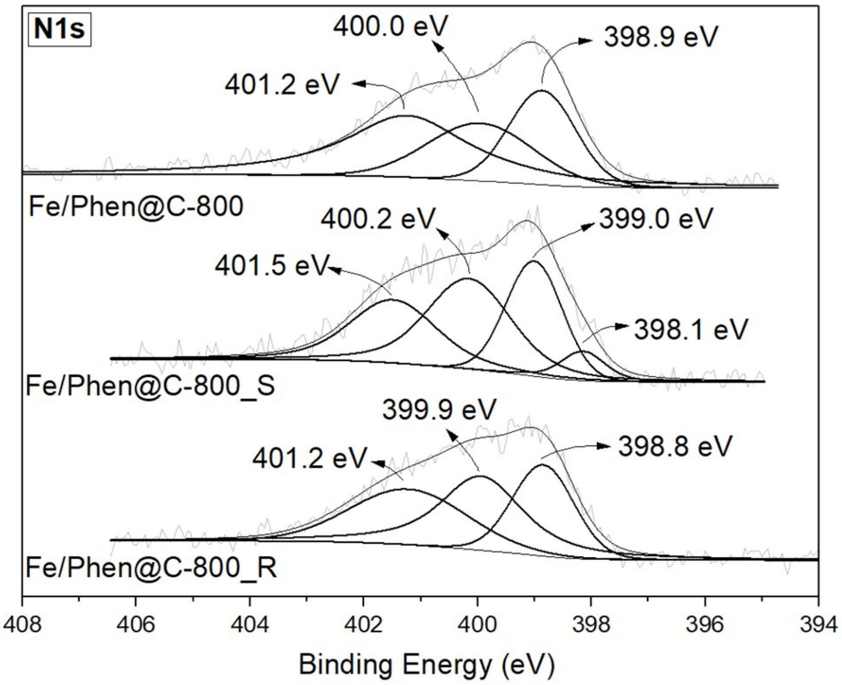

Figure S6. XPS analyses (N1s) of Fe/Phen@C-800, Fe/Phen@C-800_S and Fe/Phen@C-800_R.

b. STEM

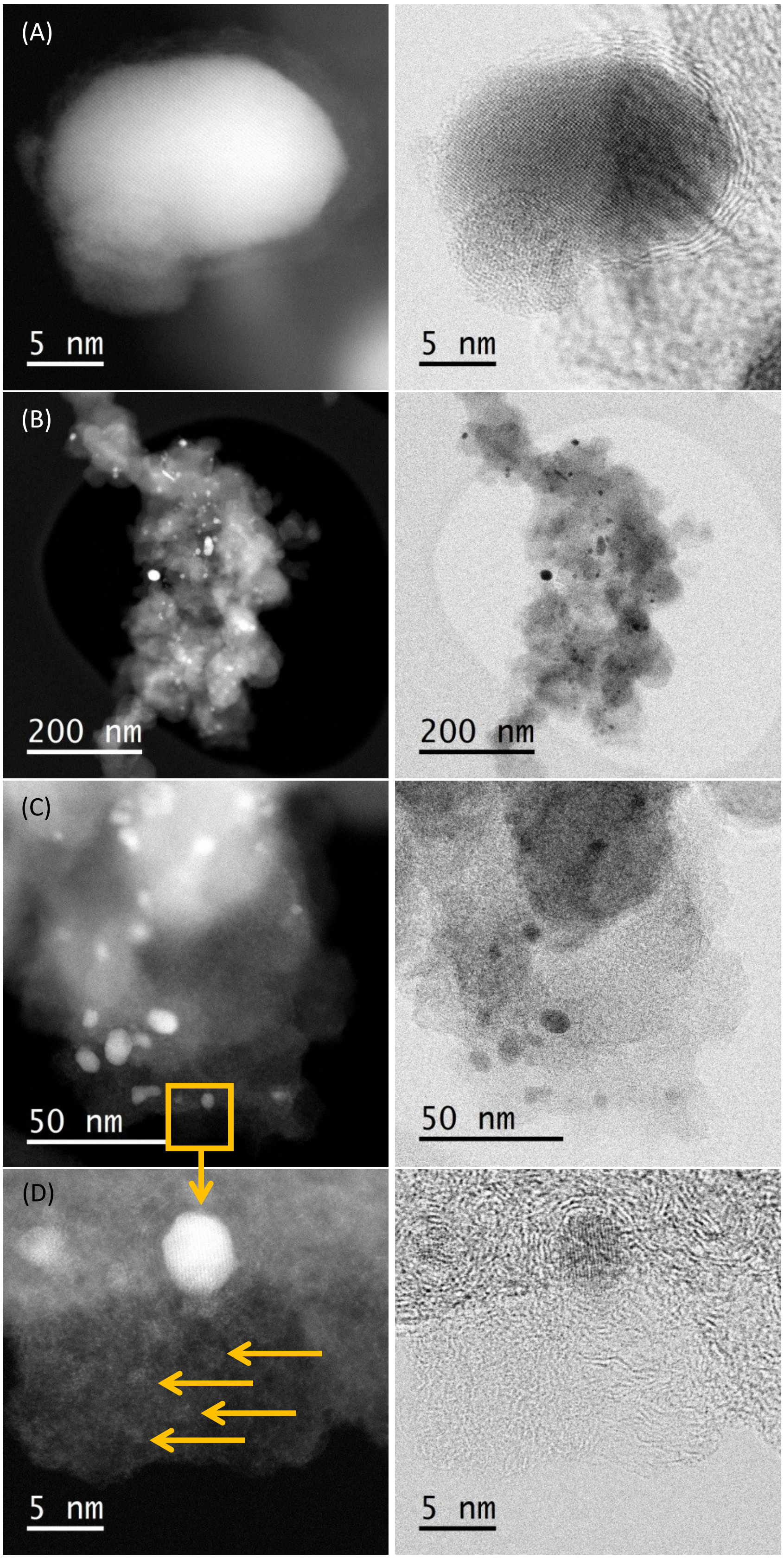

Figure S7. HAADF- (left column) and ABF- (right column) STEM images of Fe/Phen@C-800 (A) showing high resolution images of a metallic Fe particle accompanied by a small Fe oxide particle (see also Fig. 2A), the ABF image on the right highlights the enclosure of the metallic particle by some graphene layers. Line (B) shows a representative overview of an agglomeration of some support particles with their Fe particles, line (C) a more detailed view of the edge regions of some support particles and (D) high resolution images of the mixed C, N, O phase containing Fe clusters (some marked by arrows, see also Fig. S10B).

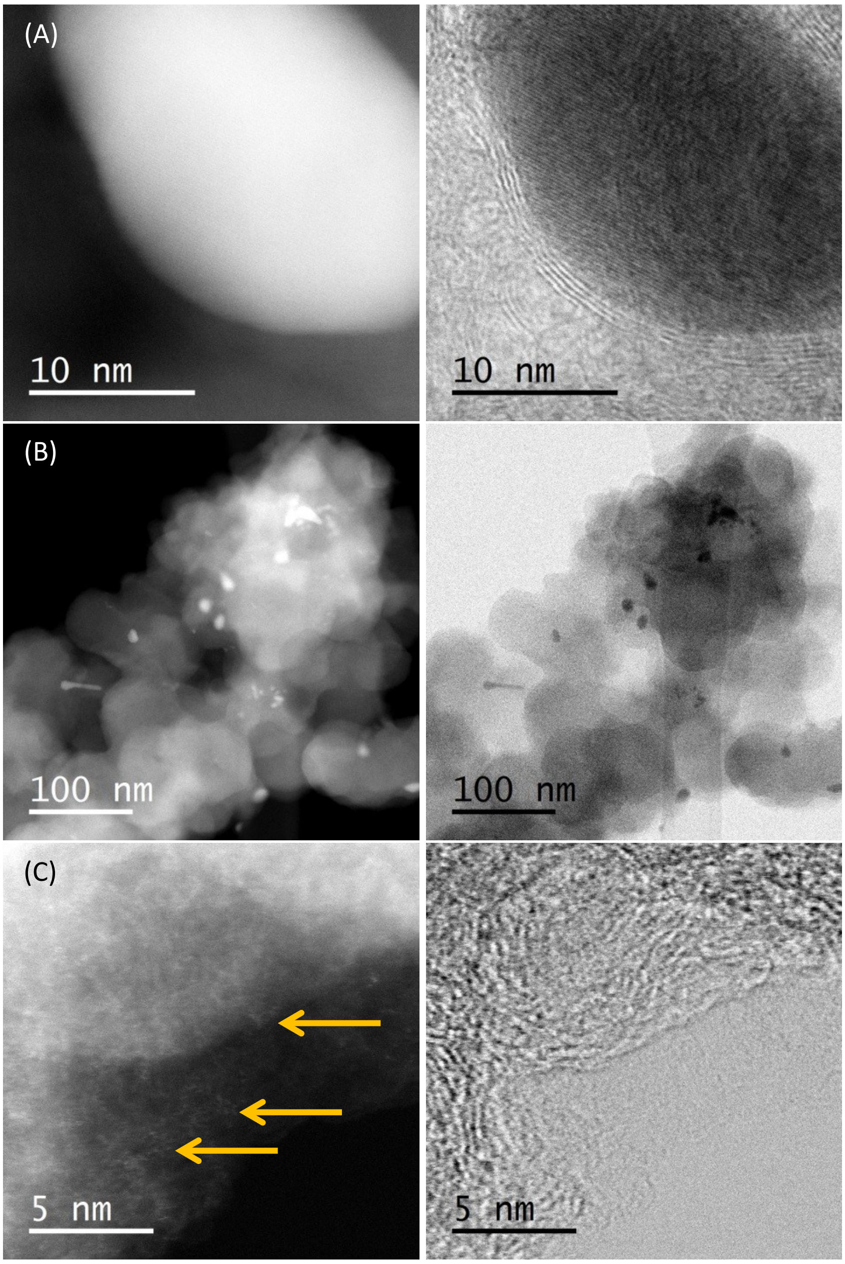

Figure S8. HAADF- (left column) and ABF- (right column) STEM images of Fe/Phen@C-800_S. Line (A) showing a carbon enclosed metallic Fe particle, line (B) a general overview (corresponding to the area of Fig. 2B) and line (C) a high resolution image of the C, N, O phase with its Fe clusters.

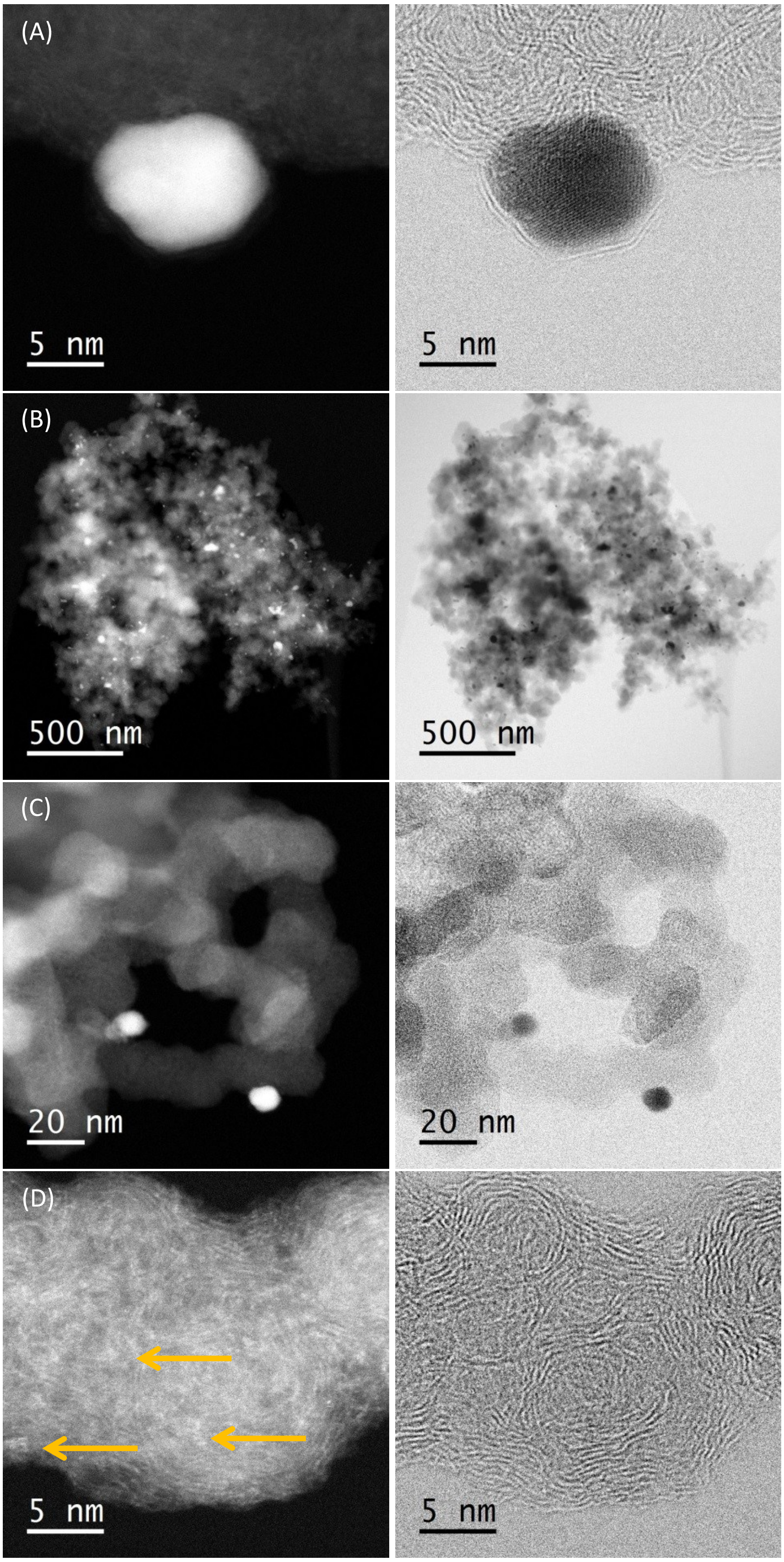

Figure S9. HAADF- (left column) and ABF- (right column) STEM images of Fe/Phen@C-800_R. Line (A) showing a carbon enclosed metallic Fe particle, line (B) a representative general overview of agglomerated support particles with their Fe particles. Line (C) a close up of the edge of such an agglomeration and line (D) a high resolution image of the C, N, O phase with its Fe clusters.

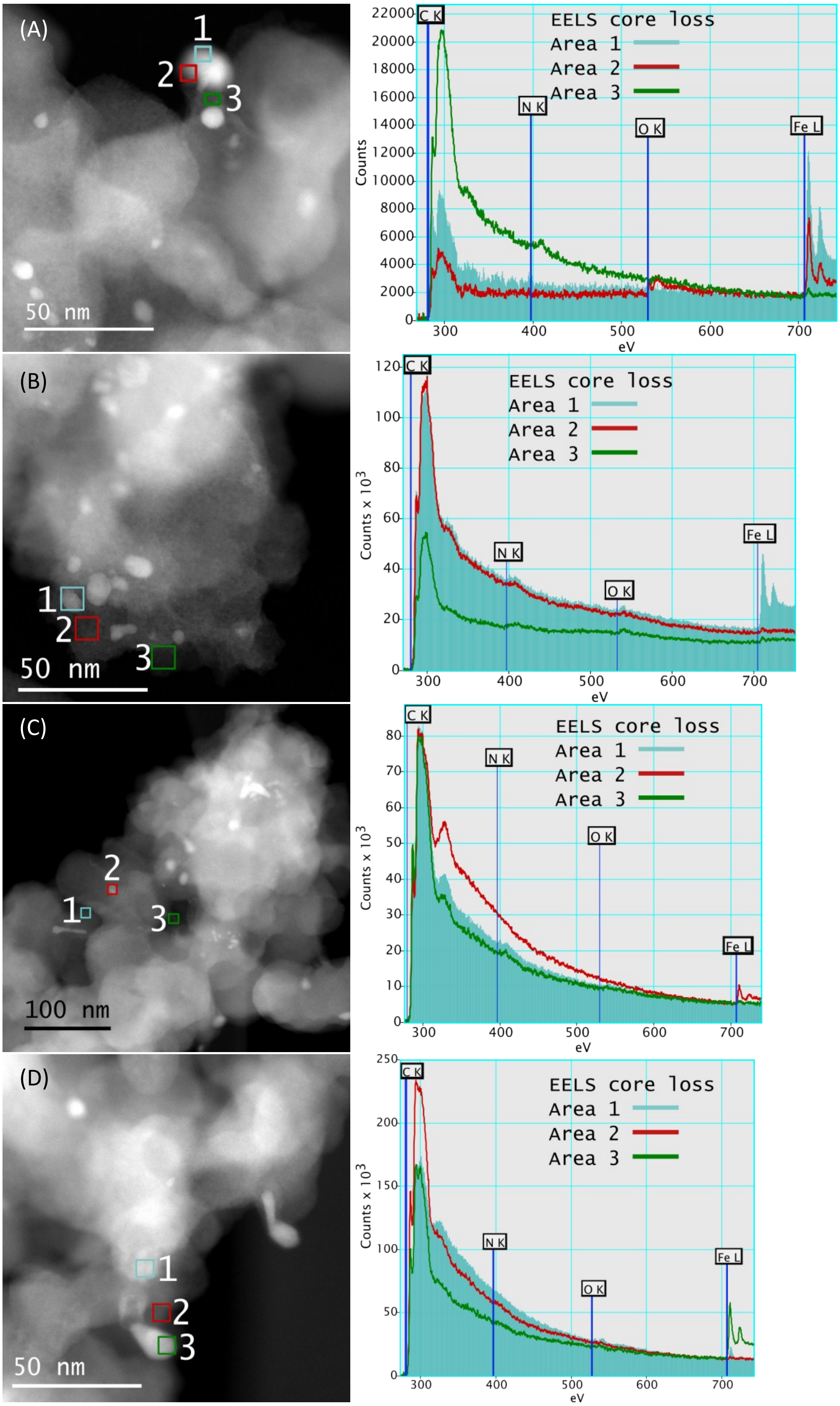

Figure S10 ADF-STEM images and EELS spectra from indicated areas of Fe/Phen@C-800 (A and B), Fe/Phen@C-800_S (C) and Fe/Phen@C-800_R (D). Lines (A), (C) and (D) show exemplary spectra of the spectrum imaging datasets used to calculate the corresponding elemental maps in Fig. 2. They also show a certain N and Fe content besides the carbon in areas close to the surface of the support particles. Area 3 in line (B) shows the spectrum of the region also shown in high resolution in Figure S7D, verifying the presence of Fe at this position and supporting the interpretation of the clusters with brighter contrast there to be of Fe. All spectra have been background subtracted and deconvolved.

8. Characterisation of the reaction products

trans-Ethyl 2-methyl-2-phenylcyclopropane-1-carboxylate (2a)

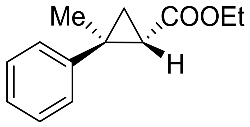

trans-

Only the *trans* isomer was isolated in a pure form.

Obtained as colourless oil after column chromatography (eluent hexane + 0.2 % AcOEt). Yield 68%.

^{1}H NMR (400 MHz $CDCl_3$) δ 7.30 (d, J = 4.3 Hz, 4H), 7.24 – 7.18 (m, 1H), 4.20 (qd, J = 7.1, 2.6 Hz, 2H), 2.04 – 1.92 (m, 1H), 1.53 (s, 3H), 1.43 (dq, J = 8.3, 4.7 Hz, 2H), 1.30 ppm (t, J = 7.1 Hz, 3H).

^{13}C NMR (101 MHz, $CDCl_3$) δ 172.17, 145.93, 128.43, 127.29, 126.43, 60.49, 30.55, 27.88, 20.78, 19.88, 14.41 ppm.

Data are in accordance with the one reported in the literature.[6]

Ethyl 2-phenylcyclopropane-1-carboxylate (2b)

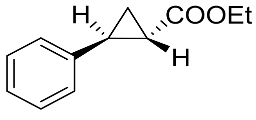

trans-

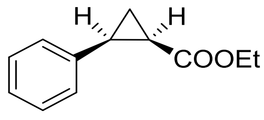

cis-

Obtained as yellow oil (*trans*) and colourless oil (*cis*) after column chromatography (eluent hexane + 0.2 % AcOEt). Yield *trans* 69%, *cis* 27% (*trans*:*cis* = 72:28).

For *trans* isomer:

^{1}H NMR (400 MHz, $CDCl_3$) δ 7.29 (d, J = 7.1 Hz, 2H), 7.24 – 7.17 (m, 1H), 7.15 – 7.05 (m, 2H), 4.17 (q, J = 7.1 Hz, 2H), 2.52 (ddd, J = 9.4, 6.5, 4.2 Hz, 1H), 1.90 (ddd, J = 8.4, 5.3, 4.2 Hz, 1H), 1.67 – 1.51 (m, 1H), 1.34 – 1.30 (m, 1H), 1.28 ppm (t, J = 7.1 Hz, 3H).

^{13}C NMR (101 MHz, $CDCl_3$) δ 173.40, 140.13, 128.45, 126.45, 126.17, 60.69, 26.16, 24.17, 17.04, 14.26 ppm.

For *cis* isomer:

^{1}H NMR (400 MHz, $CDCl_3$) δ 7.27 (s, 4H), 7.22 – 7.15 (m, 1H), 3.87 (q, J = 7.1 Hz, 2H), 2.58 (q, J = 8.7 Hz, 1H), 2.08 (ddd, J = 9.1, 7.9, 5.7 Hz, 1H), 1.72 (dt, J = 7.4, 5.4 Hz, 1H), 1.33 (td, J = 8.2, 5.1 Hz, 1H), 0.97 ppm(t, J = 7.1 Hz, 3H).

^{13}C NMR (101 MHz, $CDCl_3$) δ 170.96, 136.57, 129.29, 127.86, 126.62, 60.15, 25.45, 21.81, 14.02, 11.10 ppm.

Data are in accordance with[7] and[8] for *trans* and *cis* isomers, respectively.

Ethyl 2-(*o*-tolyl) cyclopropane-1-carboxylate (2c)

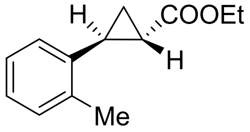

trans-

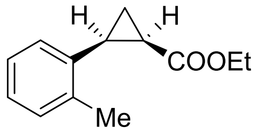

cis-

Obtained as colourless oil (*trans*) and colourless oil (*cis*) after column chromatography (eluent hexane + 0.2 % AcOEt). Yield *trans* 73%, *cis* 21% (*trans*:*cis* = 77:23).

For *trans* isomer:

^{1}H NMR (400 MHz, $CDCl_3$) δ 7.15 (tt, J = 6.4, 3.3 Hz, 3H), 7.03 – 6.96 (m, 1H), 4.20 (qd, J = 7.1, 2.4 Hz, 2H), 2.52 (ddd, J = 9.1, 6.9, 4.6 Hz, 1H), 2.39 (s, 3H), 1.84 – 1.73 (m, 1H), 1.67 – 1.52 (m, 2H), 1.30 ppm (t, J = 7.1 Hz, 4H, overlap of -CH_3 signal with that of one proton of the cyclopropane scaffold).

^{13}C NMR (101 MHz, $CDCl_3$) δ 173.90, 138.02, 137.85, 129.85, 126.71, 125.89, 125.85, 60.66, 24.64, 22.35, 19.57, 15.37, 14.34 ppm.

For *cis* isomer:

^{1}H NMR (400 MHz, $CDCl_3$) δ 7.22 – 7.18 (m, 1H), 7.13 (dd, J = 5.8, 3.5 Hz, 3H), 3.85 (q, J = 7.1 Hz, 2H), 2.45 (q, J = 8.4 Hz, 1H), 2.34 (s, 3H), 2.20 – 2.10 (m, 1H), 1.75 (dt, J = 7.5, 5.2 Hz, 1H), 1.35 (td, J = 8.2, 5.0 Hz, 1H), 0.93 ppm (t, J = 7.1 Hz, 3H).

^{13}C NMR (101 MHz, $CDCl_3$) δ 138.15, 129.39, 129.11, 126.76, 125.34, 60.05, 24.44, 21.16, 19.33, 13.93, 11.22 ppm.

Data are in accordance with the one reported in the literature.[6]

Ethyl 2-(*m*-tolyl) cyclopropane-1-carboxylate (2d)

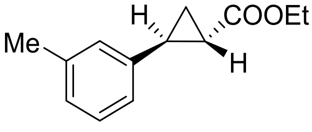

trans-

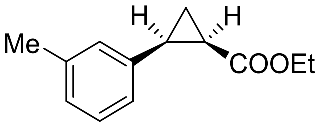

cis-

Obtained as yellow oil (*trans*) and colourless oil (*cis*) after column chromatography (eluent hexane + 0.2 % AcOEt). Yield *trans* 70%, *cis* 27% (*trans*:*cis* = 72:28).

For *trans* isomer:

^{1}H NMR (400 MHz, $CDCl_3$) δ 7.17 (t, J = 7.6 Hz, 1H), 7.02 (d, J = 7.5 Hz, 1H), 6.95 – 6.86 (m, 2H), 4.17 (q, J = 7.1 Hz, 2H), 2.55 – 2.43 (m, 1H), 2.33 (s, 3H), 1.96 – 1.84 (m, 1H), 1.58 (dt, J = 9.5, 4.9 Hz, 1H), 1.28 ppm (t, J = 7.1 Hz, 4H, overlap of -CH_3 signal with that of one proton of the cyclopropane scaffold).

^{13}C NMR (101 MHz, $CDCl_3$) δ 173.45, 140.07, 138.09, 128.36, 127.22, 126.99, 123.16, 60.62, 26.14, 24.10, 21.36, 16.99, 14.27 ppm.

For *cis* isomer:

^{1}H NMR (400 MHz, $CDCl_3$) δ 7.15 (t, J = 7.5 Hz, 1H), 7.11 – 7.04 (m, 2H), 7.00 (d, J = 7.4 Hz, 1H), 3.89 (q, J = 7.1 Hz, 2H), 2.54 (q, J = 8.6 Hz, 1H), 2.31 (s, 3H), 2.06 (ddd, J = 9.2, 7.9, 5.7 Hz, 1H), 1.69 (dt, J = 7.4, 5.5 Hz, 1H), 1.38 – 1.22 (m, 1H), 0.98 ppm (t, J = 7.1 Hz, 3H).

^{13}C NMR (101 MHz, $CDCl_3$) δ 171.02, 137.32, 136.48, 130.12, 127.75, 127.39, 126.29, 60.12, 25.40, 21.70, 21.34, 14.03, 11.10 ppm.

Data are in accordance with the one reported in the literature.[6]

Ethyl 2-(*p*-tolyl) cyclopropane-1-carboxylate (2e)

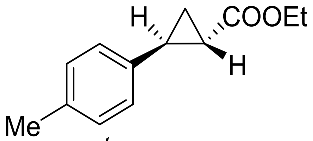

trans-

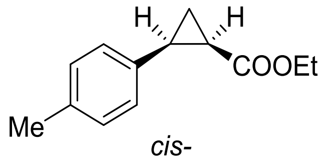

cis-

Obtained as colourless oil (*trans*) and colourless oil (*cis*) after column chromatography (eluent hexane + 0.2 % AcOEt). Yield *trans-* 72%, *cis* 18% (*trans*:*cis* = 80:20).

For *trans* isomer:

^{1}H NMR (400 MHz, $CDCl_3$) δ 7.09 (d, J = 7.9 Hz, 2H), 7.00 (d, J = 8.0 Hz, 2H), 4.17 (q, J = 7.1 Hz, 1H), 2.50 (ddd, J = 9.7, 6.4, 4.3 Hz, 1H), 2.32 (s, 3H), 1.87 (dt, J = 9.1, 4.7 Hz, 1H), 1.58 (dt, J = 9.4, 4.7 Hz, 1H), 1.28 ppm (t, J = 7.3 Hz, 4H, overlap of $-CH_3$ signal with that of one proton of the cyclopropane scaffold).

^{13}C NMR (101 MHz, $CDCl_3$) δ 173.49, 137.06, 136.06, 129.13, 126.12, 60.63, 25.93, 24.05, 20.99, 16.92, 14.28 ppm.

For *cis* isomer:

^{1}H NMR (400 MHz, $CDCl_3$) δ 7.15 (d, J = 7.9 Hz, 2H), 7.06 (d, J = 7.9 Hz, 2H), 3.89 (q, J = 7.1 Hz, 2H), 2.54 (q, J = 8.2 Hz, 1H), 2.30 (s, 3H), 2.14 – 1.96 (m, 1H), 1.80 – 1.62 (m, 1H), 1.39 – 1.20 (m, 1H), 1.01 ppm (t, J = 7.1 Hz, 3H).

^{13}C NMR (101 MHz, $CDCl_3$) δ 129.13, 128.59, 60.14, 25.20, 21.68, 14.07, 11.19, 1.01 ppm. Three quaternary C were not detected.

Data are in accordance with the one reported in the literature.[6]

Ethyl 2-(4-(*tert*-butyl) phenyl) cyclopropane-1-carboxylate (2f)

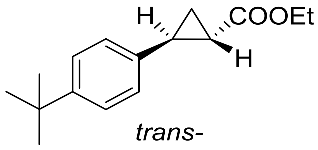

trans-

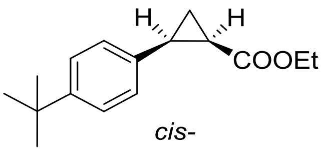

cis-

Obtained as yellow oil (*trans*) and pale-yellow oil (*cis*) after column chromatography (eluent hexane + 0.2 % AcOEt). Yield *trans-* 67%, *cis* 30% (*trans*:*cis* = 69:31).

For *trans* isomer:

^{1}H NMR (400 MHz, $CDCl_3$) δ 7.31 (d, J = 8.4 Hz, 2H), 7.04 (d, J = 8.3 Hz, 2H), 4.16 (q, J = 7.1 Hz, 2H), 2.49 (ddd, J = 9.4, 6.5, 4.2 Hz, 1H), 1.88 (ddd, J = 8.4, 5.2, 4.2 Hz, 1H), 1.58 (dt, J = 9.3, 4.9 Hz, 1H), 1.30 (s, 9H), 1.27 ppm (t, J = 7.2 Hz, 4H, overlap of $-CH_3$ signal with that of one proton of the cyclopropane scaffold).

^{13}C NMR (101 MHz, $CDCl_3$) δ 173.50, 149.46, 137.10, 125.84, 125.37, 60.62, 31.33, 25.85, 24.12, 16.95, 14.27 ppm.

For *cis* isomer:

^{1}H NMR (400 MHz, $CDCl_3$) δ 7.28 (d, J = 8.3 Hz, 2H), 7.19 (d, J = 8.2 Hz, 2H), 3.87 (qd, J = 7.1, 5.1 Hz, 2H), 2.54 (q, J = 8.6 Hz, 1H), 2.05 (ddd, J = 9.2, 7.9, 5.7 Hz, 1H), 1.69 (dt, J = 7.4, 5.4 Hz, 1H), 1.29 (s, 10H, overlap of -$C(CH_3)_3$ signal with that of one proton of the cyclopropane scaffold), 0.92 ppm (t, J = 7.1 Hz, 3H).

^{13}C NMR (101 MHz, $CDCl_3$) δ 171.07, 149.39, 133.50, 128.90, 124.76, 60.09, 34.39, 31.34, 25.05, 21.84, 13.91, 11.13 ppm.

Data are in accordance with[7] and[9] for *trans* and *cis* isomer, respectively.

Ethyl 2-(4-methoxyphenyl) cyclopropane-1-carboxylate (2g)

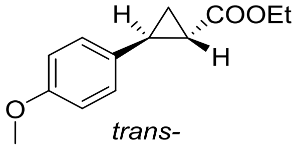

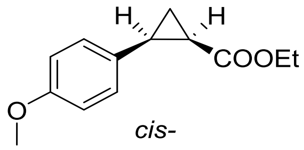

Obtained as white crystalline solid (*trans*) and light yellow solid (*cis*) after column chromatography (eluent hexane + 0.2 % AcOEt). Yield *trans* 69%, *cis*- 19% (*trans*:*cis* = 78:22).

For *trans* isomer:

^{1}H NMR (400 MHz, $CDCl_3$) δ 7.03 (d, J = 8.7 Hz, 2H), 6.82 (d, J = 8.7 Hz, 2H), 4.16 (q, J = 7.1 Hz, 2H), 3.78 (s, 3H), 2.48 (ddd, J = 10.3, 6.5, 4.2 Hz, 1H), 1.82 (dt, J = 9.2, 4.7 Hz, 1H), 1.55 (dt, J = 9.6, 4.9 Hz, 1H), 1.28 (t, J = 7.2 Hz, 3H), 1.24 ppm (dd, J = 5.2, 3.2 Hz, 1H).

^{13}C NMR (101 MHz, $CDCl_3$) δ 173.63, 158.34, 132.12, 127.40, 113.94, 60.69, 55.37, 25.68, 23.92, 16.80, 14.32 ppm.

For *cis* isomer:

^{1}H NMR (400 MHz, $CDCl_3$) δ 7.18 (d, J = 8.4 Hz, 2H), 6.80 (d, J = 8.7 Hz, 2H), 3.89 (q, J = 7.1 Hz, 2H), 3.77 (s, 3H), 2.52 (q, J = 8.6 Hz, 1H), 2.03 (ddd, J = 9.1, 7.9, 5.6 Hz, 1H), 1.65 (dt, J = 7.4, 5.4 Hz, 1H), 1.39 – 1.18 (m, 1H), 1.01 ppm (t, J = 7.1 Hz, 3H).

^{13}C NMR (101 MHz, $CDCl_3$) δ 171.11, 158.30, 130.26, 128.55, 113.31, 60.16, 55.19, 24.85, 21.70, 14.11, 11.27 ppm.

Data are in accordance with[7] and[8] for *trans* and *cis* isomer, respectively.

***trans*-Ethyl 2-(4-fluorophenyl) cyclopropane-1-carboxylate (2h)**

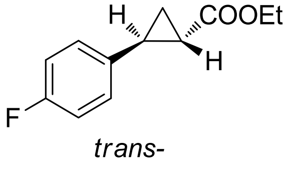

Only *trans* isomer was isolated in the pure form.

Obtained as colourless oil (eluent hexane + 0.2 % AcOEt). Yield *trans* 70%.

^{1}H NMR (300 MHz, $CDCl_3$) δ 7.06 (d, J = 8.7 Hz, 2H), 6.96 (d, J = 8.7 Hz, 2H), 4.17 (q, J = 7.1 Hz, 2H), 2.50 (ddd, J = 9.8, 6.4, 4.4 Hz, 1H), 1.98 – 1.79 (m, 1H), 1.58 (p, J = 5.1 Hz, 2H), 1.28 ppm (t, J = 7.2 Hz, 4H, overlap of $-CH_3$ signal with that of one proton of the cyclopropane scaffold).

^{13}C NMR (75 MHz, $CDCl_3$) δ 173.67, 163.59, 160.35, 136.12, 128.21, 128.10, 115.81, 115.53, 61.17, 25.83, 24.41, 17.30, 14.66 ppm.

^{19}F NMR (282 MHz, $CDCl_3$) δ -116.71 ppm.

Data are in accordance with the one reported in the literature.[7]

Ethyl 2-(4-chlorophenyl) cyclopropane-1-carboxylate (2i)

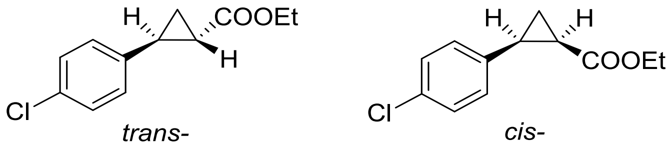

Obtained as colourless oils (*trans* and *cis*) after column chromatography (eluent hexane + 0.2 % AcOEt). Yield *trans*- 73%, *cis*- 23% (*trans*-:*cis*- = 76:24).

For *trans* isomer:

^{1}H NMR (400 MHz, $CDCl_3$) δ 7.24 (d, J = 8.5 Hz, 2H), 7.03 (d, J = 8.4 Hz, 2H), 4.17 (q, J = 7.1 Hz, 2H), 2.49 (ddd, J = 9.5, 6.4, 4.2 Hz, 1H), 1.86 (ddd, J = 8.5, 5.3, 4.3 Hz, 1H), 1.66 – 1.56 (m, 1H), 1.28 ppm (t, J = 7.1 Hz, 4H, overlap of $-CH_3$ signal with that of one proton of the cyclopropane scaffold).

^{13}C NMR (101 MHz, $CDCl_3$) δ 173.11, 138.64, 132.18, 128.55, 127.57, 60.80, 25.48, 24.15, 16.98, 14.11 ppm.

For *cis* isomer:

^{1}H NMR (400 MHz, $CDCl_3$) δ 7.21 (q, J = 8.5 Hz, 4H), 3.90 (q, J = 7.1 Hz, 2H), 2.52 (q, J = 8.6 Hz, 1H), 2.08 (ddd, J = 9.1, 8.0, 5.7 Hz, 1H), 1.67 (dt, J = 7.4, 5.4 Hz, 1H), 1.34 (td, J = 8.3, 5.1 Hz, 1H), 1.02 ppm (t, J = 7.1 Hz, 3H).

^{13}C NMR (101 MHz, $CDCl_3$) δ 170.74, 135.10, 132.41, 130.62, 128.02, 60.32, 24.80, 21.83, 14.09, 11.32 ppm.

Data are in accordance with the one reported in the literature.[8]

Ethyl 2-(4-bromophenyl) cyclopropane-1-carboxylate (2j)

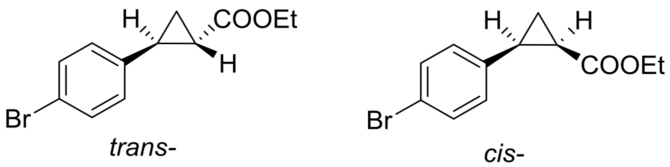

Obtained as white crystalline solid (*trans*) and colourless oil (*cis*) after column chromatography (eluent hexane + 0.2 % AcOEt). Yield *trans*- 72%, *cis*- 11% (*trans*:*cis* = 86:14).

For *trans* isomer:

^{1}H NMR (400 MHz, $CDCl_3$) δ 7.39 (d, J = 8.5 Hz, 2H), 6.97 (d, J = 8.5 Hz, 2H), 4.17 (q, J = 7.1 Hz, 2H), 2.47 (ddd, J = 10.2, 6.4, 4.2 Hz, 1H), 1.86 (ddd, J = 8.6, 5.3, 4.3 Hz, 1H), 1.69 – 1.41 (m, 1H, overlapping

with proton derived from water traces), 1.28 ppm (t, J = 7.1 Hz, 4H, overlap with one proton of the cyclopropane scaffold).

^{13}C NMR (101 MHz, CDCl3) δ 173.11, 139.19, 131.50, 127.94, 120.14, 60.83, 25.56, 24.17, 17.00, 14.25 ppm.

For *cis* isomer:

^{1}H NMR (400 MHz, $CDCl_3$) δ 7.38 (d, J = 8.4 Hz, 2H), 7.14 (d, J = 8.3 Hz, 2H), 3.90 (q, J = 7.1 Hz, 2H), 2.50 (q, J = 8.6 Hz, 1H), 2.08 (ddd, J = 9.1, 8.0, 5.7 Hz, 1H), 1.67 (dt, J = 7.4, 5.4 Hz, 1H), 1.34 (td, J = 8.2, 5.2 Hz, 1H), 1.02 ppm (t, J = 7.1 Hz, 3H).

^{13}C NMR (75 MHz, $CDCl_3$) δ 171.16, 136.02, 131.40, 131.36, 120.92, 60.76, 25.29, 22.21, 14.49, 11.71 ppm.

Data are in accordance with the one reported in the literature.[7]

trans-Ethyl 2-(naphthalen-2-yl)cyclopropane-1-carboxylate (2k)

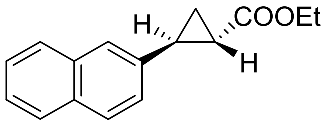

trans-

Only *trans* isomer was isolated in the pure form.

Obtained as colourless oil after column chromatography (eluent: hexane + 0.2 % AcOEt). Yield 64 %.

^{1}H NMR (300 MHz, $CDCl_3$) δ 7.79 (t, J = 9.0 Hz, 3H), 7.58 (s, 1H), 7.52 – 7.39 (m, 1H), 7.22 (dd, J = 8.5, 1.5 Hz, 1H), 4.21 (q, J = 7.1 Hz, 2H), 2.71 (ddd, J = 9.5, 6.5, 4.3 Hz, 1H), 2.17 – 1.88 (m, 1H), 1.69 (dt, J = 9.6, 4.9 Hz, 1H), 1.44 (ddd, J = 8.4, 6.5, 4.6 Hz, 1H), 1.31 ppm (t, J = 7.1 Hz, 3H).

^{13}C NMR (75 MHz, $CDCl_3$) δ 173.77, 137.93, 133.78, 132.70, 128.57, 128.02, 127.80, 126.65, 125.88, 125.19, 124.98, 61.15, 26.81, 24.53, 17.42, 14.69 ppm.

Data are in accordance with the one reported in the literature.[10]

Ethyl 2-(perfluorophenyl)cyclopropane-1-carboxylate (2l)

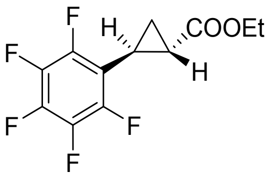

trans-

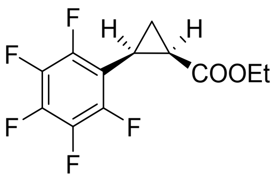

cis-

Obtained as colourless oil (mixture of diasteroisomers) after column chromatography (eluent: hexane + 0.2 % AcOEt). Yield 73% (*trans-*:*cis-* = 71:29).

^{1}H NMR (300 MHz, $CDCl_3$) δ 4.20 (q, J = 7.1 Hz, 2H, *trans*), 4.06 (qd, J = 7.1, 1.9 Hz, 2H, *cis*), 2.45 (ddd, J = 9.6, 6.8, 5.2 Hz, 1H), 2.29 – 2.14 (m, 2H, *trans+cis*), 1.63 (dt, J = 9.9, 5.1 Hz, 2H, *trans+cis*), 1.58 – 1.47 (m, 2H, *trans+cis*), 1.30 (t, J = 7.1 Hz, 3H, *trans*), 1.19 ppm (t, J = 7.1 Hz, 3H, *cis*).

^{13}C NMR (75 MHz, $CDCl_3$) δ 173.11, 61.55, 61.24, 21.37, 19.33, 15.45, 15.08, 14.62, 14.48, 13.97, 13.56 ppm.

^{19}F NMR (282 MHz, $CDCl_3$) δ -141.56 (dd, J = 22.1, 7.7 Hz, *cis*), -143.85 (dd, J = 21.9, 7.7 Hz, *trans*), -156.54 (t, J = 21.0 Hz, *cis*), -156.78 (t, J = 21.0 Hz, *trans*), -162.91 (td, J = 21.8, 7.8 Hz, *trans*), -163.69 ppm (td, J = 22.0, 7.8 Hz, *cis*).

Data are in accordance with the one reported in the literature. [7, 11]

Ethyl (2-(4-chloromethylphenyl)-2-methylcyclopropane-1-carboxylate (2m)

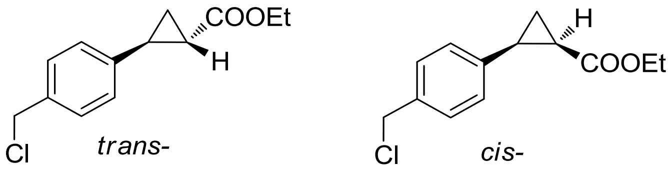

Obtained as colourless oil (both *trans* and *cis*) after column chromatography (eluent hexane + 0.2 % AcOEt). Yield *trans*- 67%, *cis* 15% (*trans*:*cis* = 82:18).

For *trans* isomer:

^1H NMR (300 MHz, $CDCl_3$) δ 7.30 (d, *J* = 8.1 Hz, 2H), 7.09 (d, *J* = 8.1 Hz, 2H), 4.56 (s, 2H), 4.17 (q, *J* = 7.1 Hz, 2H), 2.56 – 2.45 (m, 1H), 1.94 – 1.85 (m, 1H), 1.65 – 1.55 (m, 1H), 1.28 ppm (t, *J* = 7.1 Hz, 3H, overlap of -CH_3 signal with that of one proton of the cyclopropane scaffold).

^{13}C NMR (75 MHz, $CDCl_3$) δ 173.39, 140.73, 135.87, 128.92, 126.69, 60.92, 46.12, 25.99, 24.42, 17.24, 14.39 ppm.

For *cis* isomer:

^1H NMR (300 MHz, $CDCl_3$) δ 7.29 (d, *J* = 8.5 Hz, 2H), 7.25 (d, *J* = 8.5 Hz, 2H), 4.55 (s, 2H), 3.88 (qd, *J* = 7.1, 1.0 Hz, 2H), 2.56 (q, *J* = 8.5 Hz, 1H), 2.08 (ddd, *J* = 9.1, 7.8, 5.7 Hz, 1H), 1.74 – 1.65 (m, 1H), 1.34 (td, *J* = 8.2, 5.3 Hz, 1H), 0.99 ppm (t, *J* = 7.1 Hz, 3H).

^{13}C NMR (75 MHz, $CDCl_3$) δ 155.15, 137.15, 135.92, 129.81, 128.34, 60.42, 46.28, 25.31, 22.00, 14.19, 11.45 ppm.

Data are in accordance with the one reported in the literature.[12]

Ethyl 2-(4-(benzyloxy)-3-methoxyphenyl)cyclopropanecarboxylate (2n)

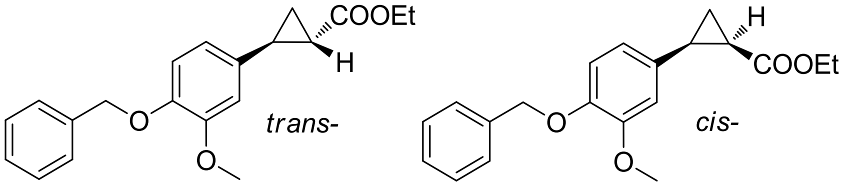

Obtained as colourless oil (both *trans* and *cis*) after column chromatography (gradient elution hexane:AcOEt from 97:3 to 95:5). Yield *trans*- 41%, *cis* 11% (*trans*:*cis* = 79:21).

For *trans* isomer:

^1H NMR (400 MHz, $CDCl_3$) δ 7.34 (d, *J* = 7.2 Hz, 2H), 7.30 – 7.25 (m, 2H), 7.23 – 7.18 (m, 1H), 6.71 (d, *J* = 8.3 Hz, 1H), 6.59 (d, *J* = 2.0 Hz, 1H), 6.49 (dd, *J* = 8.3, 2.0 Hz, 1H), 5.04 (s, 2H), 4.09 (q, *J* = 7.1 Hz, 2H), 3.80 (s, 3H), 2.39 (ddd, *J* = 9.3, 6.5, 4.2 Hz, 1H), 1.76 (ddd, *J* = 8.5, 5.2, 4.2 Hz, 1H), 1.50 – 1.44 (m, 1H), 1.20 ppm (t, *J* = 7.2 Hz, 3H, overlap of -CH_3 signal with that of one proton of the cyclopropane scaffold).

^{13}C NMR (100 MHz, $CDCl_3$) δ 173.59, 149.89, 147.10, 137.37, 133.47, 128.65, 127.93, 127.39, 118.23, 114.57, 110.83, 71.40, 60.80, 56.19, 26.10, 24.05, 16.89, 14.40 ppm.

For *cis* isomer:

^{1}H NMR (300 MHz, $CDCl_3$) δ 7.42 (d, *J* = 7.1 Hz, 2H), 7.35 (t, *J* = 7.2 Hz, 2H), 7.29 (t, *J* = 6.9 Hz, 1H, overlapped with $CDCl_3$ signal), 6.82 (d, *J* = 1.2 Hz, 1H), 6.78 (d, *J* = 8.2 Hz, 1H), 6.74 (dd, *J* = 8.3 Hz, 1.2 Hz, 1H), 5.12 (s, 2H), 3.88 (q, *J* = 7.1 Hz, 2H, overlapped with -OCH_3 signal), 3.87 (s, 3H, -OCH_3), 2.52 (q, *J* = 8.6 Hz, 1H), 2.08 – 1.99 (m, 1H), 1.65 (dt, *J* = 7.1, 5.4 Hz, 1H), 1.29 (td, *J* = 8.1, 5.2 Hz, 1H), 0.98 ppm (t, *J* = 7.1 Hz, 3H).

^{13}C NMR (100 MHz, $CDCl_3$) δ 171.16, 149.32, 147.15, 137.49, 130.02, 128.63, 127.89, 127.44, 121.54, 113.83, 113.36, 71.24, 60.33, 56.13, 25.34, 22.00, 14.27, 11.46 ppm.

Ethyl (2-(4-methylphenyl)-2-methylcyclopropane-1-carboxylate (2o)

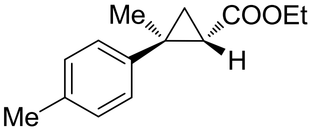

trans-

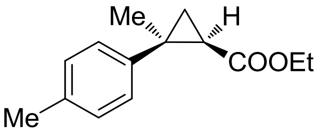

cis-

Obtained as colourless oil (both *trans* and *cis*) after column chromatography (eluent hexane + 0.2 % AcOEt). Yield *trans*- 67%, *cis* 15% (*trans*:*cis* = 82:18).

For *trans* isomer:

^{1}H NMR (400 MHz, $CDCl_3$) δ 7.11 (d, *J* = 8.1 Hz, 2H), 7.02 (d, *J* = 7.9 Hz, 2H), 4.11 (qd, *J* = 7.1, 2.7 Hz, 2H), 2.23 (s, 3H), 1.85 (dd, *J* = 8.3, 6.0 Hz, 1H), 1.43 (s, 3H), 1.36 – 1.27 (m, 2H), 1.21 ppm (t, *J* = 7.1 Hz, 3H).

^{13}C NMR (100 MHz, $CDCl_3$) δ 172.38, 143.15, 136.15, 129.22, 127.30, 60.56, 30.41, 27.98, 21.07, 20.94, 20.07, 14.54. ppm.

For *cis* isomer:

^{1}H NMR (400 MHz, $CDCl_3$) δ 7.16 (d, *J* = 8.1 Hz, 2H), 7.07 (d, *J* = 7.9 Hz, 2H), 3.93 – 3.80 (m, 2H), 2.30 (s, 3H), 1.88 (dd, *J* = 7.8, 5.4 Hz, 1H), 1.75 (t, *J* = 5.0 Hz, 1H), 1.45 (s, 3H), 1.12 (dd, *J* = 7.8, 4.6 Hz, 1H), 0.99 ppm (t, *J* = 7.1 Hz, 3H).

^{13}C NMR (100 MHz, $CDCl_3$) δ 171.49, 138.99, 136.25, 129.03, 128.75, 60.22, 31.90, 28.79, 28.62, 21.24, 19.68, 14.19 ppm.

Ethyl (2-(4-fluorophenyl)-2-methylcyclopropane-1-carboxylate (2p)

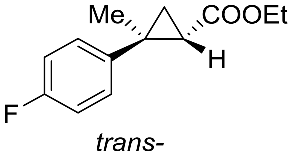

trans-

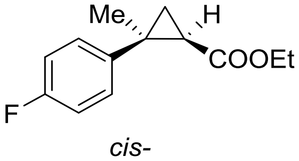

cis-

Obtained as colourless oil (both *trans* and *cis*) after column chromatography (eluent hexane + 0.2 % AcOEt). Yield *trans*- 70%, *cis* 23% (*trans*:*cis* = 76:24).

For *trans* isomer:

^{1}H NMR (300 MHz, $CDCl_3$) δ 7.26 (d, J = 8.8 Hz, 2H), 6.98 (d, J = 8.8 Hz, 2H), 4.19 (qd, J = 7.1, 2.5 Hz, 2H), 1.92 (dd, J = 8.4, 6.0 Hz, 1H), 1.50 (s, 3H), 1.43 (t, J = 5.3 Hz, 1H), 1.37 (dd, J = 8.4, 4.7 Hz, 1H), 1.30 ppm (t, J = 7.1 Hz, 3H).

^{19}F NMR (282 MHz, $CDCl_3$) δ -116.67 ppm.

^{13}C NMR (75 MHz, $CDCl_3$) δ 172.43, 163.42, 160.17, 142.15, 142.10, 129.44, 129.33, 115.73, 115.44, 60.97, 30.47, 28.16, 21.13, 20.55, 14.81 ppm.

For *cis* isomer:

^{1}H NMR (300 MHz, $CDCl_3$) δ 7.22 (dd, J = 5.9, 2.7 Hz, 2H), 6.95 (t, J = 8.7 Hz, 2H), 3.86 (qq, J = 6.8, 3.7 Hz, 2H), 1.89 (dd, J = 7.8, 5.5 Hz, 1H), 1.74 (t, J = 5.0 Hz, 1H), 1.44 (s, 3H), 1.15 (dd, J = 7.8, 4.7 Hz, 1H), 0.99 ppm(t, J = 7.1 Hz, 3H).

^{19}F NMR (282 MHz, $CDCl_3$) δ -116.48 ppm.

^{13}C NMR (75 MHz, $CDCl_3$) δ 130.68, 130.57, 115.53, 115.25, 60.59, 31.69, 30.10, 28.94, 20.05, 14.45 ppm. Quaternary C were not detected.

Data are in accordance with the one reported in the literature.[12]

Ethyl 2-(4-chlorophenyl)-2-methylcyclopropane-1-carboxylate (2q)

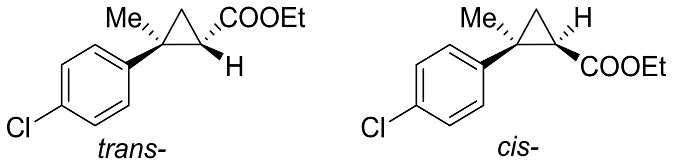

Obtained as light-yellow oil (*trans*) and colourless oil (*cis*) after column chromatography (eluent hexane + 0.2 % AcOEt). Yield *trans* 71%, *cis* 20% (*trans*:*cis* = 78:22).

For *trans* isomer:

^{1}H NMR (400 MHz, $CDCl_3$) δ 7.24 (q, J = 8.6 Hz, 4H), 4.19 (ddq, J = 11.2, 7.1, 3.8 Hz, 2H), 1.92 (dd, J = 8.4, 6.0 Hz, 1H), 1.49 (s, 3H), 1.44 (t, J = 5.4 Hz, 1H), 1.37 (dd, J = 8.4, 4.8 Hz, 1H), 1.29 ppm (t, J = 7.1 Hz, 3H).

^{13}C NMR (101 MHz, $CDCl_3$) δ 171.85, 144.43, 132.20, 128.74, 128.55, 60.59, 29.95, 27.83, 20.71, 19.80, 14.39 ppm.

For *cis* isomer:

^{1}H NMR (400 MHz, $CDCl_3$) δ 7.22 (q, J = 8.5 Hz, 4H), 3.88 (ddq, J = 10.8, 7.1, 3.6 Hz, 2H), 1.91 (dd, J = 7.7, 5.6 Hz, 1H), 1.74 (t, J = 5.0 Hz, 1H), 1.44 (s, 3H), 1.15 (dd, J = 7.7, 4.7 Hz, 1H), 1.00 ppm (t, J = 7.1 Hz, 3H).

^{13}C NMR (75 MHz, $CDCl_3$) δ 170.99, 140.44, 132.35, 130.12, 128.33, 60.22, 31.32, 28.53, 28.32, 19.55, 14.04 ppm.

Data are in accordance with the one reported in the literature.[13]

Ethyl 2,2-diphenylcyclopropane-1-carboxylate (2r)

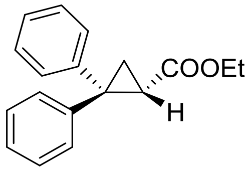

Obtained as colourless oil after column chromatography (eluent hexane + 0.2 % AcOEt). Yield 86%.

^{1}H NMR (300 MHz, $CDCl_3$) δ 7.50 – 7.02 (m, 10H), 4.11 – 3.75 (m, 2H), 2.57 (ddd, J = 8.1, 5.9, 0.7 Hz, 1H), 2.27 – 2.16 (m, 1H), 1.62 (dd, J = 8.2, 4.8 Hz, 1H), 1.04 ppm (t, J = 7.1 Hz, 3H).

^{13}C NMR (75 MHz, $CDCl_3$) δ 171.01, 145.29, 140.67, 130.17, 128.84, 128.67, 127.99, 127.35, 126.90, 60.83, 40.21, 29.46, 20.51, 14.41 ppm.

Data are in accordance with the one reported in the literature.[12]

Ethyl 1,1a,6,6a-tetrahydrocyclopropa[a]indene-1-carboxylate (2s)

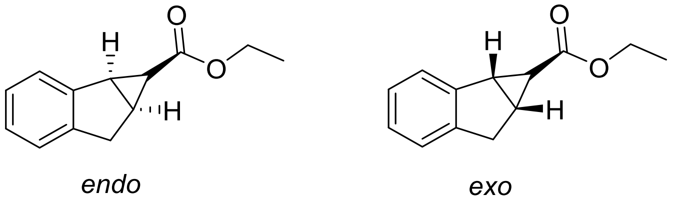

Obtained as pale yellow crystalline solid (*exo*) and colourless oil (*endo*) after column chromatography (eluent hexane + 0.2 % AcOEt). Yield *exo* 54%, *endo* 13% (*endo*:*exo* = 80:20).

For *exo* isomer:

^{1}H NMR (300 MHz, $CDCl_3$) δ 7.37 – 7.31 (m, 1H), 7.19 – 7.08 (m, 3H), 4.15 (q, J = 7.1 Hz, 2H), 3.28 (dd, J = 17.5, 6.4 Hz, 1H), 3.04 (d, J = 17.7 Hz, 1H), 2.95 (d, J = 6.5 Hz, 1H), 2.44 (td, J = 6.4, 3.3 Hz, 1H), 1.27 (t, J = 7.1 Hz, 3H), 1.21 ppm (t, J = 2.8 Hz, 1H).

^{13}C NMR (75 MHz, $CDCl_3$) δ 172.75, 143.64, 141.77, 126.45, 126.33, 125.22, 123.94, 60.56, 35.36, 34.33, 30.75, 26.38, 14.29 ppm.

For *endo* isomer:

^{1}H NMR (400 MHz, $CDCl_3$) δ 7.32 – 7.28 (m, 1H), 7.12 (q, J = 3.6 Hz, 3H), 3.93 – 3.70 (m, 2H), 3.36 (d, J = 17.2 Hz, 1H), 3.23 (d, J = 6.8 Hz, 1H), 3.19 (d, J = 6.8 Hz, 1H), 3.08 – 2.82 (m, 1H), 2.27 (q, J = 6.8 Hz, 1H), 2.01 (t, J = 8.2 Hz, 1H), 0.95 ppm (t, J = 7.1 Hz, 3H).

^{13}C NMR (101 MHz, $CDCl_3$) δ 169.57, 144.67, 139.88, 126.48, 125.98, 124.65, 123.90, 59.88, 32.43, 31.65, 24.65, 23.55, 13.95 ppm.

Data are in accordance with the one reported in the literature.[14, 15]

Ethyl 2-hexylcyclopropane-1-carboxylate (2t)

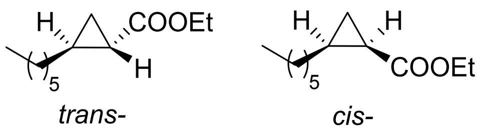

Obtained as colourless oil (mixture of *trans* and *cis* isomers) after column chromatography (eluent: hexane + 0.2 % AcOEt). Yield 69%. (*trans:cis* = 55:45)

^{1}H NMR (300 MHz, $CDCl_3$) δ 4.31 – 3.97 (m, both isomers), 1.70 – 1.64 (m, 1H, *cis*), 1.54 – 1.19 (m, both isomers), 1.17 – 1.10 (m), 1.08 – 0.92 (m, 1H, *cis*), 0.90 – 0.80 (m, both isomers), 0.71 – 0.64 ppm (m, 1H, *trans*).

^{13}C NMR (75 MHz, $CDCl_3$) δ 174.83, 60.43, 60.35, 33.21, 31.99, 31.94, 29.85, 29.77, 29.20, 29.11, 27.17, 23.11, 22.77, 22.13, 20.38, 18.39, 15.68, 14.52, 14.44, 14.23, 13.50 ppm.

Data are in accordance with the one reported in the literature.[16]

Ethyl 2-(cyclohex-3-en-1-yl) cyclopropane-1-carboxylate (2u)

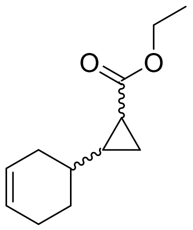

Obtained as colourless oil (mixture of 4 isomers) after column chromatography (eluent: hexane + 0.2 % AcOEt). Yield 57 %.

^{1}H NMR (300 MHz, $CDCl_3$) δ 5.64 (s, 2H), 4.12 (q, J = 7.0 Hz, 2H), 2.23 – 1.97 (m, 3H), 1.92 – 1.65 (m, 2H), 1.51 – 1.29 (m, 3H), 1.25 (t, J = 7.1, 3H), 1.19 – 1.10 (m, 1H), 1.09 – 0.95 (m, 1H), 0.81 – 0.70 ppm (m, 1H).

^{13}C NMR (75 MHz, $CDCl_3$) δ 174.71, 174.67, 127.16, 127.08, 126.36, 126.15, 60.46, 60.42, 37.75, 37.71, 31.92, 31.87, 31.69, 31.29, 31.09, 28.89, 28.80, 28.62, 28.58, 28.41, 28.17, 28.00, 27.56, 25.07, 24.96, 24.82, 19.23, 19.08, 18.19, 18.09, 14.51, 14.42, 14.40, 14.20, 12.70, 12.58 ppm.

Ethyl–6,6–dimethyl–spiro [bicyclo [3.1.1] heptane–2,1'–cyclopropane]–2'–carboxylate (2v)

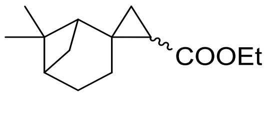

Obtained as colourless oil after column chromatography (eluent: hexane + 0.2 % AcOEt). Yield 48 %.

The product is a mixture of isomers. 1R,2R,2'R and 1R,2R,2'S isomers are detectable by ^{1}H and ^{13}C NMR as the major products in a ca. 58:42 ratio. ^{13}C NMR indicates the presence of two other diastereomer in minor amount. Assignment of 1R,2R,2'R and 1R,2R,2'S signals, isolated in the pure form were previously reported in the literature.[17]

Signals of the mixture are reported below indicating 1R,2R,2'R isomer as **2s-1** and 1R,2R,2'S as **2s-2**. When clearly distinguishable from the signals of the major isomers, minor isomers will be indicated as **2s-min**.

^{1}H NMR (300 MHz, $CDCl_3$) δ 4.20 – 4.02 (m, 2H **2s-1** +2H **2s-2** + **2s-min**), 2.29 – 2.09 (m, 2H **2s-1** + 1H **2s-2**), 2.00 – 1.73 (m, 4H **2s-1** + 4H **2s-2**), 1.66 – 1.58 (m, 1H **2s-2**), 1.53 (d, J = 10.0 Hz, 1H **2s-2**), 1.44 (d, J = 10.0 Hz, 1H **2s-1**), 1.41 – 1.32 (m, 1H **2s-1** +1H **2s-2**), 1.30 – 1.24 (m, 4H **2s-1** +4H **2s-2**), 1.22 (s, 3H **2s-1**), 1.20 – 1.12 (m, 1H **2s-1** +4H **2s-2**), 1.04 (dd, J = 8.0, 4.5 Hz, 1H **2s-1**), 0.97 (s, 3H **2s-2**), 0.96 (s, 3H **2s-1**), 0.91 (dd, J = 8.1, 4.4 Hz, 1H **2s-2** overlapped with **2s-min**), 0.84 – 0.73 ppm (m, 1H **2s-min**).

^{13}C NMR (75 MHz, $CDCl_3$) δ 173.32 (**2s-min**), 172.91 (**2s-2**), 172.84 (**2s-1**), 60.43 (**2s-1**), 60.42 (**2s-2**), 60.35 (**2s-min**), 52.97 (**2s-min**), 52.74 (**2s-2**), 45.07 (**2s-min**), 43.57 (**2s-1**), 41.22 (**2s-2**), 41.07 (**2s-1**), 40.89 (**2s-2**), 40.82 (**2s-min**), 40.69 (**2s-1**), 40.51 (**2s-min**), 40.13 (**2s-min**), 32.09 (**2s-1**), 32.02 (**2s-min**), 31.86 (**2s-min**), 31.39 (**2s-2**), 30.72 (**2s-min**), 28.69 (**2s-1**), 28.60 (**2s-min**), 28.56 (**2s-min**), 27.87 (**2s-min**), 27.11 (**2s-1**), 27.08 (**2s-2**), 26.97 (**2s-1**), 26.85 (**2s-2**), 26.68 (**2s-min**), 26.41 (**2s-2**), 26.00 (**2s-1**), 24.73 (**2s-1**), 24.61 (**2s-1**), 24.47 (**2s-min**), 24.23 (**2s-min**), 24.09 (**2s-2**), 23.73 (**2s-2**), 22.33 (**2s-2**), 22.07 (**2s-2**), 22.04 (**2s-min**), 21.95 (**2s-2**),, 21.82 (**2s-min**), 21.34 (**2s-min**), 20.26 (**2s-min**), 19.65 (**2s-min**), 14.92 (**2s-min**), 14.84 (**2s-2**), 14.77 ppm (**2s-1**).

***t*-butyl–2–methyl–2–phenylcyclopropanecarboxylate (2aa)**

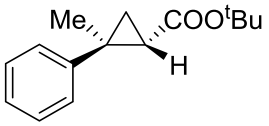

Obtained as *trans* isomer after column chromatography (eluent: hexane + 0.2 % AcOEt). Yield 81 %.

^{1}H NMR (300 MHz, $CDCl_3$) δ 7.32 – 7.27 (m, 4H), 7.23 – 7.16 (m, 1H), 1.90 (dd, J = 8.3, 6.1 Hz, 1H), 1.51 (s, 3H), 1.49 (s, 9H), 1.40 – 1.35 (m, 1H), 1.32 (t, J = 4.2 Hz, 1H).

^{13}C NMR (75 MHz, $CDCl_3$) δ 171.72, 146.61, 128.77, 127.54, 126.67, 80.77, 29.58, 28.69, 20.74, 20.00.

Data are in accordance with the one reported in the literature.[12]

(2-Methyl-2-phenylcyclopropyl) (phenyl) methanone (2ab)

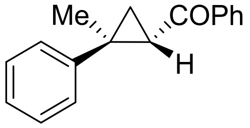

Obtained as colourless oil (only *trans* isomer) after column chromatography (eluent: hexane + 0.2 % AcOEt). Yield 71 %.

^{1}H NMR (300 MHz, $CDCl_3$) δ 8.02 (dd, J = 8.3, 1.3 Hz, 2H), 7.61 – 7.54 (m, 1H), 7.52 – 7.45 (m, 2H), 7.42 – 7.38 (m, 4H), 7.34 – 7.25 (m, 1H), 2.93 (dd, J = 7.9, 6.2 Hz, 1H), 1.91 (dd, J = 6.1, 4.6 Hz, 1H), 1.64 (dd, J = 7.9, 4.5 Hz, 1H), 1.46 ppm (s, 3H).

^{13}C NMR (75 MHz, $CDCl_3$) δ 197.90, 146.19, 139.12, 133.16, 129.11, 129.01, 128.55, 127.07, 126.97, 34.49, 33.99, 21.35, 19.17 ppm.

Data are in accordance with the one reported in the literature.[18]

(2-Methylcyclopropane-1,1,2-triyl) tribenzene (2ac)

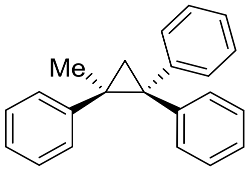

Obtained as white solid after column chromatography (eluent: hexane + 0.2 % AcOEt). Yield 38 %.

^{1}H NMR (300 MHz, $CDCl_3$) δ 7.64 – 7.57 (m, 2H), 7.40 (t, J = 7.5 Hz, 2H), 7.32 – 7.19 (m, 5H), 7.12 (ddd, J = 8.0, 6.1, 1.6 Hz, 3H), 7.07 – 6.92 (m, 3H), 2.29 (d, J = 5.2 Hz, 1H), 1.63 (d, J = 5.3 Hz, 1H), 1.44 ppm (s, 3H).

^{13}C NMR (75 MHz, $CDCl_3$) δ 144.12, 143.60, 143.56, 130.74, 129.95, 128.91, 128.76, 128.17, 127.90, 126.64, 126.01, 125.74, 43.35, 33.24, 26.62, 25.44 ppm.

Data are in accordance with the one reported in the literature.[19]

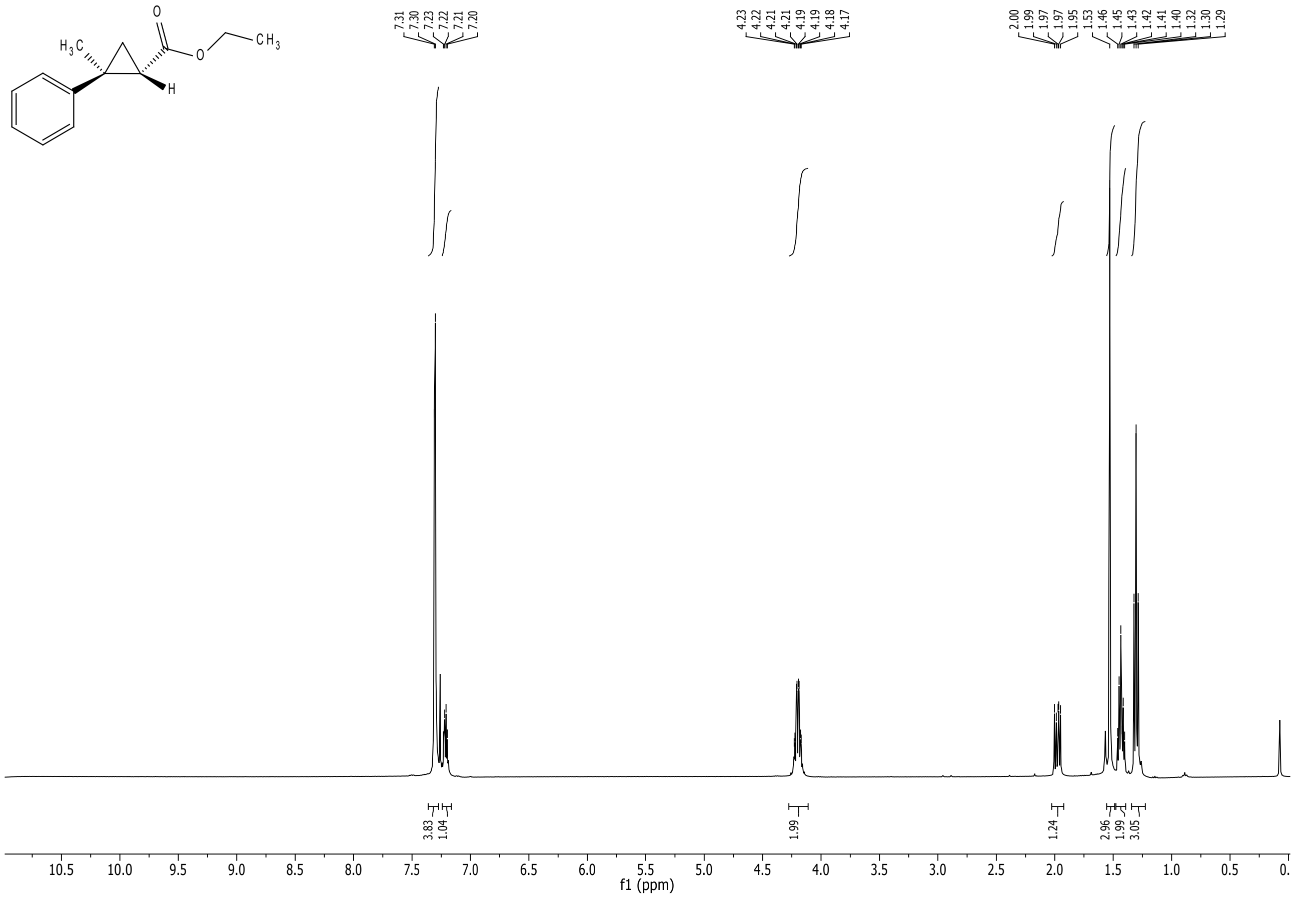
O
H3C
CH3
O
H
7.31
7.30
7.23
7.22
7.21
7.20
4.23
4.22
4.21
4.21
4.19
4.19
4.18
4.17
2.00
1.99
1.97
1.97
1.95
1.53
1.46
1.45
1.43
1.42
1.41
1.40
1.32
1.30
1.29
3.83
1.04
1.99
1.24
2.96
1.99
3.05
f1 (ppm)

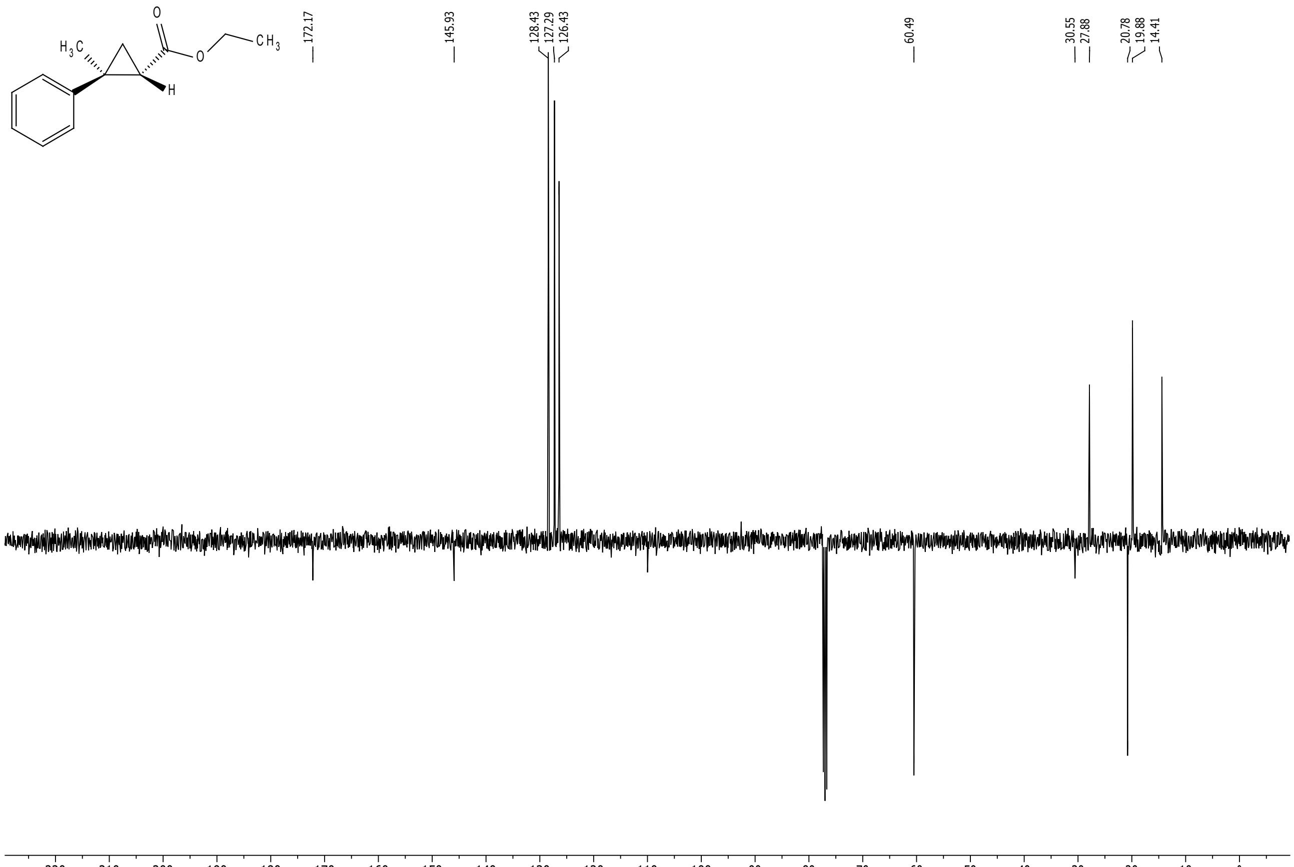
O
H3C
CH3
O
H
172.17
145.93
128.43
127.29
126.43
60.49
30.55
27.88
20.78
19.88
14.41
f1 (ppm)

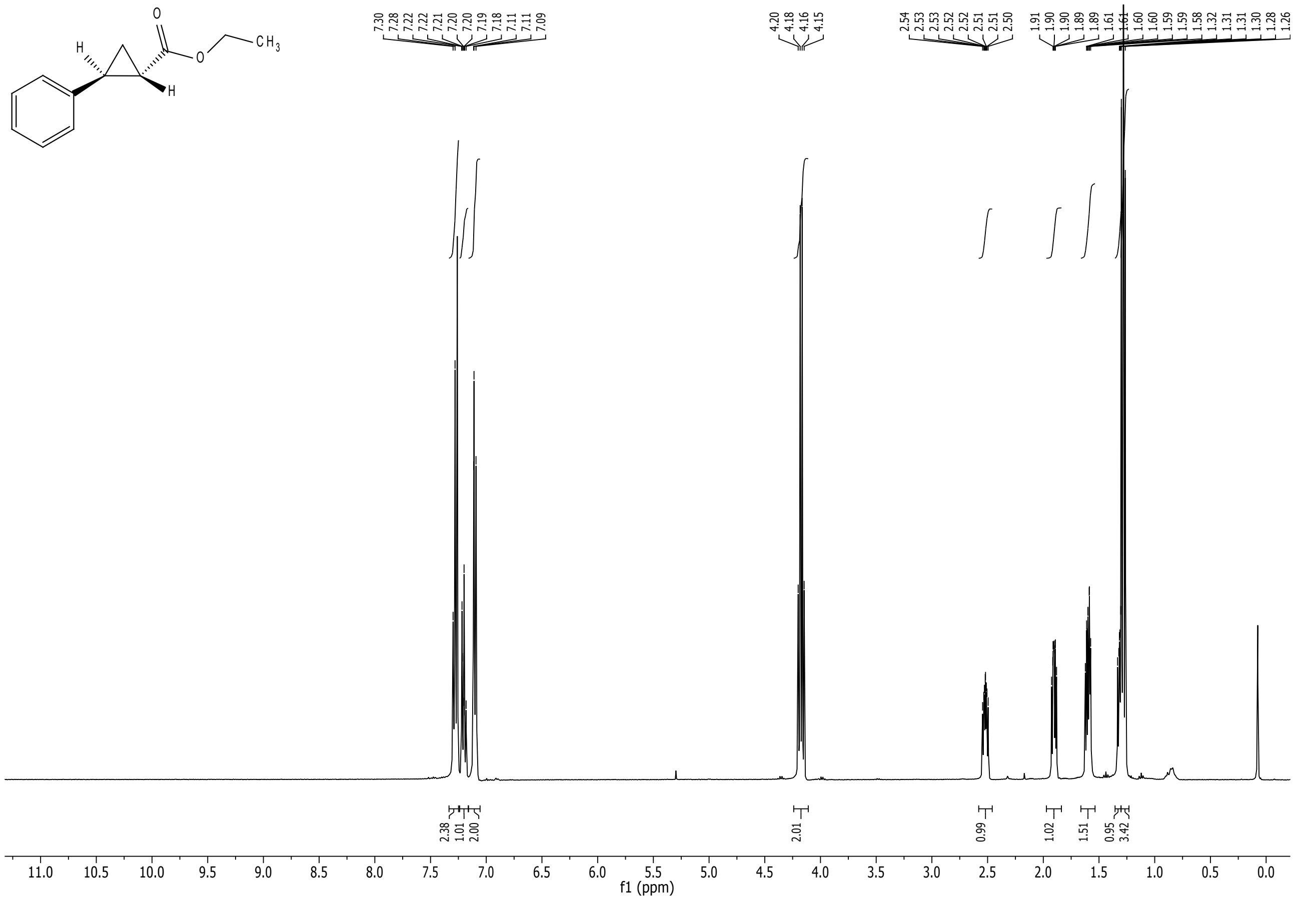
O
O
CH3
H
H
7.30 7.28 7.22 7.22 7.21 7.20 7.20 7.19 7.18 7.11 7.11 7.09
4.20 4.18 4.16 4.15
2.54 2.53 2.53 2.52 2.52 2.51 2.51 2.50
1.91 1.90 1.90 1.89 1.89 1.61 1.61 1.60 1.60 1.59 1.59 1.58 1.32 1.31 1.31 1.30 1.28 1.26
2.38 1.01 2.00
2.01
0.99
1.02
1.51
0.95 3.42
f1 (ppm)

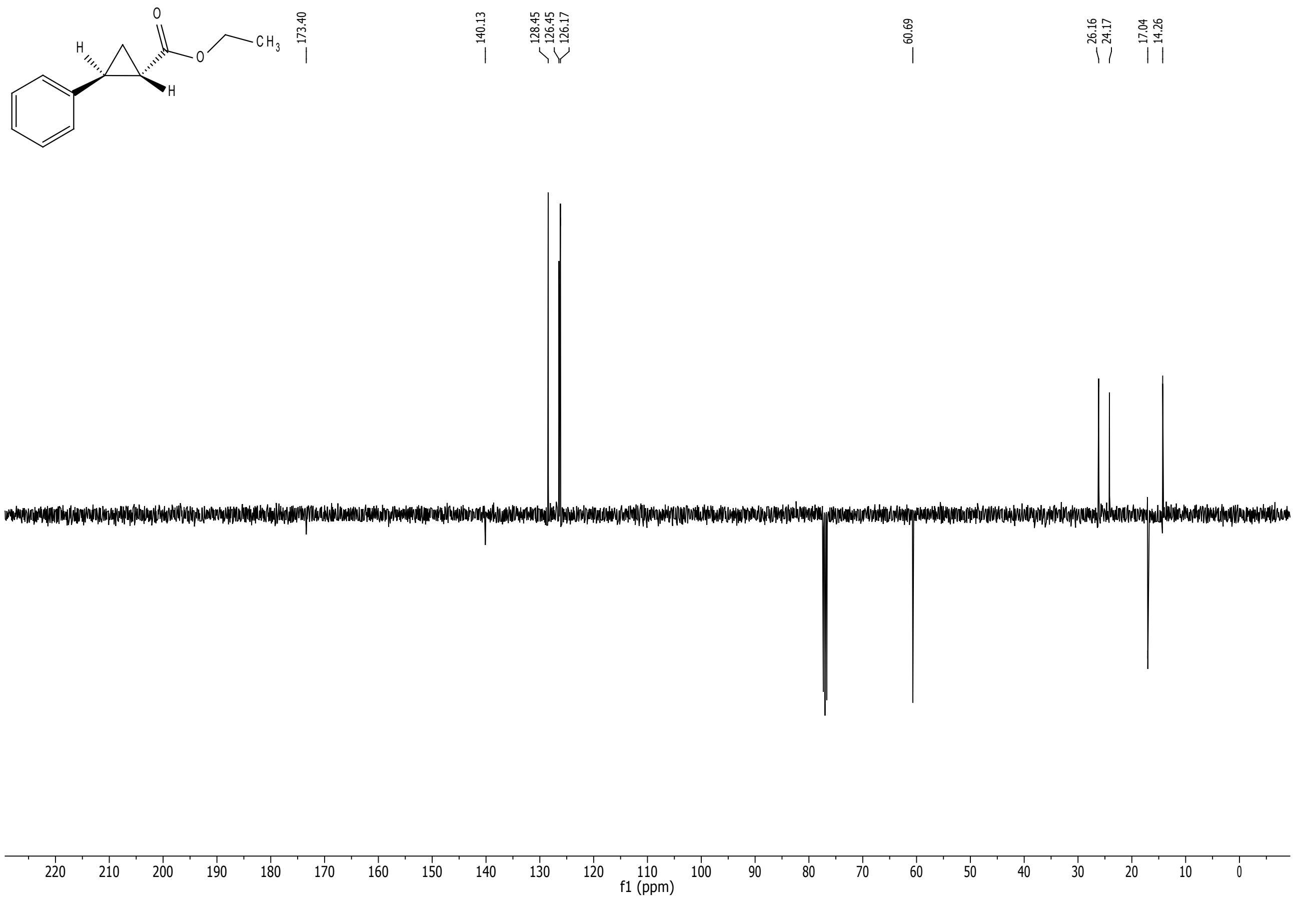
O
O
CH3
H
H
173.40
140.13
128.45 126.45 126.17
60.69
26.16 24.17
17.04 14.26
f1 (ppm)

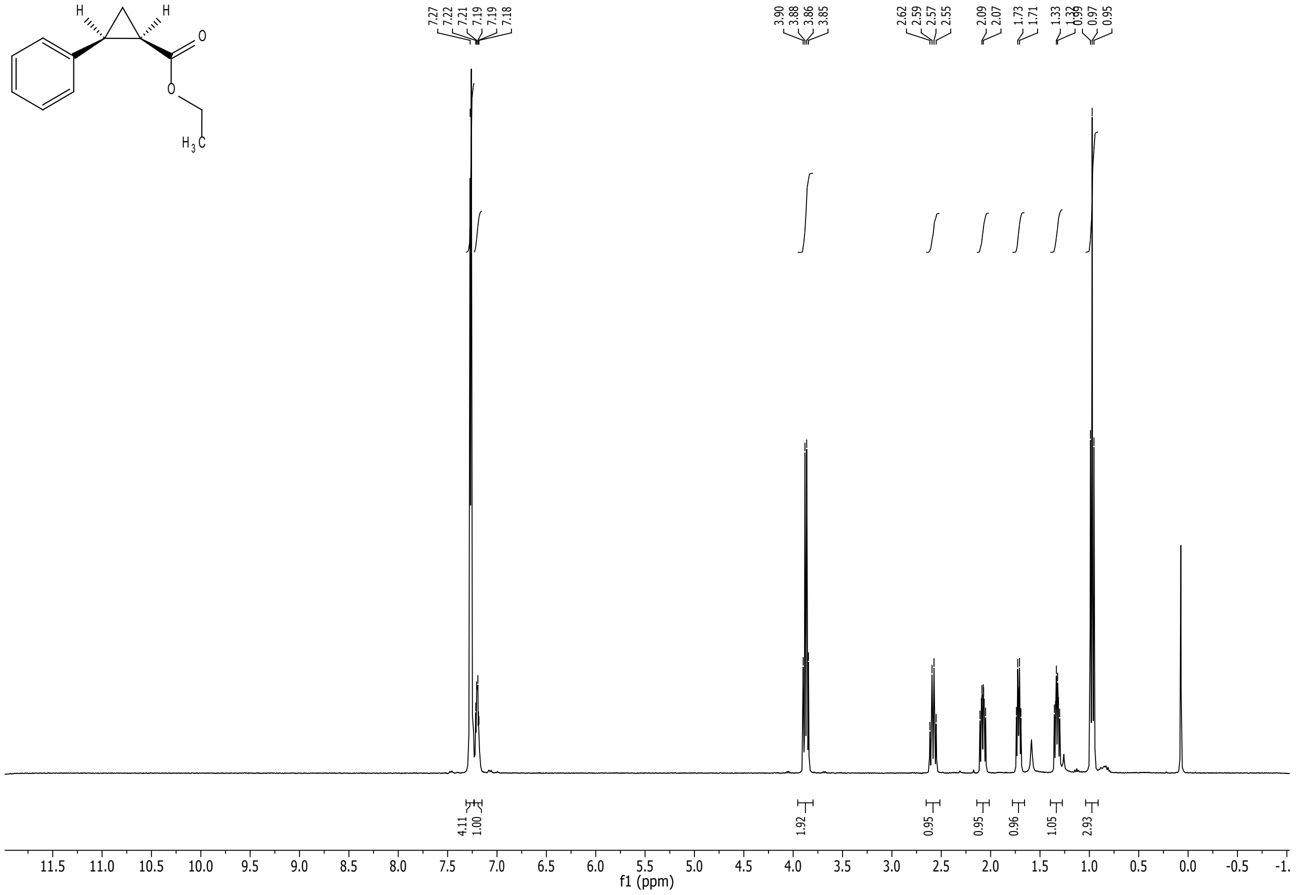
H
H
O
O
H_3C
7.27
7.22
7.21
7.19
7.19
7.18
3.90
3.88
3.86
3.85
2.62
2.59
2.57
2.55
2.09
2.07
1.73
1.71
1.33
0.97
0.95
4.11
1.00
1.92
0.95
0.95
0.96
1.05
2.93
f1 (ppm)

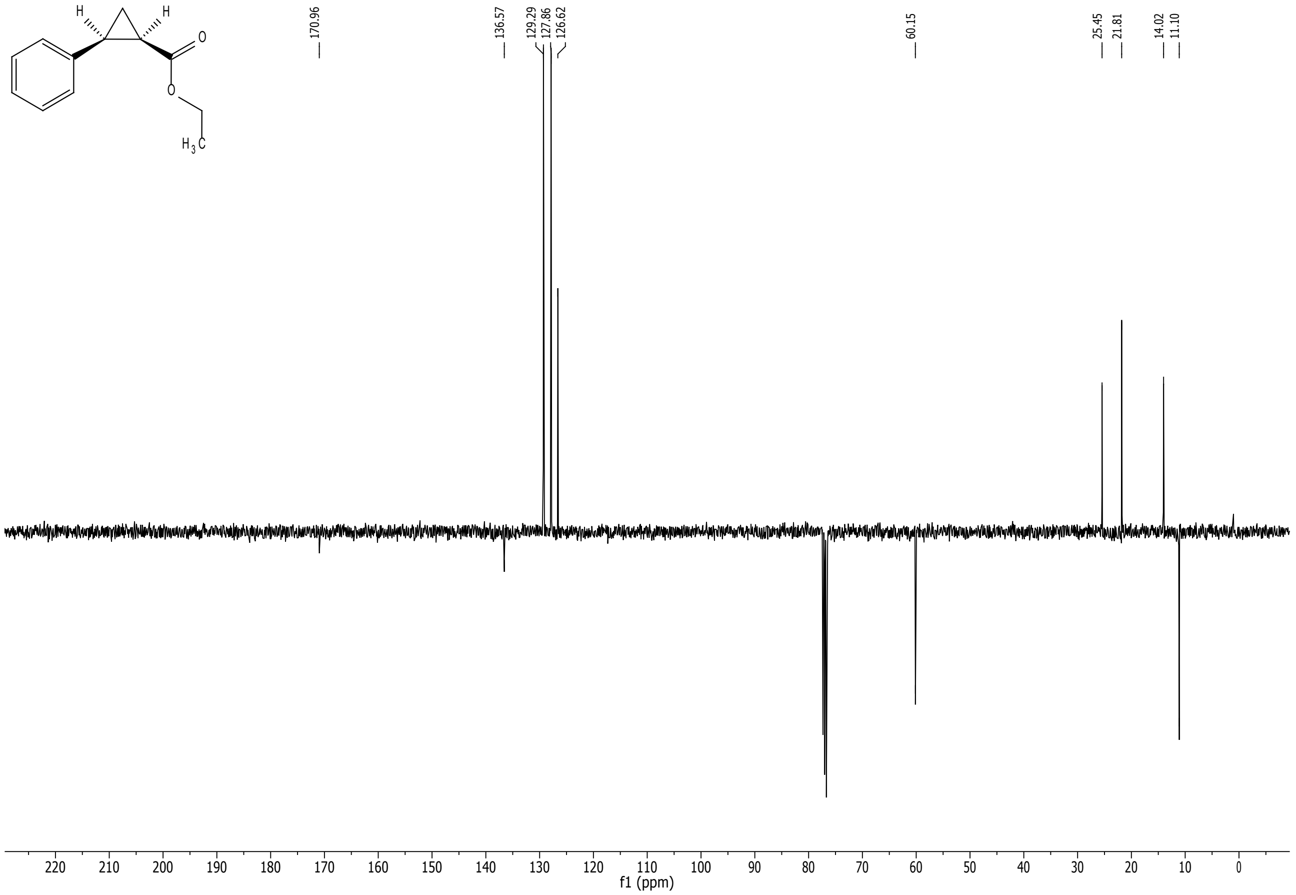
H
H
O
O
H_3C
170.96
136.57
129.29
127.86
126.62
60.15
25.45
21.81
14.02
11.10
f1 (ppm)

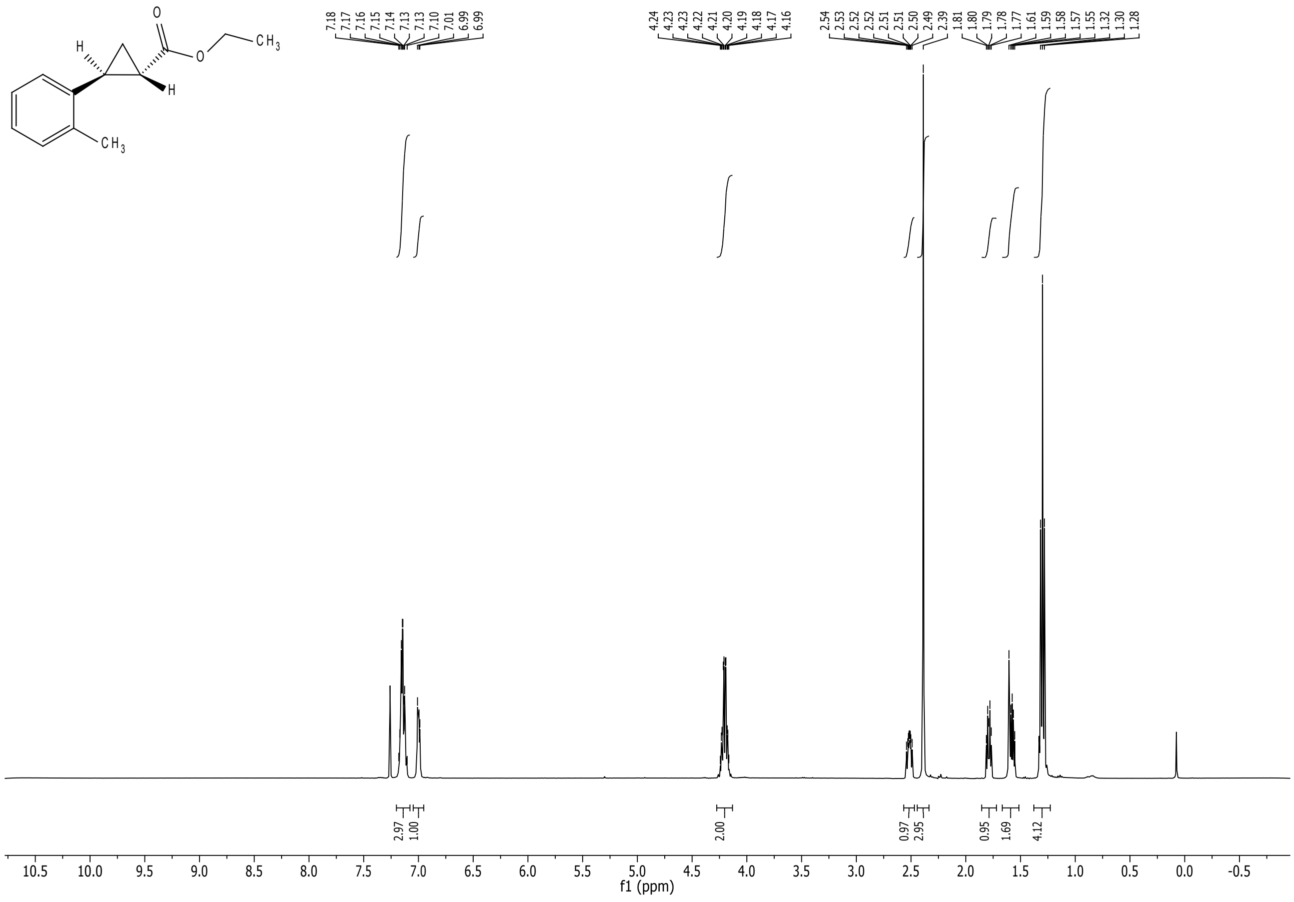
O
O
CH3
H
H
CH3
7.18 7.17 7.16 7.15 7.14 7.13 7.13 7.10 7.01 6.99 6.99
4.24 4.23 4.23 4.22 4.21 4.20 4.19 4.18 4.17 4.16
2.54 2.53 2.52 2.52 2.51 2.51 2.50 2.49 2.39 1.81 1.80 1.79 1.78 1.77 1.61 1.59 1.58 1.57 1.55 1.32 1.30 1.28
2.97 1.00 2.00 0.97 2.95 0.95 1.69 4.12
f1 (ppm)

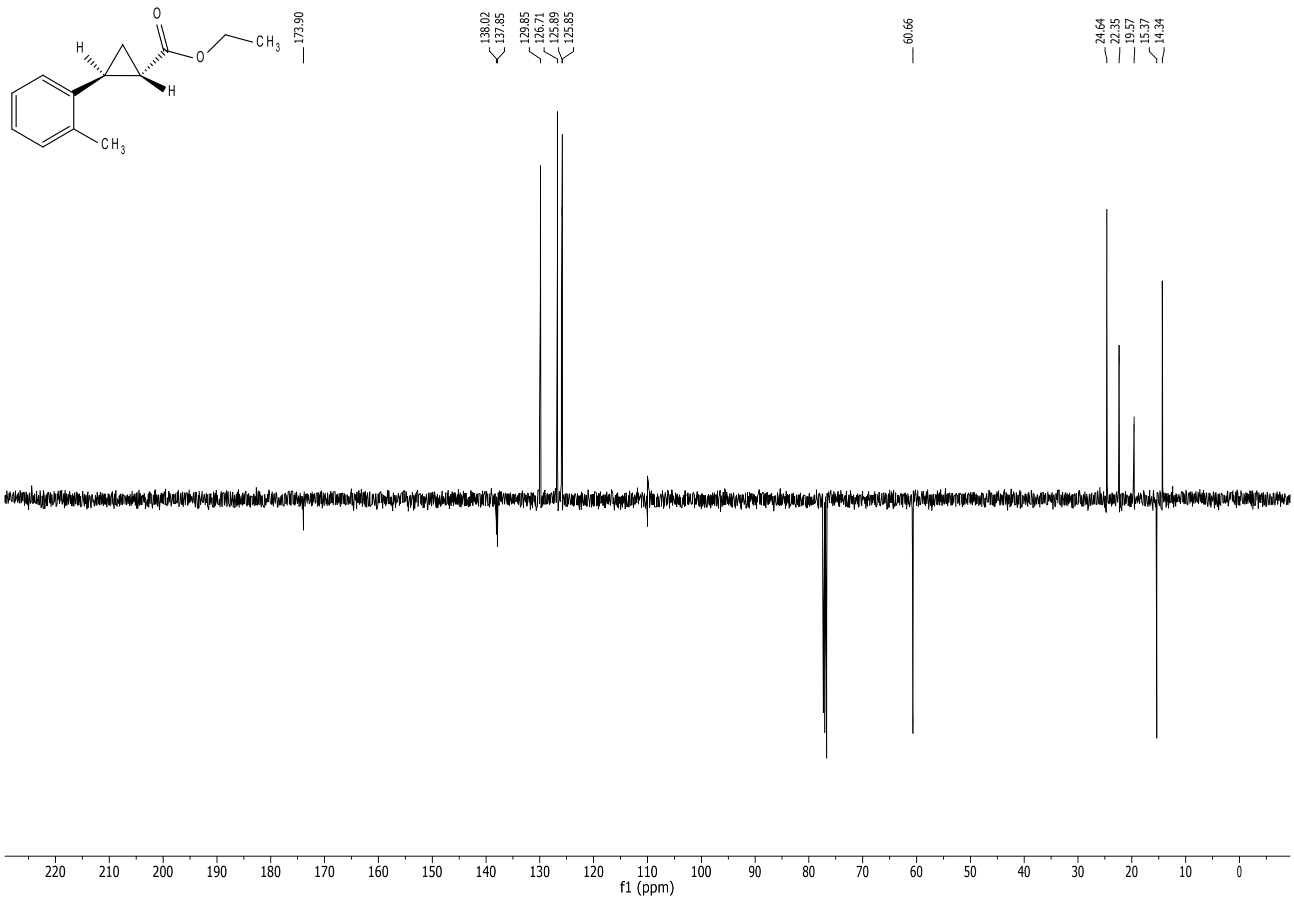
O
O
CH3
H
H
CH3
173.90
138.02 137.85 129.85 126.71 125.89 125.85
60.66
24.64 22.35 19.57 15.37 14.34
f1 (ppm)

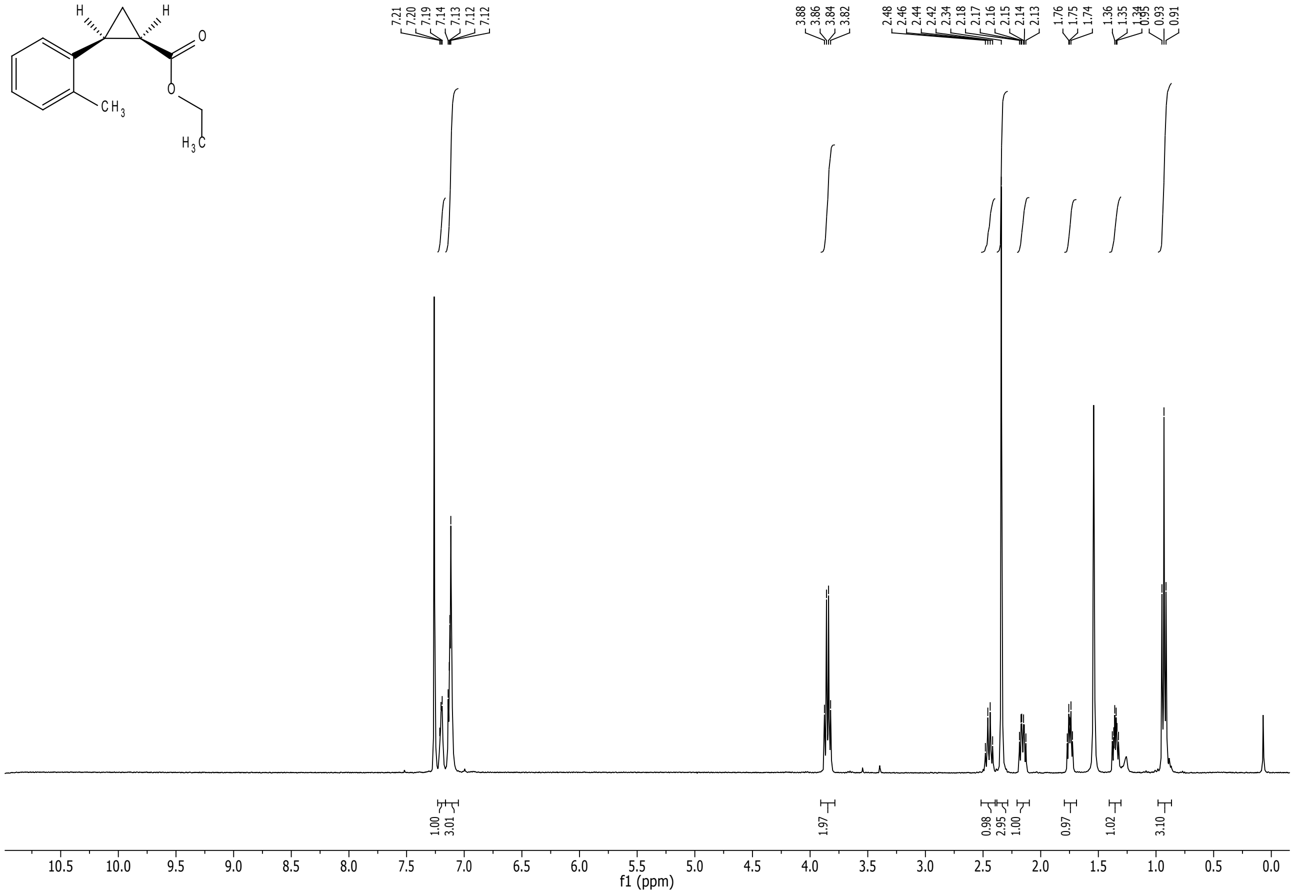

7.21 7.20 7.19 7.14 7.13 7.12 7.12
3.88 3.86 3.84 3.82
2.48 2.46 2.44 2.42 2.34 2.18 2.17 2.16 2.15 2.14 2.13
1.76 1.75 1.74
1.36 1.35 1.34 0.95 0.93 0.91
1.00 3.01 1.97 0.98 2.95 1.00 0.97 1.02 3.10
10.5 10.0 9.5 9.0 8.5 8.0 7.5 7.0 6.5 6.0 5.5 5.0 4.5 4.0 3.5 3.0 2.5 2.0 1.5 1.0 0.5 0.0
f1 (ppm)

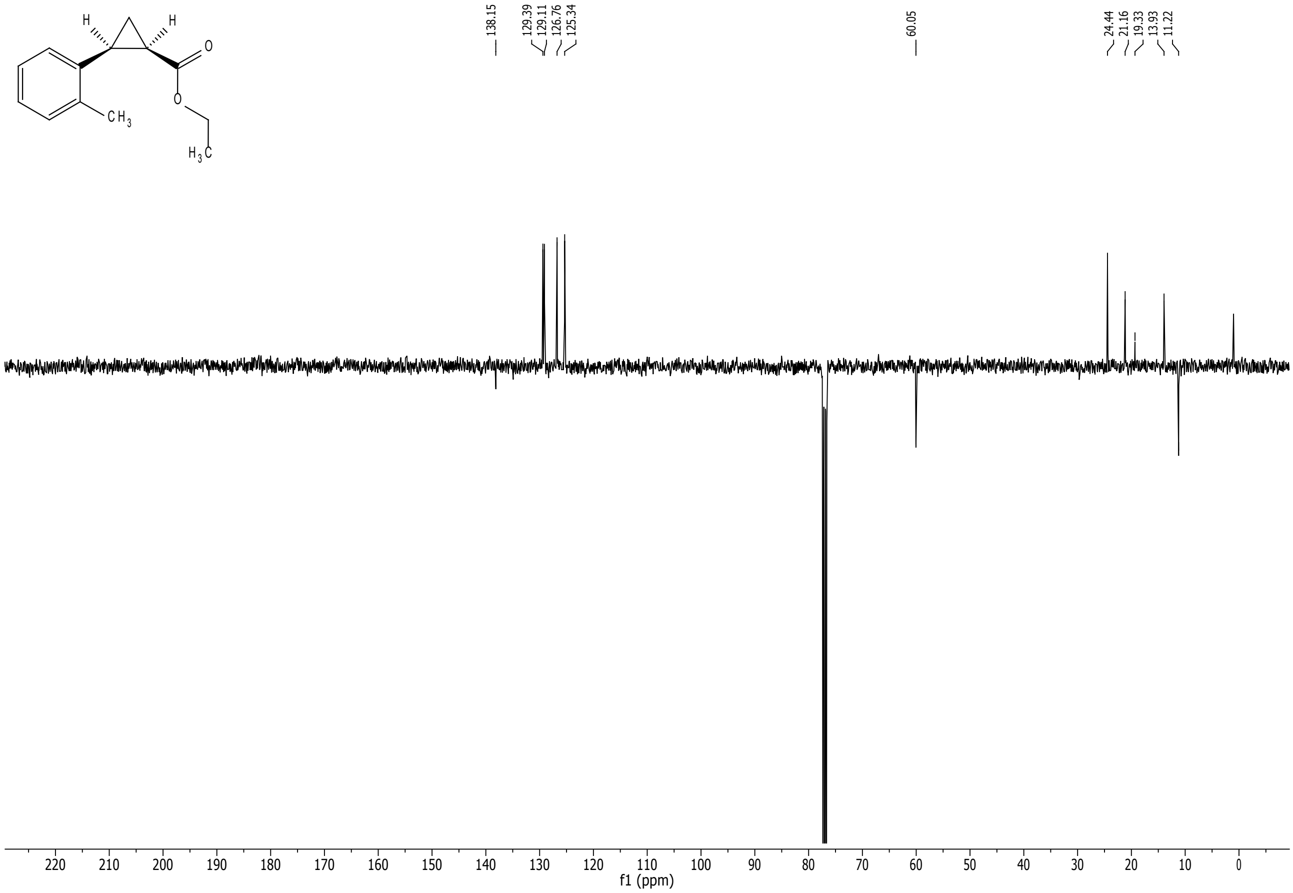

138.15
129.39 129.11 126.76 125.34
60.05
24.44 21.16 19.33 13.93 11.22
220 210 200 190 180 170 160 150 140 130 120 110 100 90 80 70 60 50 40 30 20 10 0
f1 (ppm)

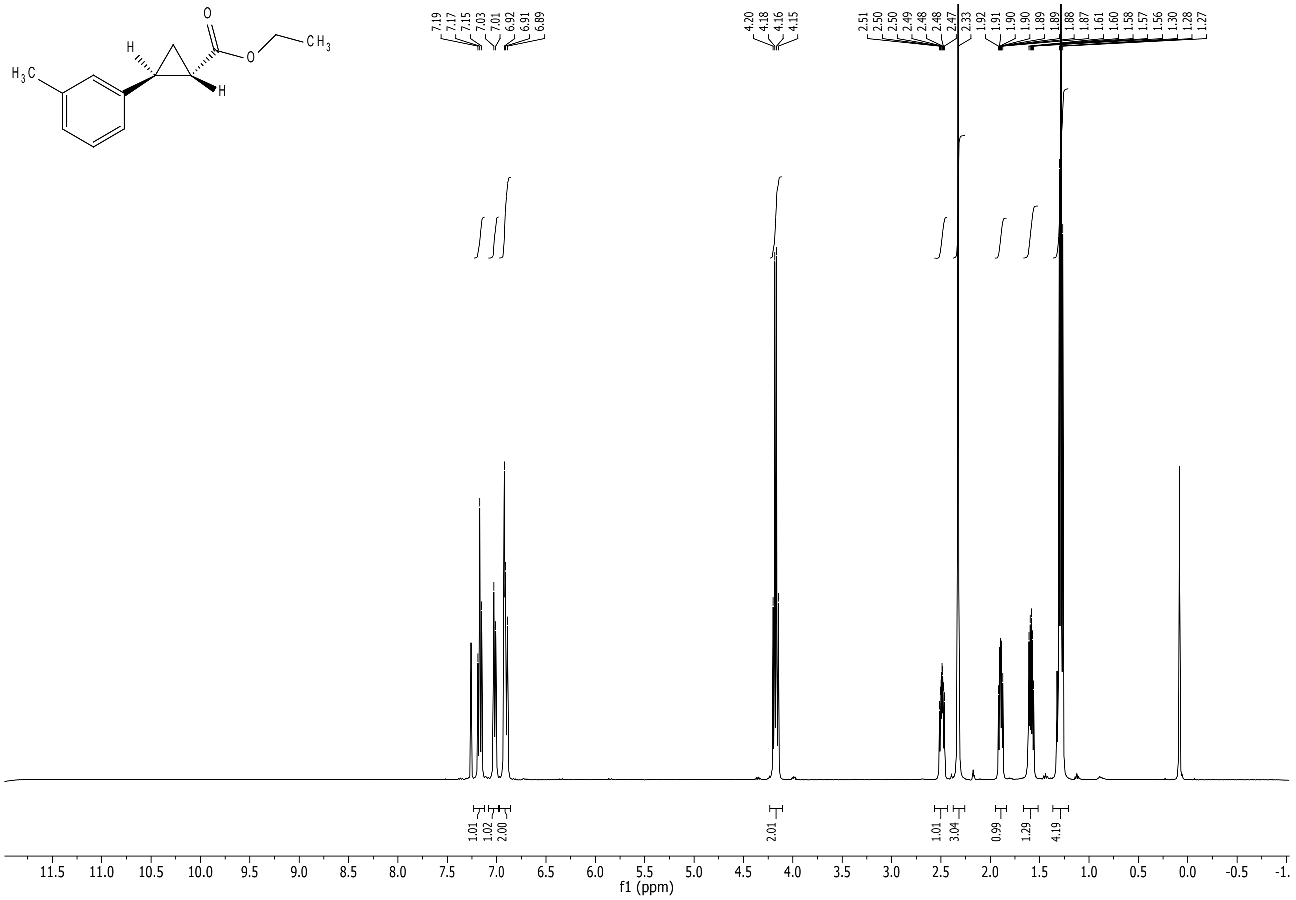
7.19
7.17
7.15
7.03
7.01
6.92
6.91
6.89
4.20
4.18
4.16
4.15
2.51
2.50
2.50
2.49
2.48
2.48
2.47
2.33
1.92
1.91
1.90
1.90
1.89
1.89
1.88
1.87
1.61
1.60
1.58
1.58
1.57
1.56
1.30
1.28
1.27
1.01
1.02
2.00
2.01
1.01
3.04
0.99
1.29
4.19
f1 (ppm)

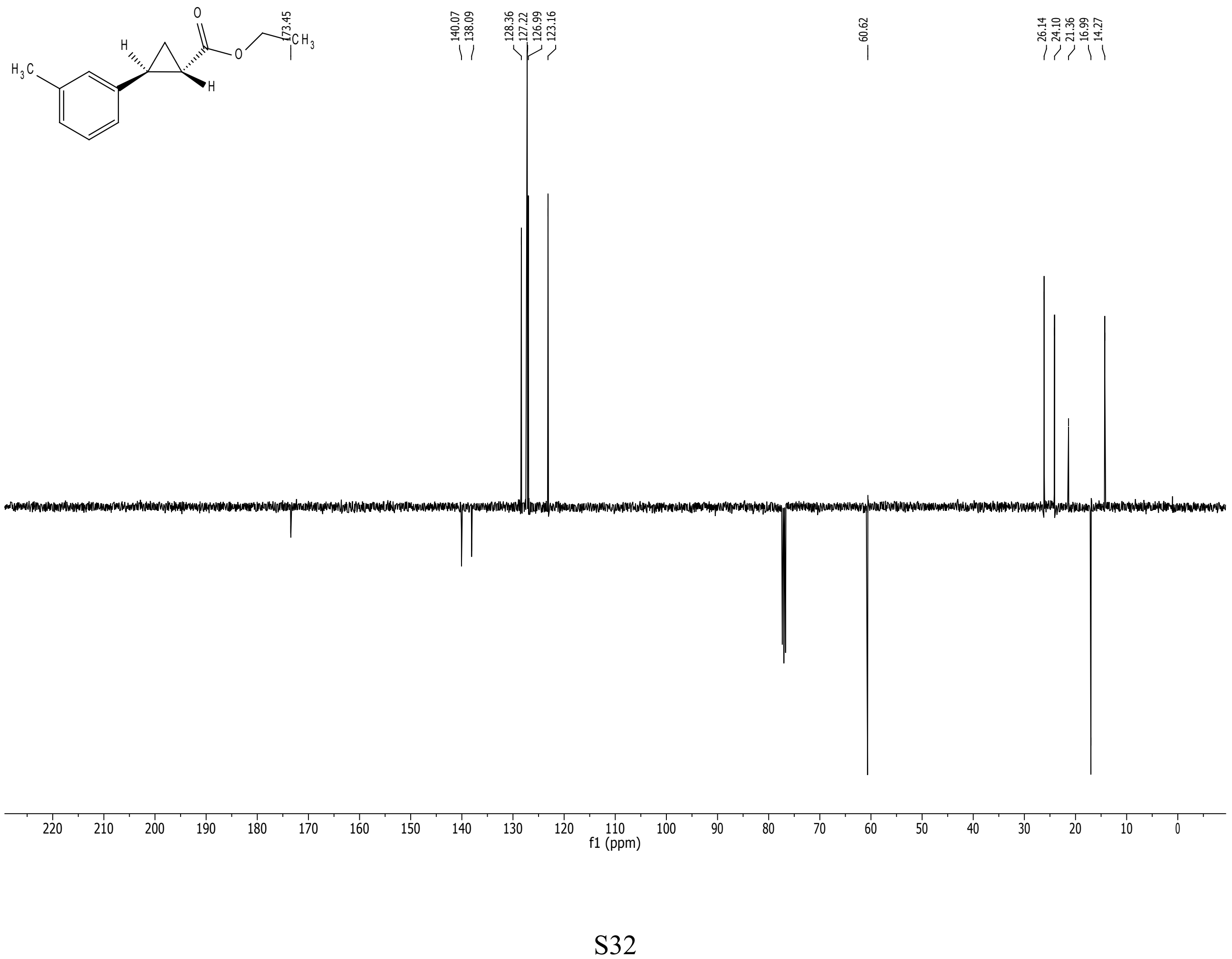
173.45
140.07
138.09
128.36
127.22
126.99
123.16
60.62
26.14
24.10
21.36
16.99
14.27
f1 (ppm)

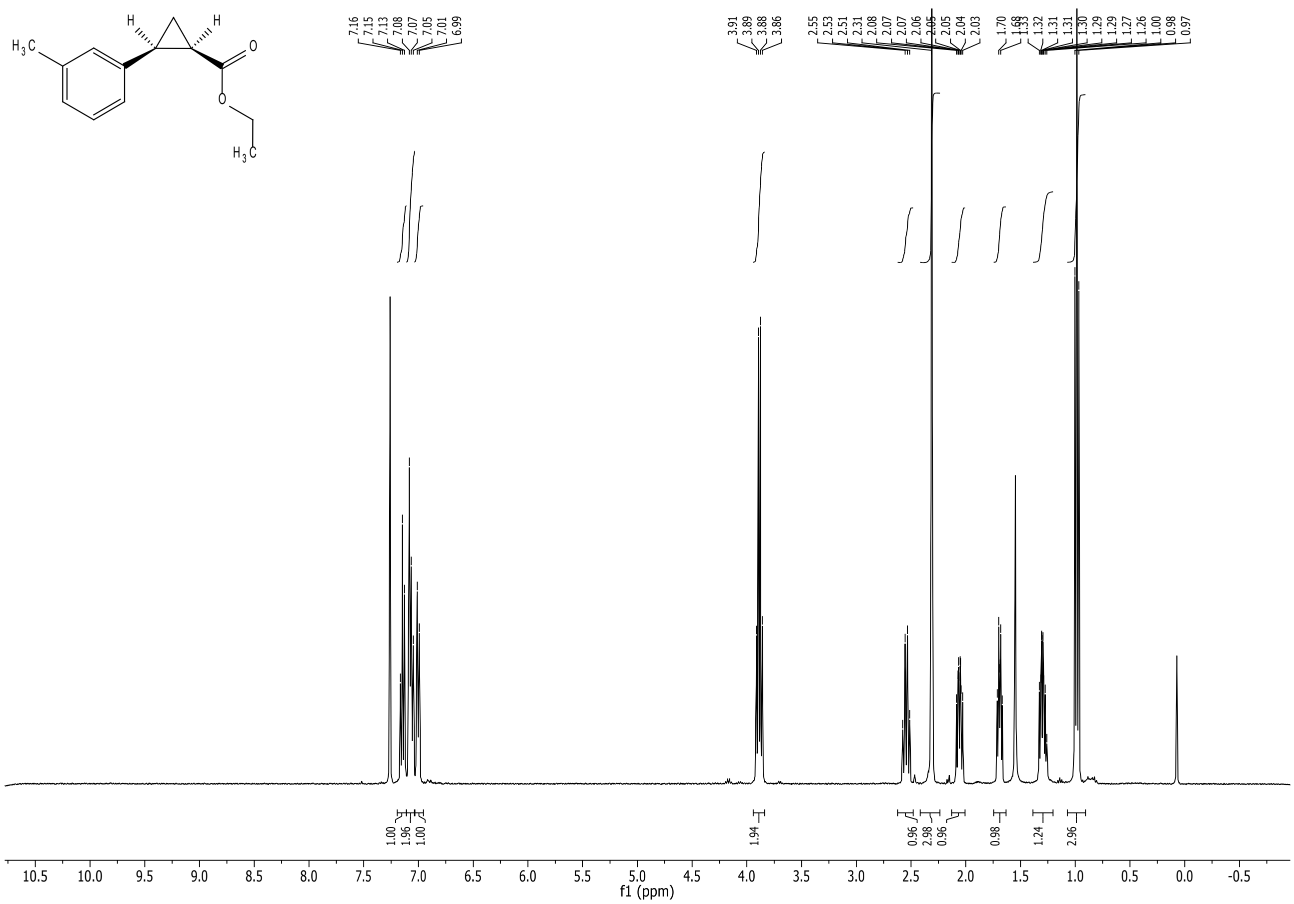
H
H
H_3C
O
O
H_3C
7.16
7.15
7.13
7.08
7.07
7.05
7.01
6.99
3.91
3.89
3.88
3.86
2.55
2.53
2.51
2.31
2.08
2.07
2.07
2.06
2.05
2.05
2.04
2.03
1.70
1.68
1.33
1.32
1.31
1.31
1.30
1.29
1.29
1.27
1.26
1.00
0.98
0.97
1.00
1.96
1.00
1.94
0.96
2.98
0.96
0.98
1.24
2.96
10.5 10.0 9.5 9.0 8.5 8.0 7.5 7.0 6.5 6.0 5.5 5.0 4.5 4.0 3.5 3.0 2.5 2.0 1.5 1.0 0.5 0.0 -0.5
f1 (ppm)

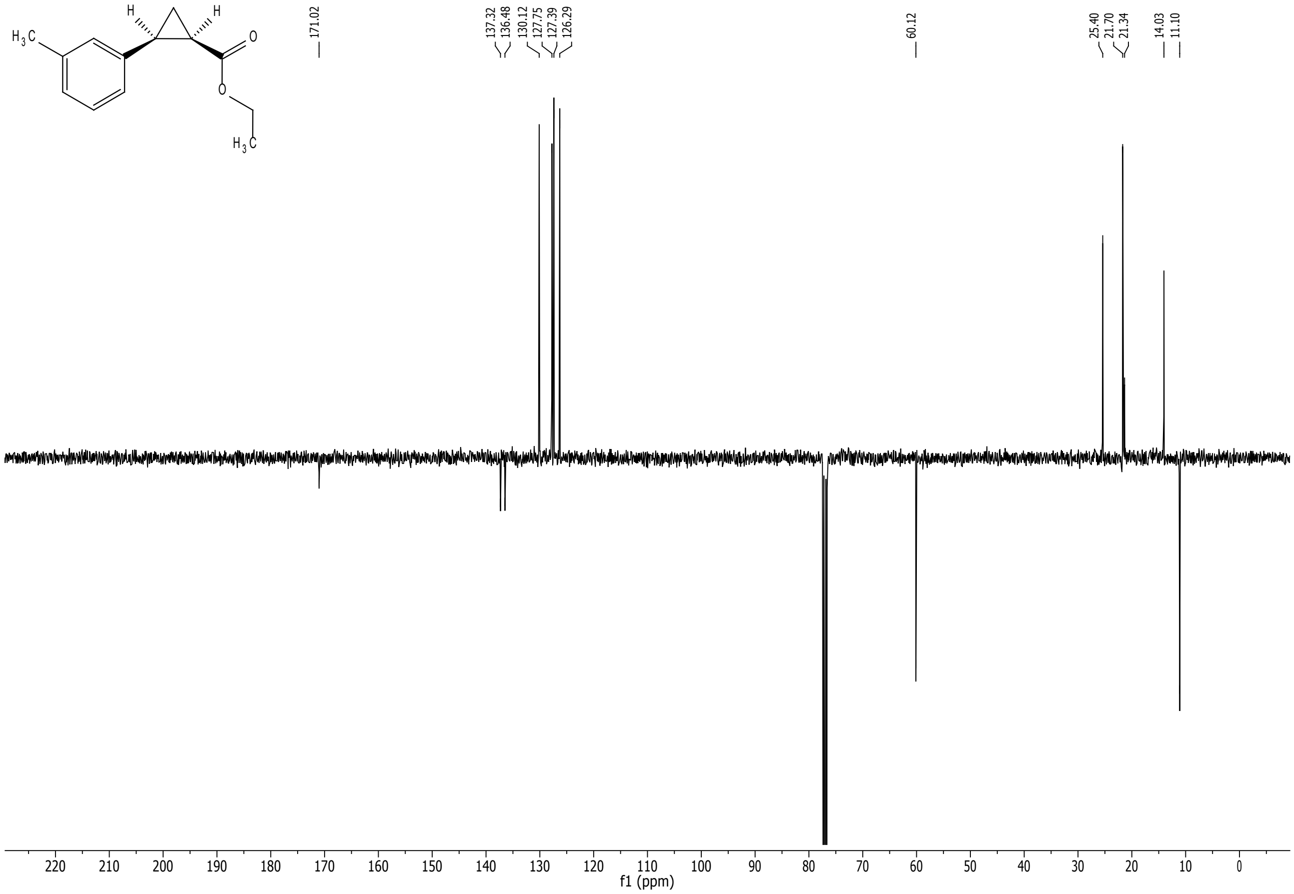
H
H
H_3C
O
O
H_3C
171.02
137.32
136.48
130.12
127.75
127.39
126.29
60.12
25.40
21.70
21.34
14.03
11.10
220 210 200 190 180 170 160 150 140 130 120 110 100 90 80 70 60 50 40 30 20 10 0
f1 (ppm)

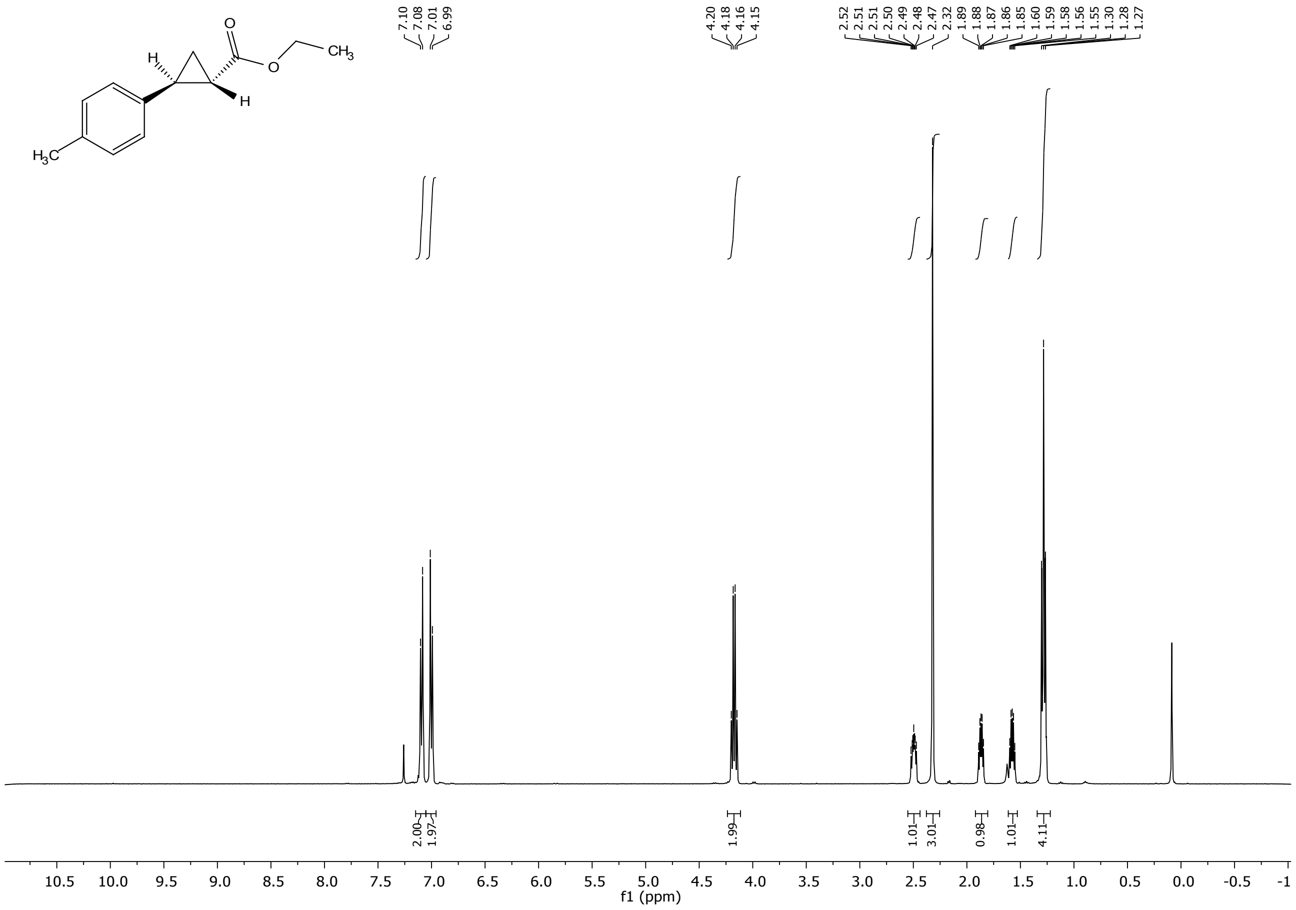
7.10
7.08
7.01
6.99
4.20
4.18
4.16
4.15
2.52
2.51
2.51
2.50
2.49
2.48
2.47
2.32
1.89
1.88
1.87
1.86
1.85
1.60
1.59
1.58
1.56
1.55
1.30
1.28
1.27
2.00
1.97
1.99
1.01
3.01
0.98
1.01
4.11
10.5 10.0 9.5 9.0 8.5 8.0 7.5 7.0 6.5 6.0 5.5 5.0 4.5 4.0 3.5 3.0 2.5 2.0 1.5 1.0 0.5 0.0 -0.5 -1
f1 (ppm)

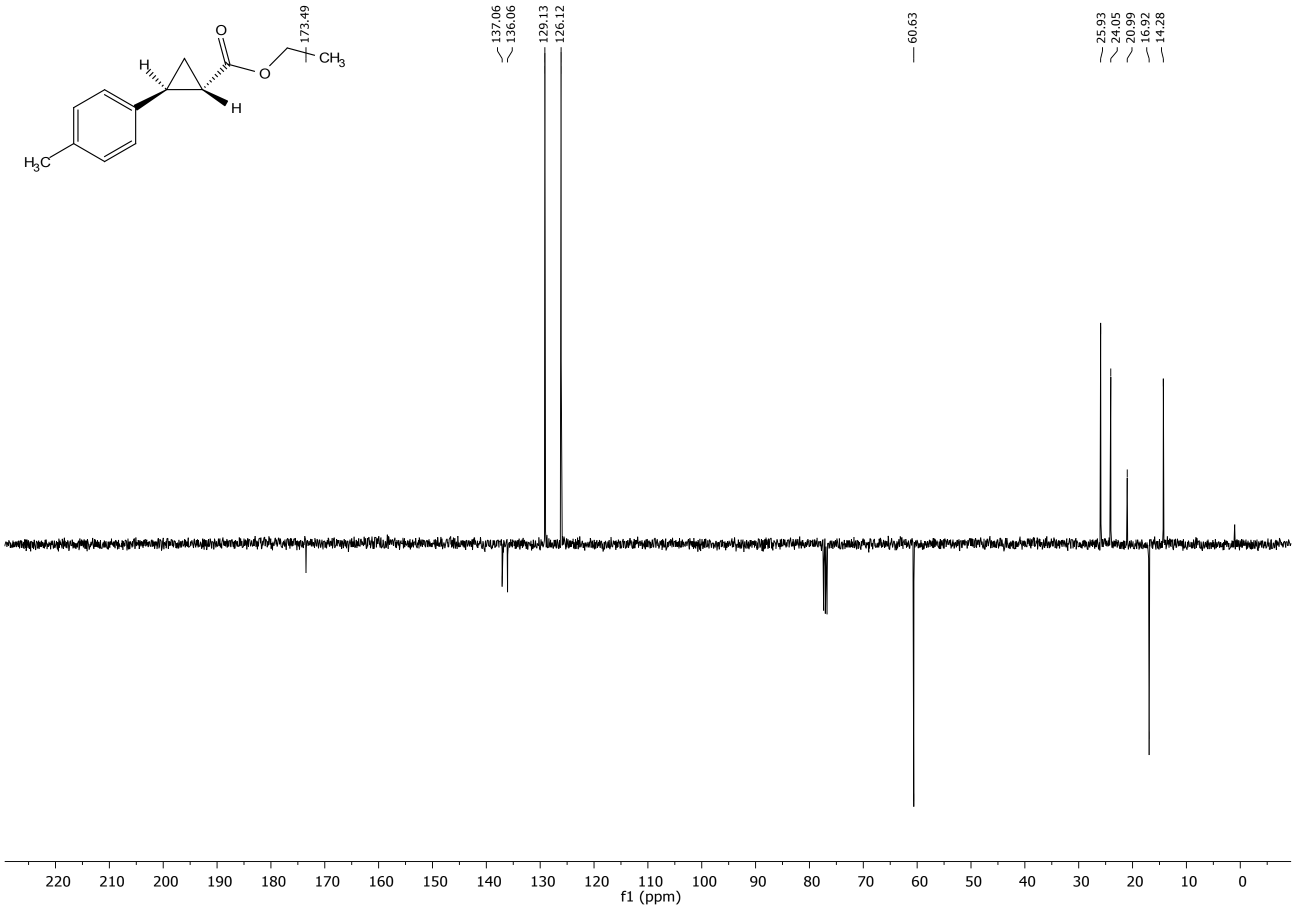
173.49
137.06
136.06
129.13
126.12
60.63
25.93
24.05
20.99
16.92
14.28
O
O
H
H
CH3
H3C
f1 (ppm)

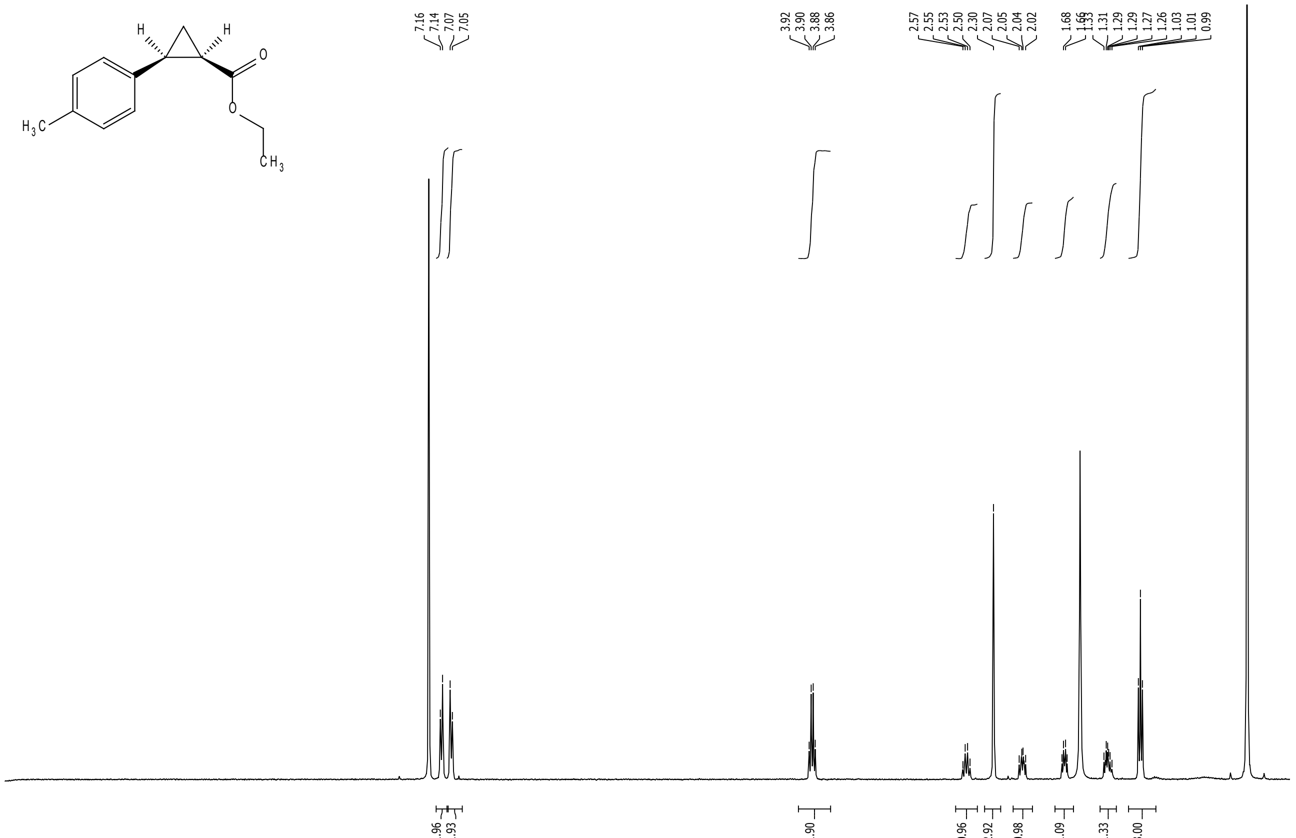
7.16
7.14
7.07
7.05
3.92
3.90
3.88
3.86
2.57
2.55
2.53
2.50
2.30
2.07
2.05
2.04
2.02
1.68
1.66
1.33
1.31
1.29
1.29
1.27
1.26
1.03
1.01
0.99
1.96
1.93
1.90
0.96
2.92
0.98
1.09
1.33
3.00
H
H
O
O
CH3
H3C
f1 (ppm)

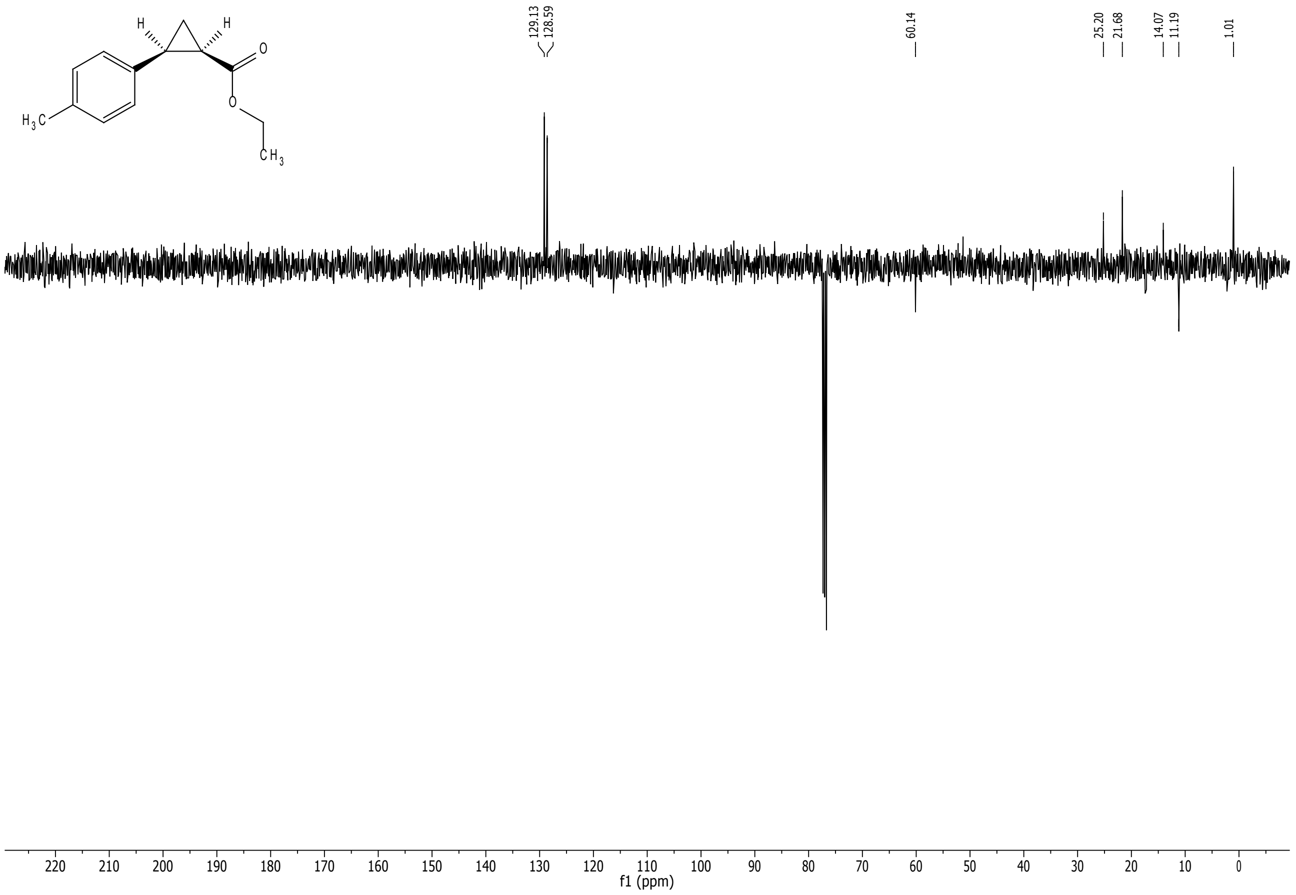
129.13
128.59
60.14
25.20
21.68
14.07
11.19
1.01
H_3C
H
H
O
O
CH_3
220 210 200 190 180 170 160 150 140 130 120 110 100 90 80 70 60 50 40 30 20 10 0
f1 (ppm)

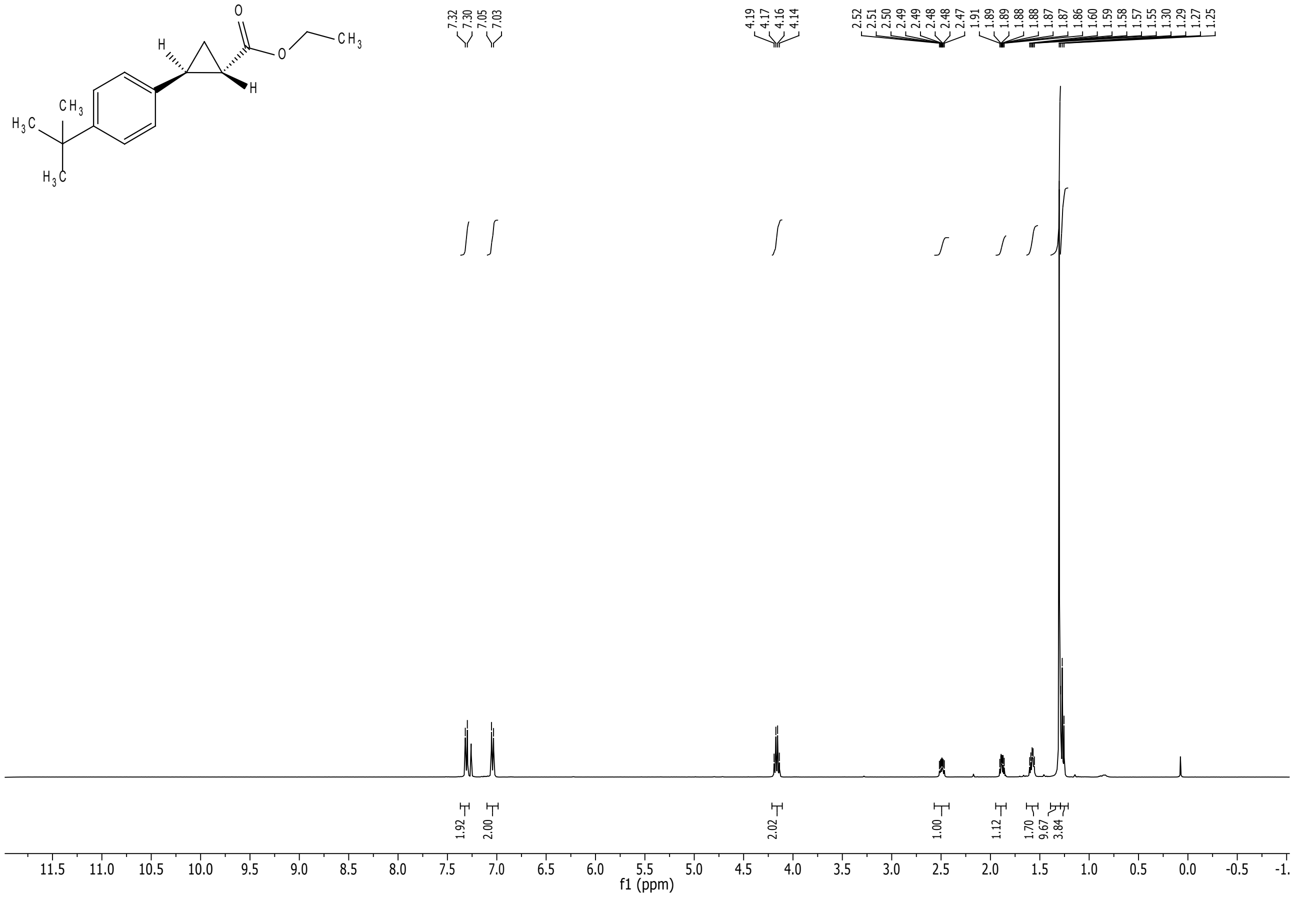
7.32
7.30
7.05
7.03
4.19
4.17
4.16
4.14
2.52
2.51
2.50
2.49
2.48
2.48
2.47
1.91
1.89
1.89
1.88
1.88
1.87
1.86
1.60
1.59
1.58
1.57
1.55
1.30
1.29
1.27
1.25
O
O
CH_3
H
H
CH_3
H_3C
H_3C
1.92
2.00
2.02
1.00
1.12
1.70
9.67
3.84
11.5 11.0 10.5 10.0 9.5 9.0 8.5 8.0 7.5 7.0 6.5 6.0 5.5 5.0 4.5 4.0 3.5 3.0 2.5 2.0 1.5 1.0 0.5 0.0 -0.5 -1.
f1 (ppm)

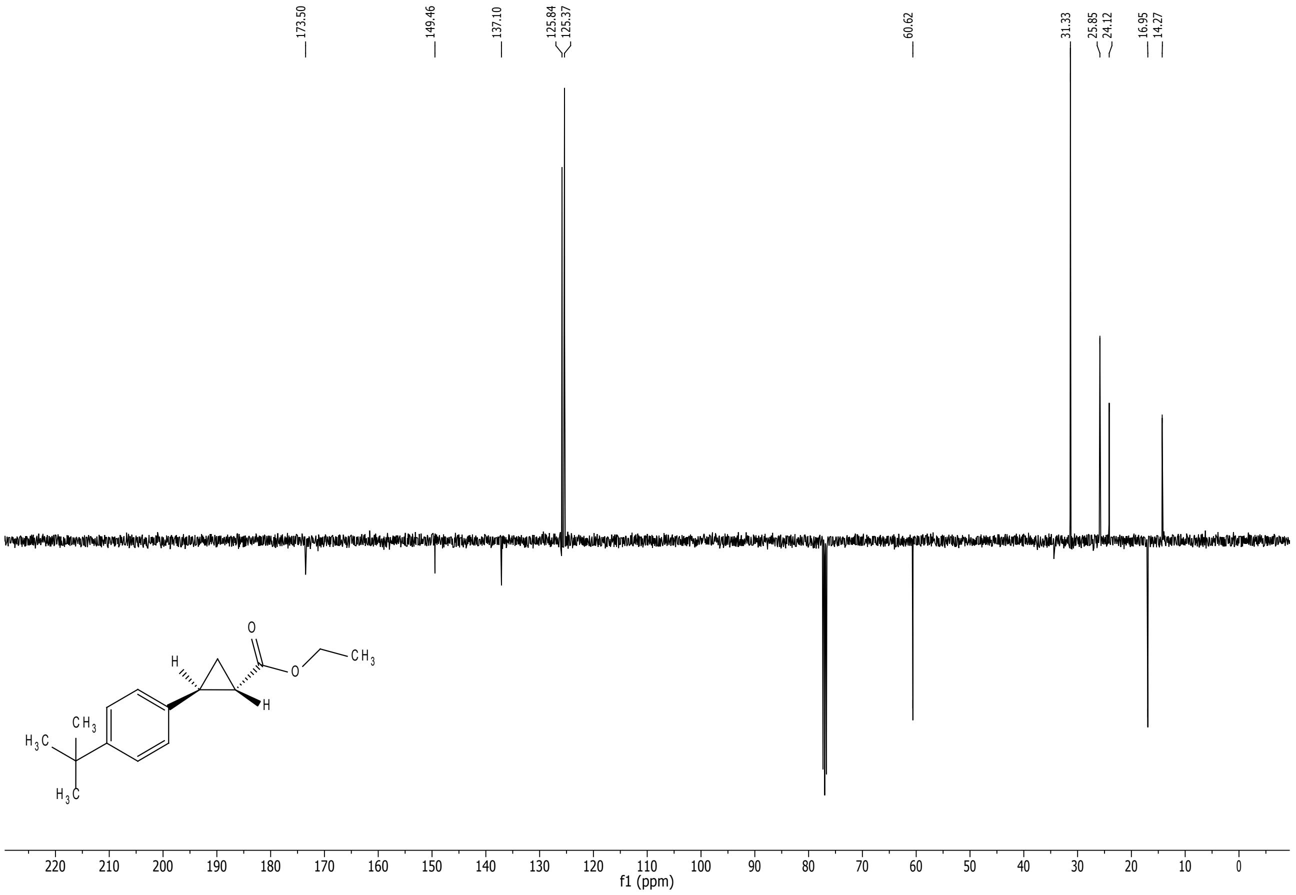
173.50
149.46
137.10
125.84
125.37
60.62
31.33
25.85
24.12
16.95
14.27
f1 (ppm)

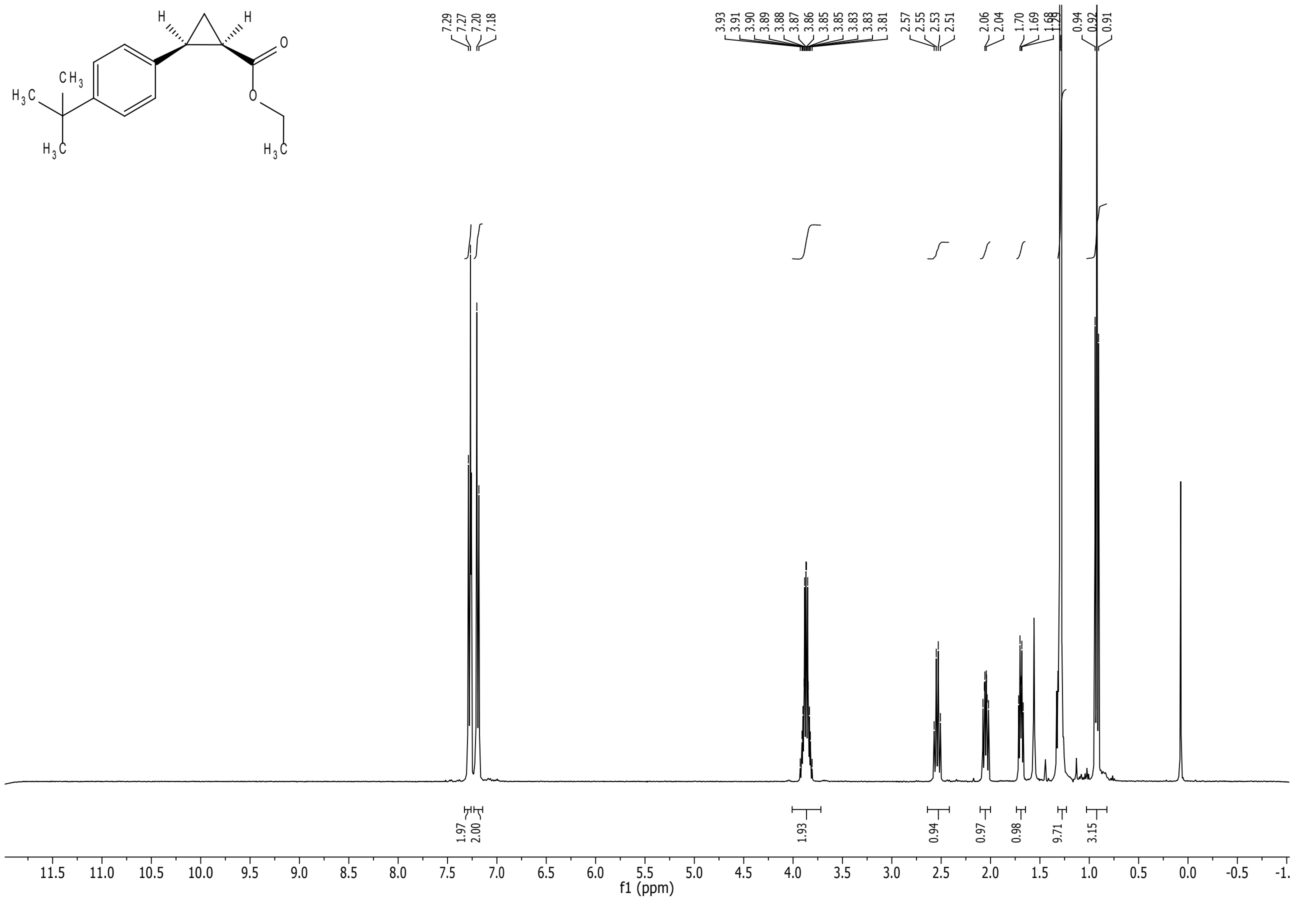
7.29
7.27
7.20
7.18
3.93
3.91
3.90
3.89
3.88
3.87
3.86
3.85
3.85
3.83
3.83
3.81
2.57
2.55
2.53
2.51
2.06
2.04
1.70
1.69
0.94
0.92
0.91
1.97
2.00
1.93
0.94
0.97
0.98
9.71
3.15
f1 (ppm)

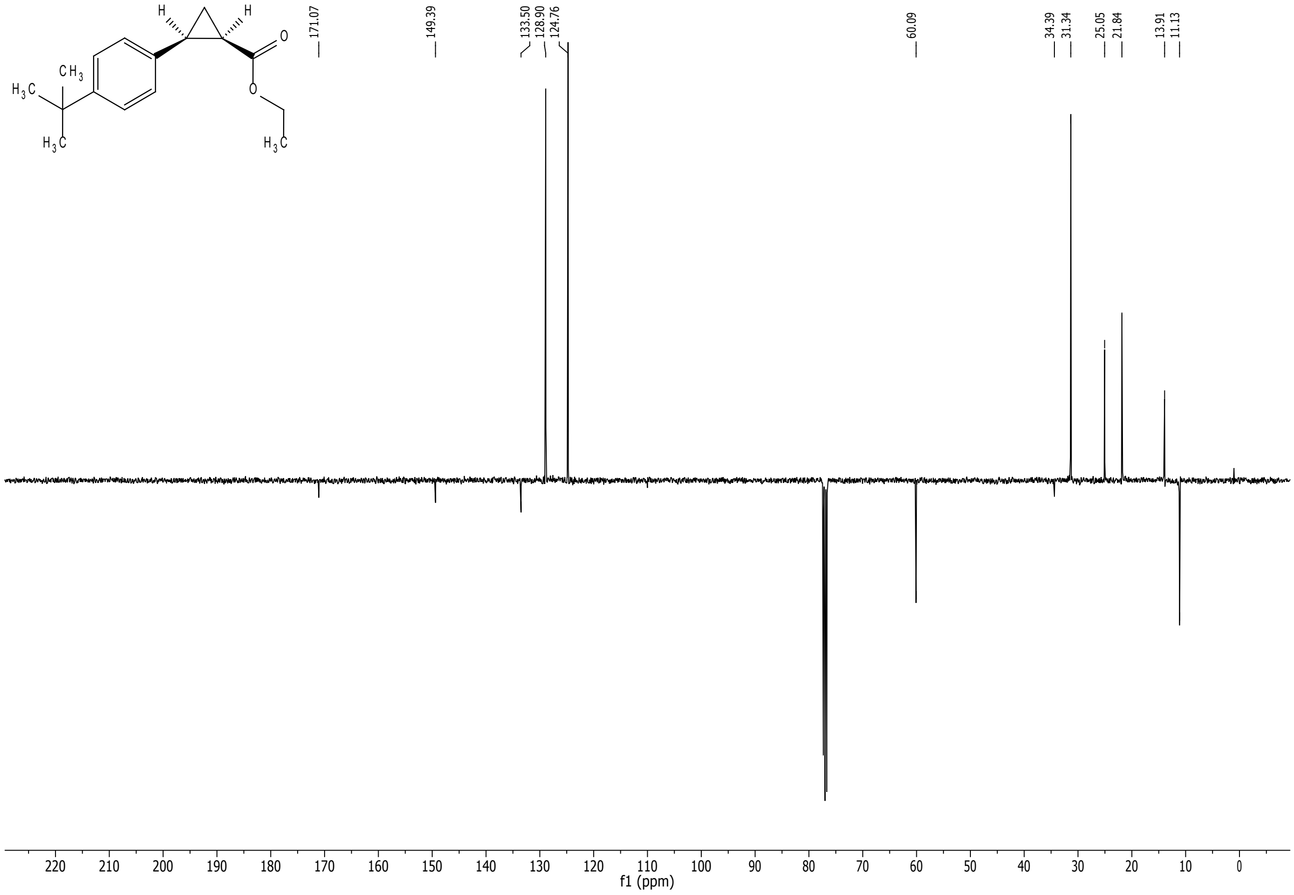

171.07
149.39
133.50
128.90
124.76
60.09
34.39
31.34
25.05
21.84
13.91
11.13
f1 (ppm)

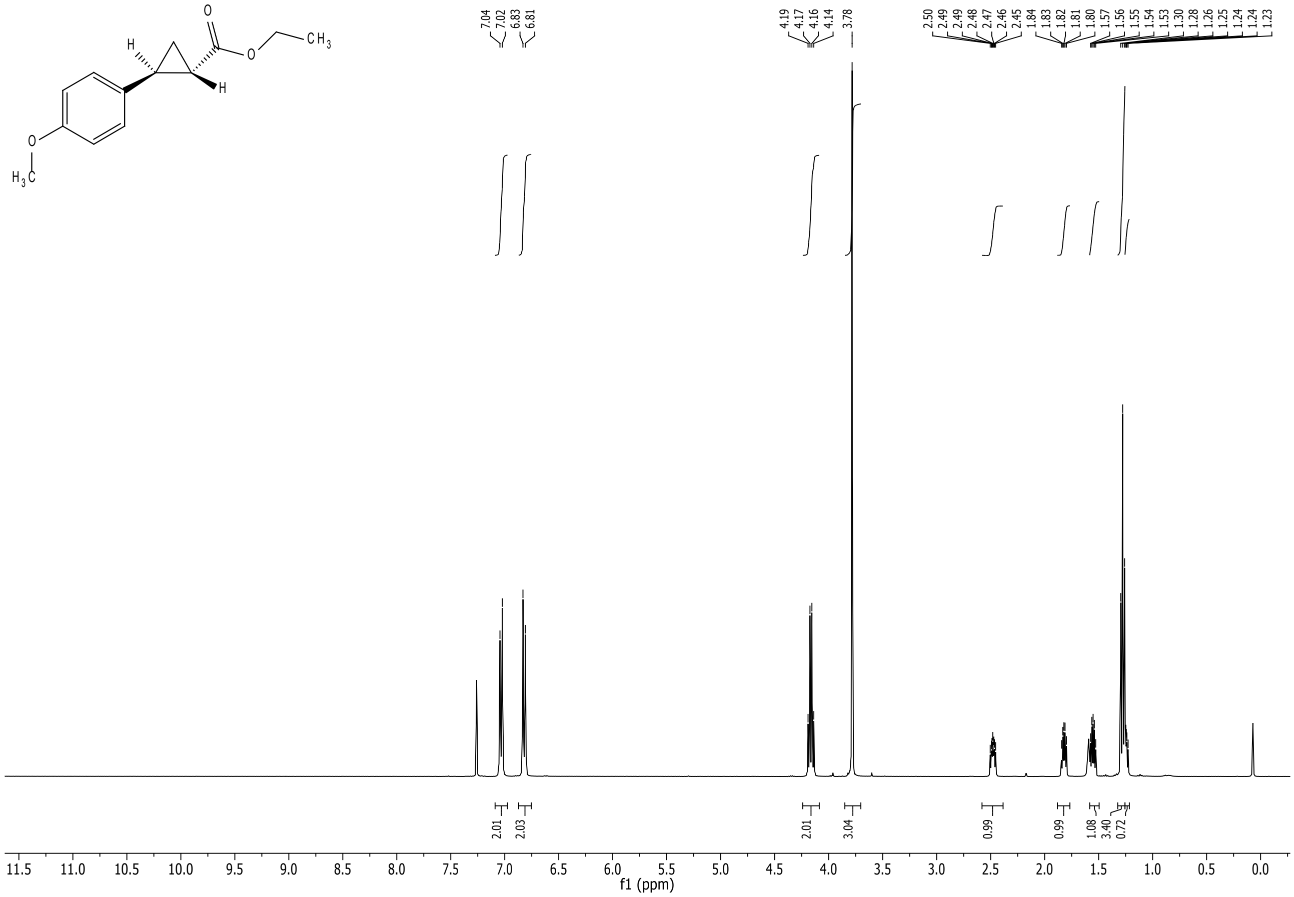
O
H
H
O
CH3
O
H3C
7.04
7.02
6.83
6.81
4.19
4.17
4.16
4.14
3.78
2.50
2.49
2.49
2.48
2.47
2.46
2.45
1.84
1.83
1.82
1.81
1.80
1.57
1.56
1.55
1.54
1.53
1.30
1.28
1.26
1.25
1.24
1.24
1.23
2.01
2.03
2.01
3.04
0.99
0.99
1.08
3.40
0.72
f1 (ppm)

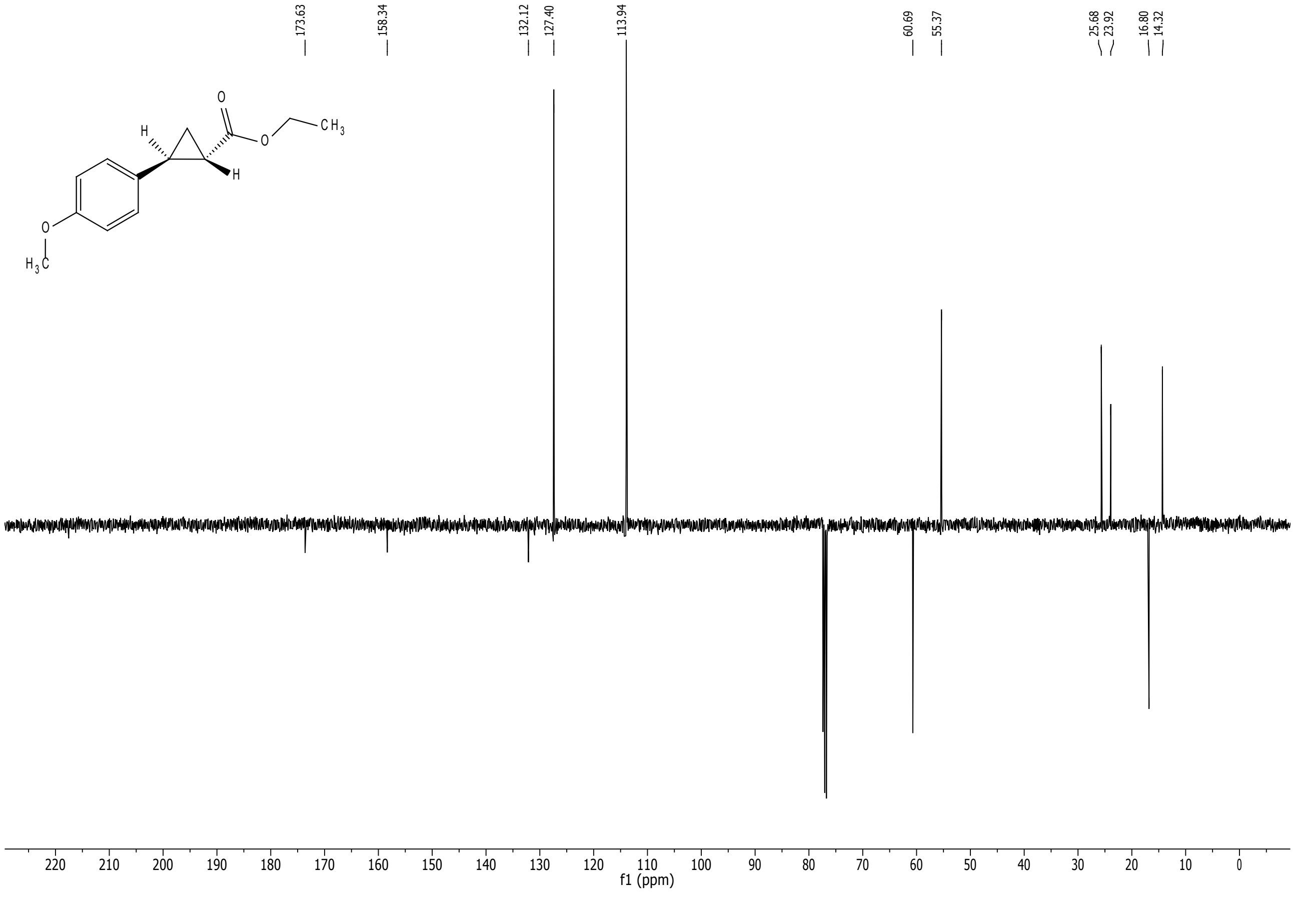
173.63
158.34
132.12
127.40
113.94
60.69
55.37
25.68
23.92
16.80
14.32
O
H
H
O
CH3
O
H3C
f1 (ppm)

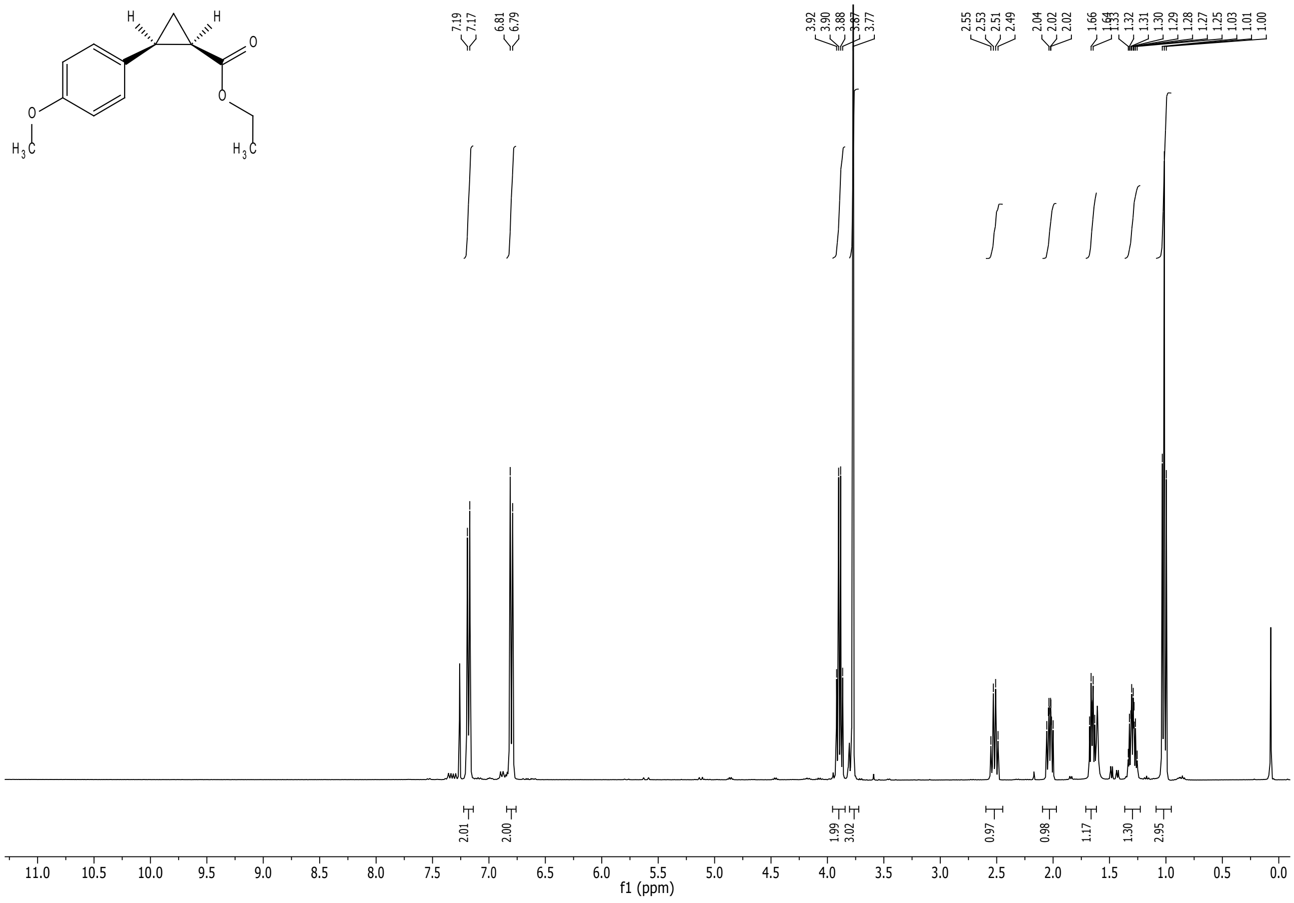
H
H
O
O
O
H_3C
H_3C
7.19
7.17
6.81
6.79
3.92
3.90
3.88
3.87
3.77
2.55
2.53
2.51
2.49
2.04
2.02
2.02
1.66
1.64
1.33
1.32
1.31
1.30
1.29
1.28
1.27
1.25
1.03
1.01
1.00
2.01
2.00
1.99
3.02
0.97
0.98
1.17
1.30
2.95
f1 (ppm)

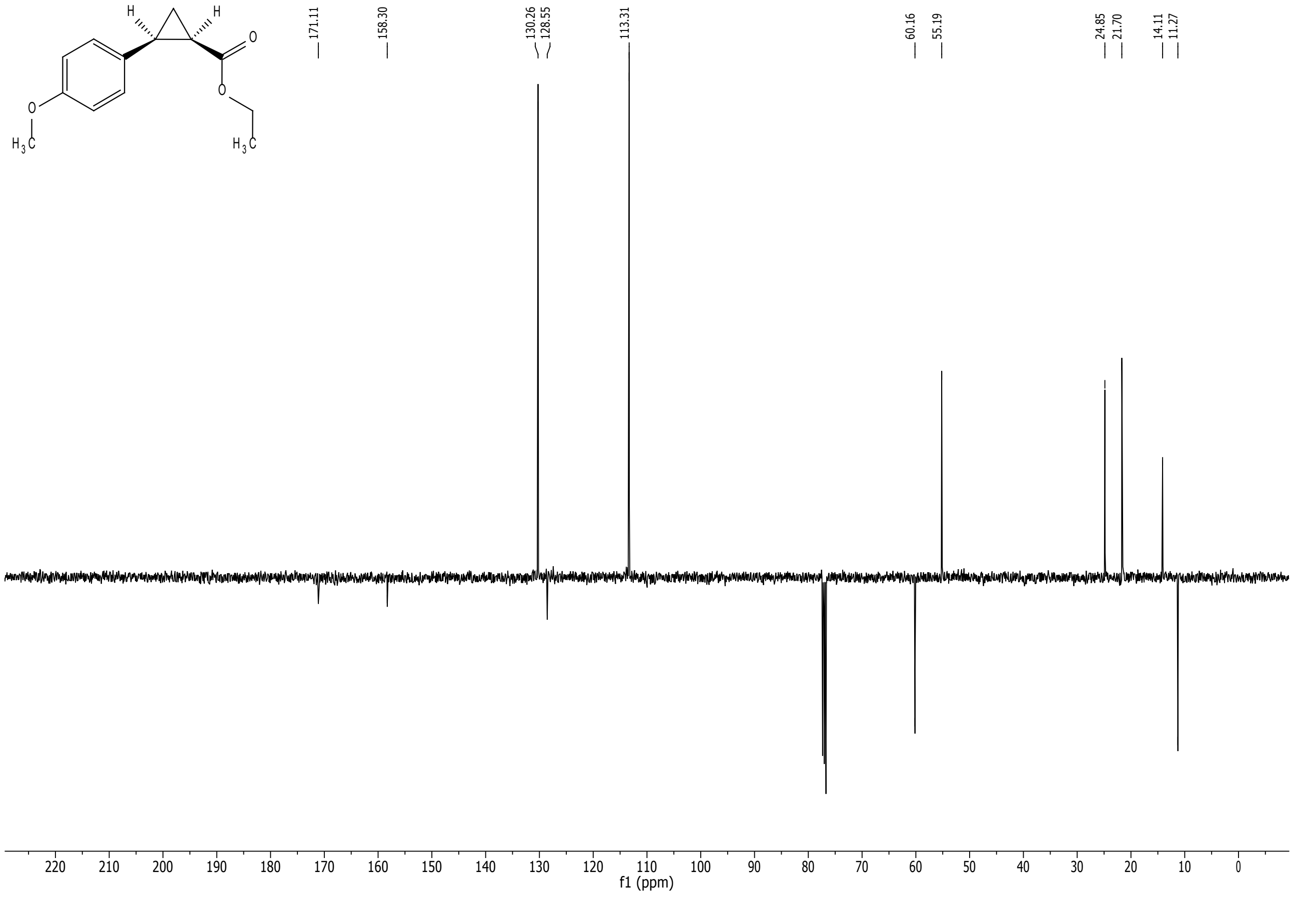
H
H
O
O
O
H_3C
H_3C
171.11
158.30
130.26
128.55
113.31
60.16
55.19
24.85
21.70
14.11
11.27
f1 (ppm)

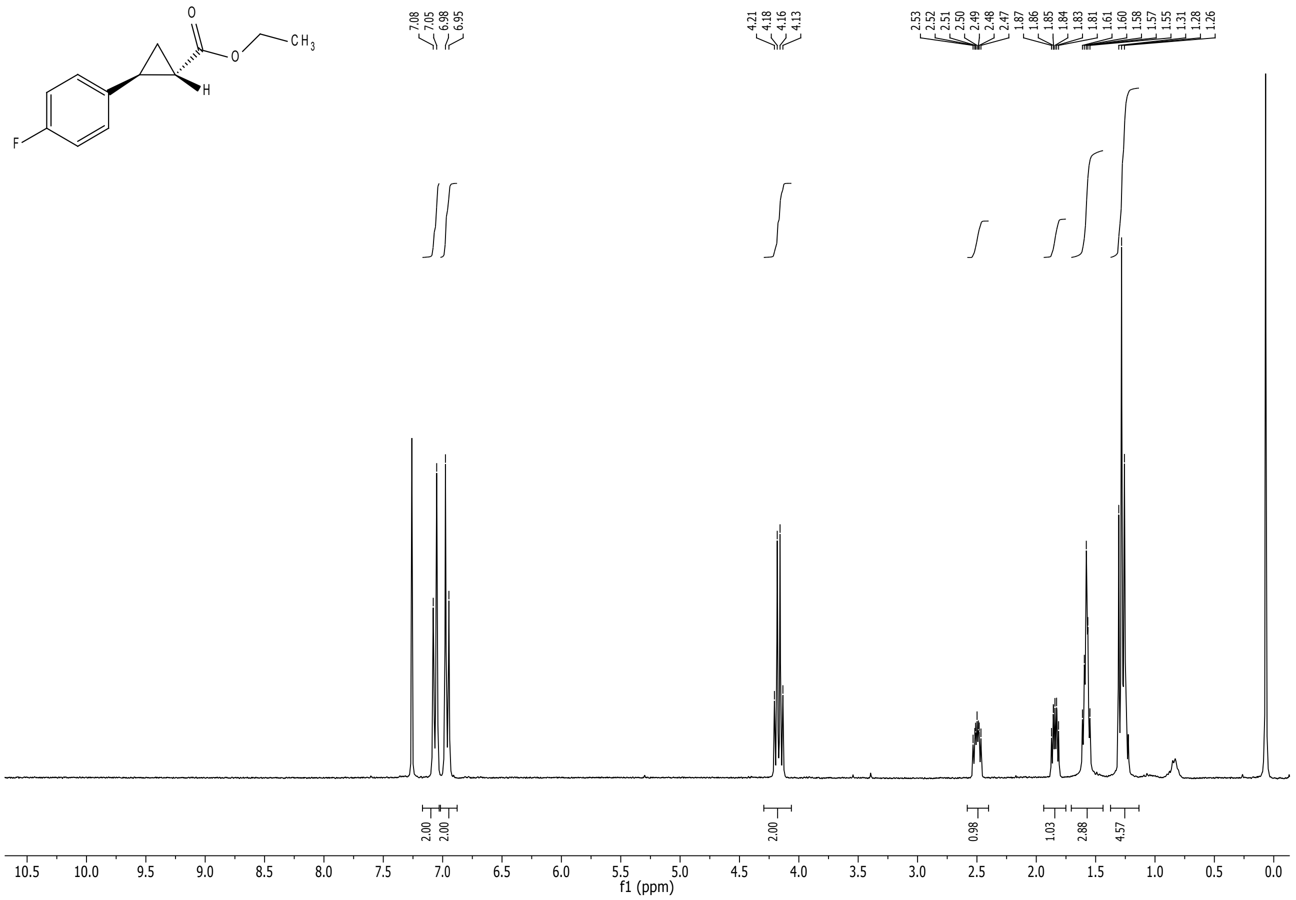
7.08
7.05
6.98
6.95
4.21
4.18
4.16
4.13
2.53
2.52
2.51
2.50
2.49
2.48
2.47
1.87
1.86
1.85
1.84
1.83
1.81
1.61
1.60
1.58
1.57
1.55
1.31
1.28
1.26
2.00
2.00
2.00
0.98
1.03
2.88
4.57
10.5 10.0 9.5 9.0 8.5 8.0 7.5 7.0 6.5 6.0 5.5 5.0 4.5 4.0 3.5 3.0 2.5 2.0 1.5 1.0 0.5 0.0
f1 (ppm)

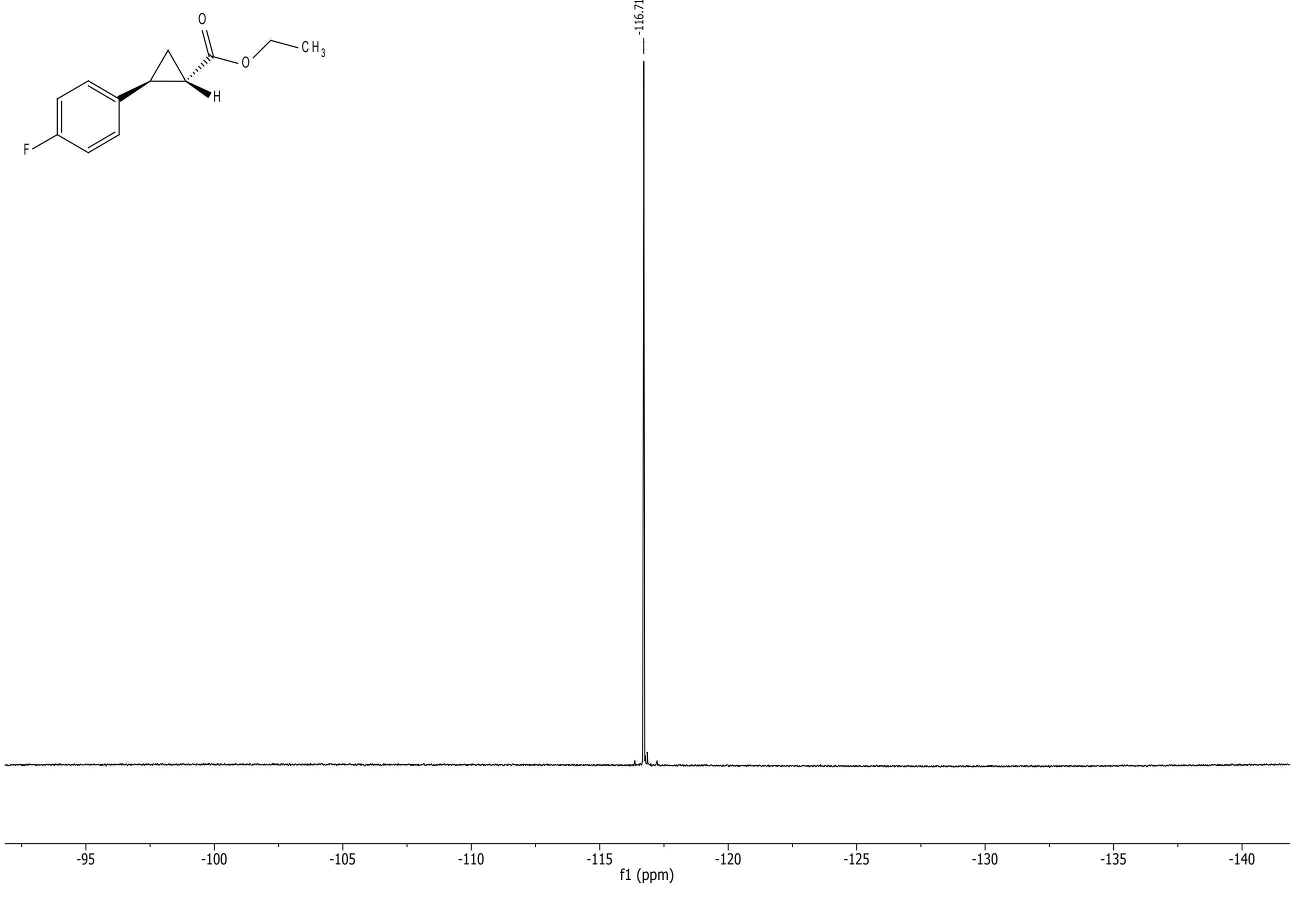
-116.71
-95 -100 -105 -110 -115 -120 -125 -130 -135 -140
f1 (ppm)

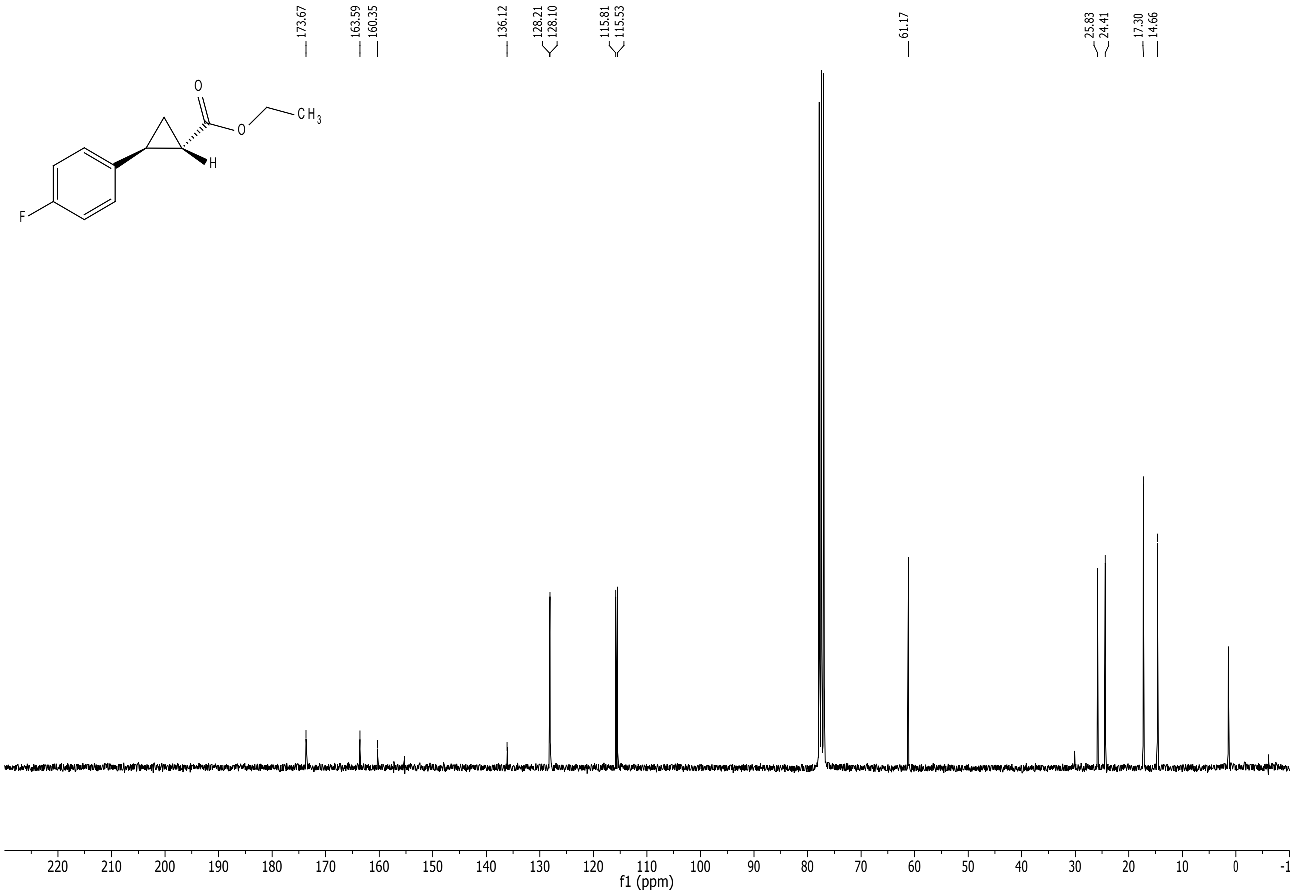
173.67
163.59
160.35
136.12
128.21
128.10
115.81
115.53
61.17
25.83
24.41
17.30
14.66
O
O
CH3
H
F
220 210 200 190 180 170 160 150 140 130 120 110 100 90 80 70 60 50 40 30 20 10 0 -1
f1 (ppm)

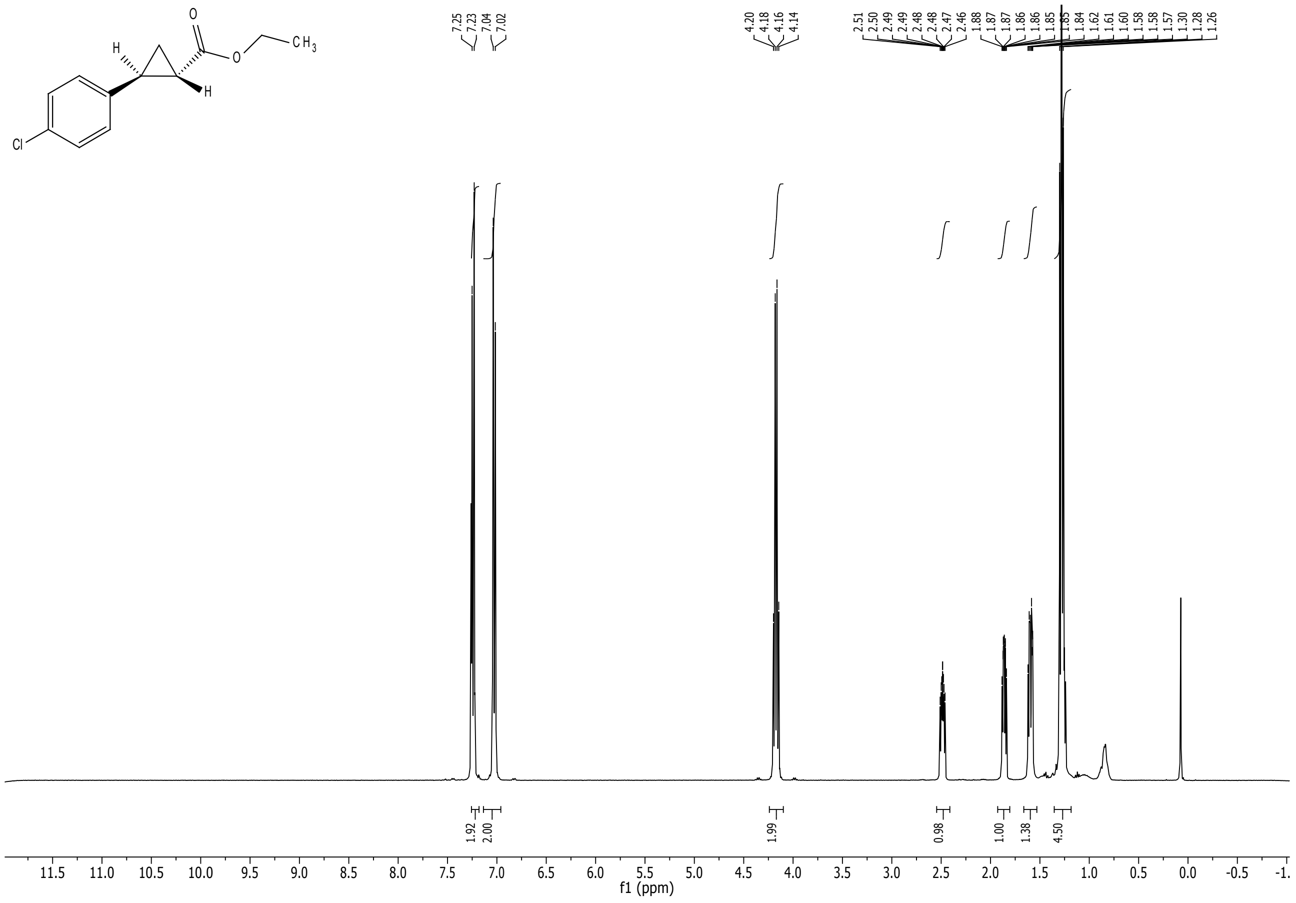

O
H
O
CH3
H
Cl
7.25
7.23
7.04
7.02
4.20
4.18
4.16
4.14
2.51
2.50
2.49
2.49
2.48
2.48
2.47
2.46
1.88
1.87
1.87
1.86
1.86
1.85
1.85
1.84
1.62
1.61
1.60
1.58
1.57
1.30
1.28
1.26
1.92
2.00
1.99
0.98
1.00
1.38
4.50
f1 (ppm)

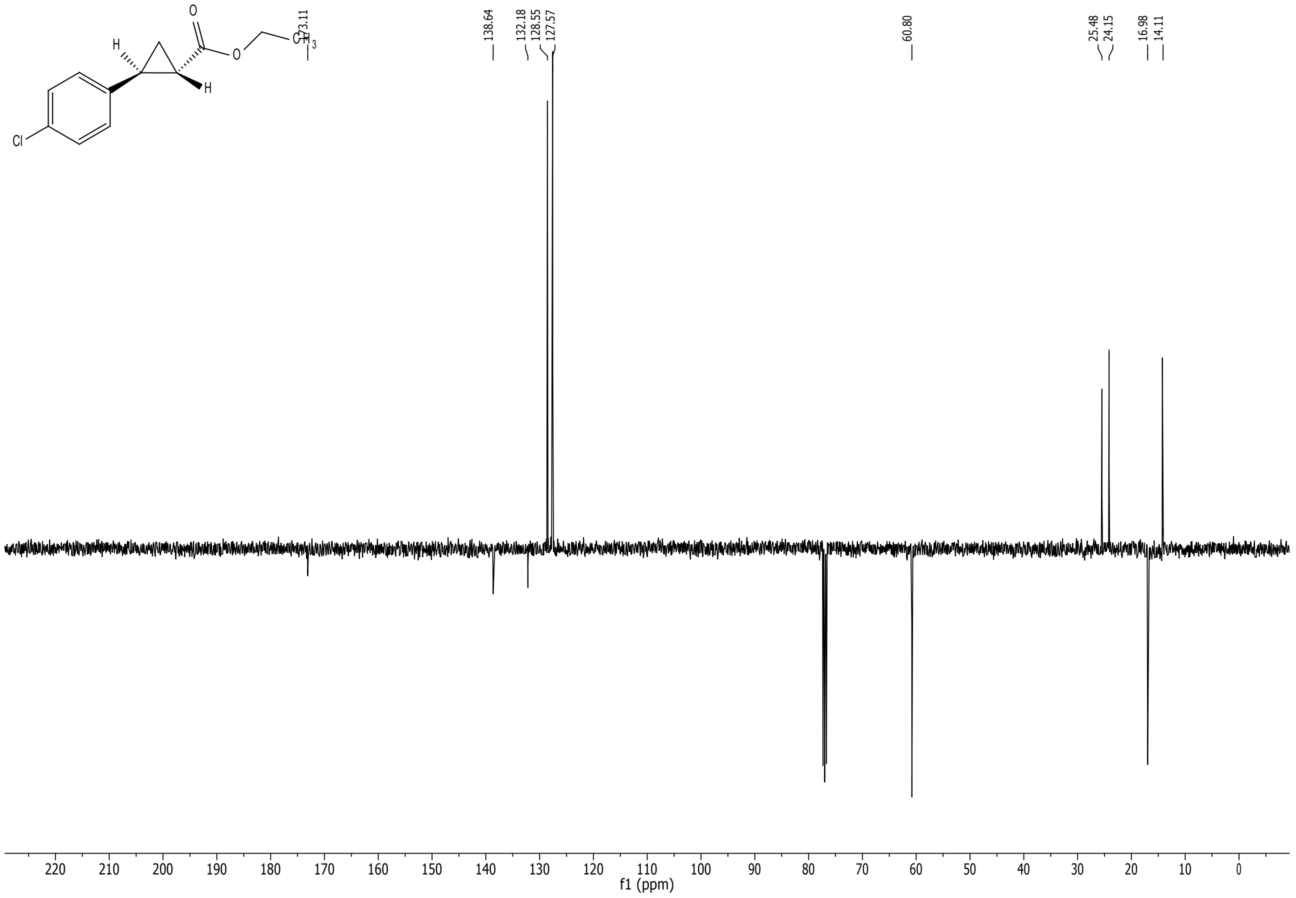

O
H
O
CH3
H
Cl
173.11
138.64
132.18
128.55
127.57
60.80
25.48
24.15
16.98
14.11
f1 (ppm)

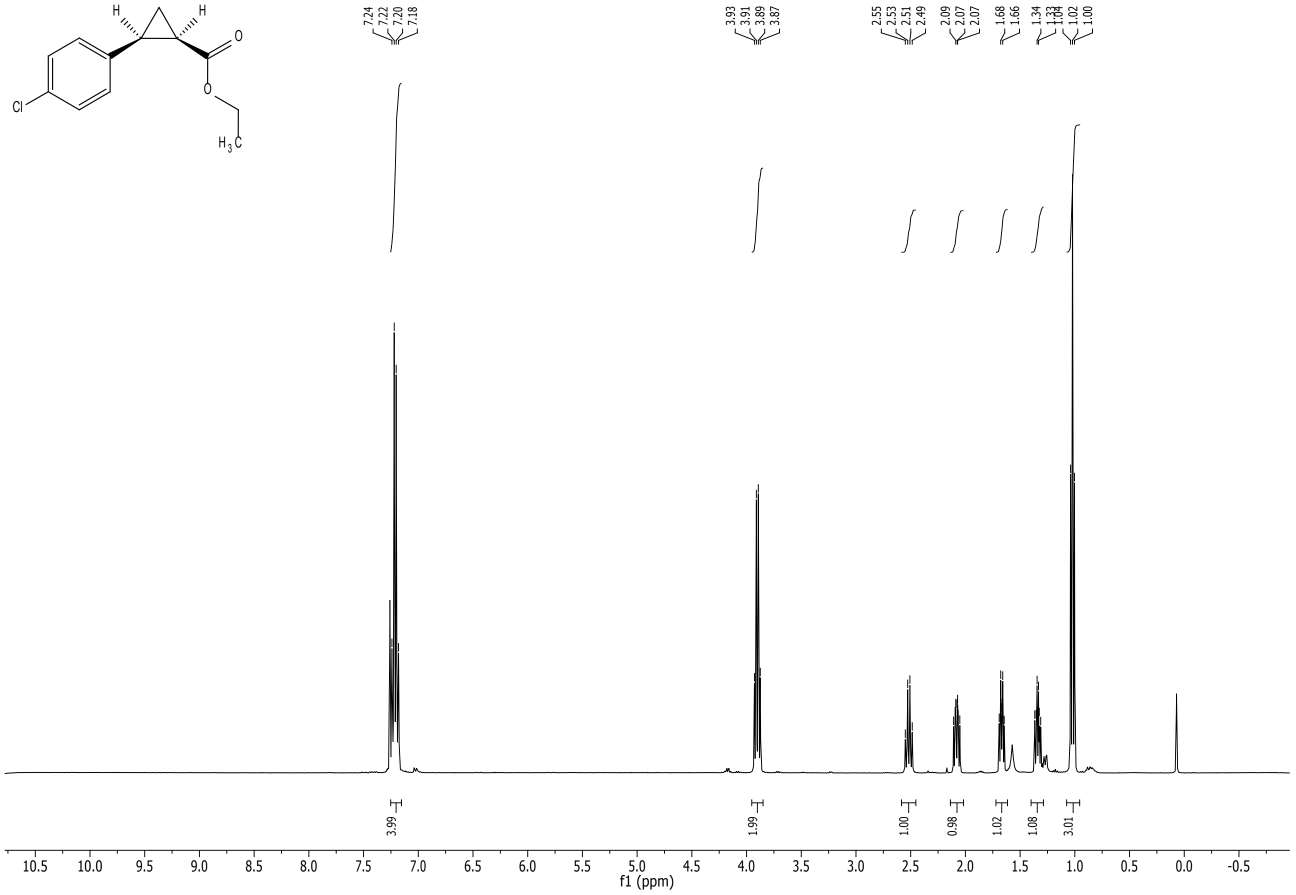
H
H
O
O
Cl
H_3C
7.24
7.22
7.20
7.18
3.93
3.91
3.89
3.87
2.55
2.53
2.51
2.49
2.09
2.07
2.07
1.68
1.66
1.34
1.33
1.04
1.02
1.00
3.99
1.99
1.00
0.98
1.02
1.08
3.01
f1 (ppm)

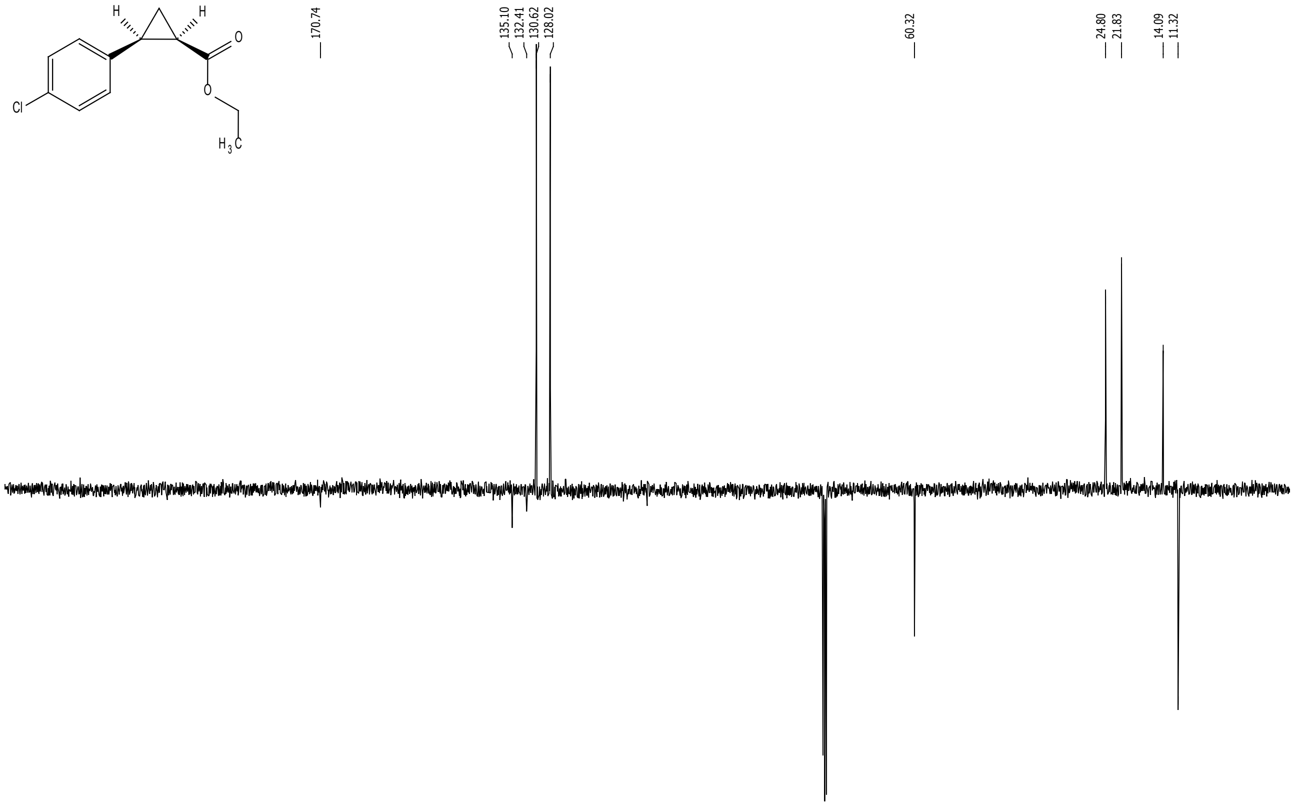
H
H
O
O
Cl
H_3C
170.74
135.10
132.41
130.62
128.02
60.32
24.80
21.83
14.09
11.32
f1 (ppm)

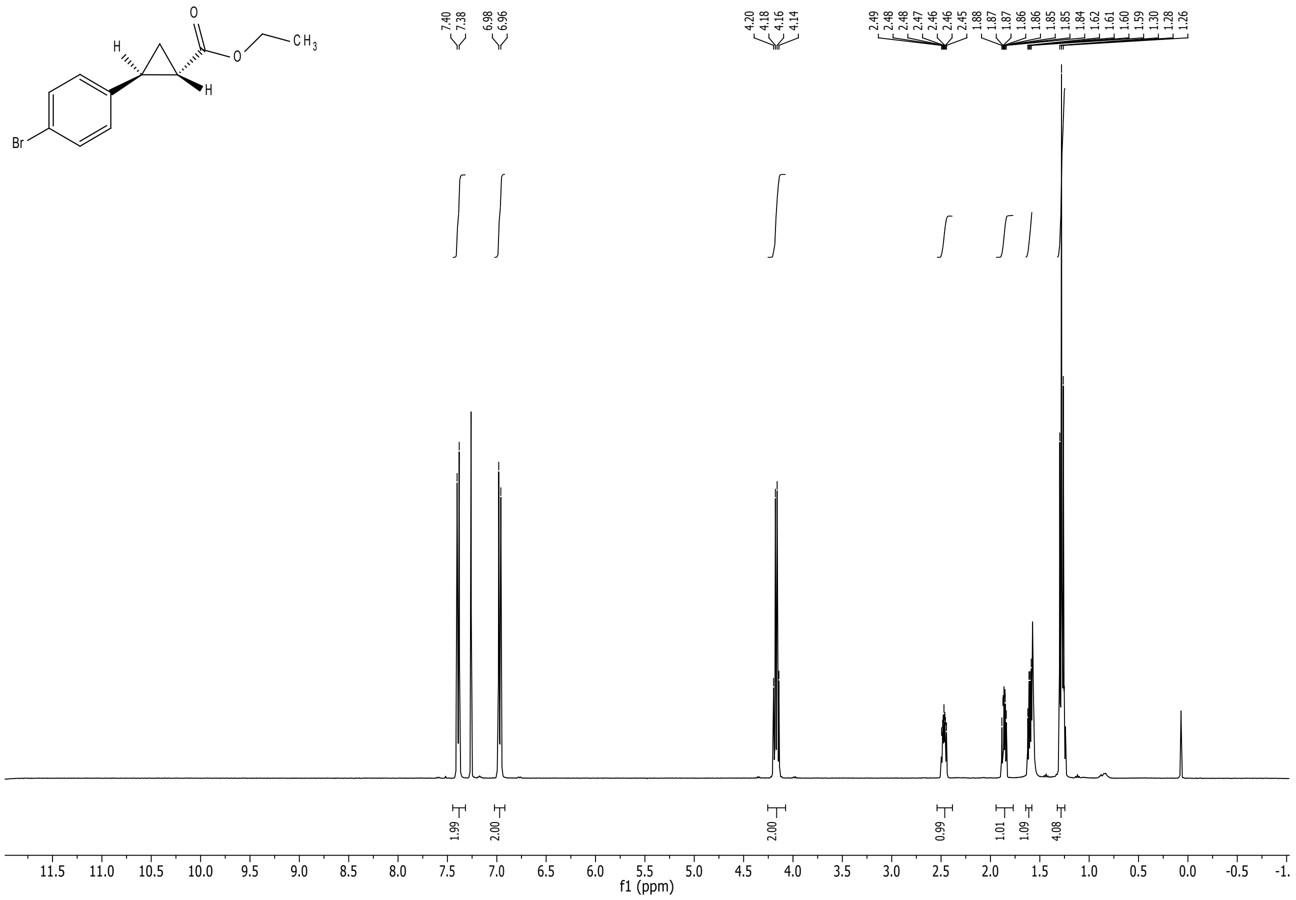

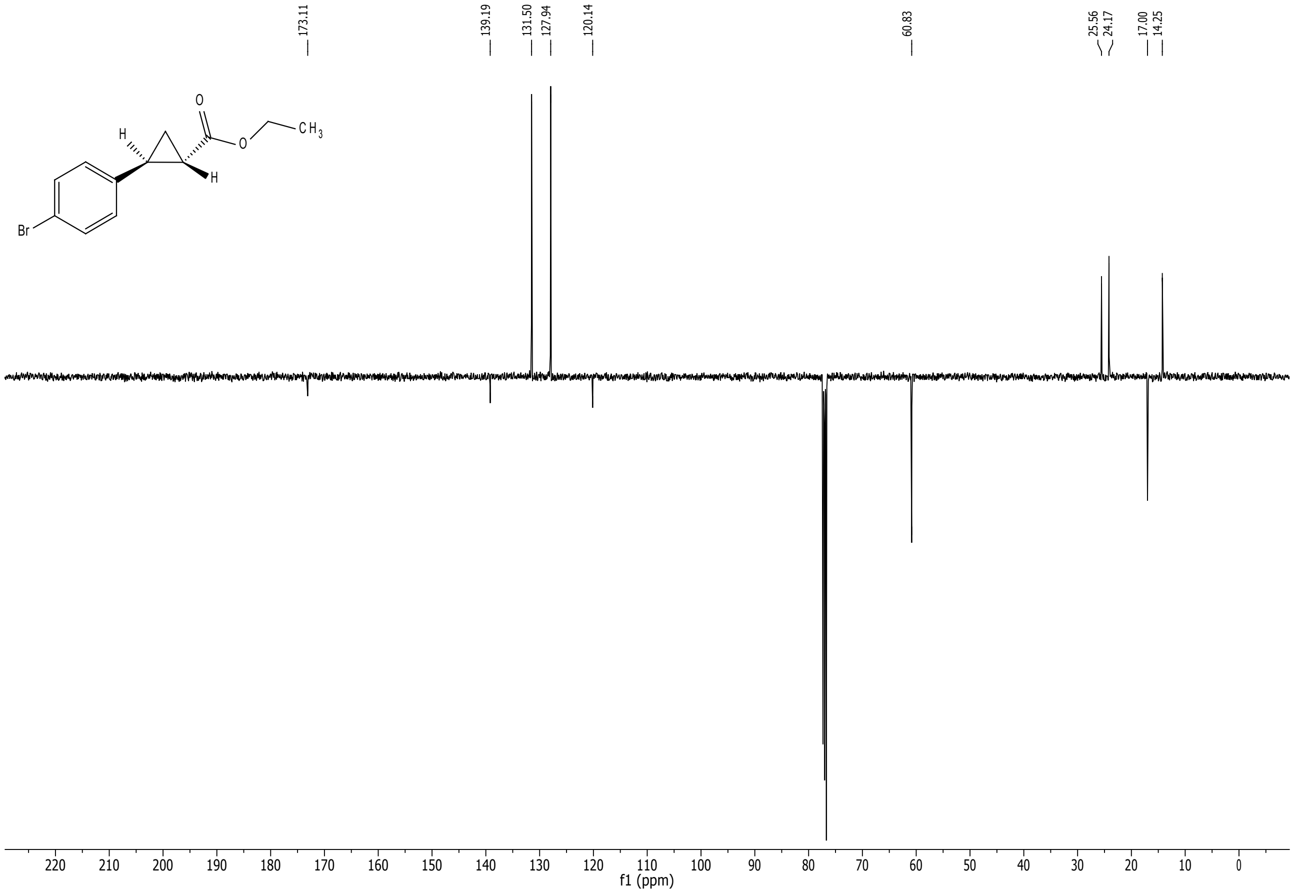

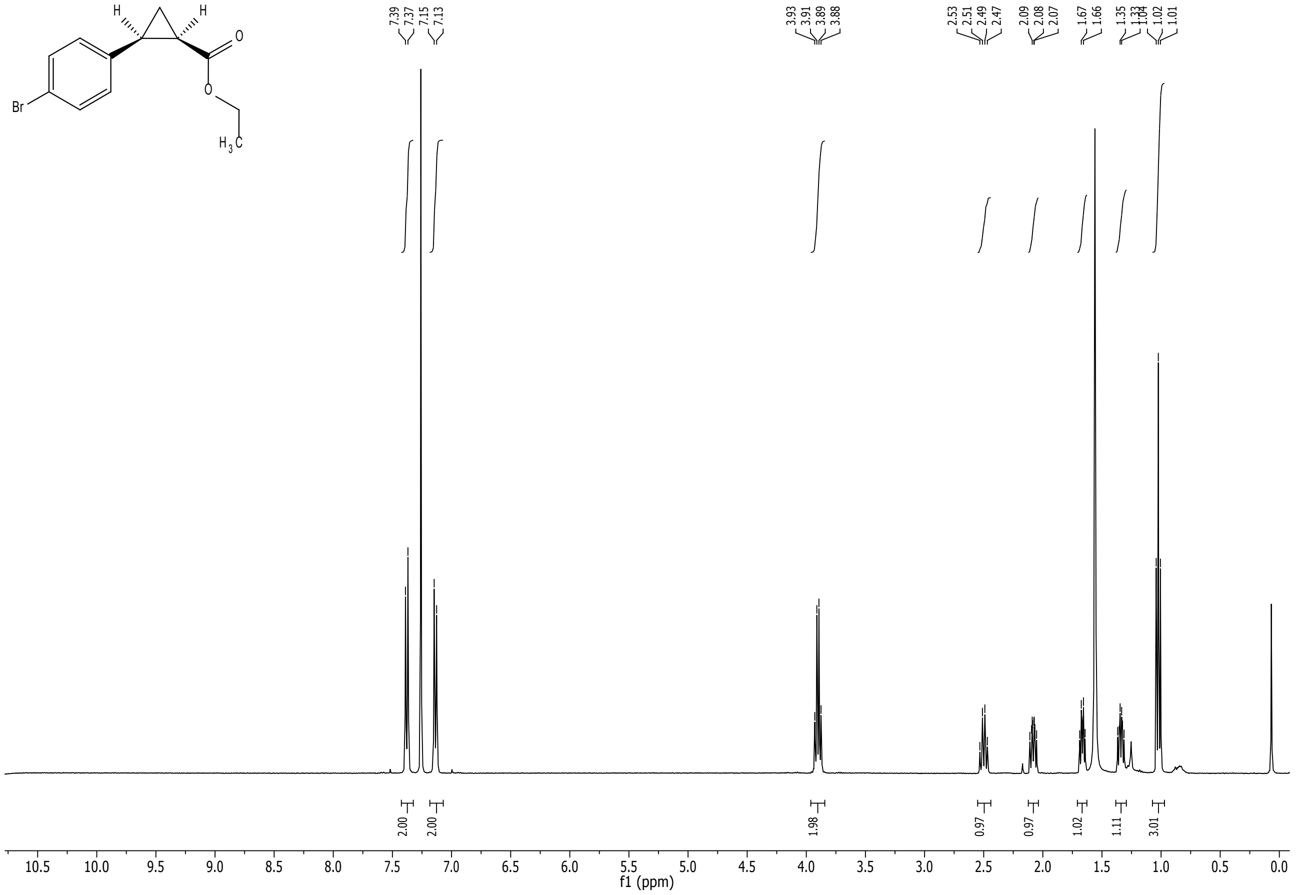
7.39
7.37
7.15
7.13
3.93
3.91
3.89
3.88
2.53
2.51
2.49
2.47
2.09
2.08
2.07
1.67
1.66
1.35
1.33
1.04
1.02
1.01
2.00
2.00
1.98
0.97
0.97
1.02
1.11
3.01
f1 (ppm)

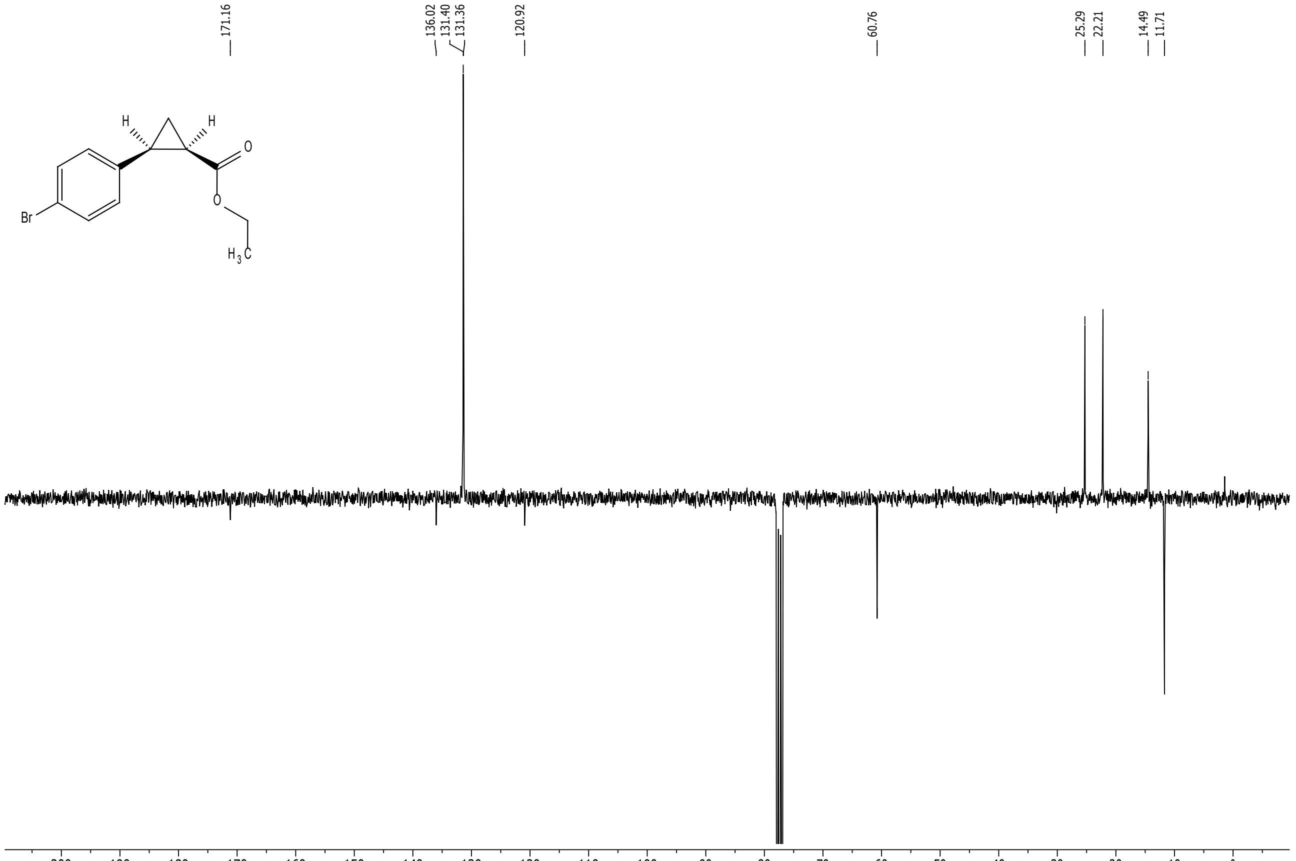
171.16
136.02
131.40
131.36
120.92
60.76
25.29
22.21
14.49
11.71
f1 (ppm)

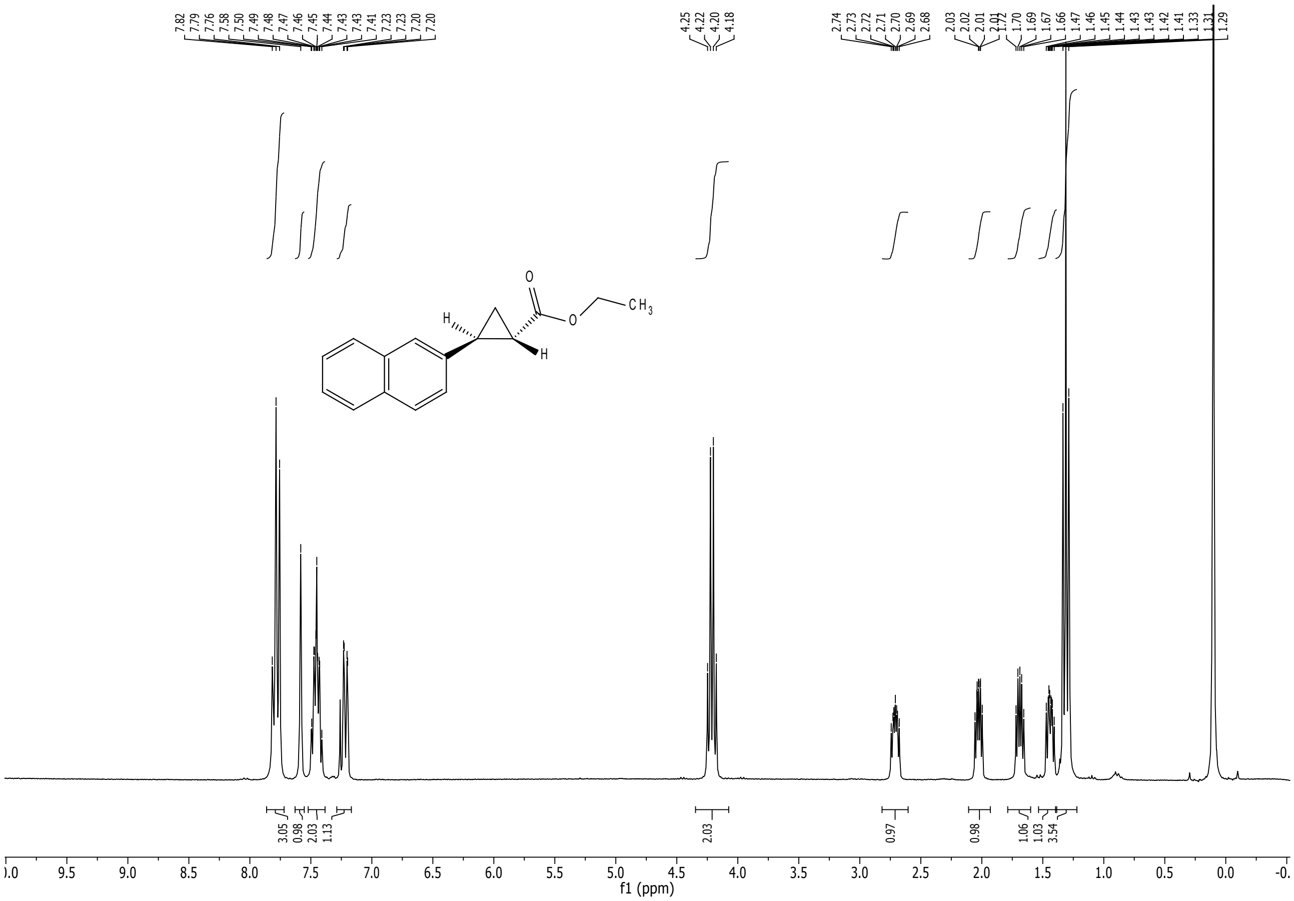
7.82 7.79 7.76 7.58 7.50 7.49 7.48 7.47 7.46 7.45 7.44 7.43 7.43 7.41 7.23 7.23 7.20 7.20
4.25 4.22 4.20 4.18
2.74 2.73 2.72 2.71 2.70 2.69 2.68
2.03 2.02 2.01 2.01 1.72 1.70 1.69 1.67 1.66 1.47 1.46 1.45 1.44 1.43 1.42 1.41 1.33 1.31 1.29
H
O
O
CH3
H
3.05 0.98 2.03 1.13
2.03
0.97
0.98
1.06 1.03 3.54
f1 (ppm)

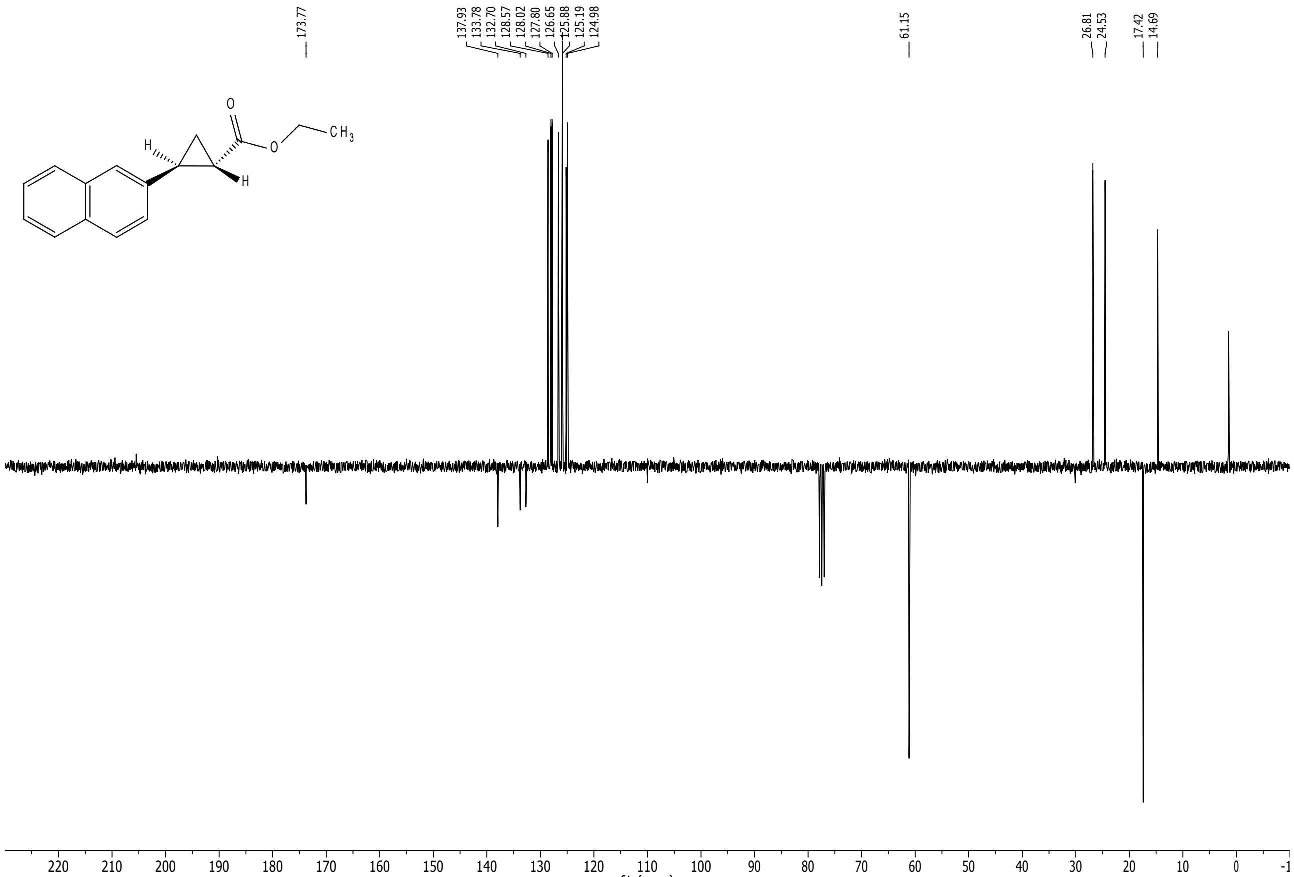
173.77
137.93 133.78 132.70 128.57 128.02 127.80 126.65 125.88 125.19 124.98
61.15
26.81 24.53
17.42 14.69
H
O
O
CH3
H
f1 (ppm)

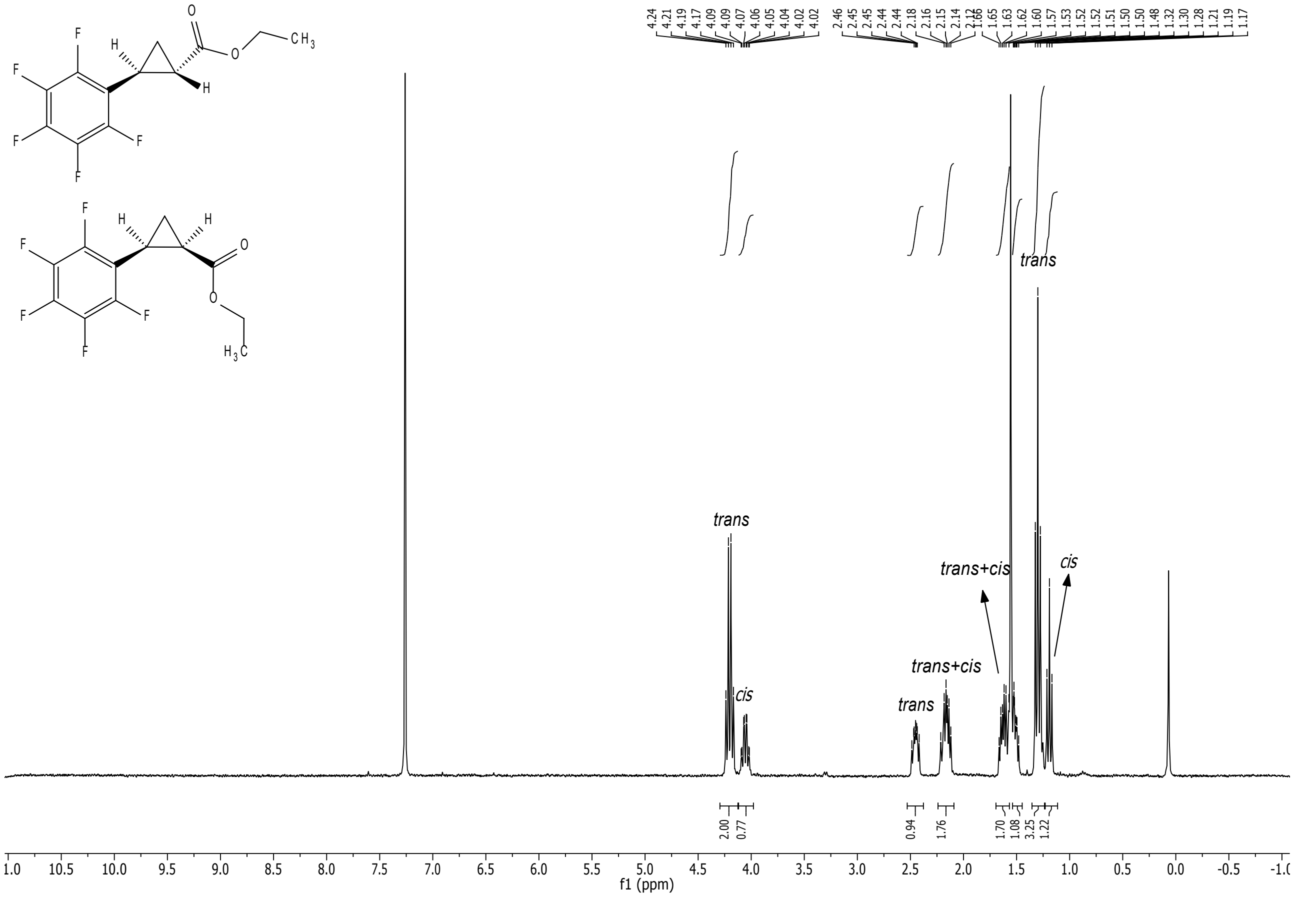
4.24 4.21 4.19 4.17 4.09 4.07 4.06 4.05 4.04 4.02 4.02
2.46 2.45 2.45 2.44 2.44 2.18 2.16 2.15 2.14 2.12
1.66 1.65 1.63 1.62 1.60 1.57 1.53 1.52 1.51 1.50 1.50 1.48 1.32 1.30 1.28 1.21 1.19 1.17
trans
cis
trans
trans+cis
trans+cis
trans
cis
2.00 0.77 0.94 1.76 1.70 1.08 3.25 1.22
f1 (ppm)

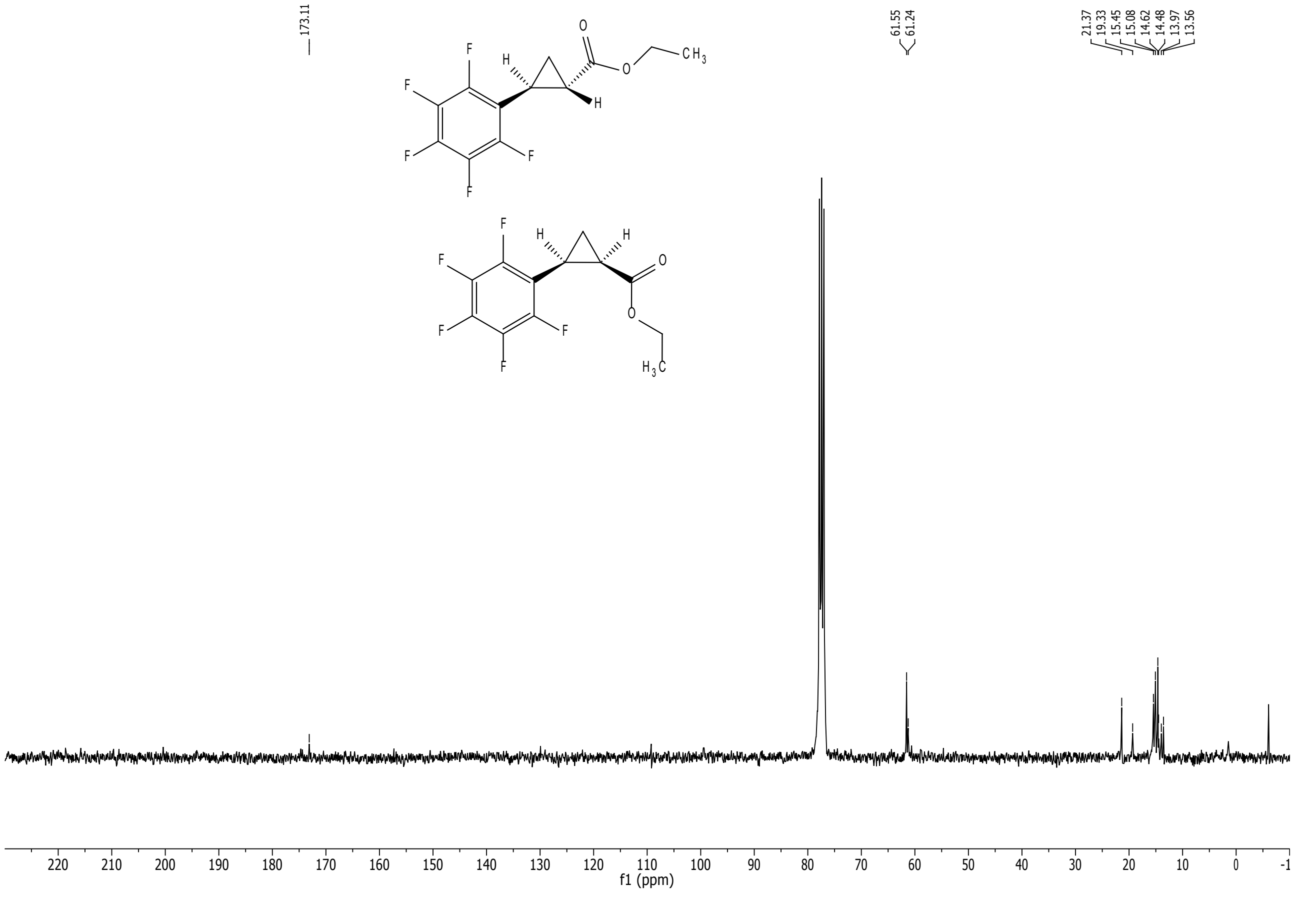
173.11
61.55 61.24
21.37 19.33 15.45 15.08 14.62 14.48 13.97 13.56
f1 (ppm)

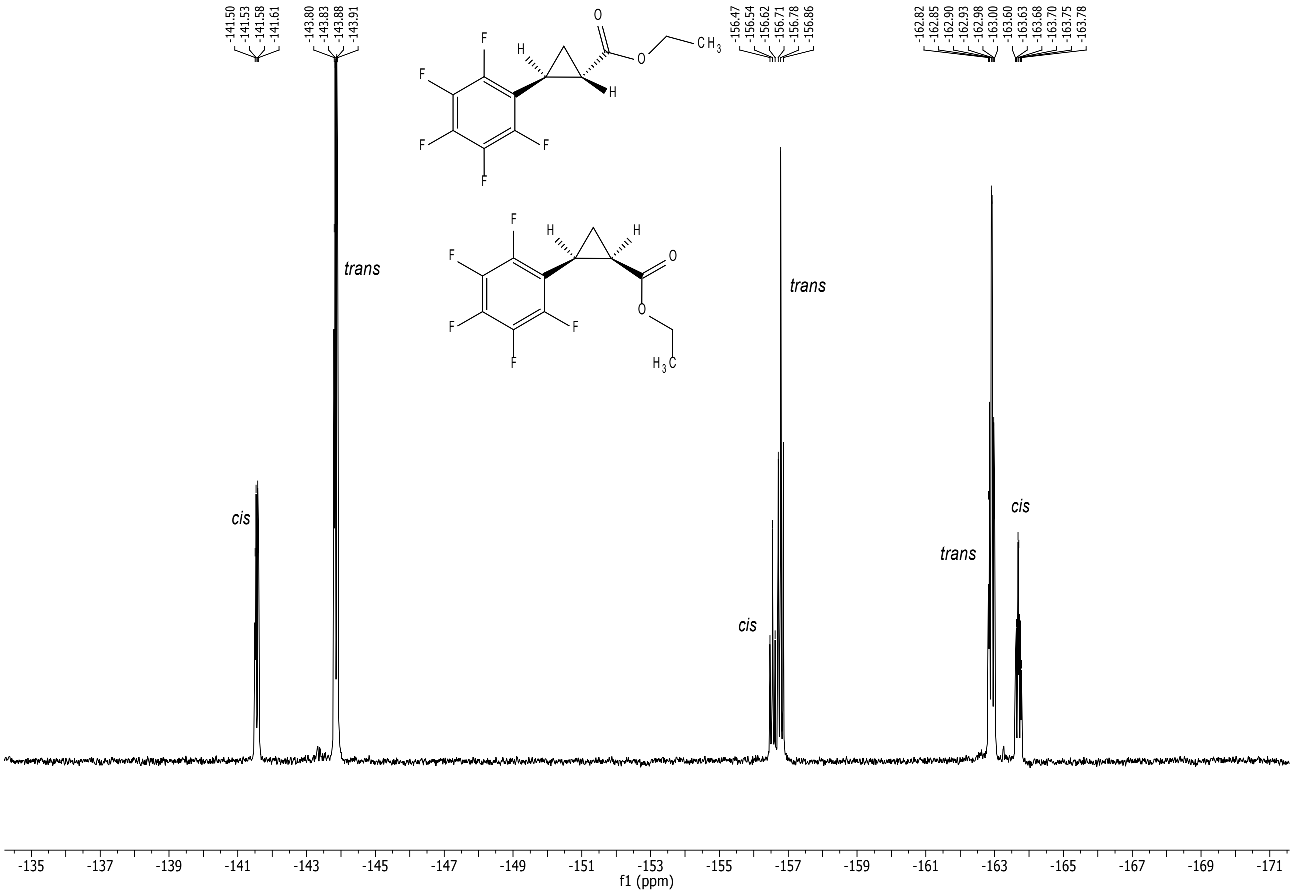
-141.50
-141.53
-141.58
-141.61
-143.80
-143.83
-143.88
-143.91
-156.47
-156.54
-156.62
-156.71
-156.78
-156.86
-162.82
-162.85
-162.90
-162.93
-162.98
-163.00
-163.60
-163.63
-163.68
-163.70
-163.75
-163.78
cis
trans
cis
trans
trans
cis
-135
-137
-139
-141
-143
-145
-147
-149
-151
-153
-155
-157
-159
-161
-163
-165
-167
-169
-171
f1 (ppm)

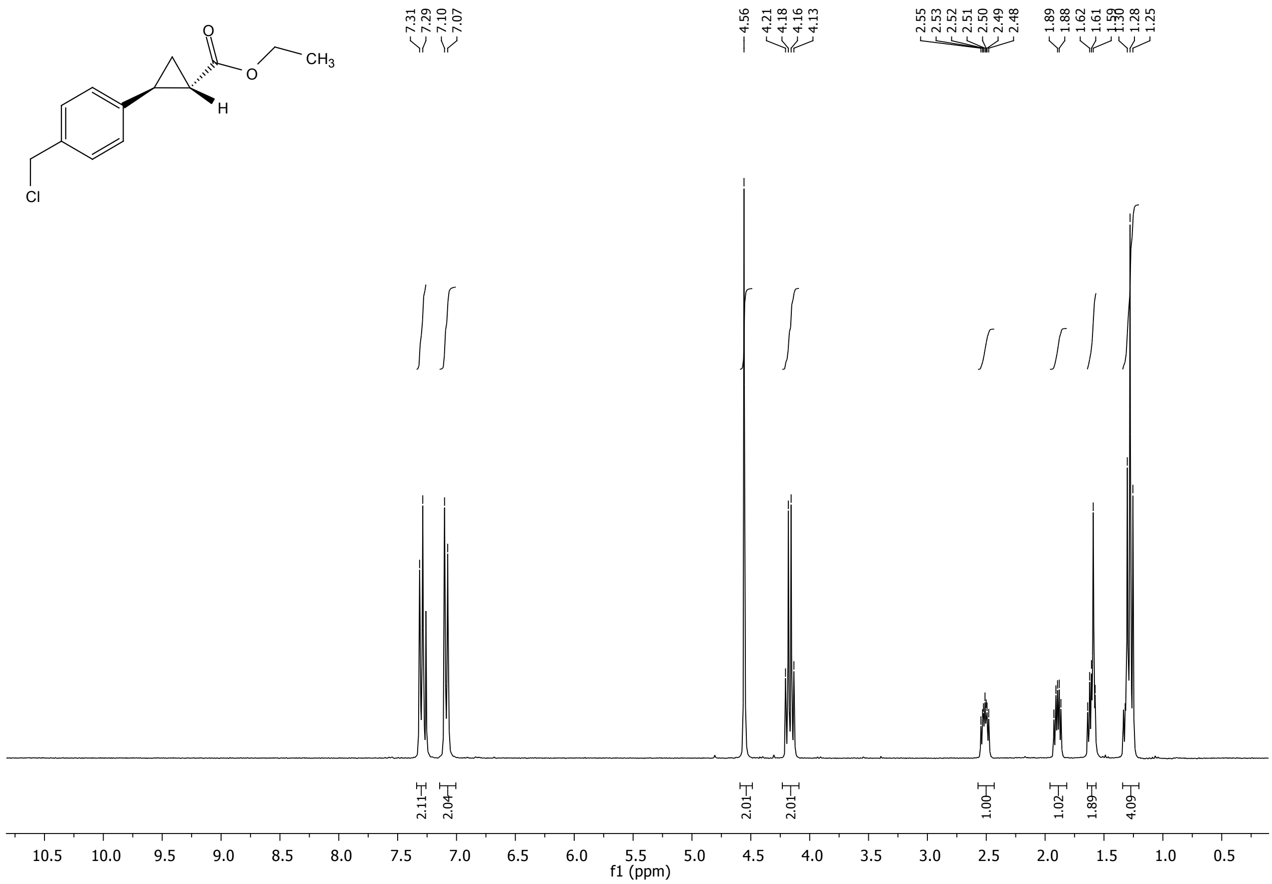
7.31
7.29
7.10
7.07
4.56
4.21
4.18
4.16
4.13
2.55
2.53
2.52
2.51
2.50
2.49
2.48
1.89
1.88
1.62
1.61
1.28
1.25
O
O
CH3
H
Cl
2.11
2.04
2.01
2.01
1.00
1.02
1.89
4.09
10.5
10.0
9.5
9.0
8.5
8.0
7.5
7.0
6.5
6.0
5.5
5.0
4.5
4.0
3.5
3.0
2.5
2.0
1.5
1.0
0.5
f1 (ppm)

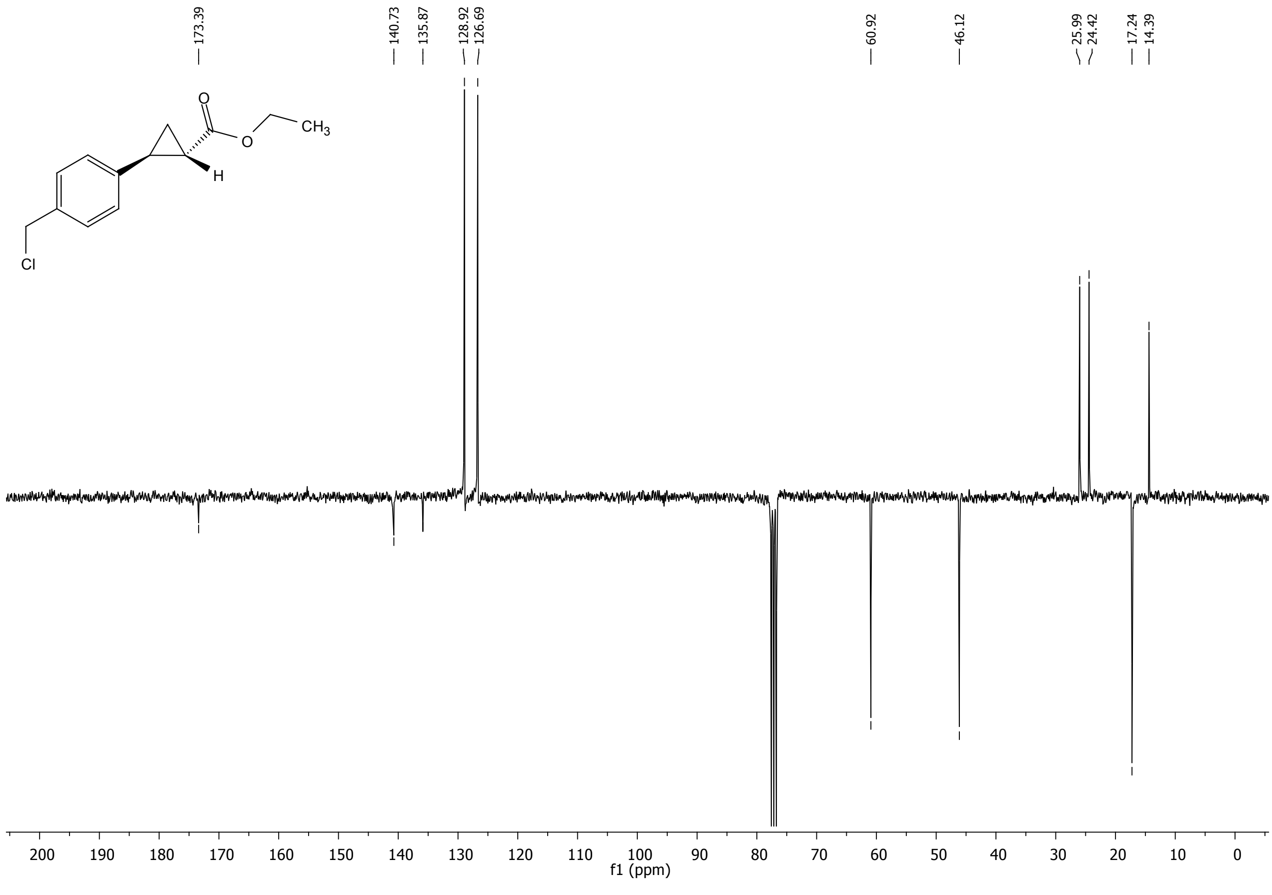
173.39
140.73
135.87
128.92
126.69
60.92
46.12
25.99
24.42
17.24
14.39
O
O
CH3
H
Cl
200
190
180
170
160
150
140
130
120
110
100
90
80
70
60
50
40
30
20
10
0
f1 (ppm)

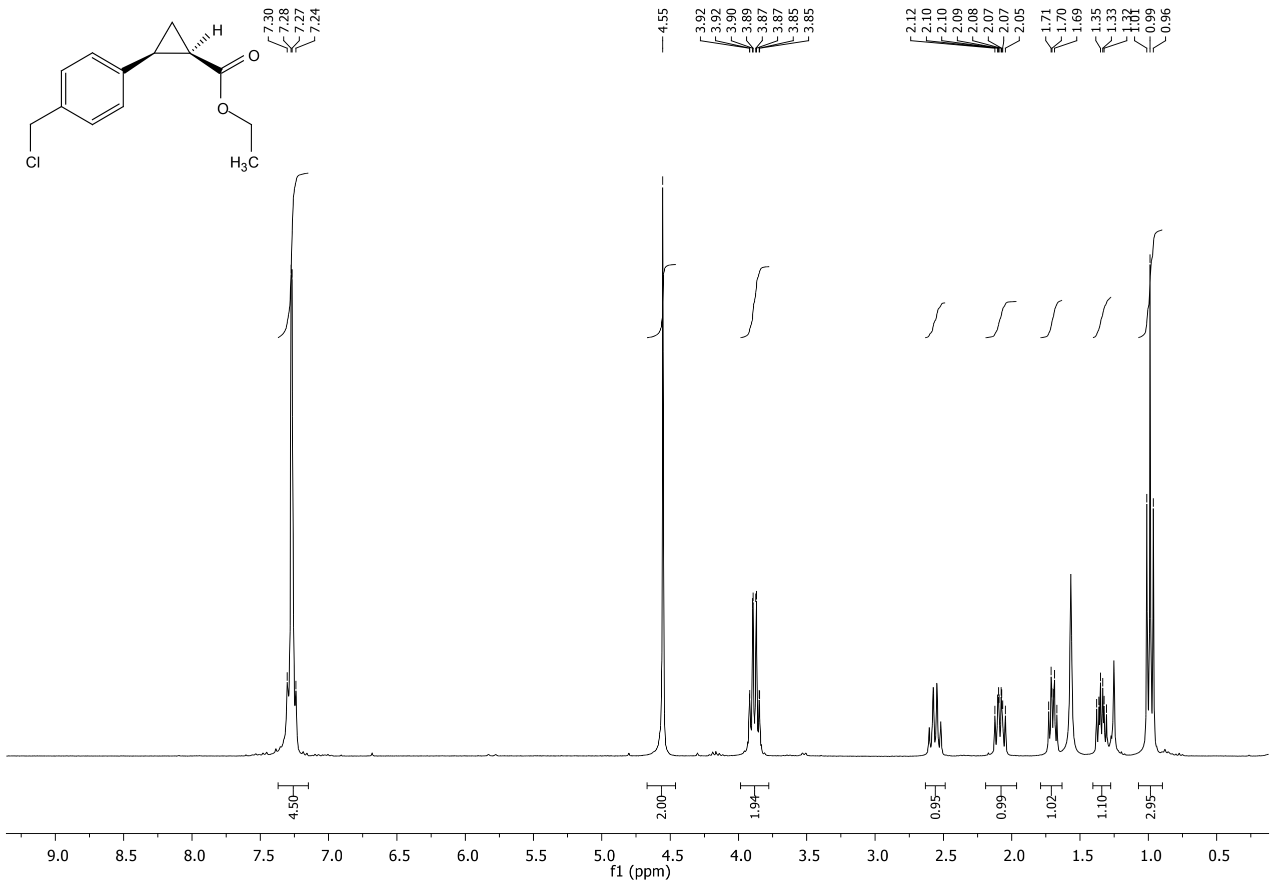
7.30
7.28
7.27
7.24
4.55
3.92
3.92
3.90
3.89
3.87
3.87
3.85
3.85
2.12
2.10
2.10
2.09
2.08
2.07
2.07
2.05
1.71
1.70
1.69
1.35
1.33
1.32
1.01
0.99
0.96
H
O
O
Cl
H_3C
4.50
2.00
1.94
0.95
0.99
1.02
1.10
2.95
9.0 8.5 8.0 7.5 7.0 6.5 6.0 5.5 5.0 4.5 4.0 3.5 3.0 2.5 2.0 1.5 1.0 0.5
f1 (ppm)

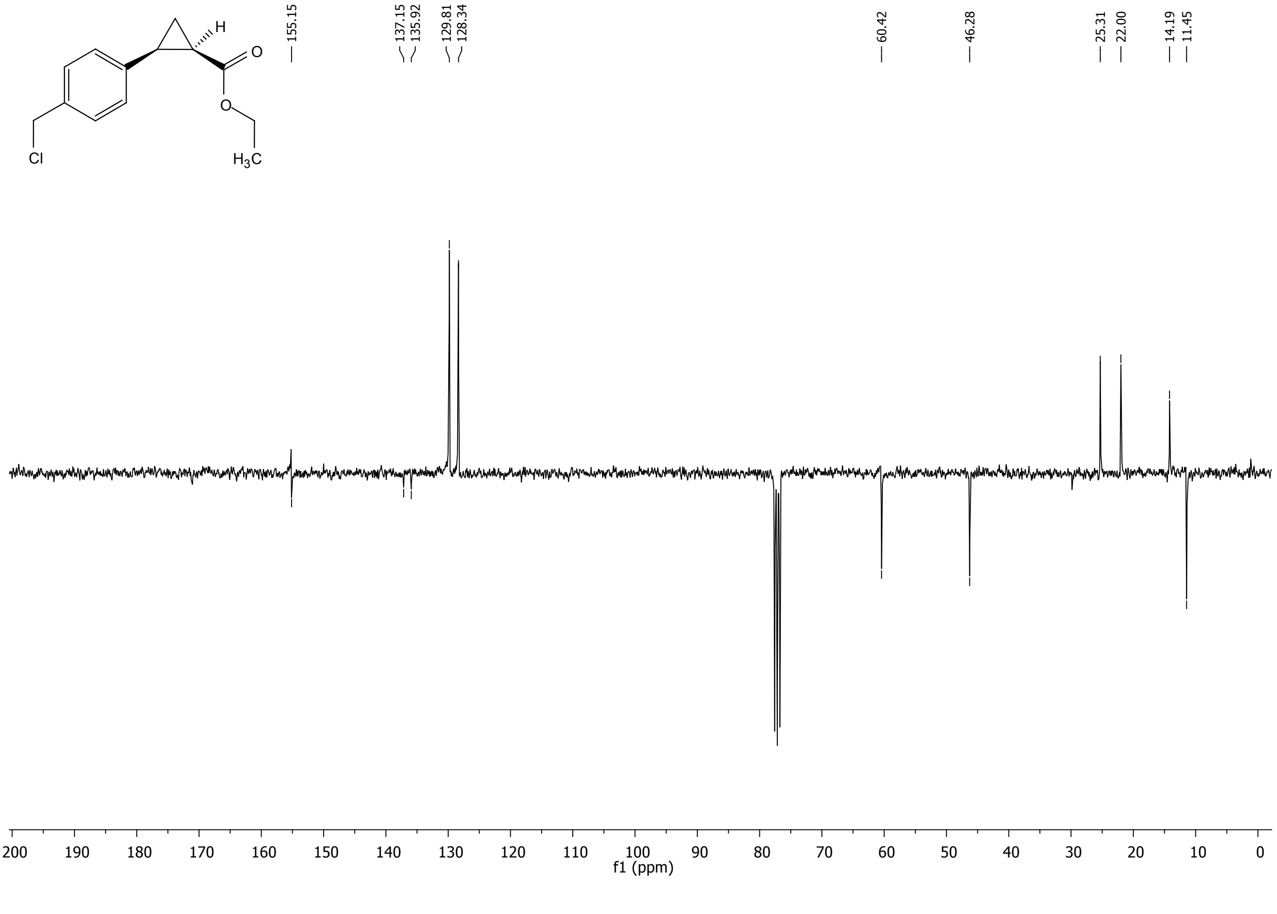
155.15
137.15
135.92
129.81
128.34
60.42
46.28
25.31
22.00
14.19
11.45
H
O
O
Cl
H_3C
200 190 180 170 160 150 140 130 120 110 100 90 80 70 60 50 40 30 20 10 0
f1 (ppm)

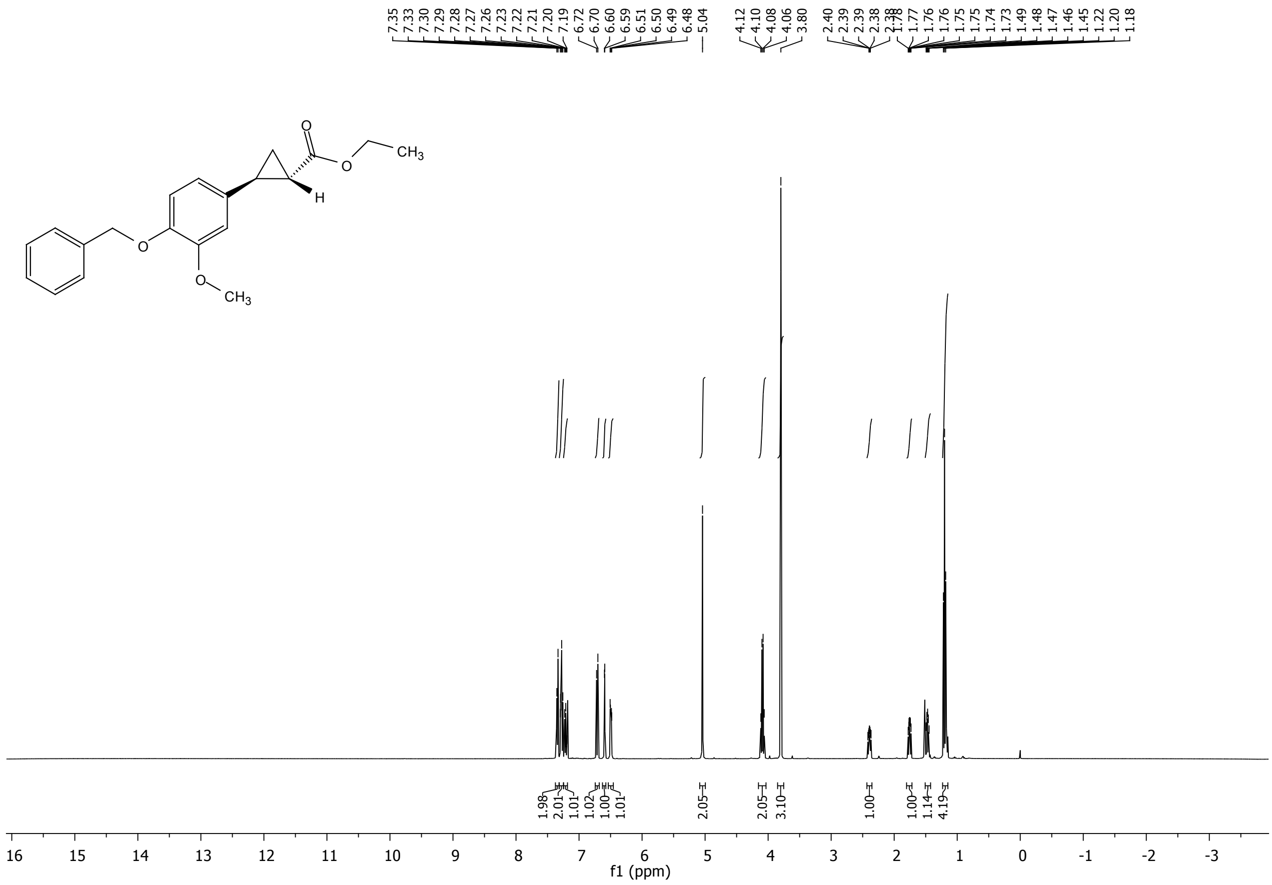
7.35 7.33 7.30 7.29 7.28 7.27 7.26 7.23 7.22 7.21 7.20 7.19 6.72 6.70 6.60 6.59 6.51 6.50 6.49 6.48 5.04 4.12 4.10 4.08 4.06 3.80 2.40 2.39 2.39 2.38 1.78 1.77 1.76 1.76 1.75 1.75 1.74 1.73 1.49 1.48 1.47 1.46 1.45 1.22 1.20 1.18
O
O
CH3
H
O
O
CH3
1.98 2.01 1.01 1.02 1.00 1.01 2.05 2.05 3.10 1.00 1.00 1.14 4.19
16 15 14 13 12 11 10 9 8 7 6 5 4 3 2 1 0 -1 -2 -3
f1 (ppm)

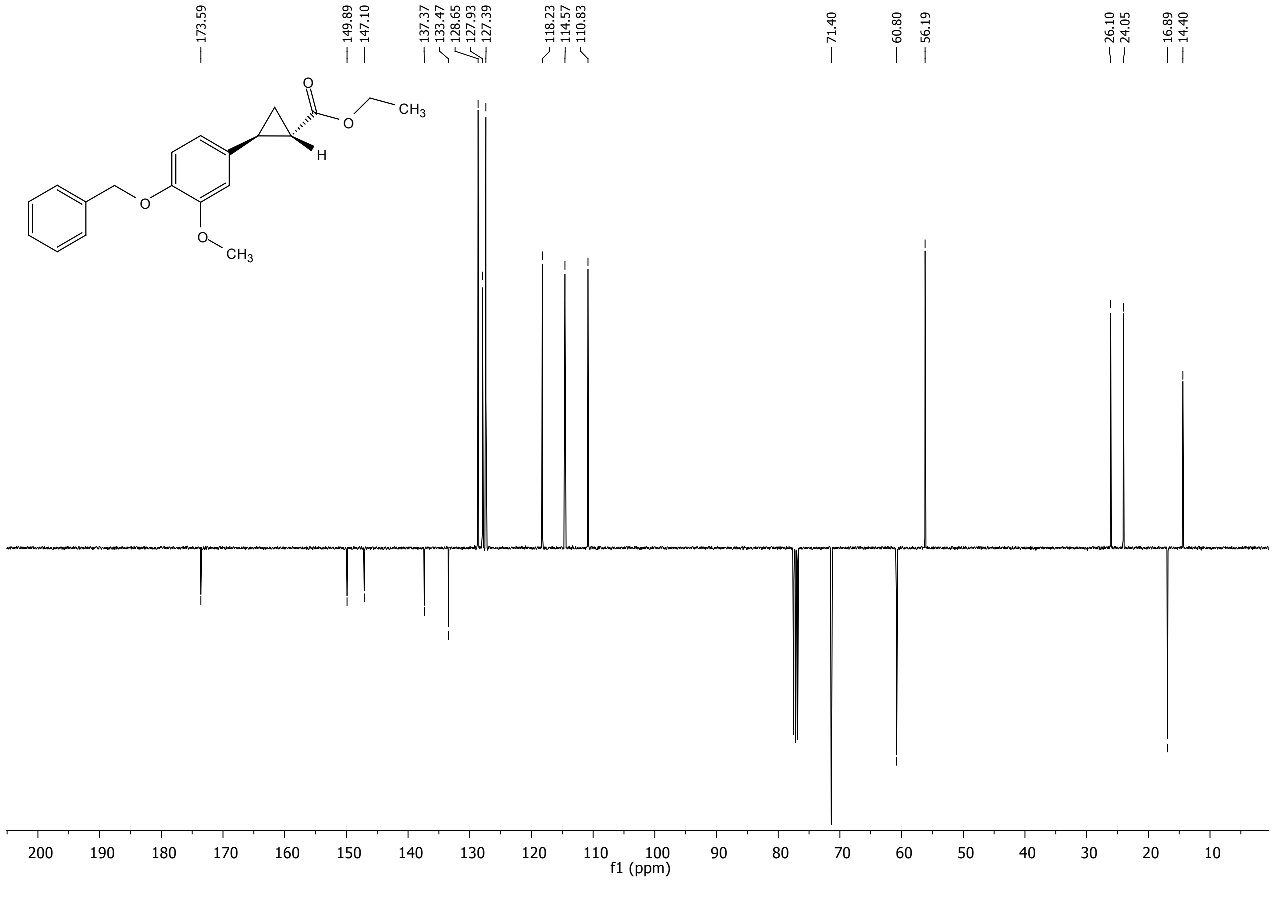
173.59 149.89 147.10 137.37 133.47 128.65 127.93 127.39 118.23 114.57 110.83 71.40 60.80 56.19 26.10 24.05 16.89 14.40
O
O
CH3
H
O
O
CH3
200 190 180 170 160 150 140 130 120 110 100 90 80 70 60 50 40 30 20 10
f1 (ppm)

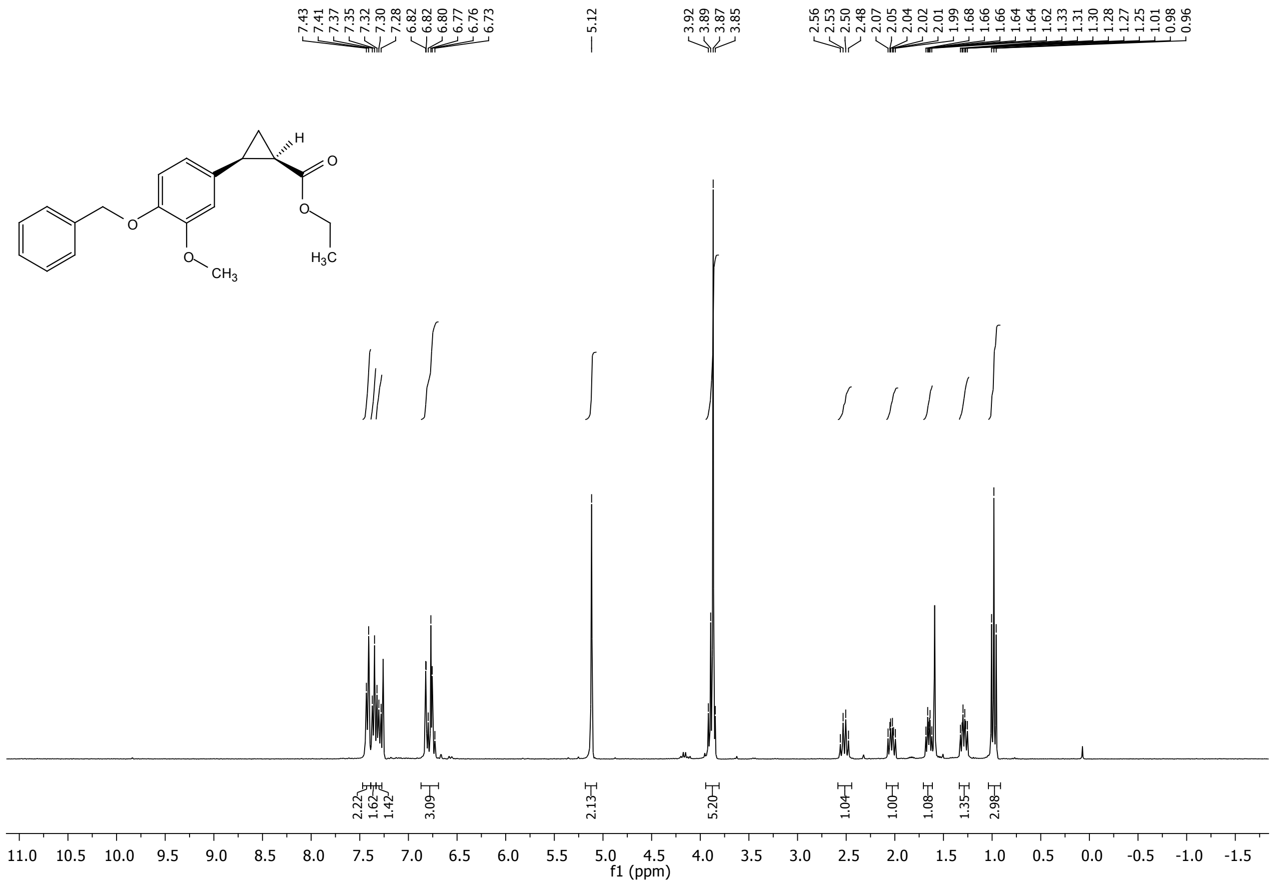
7.43 7.41 7.37 7.35 7.32 7.30 7.28 6.82 6.80 6.77 6.76 6.73
5.12
3.92 3.89 3.87 3.85
2.56 2.53 2.50 2.48 2.07 2.05 2.04 2.02 2.01 1.99 1.68 1.66 1.66 1.64 1.62 1.33 1.31 1.30 1.28 1.27 1.25 1.01 0.98 0.96
H
O
O
O
O
CH3
H3C
2.22 1.62 1.42 3.09 2.13 5.20 1.04 1.00 1.08 1.35 2.98
11.0 10.5 10.0 9.5 9.0 8.5 8.0 7.5 7.0 6.5 6.0 5.5 5.0 4.5 4.0 3.5 3.0 2.5 2.0 1.5 1.0 0.5 0.0 -0.5 -1.0 -1.5
f1 (ppm)

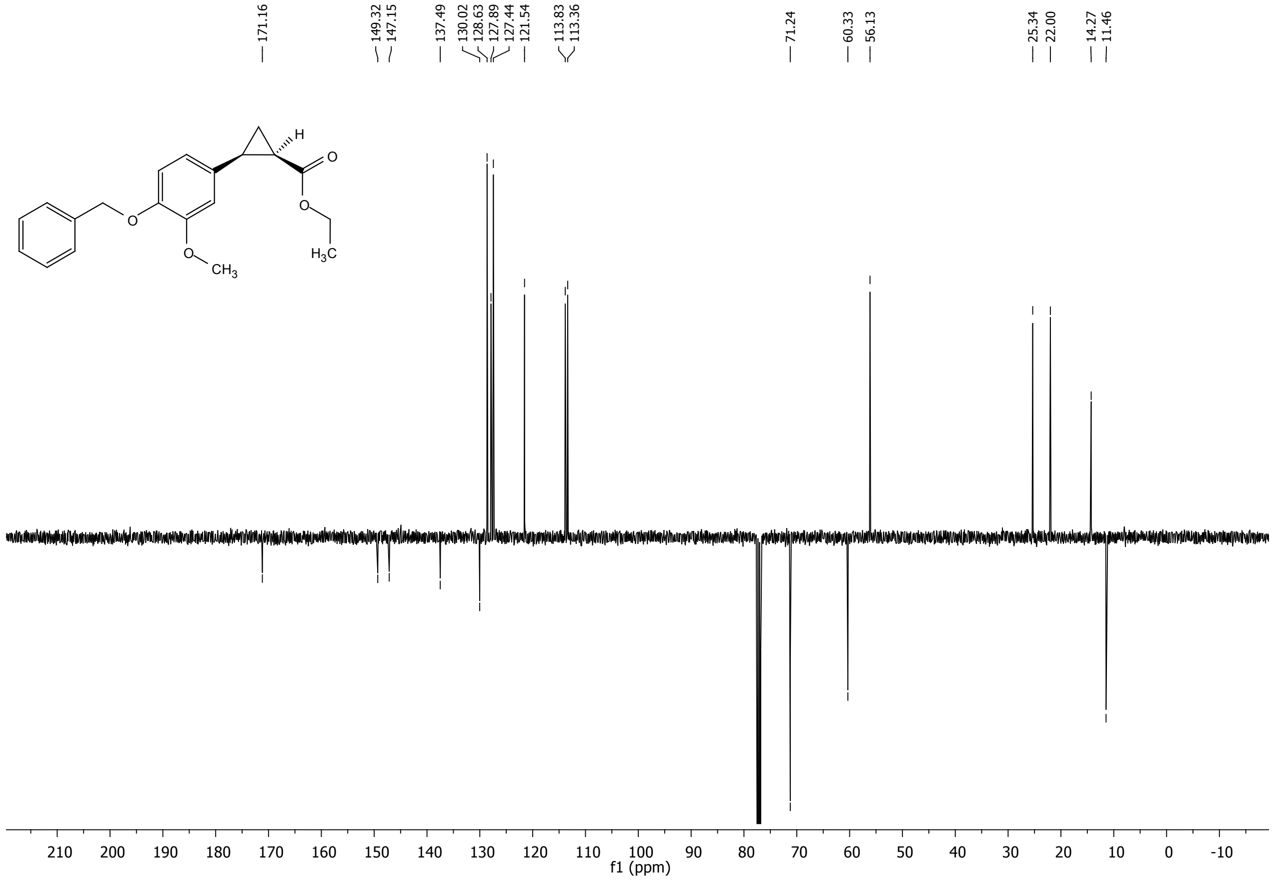
171.16
149.32 147.15
137.49 130.02 128.63 127.89 127.44 121.54
113.83 113.36
71.24
60.33 56.13
25.34 22.00
14.27 11.46
H
O
O
O
O
CH3
H3C
210 200 190 180 170 160 150 140 130 120 110 100 90 80 70 60 50 40 30 20 10 0 -10
f1 (ppm)

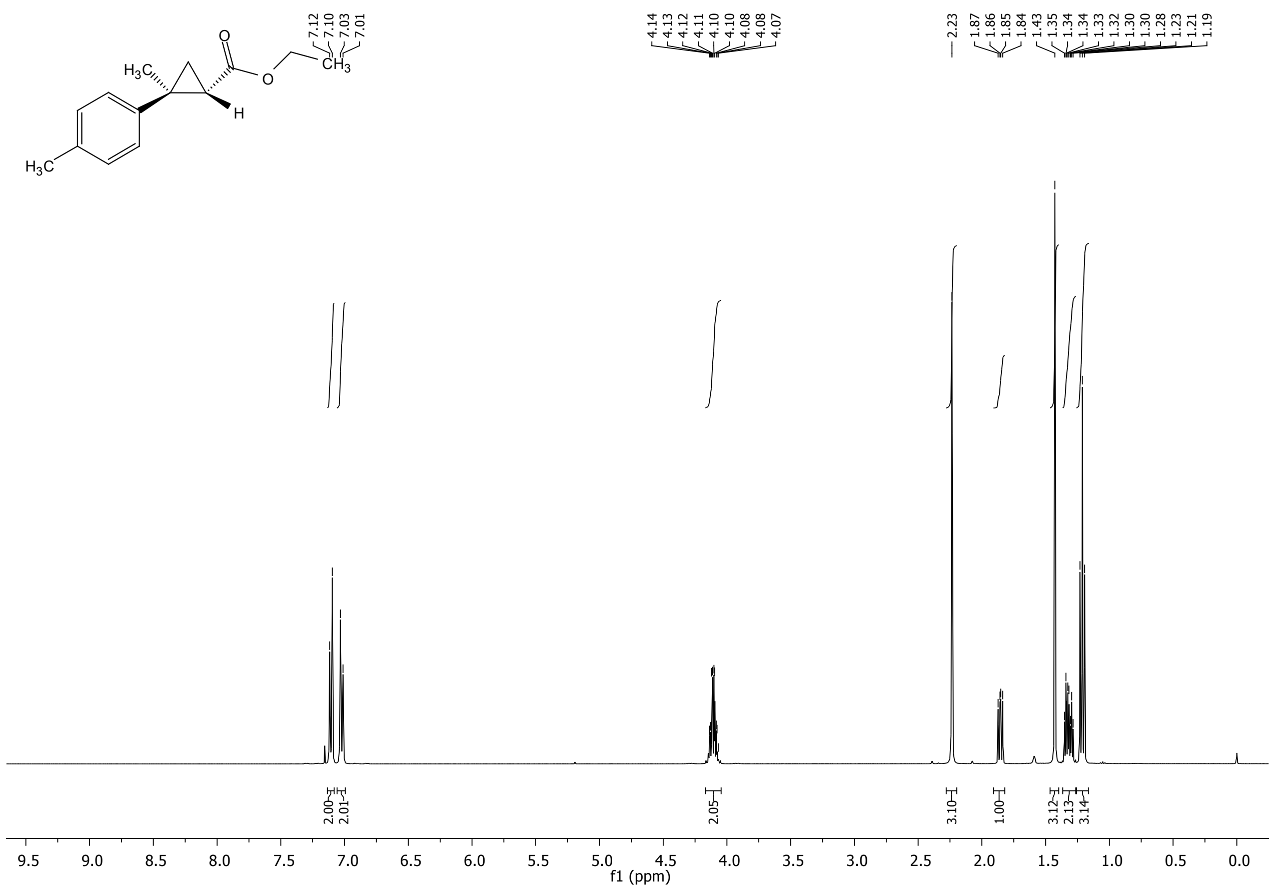
H3C
O
O
CH3
H
H3C
7.12
7.10
7.03
7.01
4.14
4.13
4.12
4.11
4.10
4.10
4.08
4.08
4.07
2.23
1.87
1.86
1.85
1.84
1.43
1.35
1.34
1.34
1.33
1.32
1.30
1.30
1.28
1.23
1.21
1.19
2.00
2.01
2.05
3.10
1.00
3.12
2.13
3.14
9.5 9.0 8.5 8.0 7.5 7.0 6.5 6.0 5.5 5.0 4.5 4.0 3.5 3.0 2.5 2.0 1.5 1.0 0.5 0.0
f1 (ppm)

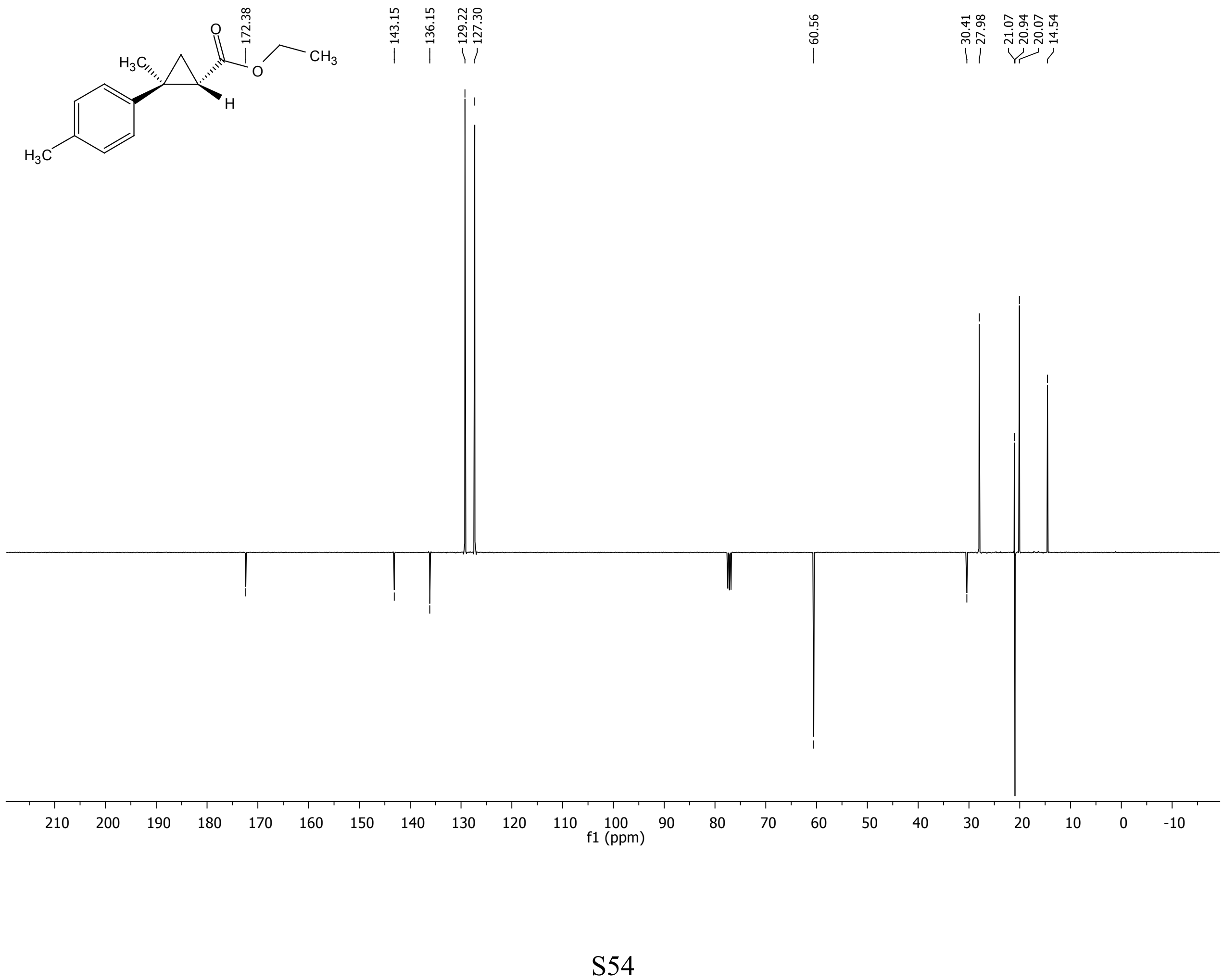
H3C
O
O
CH3
H
H3C
172.38
143.15
136.15
129.22
127.30
60.56
30.41
27.98
21.07
20.94
20.07
14.54
210 200 190 180 170 160 150 140 130 120 110 100 90 80 70 60 50 40 30 20 10 0 -10
f1 (ppm)

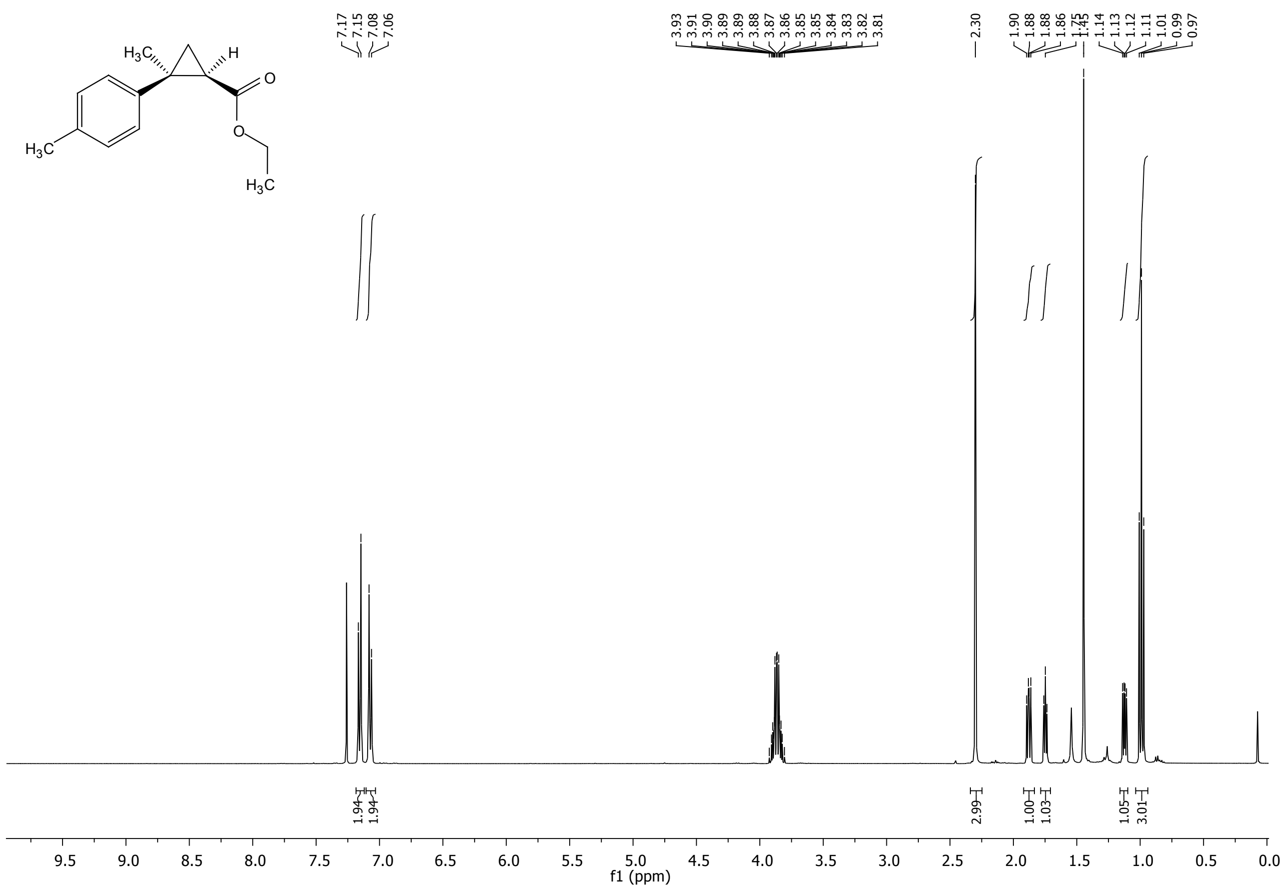
H3C
H
O
O
H3C
H3C
7.17
7.15
7.08
7.06
3.93
3.91
3.90
3.89
3.89
3.88
3.87
3.86
3.85
3.85
3.84
3.83
3.82
3.81
2.30
1.90
1.88
1.88
1.86
1.75
1.45
1.14
1.13
1.12
1.11
1.01
0.99
0.97
1.94
1.94
2.99
1.00
1.03
1.05
3.01
9.5 9.0 8.5 8.0 7.5 7.0 6.5 6.0 5.5 5.0 4.5 4.0 3.5 3.0 2.5 2.0 1.5 1.0 0.5 0.0
f1 (ppm)

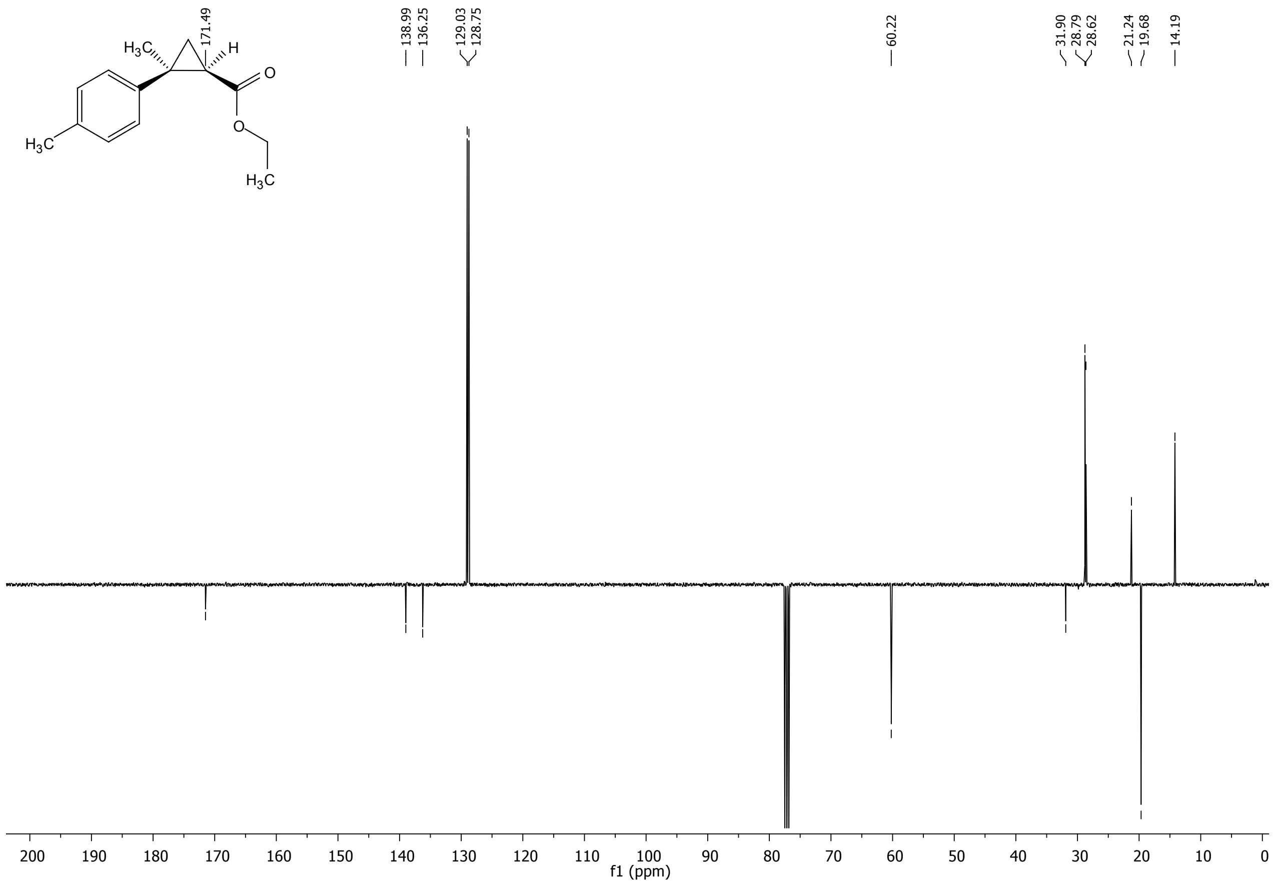
H3C
H
O
O
H3C
H3C
171.49
138.99
136.25
129.03
128.75
60.22
31.90
28.79
28.62
21.24
19.68
14.19
200 190 180 170 160 150 140 130 120 110 100 90 80 70 60 50 40 30 20 10 0
f1 (ppm)

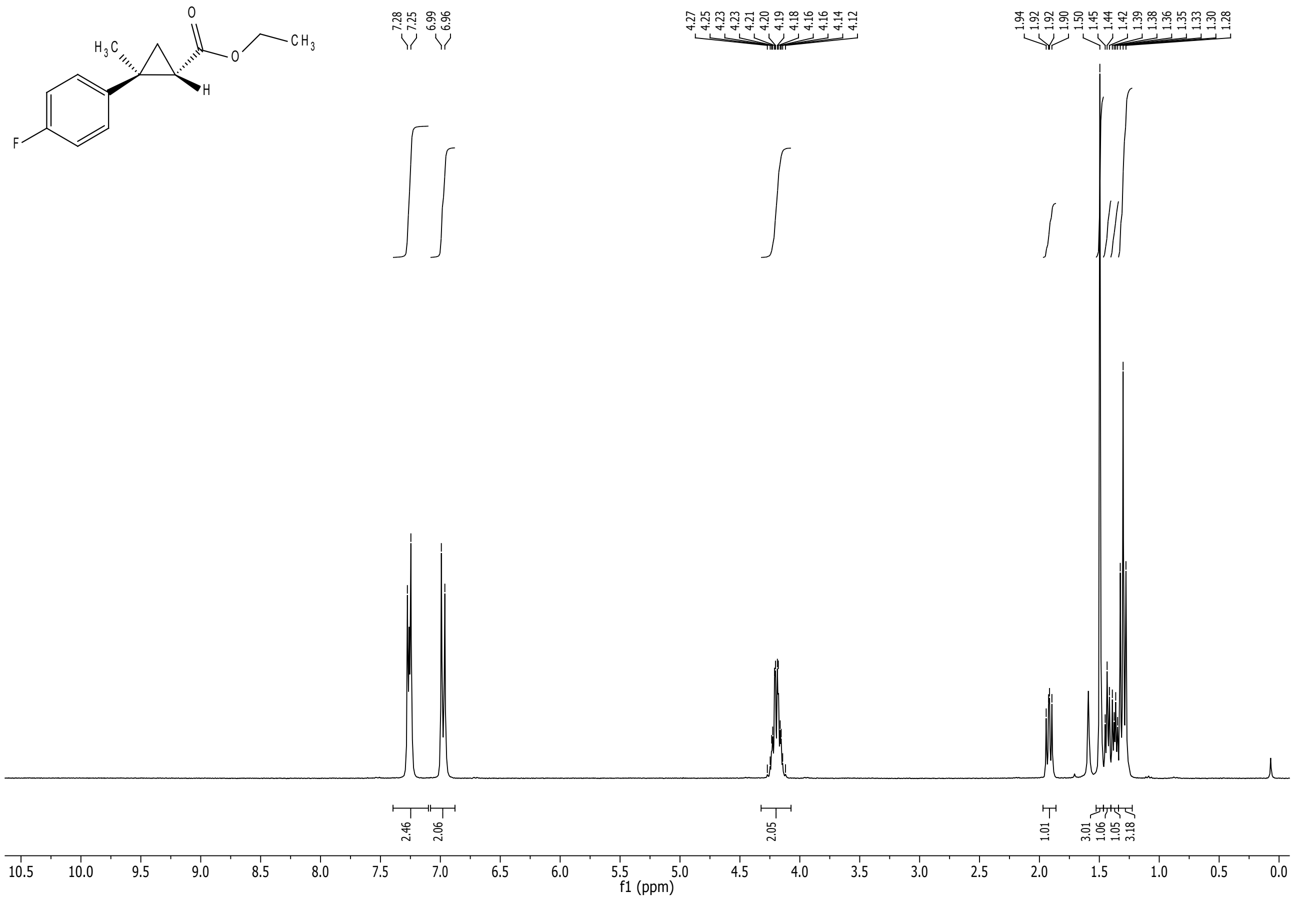

O
H_3C
O
CH_3
H
F
7.28
7.25
6.99
6.96
4.27
4.25
4.23
4.23
4.21
4.20
4.19
4.18
4.16
4.16
4.14
4.12
1.94
1.92
1.92
1.90
1.50
1.45
1.44
1.42
1.39
1.38
1.36
1.35
1.33
1.30
1.28
2.46
2.06
2.05
1.01
3.01
1.06
1.05
3.18
10.5 10.0 9.5 9.0 8.5 8.0 7.5 7.0 6.5 6.0 5.5 5.0 4.5 4.0 3.5 3.0 2.5 2.0 1.5 1.0 0.5 0.0
f1 (ppm)

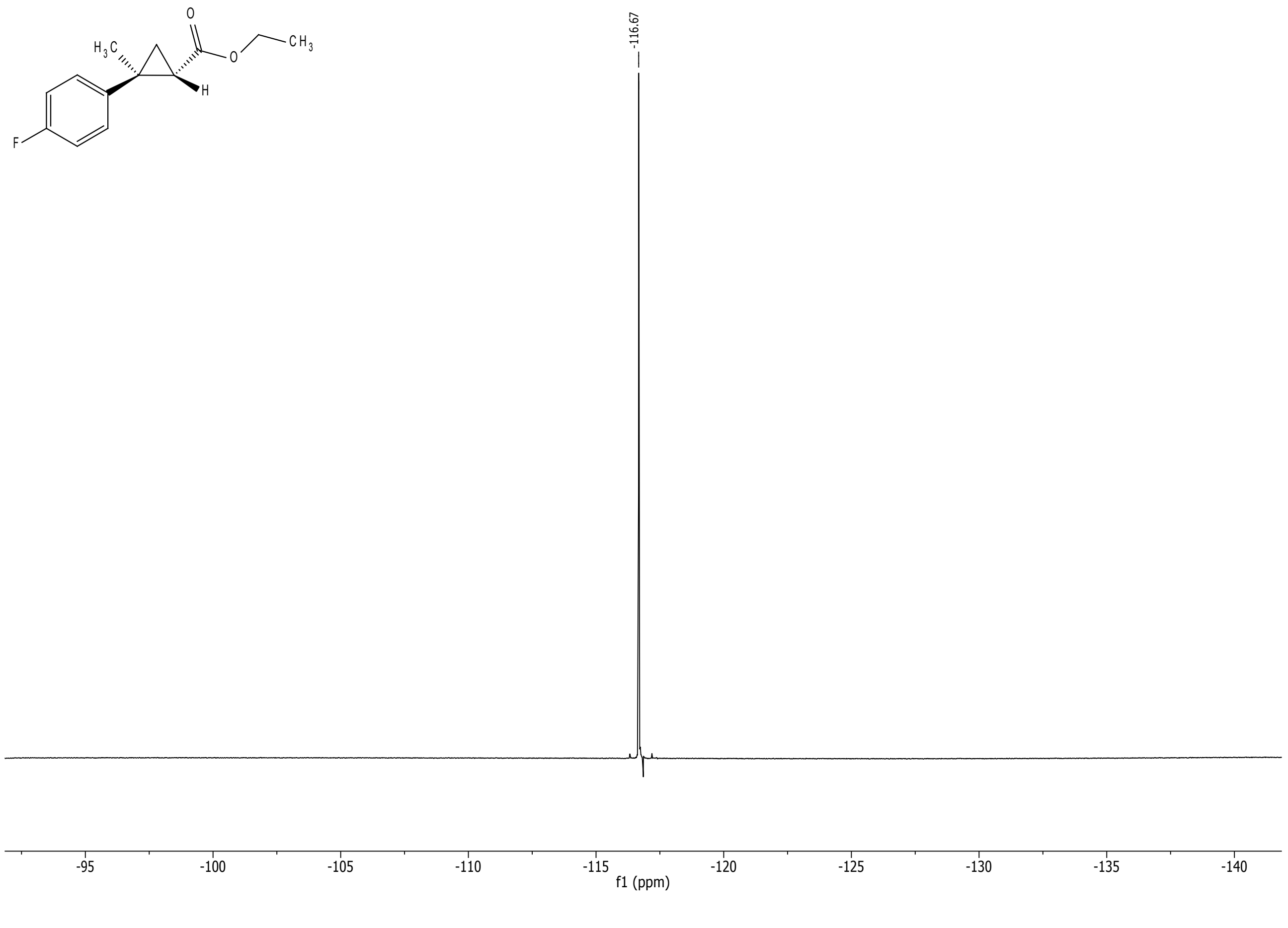

O
H_3C
O
CH_3
H
F
-116.67
-95 -100 -105 -110 -115 -120 -125 -130 -135 -140
f1 (ppm)

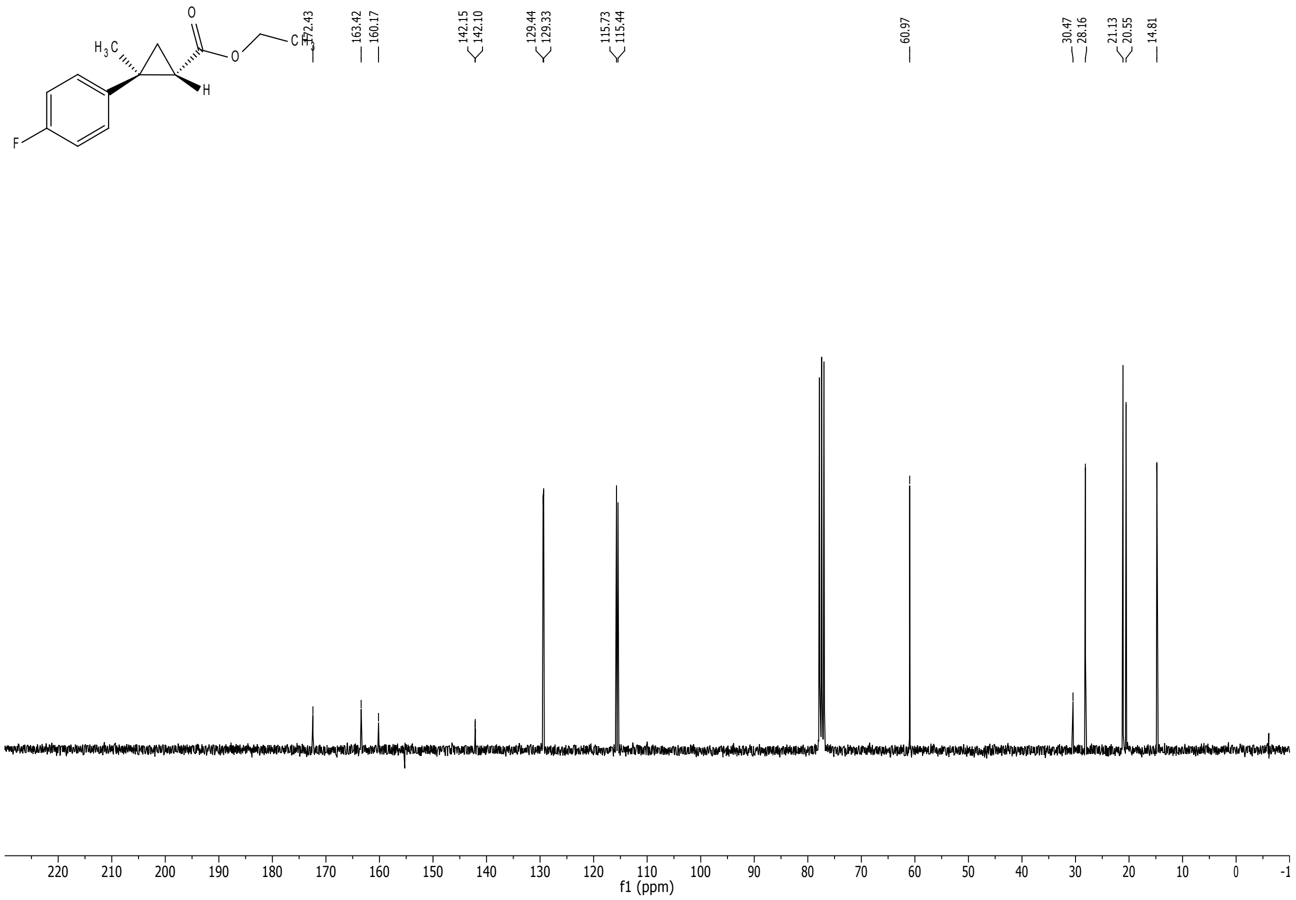
H3C
O
O
CH3
H
F
172.43
163.42
160.17
142.15
142.10
129.44
129.33
115.73
115.44
60.97
30.47
28.16
21.13
20.55
14.81
220 210 200 190 180 170 160 150 140 130 120 110 100 90 80 70 60 50 40 30 20 10 0 -1
f1 (ppm)

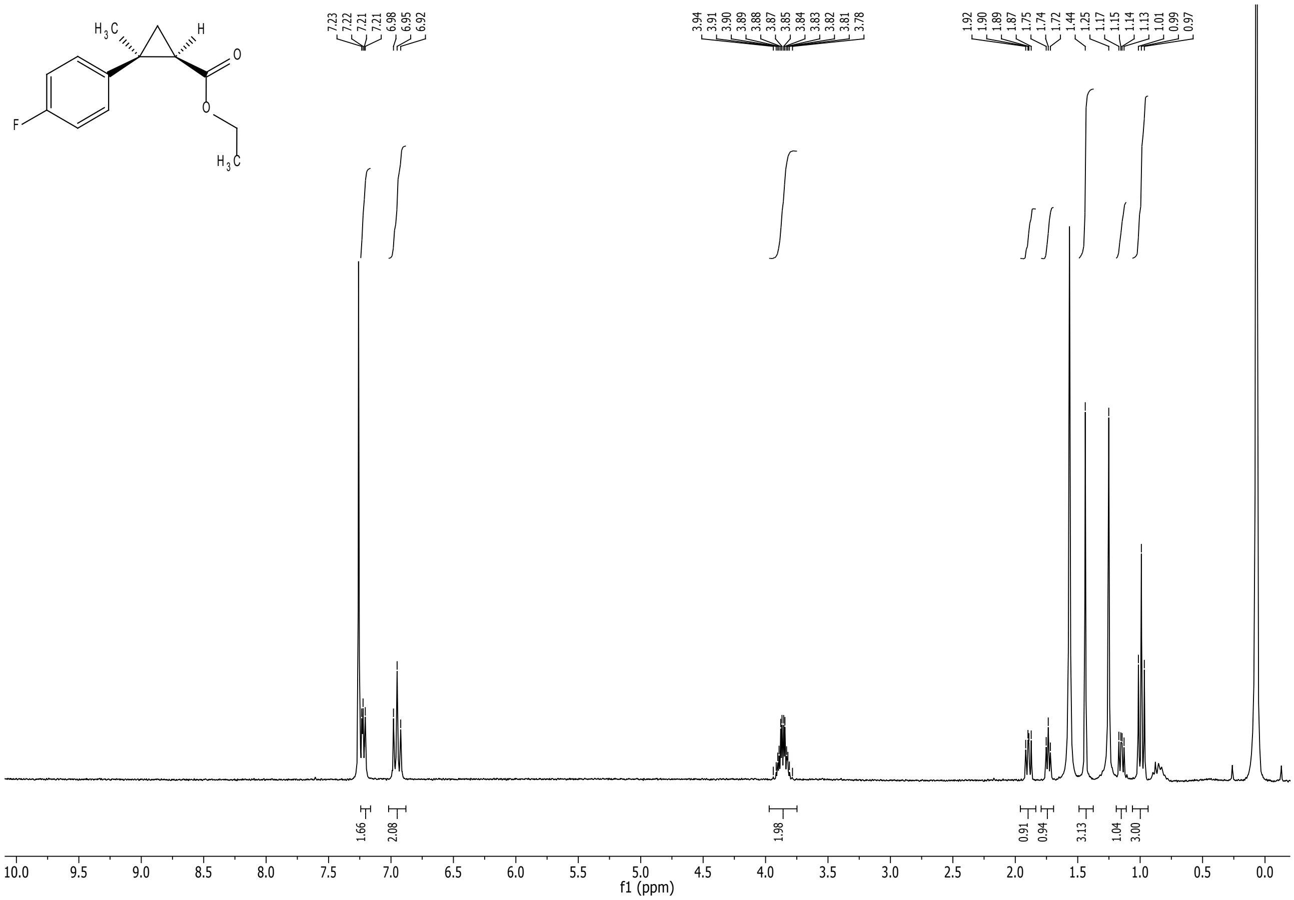
H3C
H
O
O
F
H3C
7.23
7.22
7.21
7.21
6.98
6.95
6.92
3.94
3.91
3.90
3.89
3.88
3.87
3.85
3.84
3.83
3.82
3.81
3.78
1.92
1.90
1.89
1.87
1.75
1.74
1.72
1.44
1.25
1.17
1.15
1.14
1.13
1.01
0.99
0.97
1.66
2.08
1.98
0.91
0.94
3.13
1.04
3.00
f1 (ppm)

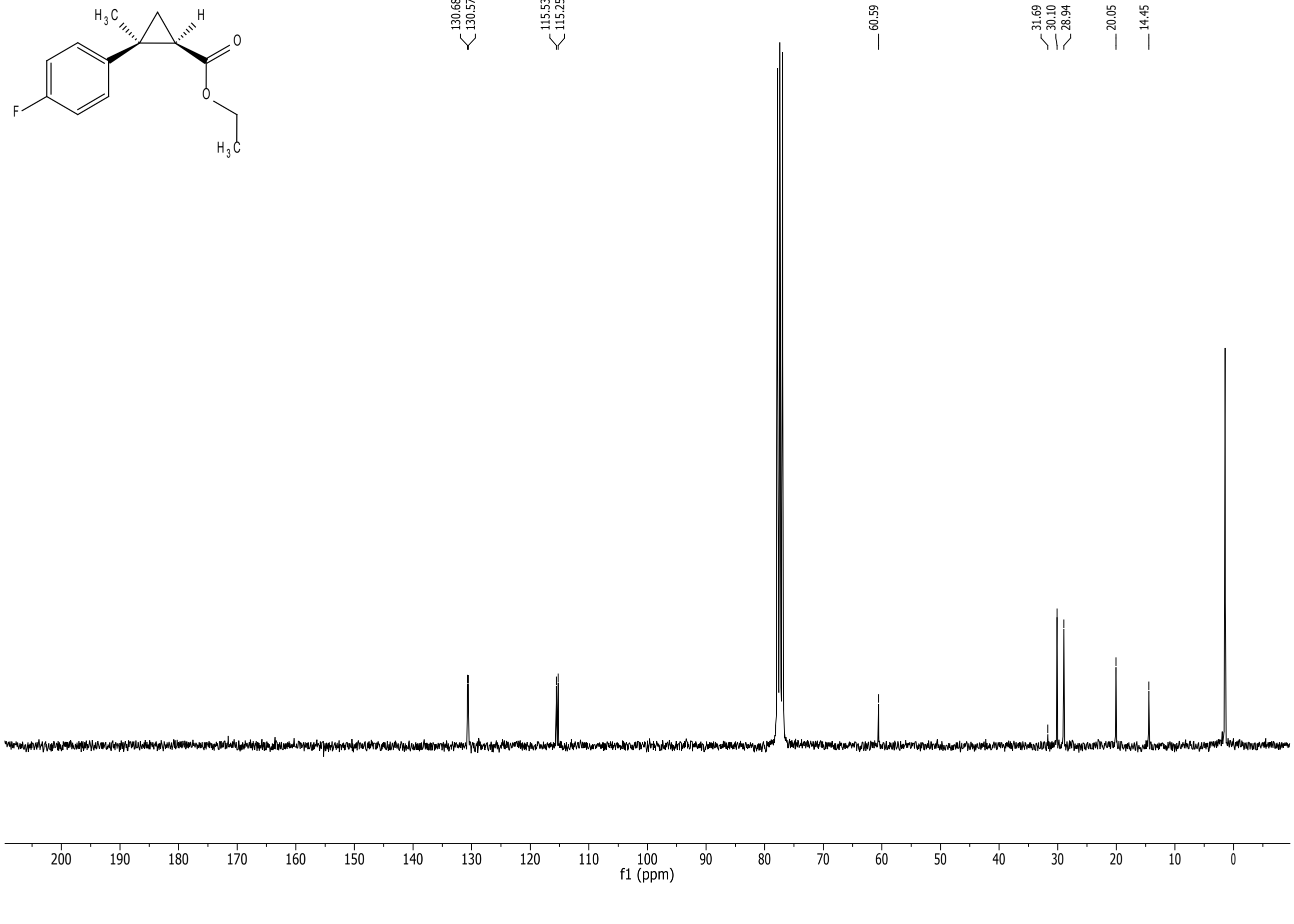
H3C
H
O
O
F
H3C
130.68
130.57
115.53
115.25
60.59
31.69
30.10
28.94
20.05
14.45
f1 (ppm)

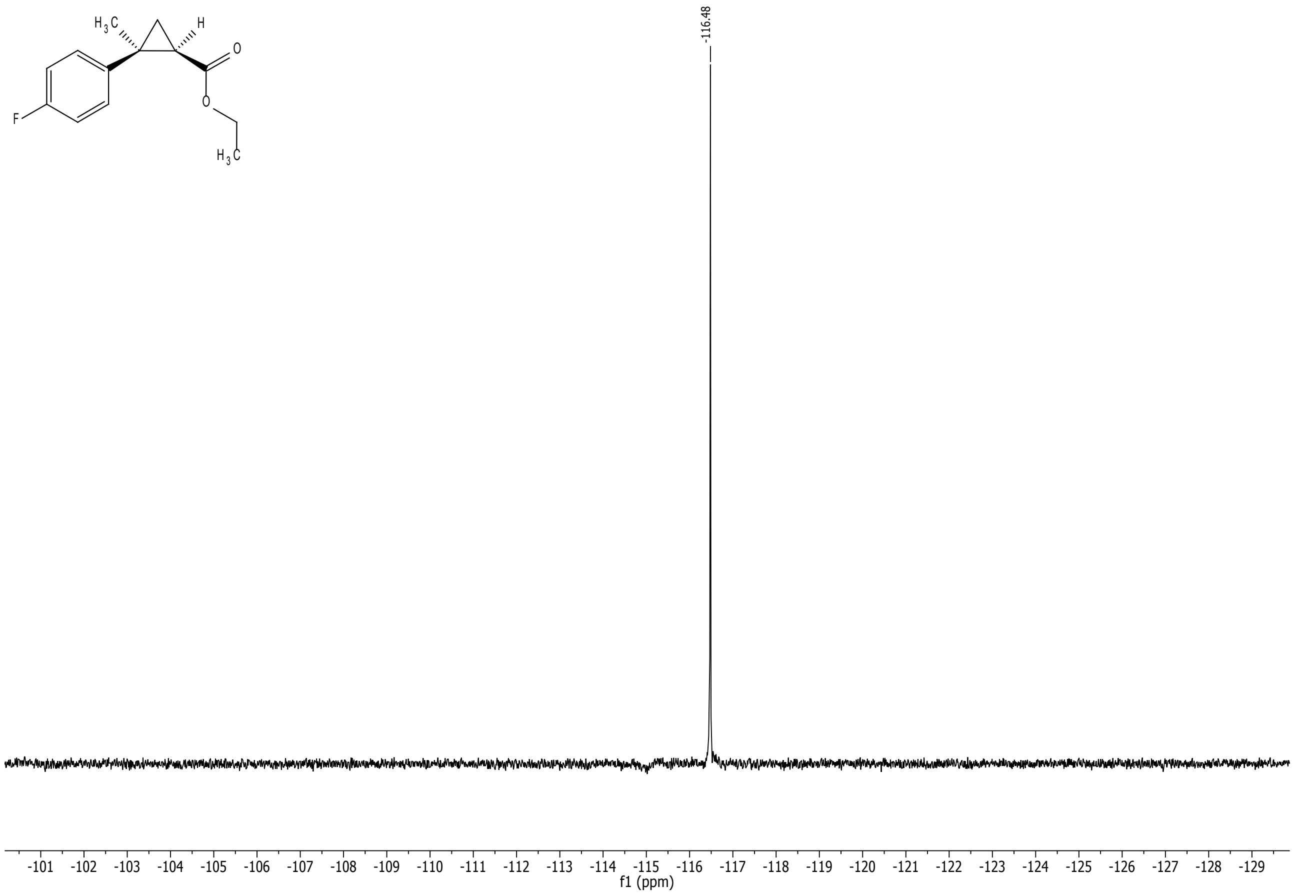

-116.48
f1 (ppm)

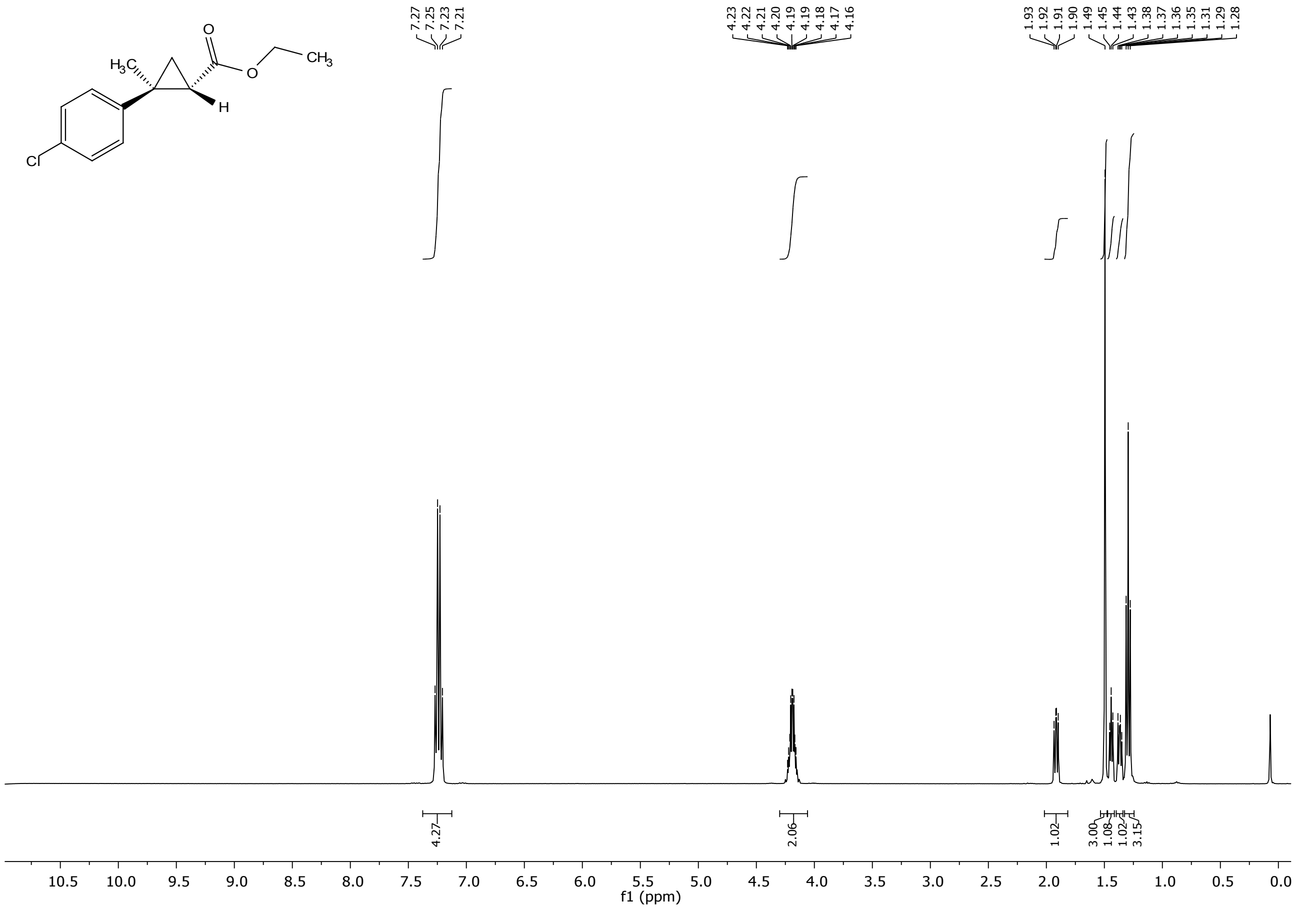
O
O
CH3
H3C
H
Cl
7.27
7.25
7.23
7.21
4.23
4.22
4.21
4.20
4.19
4.19
4.18
4.17
4.16
1.93
1.92
1.91
1.90
1.49
1.45
1.44
1.43
1.38
1.37
1.36
1.35
1.31
1.29
1.28
4.27
2.06
1.02
3.00
1.08
1.02
3.15
10.5 10.0 9.5 9.0 8.5 8.0 7.5 7.0 6.5 6.0 5.5 5.0 4.5 4.0 3.5 3.0 2.5 2.0 1.5 1.0 0.5 0.0
f1 (ppm)

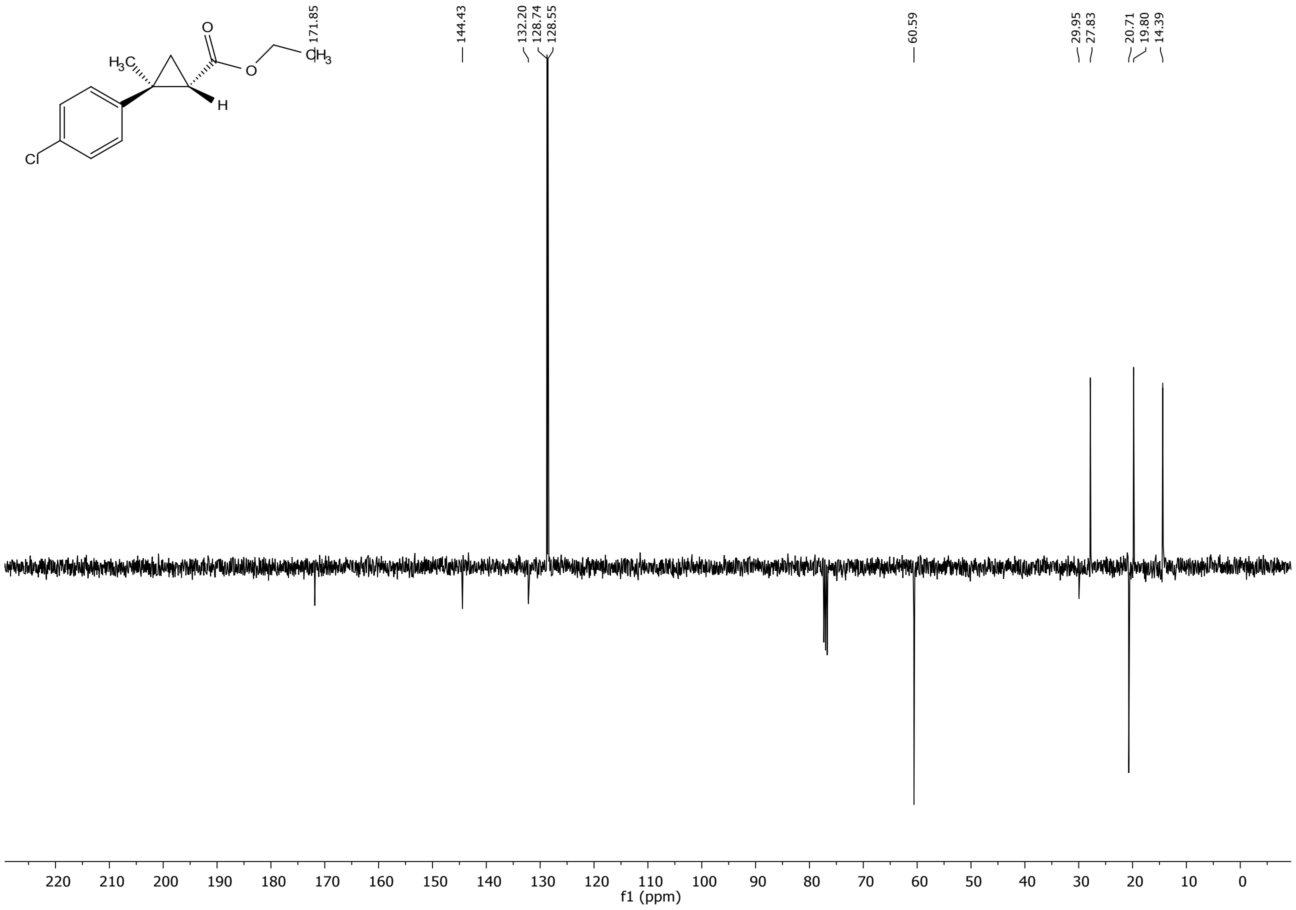

171.85
144.43
132.20
128.74
128.55
60.59
29.95
27.83
20.71
19.80
14.39
f1 (ppm)

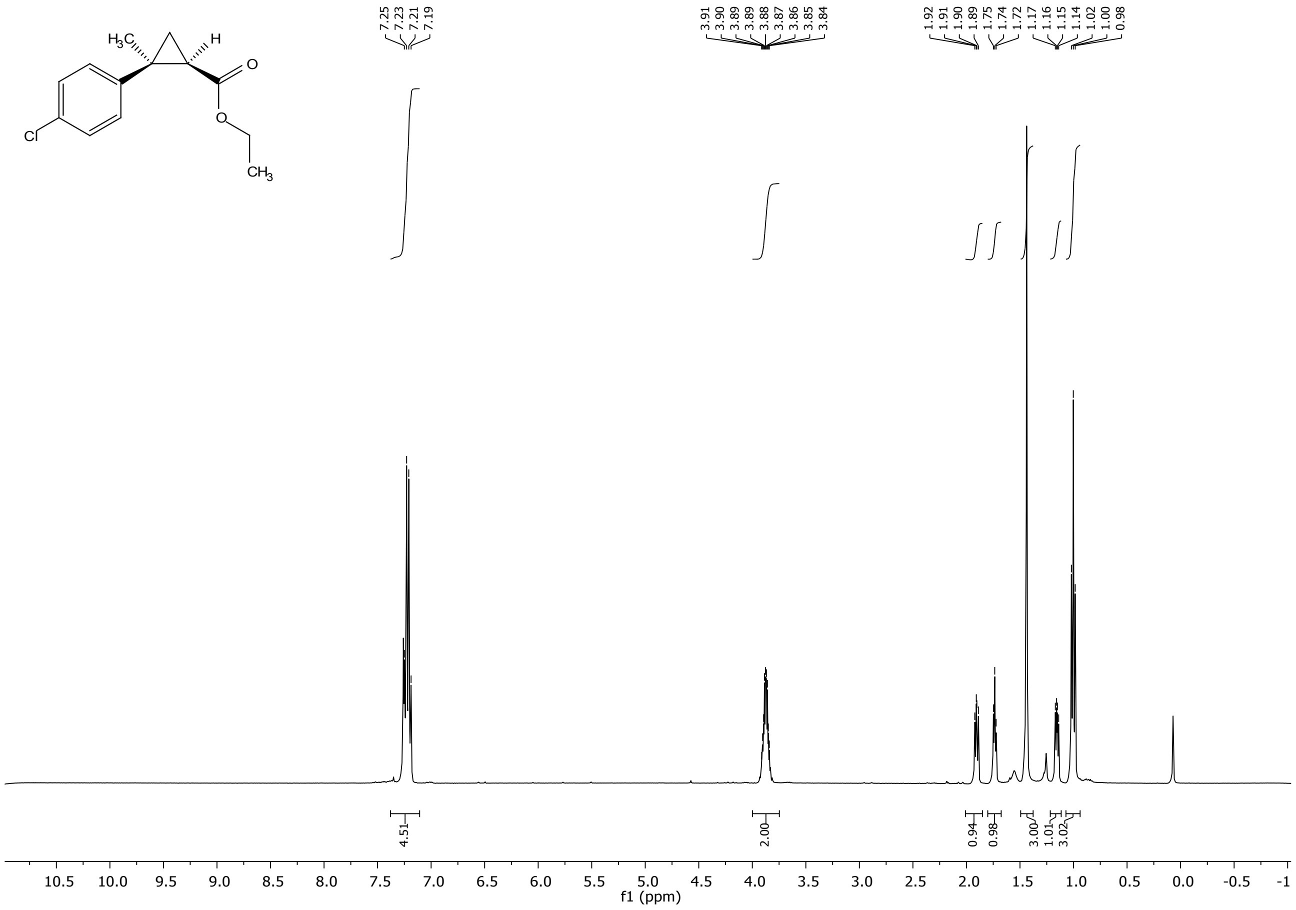
H3C
H
O
O
Cl
CH3
7.25 7.23 7.21 7.19
3.91 3.90 3.89 3.89 3.88 3.87 3.86 3.85 3.84
1.92 1.91 1.90 1.89 1.75 1.74 1.72 1.17 1.16 1.15 1.14 1.02 1.00 0.98
4.51
2.00
0.94
0.98
3.00
1.01
3.02
10.5 10.0 9.5 9.0 8.5 8.0 7.5 7.0 6.5 6.0 5.5 5.0 4.5 4.0 3.5 3.0 2.5 2.0 1.5 1.0 0.5 0.0 -0.5 -1
f1 (ppm)

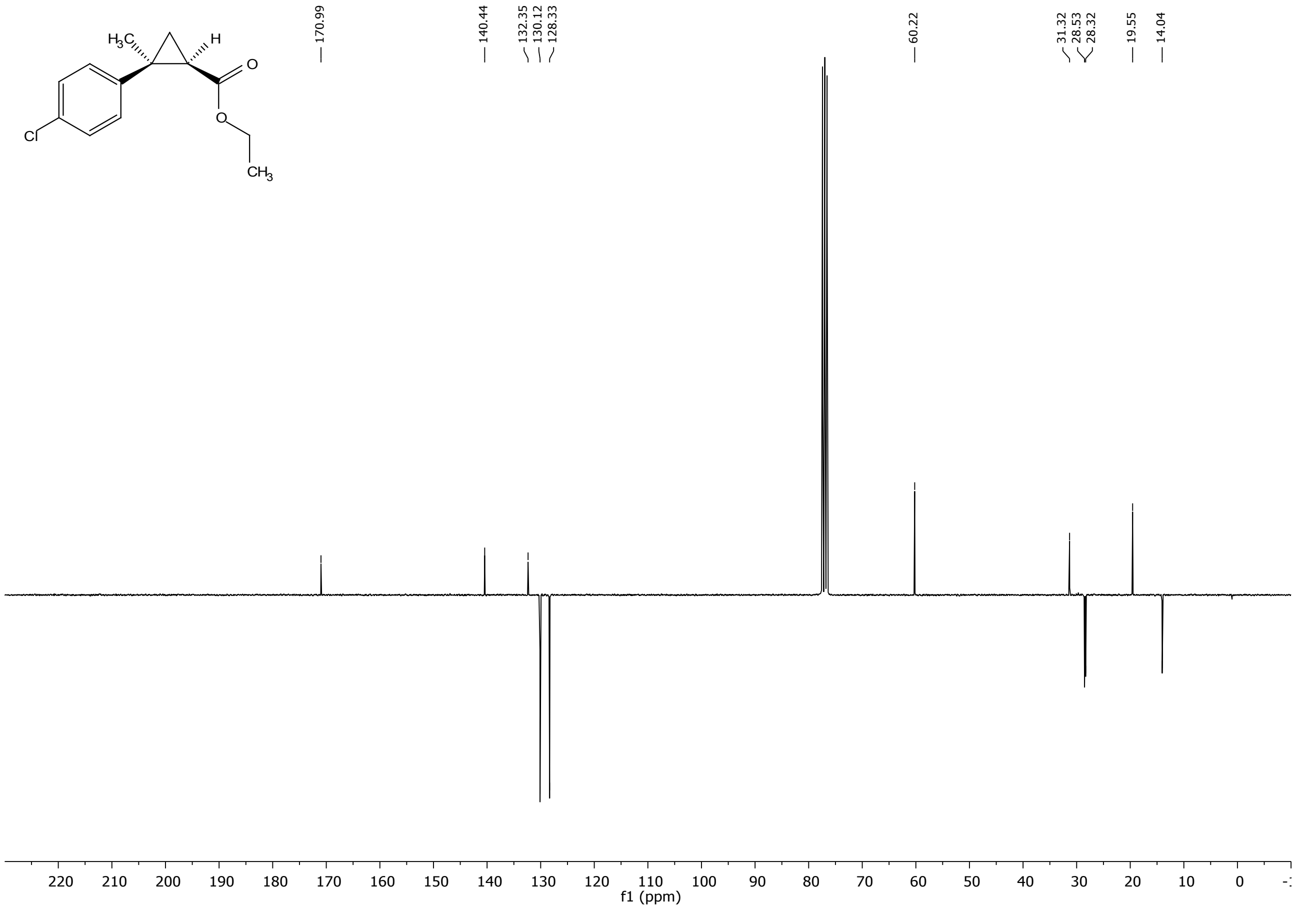

H3C
H
O
O
Cl
CH3
170.99
140.44
132.35
130.12
128.33
60.22
31.32
28.53
28.32
19.55
14.04
220 210 200 190 180 170 160 150 140 130 120 110 100 90 80 70 60 50 40 30 20 10 0
f1 (ppm)

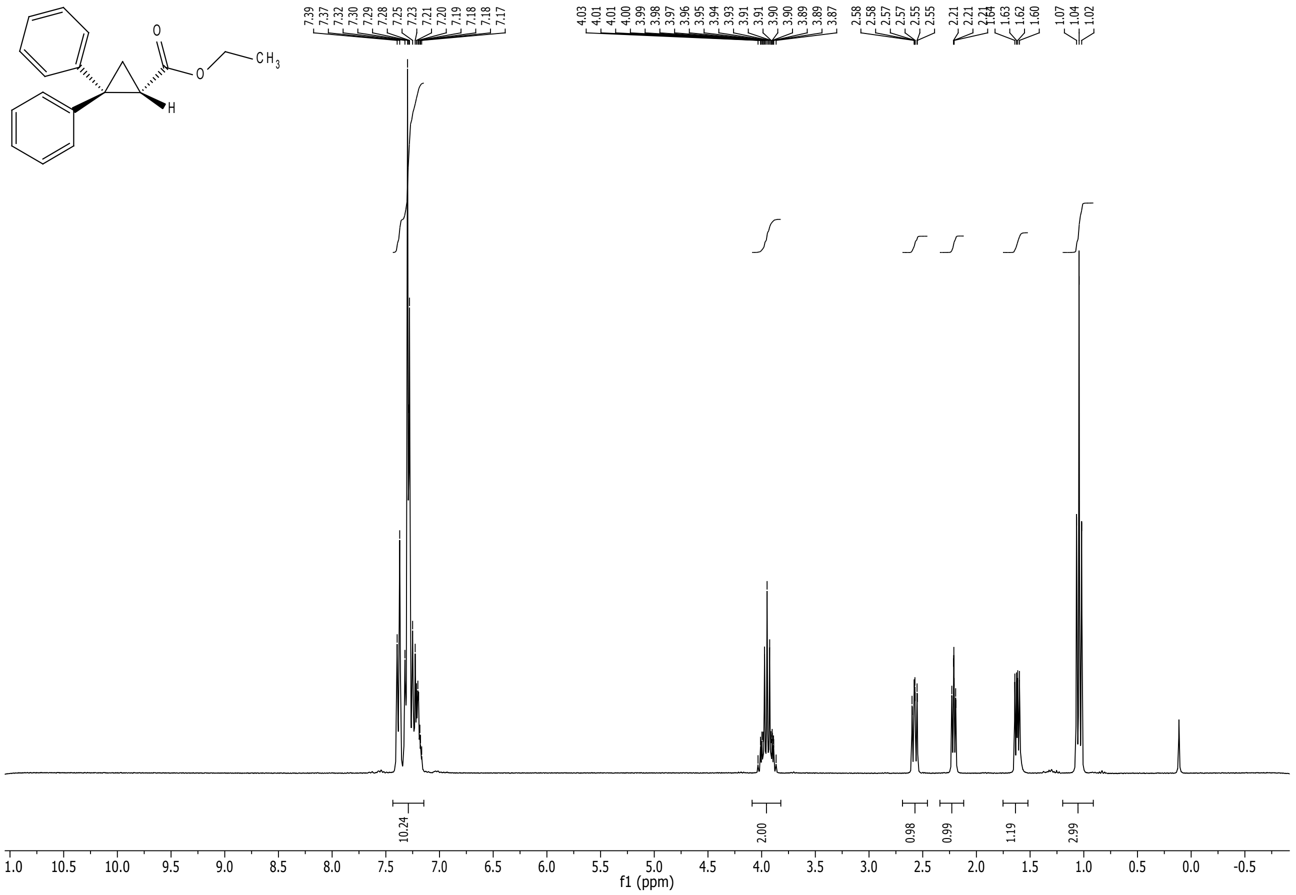
O
O
CH3
H
7.39
7.37
7.32
7.30
7.29
7.28
7.25
7.23
7.21
7.20
7.19
7.18
7.18
7.17
4.03
4.01
4.01
4.00
3.99
3.98
3.97
3.96
3.95
3.94
3.93
3.91
3.91
3.90
3.90
3.89
3.89
3.87
2.58
2.58
2.57
2.57
2.55
2.55
2.21
2.21
2.21
1.64
1.63
1.62
1.60
1.07
1.04
1.02
10.24
2.00
0.98
0.99
1.19
2.99
f1 (ppm)

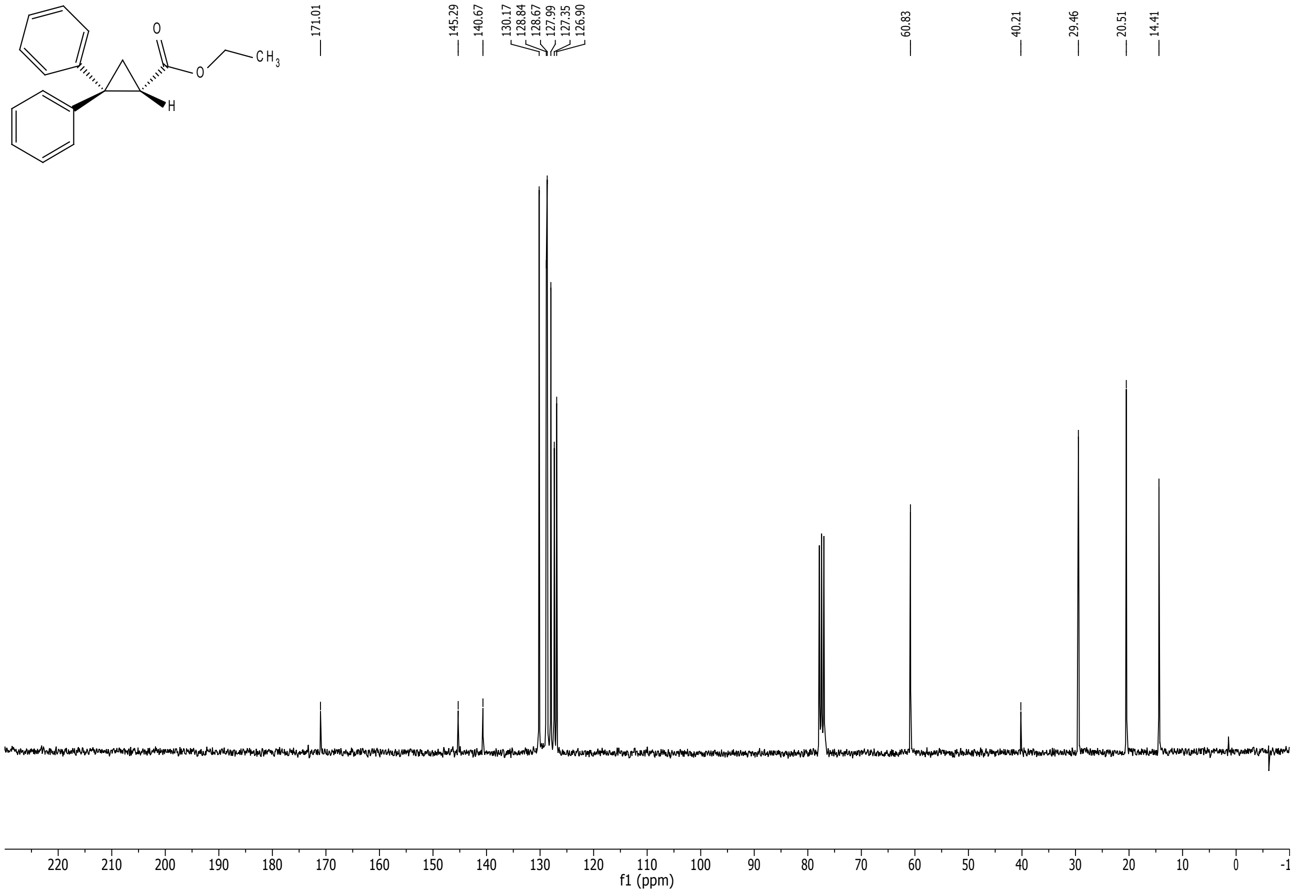
O
O
CH3
H
171.01
145.29
140.67
130.17
128.84
128.67
127.99
127.35
126.90
60.83
40.21
29.46
20.51
14.41
f1 (ppm)

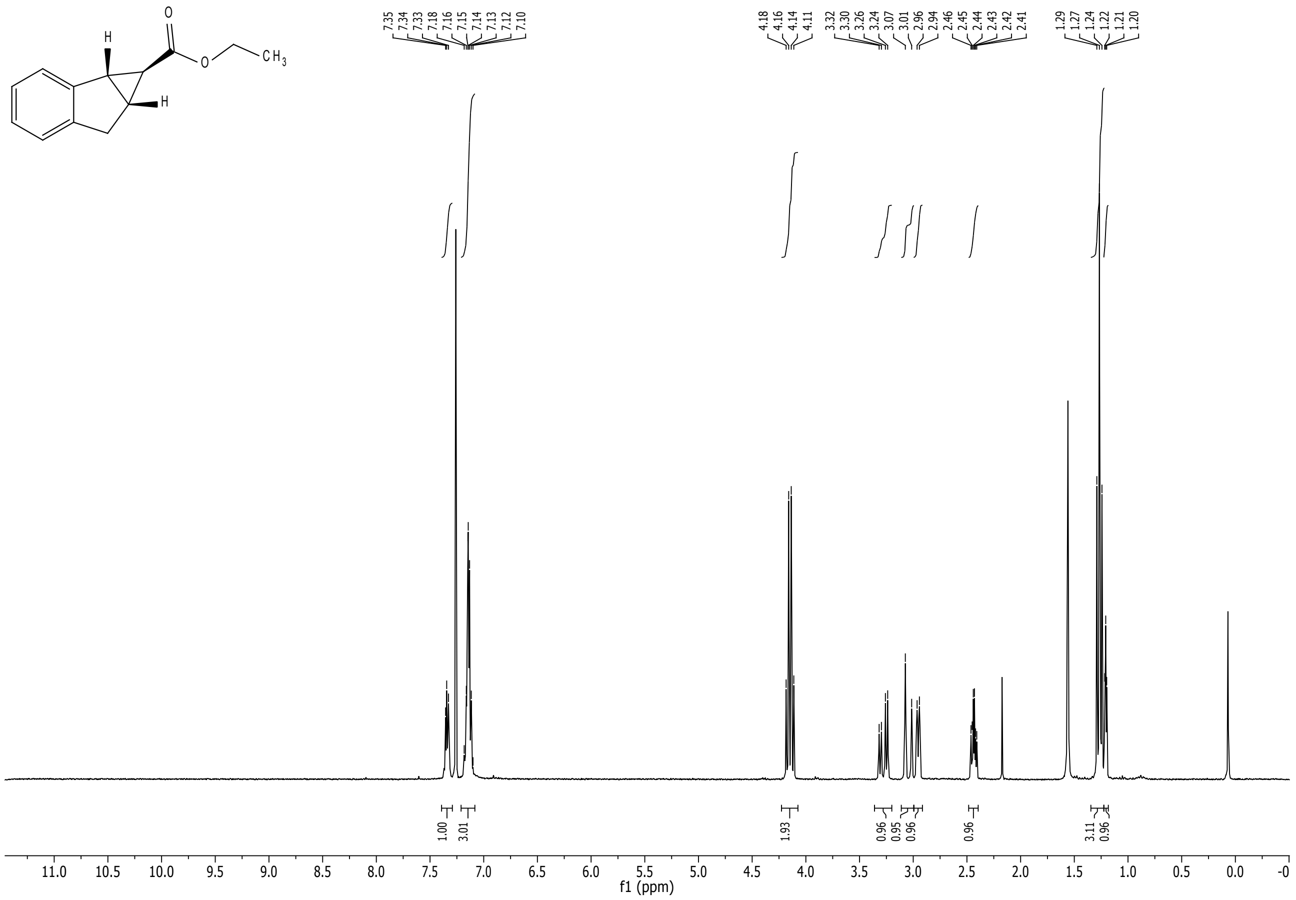
7.35 7.34 7.33 7.18 7.16 7.15 7.14 7.13 7.12 7.10
4.18 4.16 4.14 4.11
3.32 3.30 3.26 3.24 3.07 3.01 2.96 2.94
2.46 2.45 2.44 2.43 2.42 2.41
1.29 1.27 1.24 1.22 1.21 1.20
1.00 3.01 1.93 0.96 0.95 0.96 0.96 3.11 0.96
f1 (ppm)

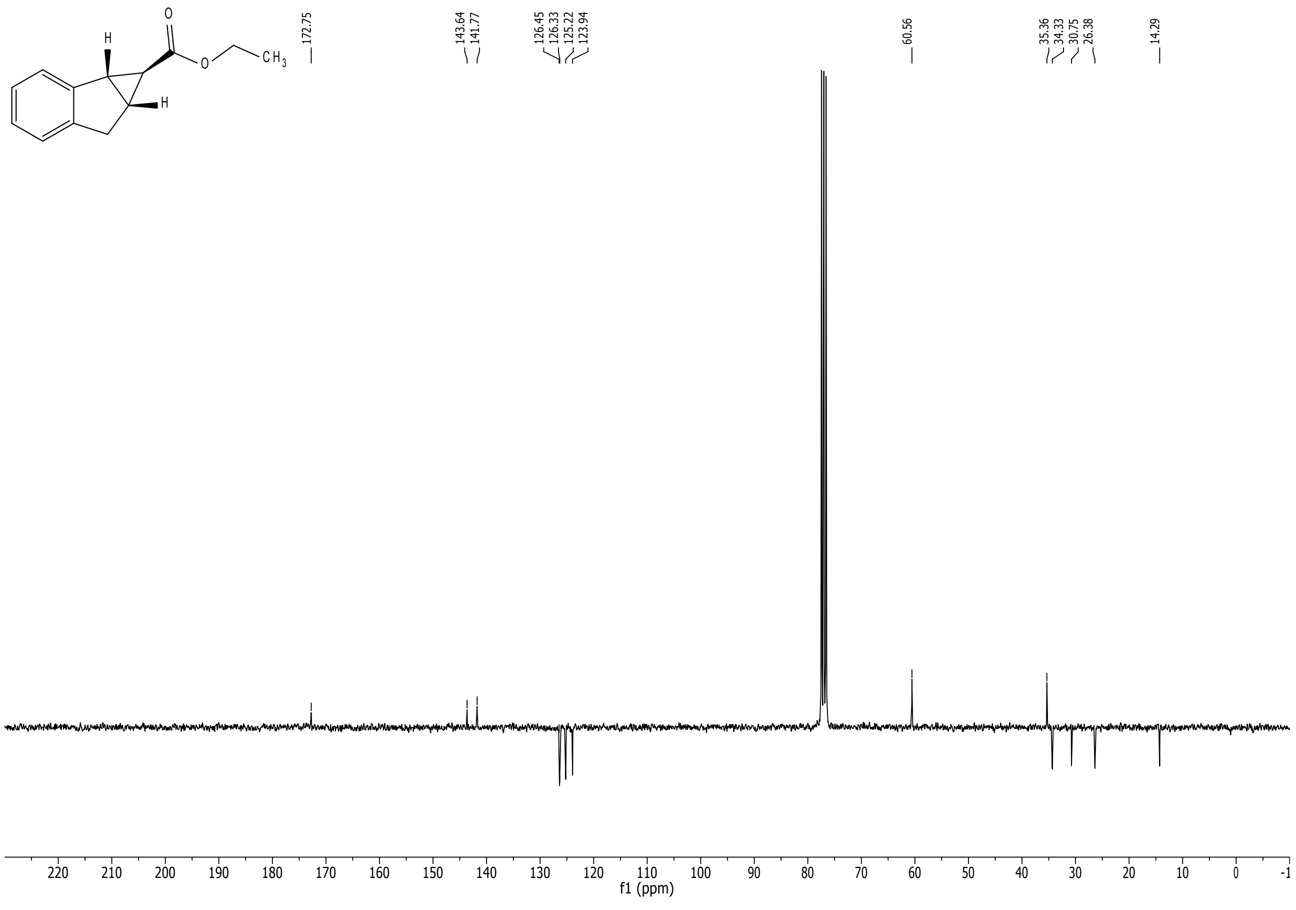
172.75
143.64 141.77
126.45 126.33 125.22 123.94
60.56
35.36 34.33 30.75 26.38
14.29
f1 (ppm)

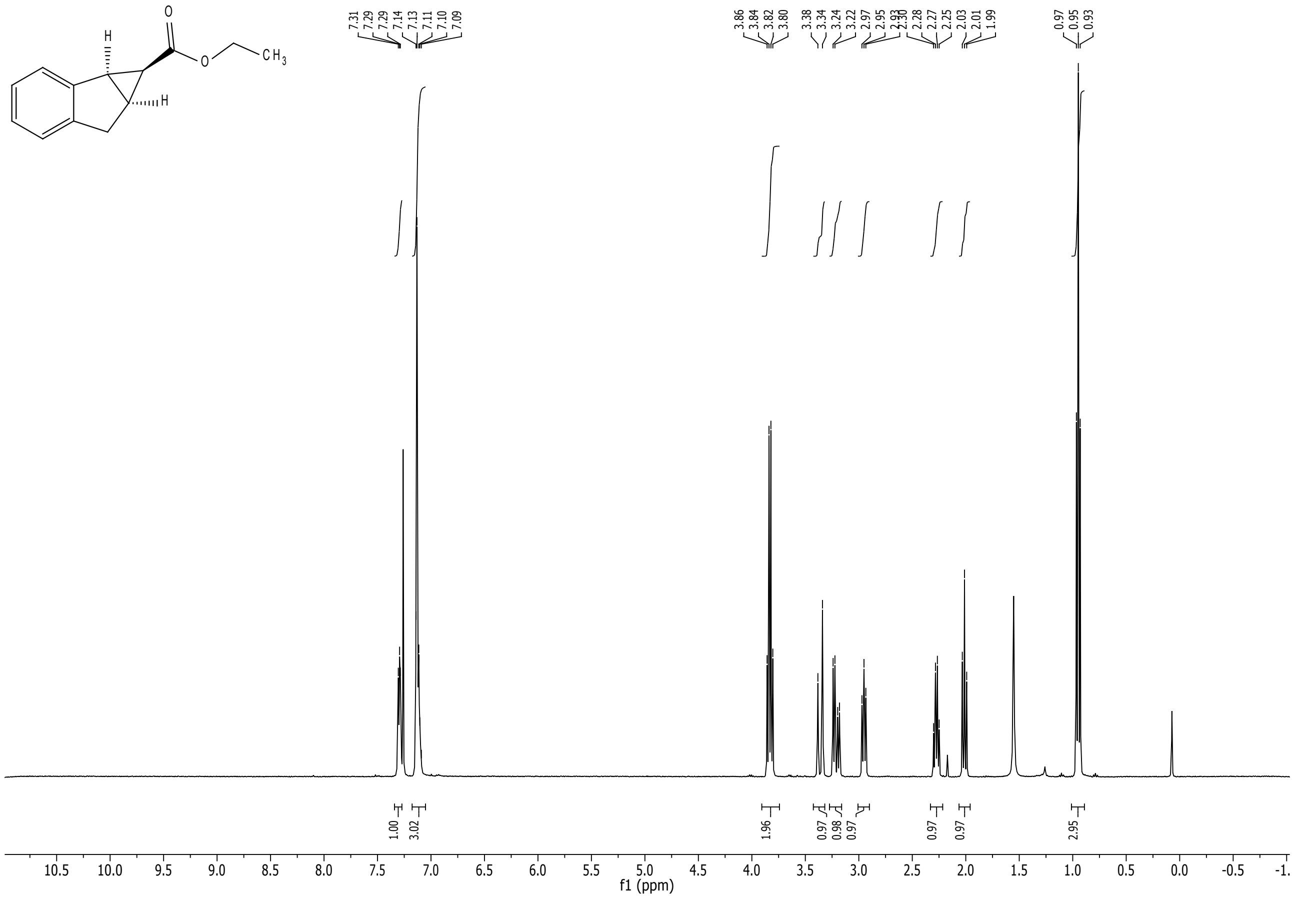

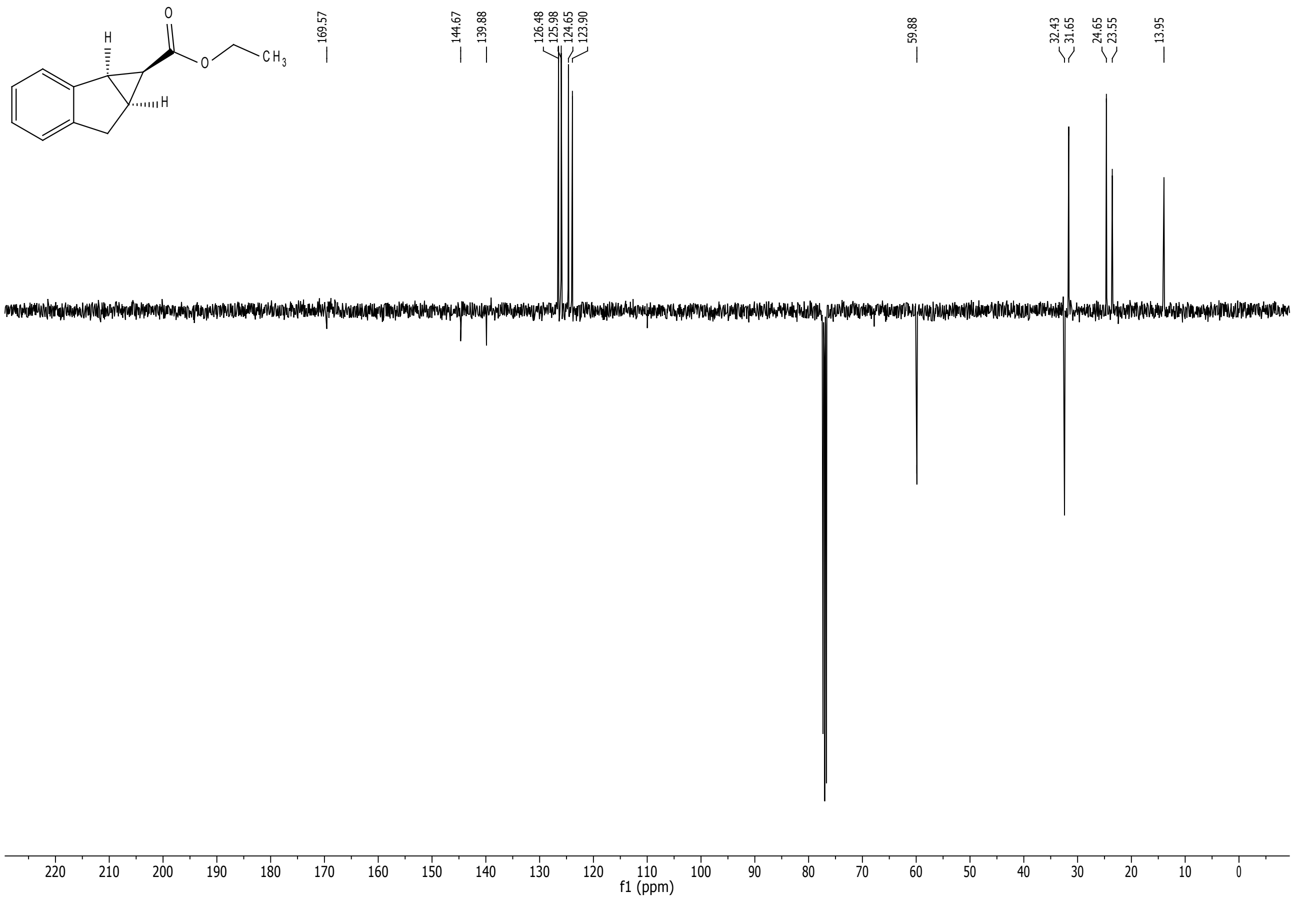

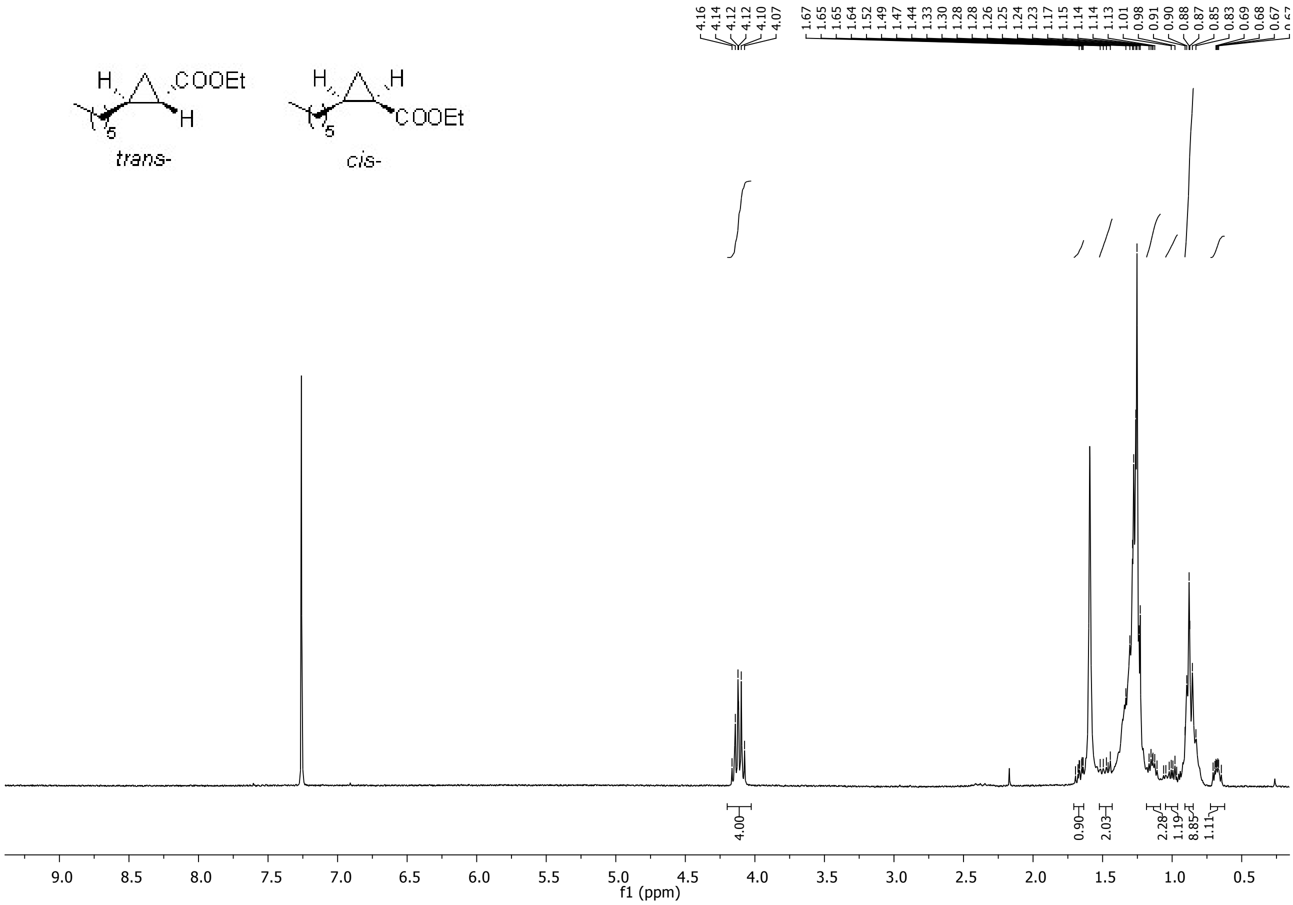
4.16 4.14 4.12 4.12 4.10 4.07
1.67 1.65 1.65 1.64 1.52 1.49 1.47 1.44 1.33 1.30 1.28 1.28 1.26 1.25 1.24 1.23 1.17 1.15 1.14 1.14 1.13 1.01 0.98 0.91 0.90 0.88 0.87 0.85 0.83 0.69 0.68
H
COOEt
H
5
trans-
H
H
COOEt
5
cis-
4.00
0.90
2.03
2.28
1.19
8.85
1.11
9.0 8.5 8.0 7.5 7.0 6.5 6.0 5.5 5.0 4.5 4.0 3.5 3.0 2.5 2.0 1.5 1.0 0.5
f1 (ppm)

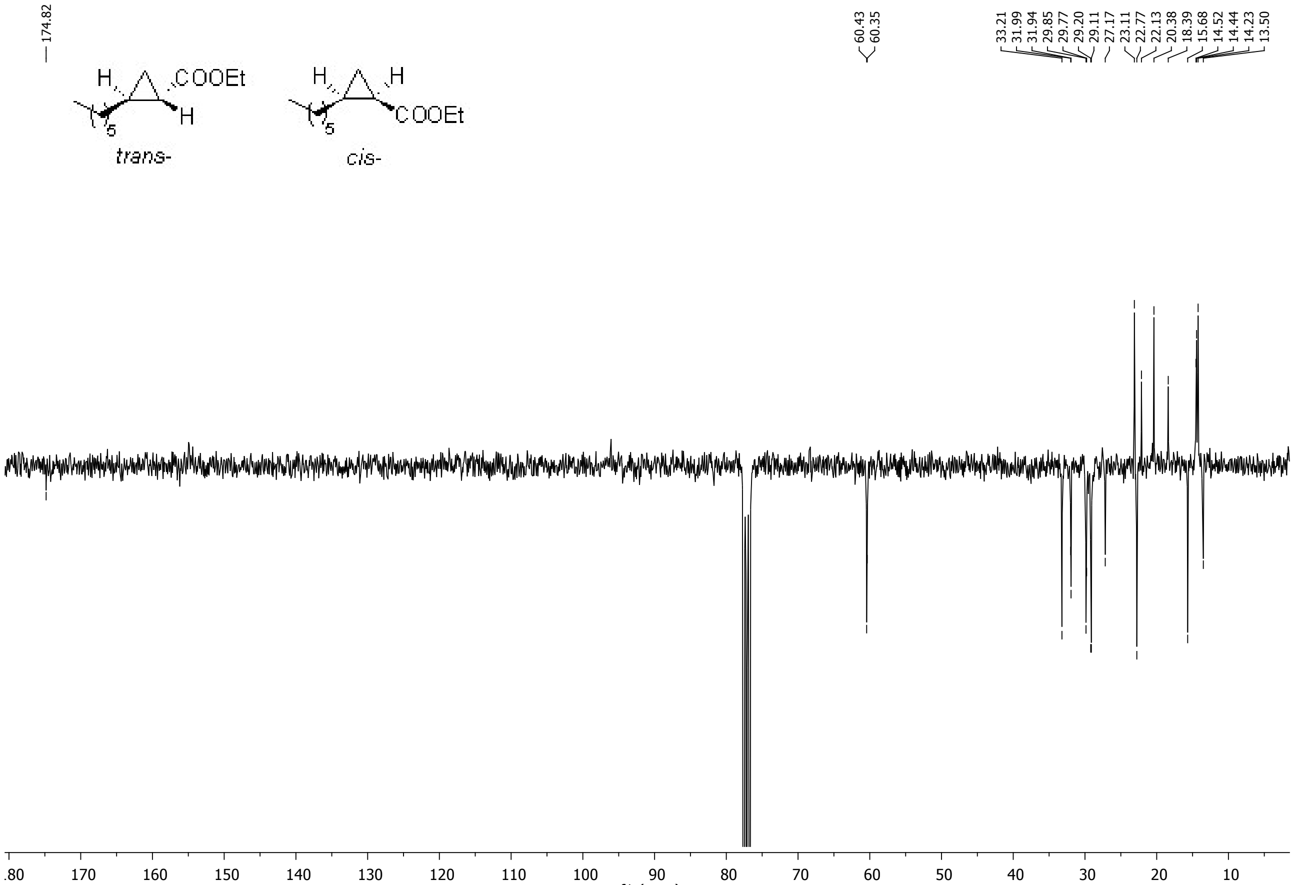
174.82
60.43 60.35
33.21 31.99 31.94 29.85 29.77 29.20 29.11 27.17 23.11 22.77 22.13 20.38 18.39 15.68 14.52 14.44 14.23 13.50
H
COOEt
H
5
trans-
H
H
COOEt
5
cis-
180 170 160 150 140 130 120 110 100 90 80 70 60 50 40 30 20 10
f1 (ppm)

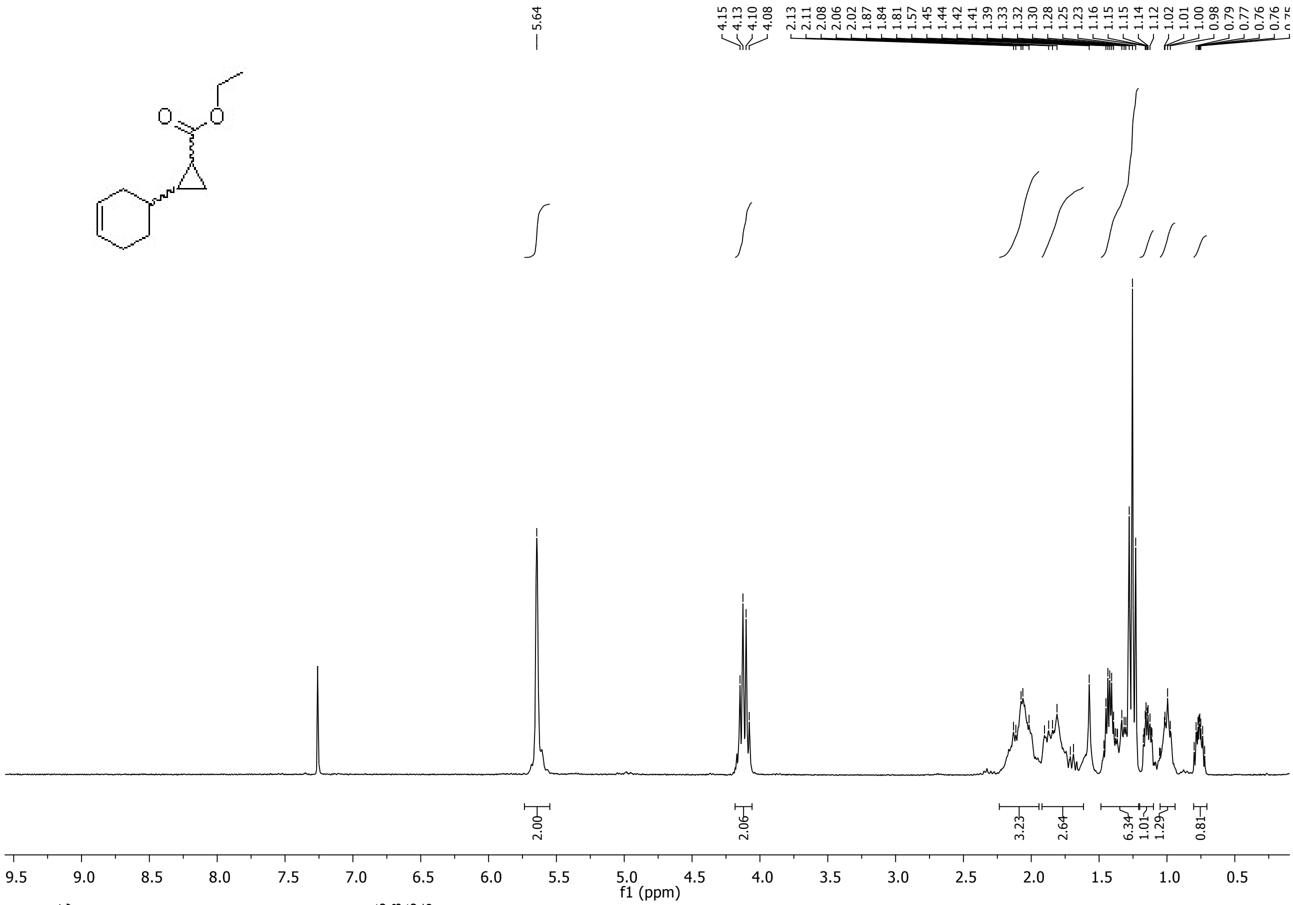
5.64
4.15
4.13
4.10
4.08
2.13
2.11
2.08
2.06
2.02
1.87
1.84
1.81
1.57
1.45
1.44
1.42
1.41
1.39
1.33
1.32
1.30
1.28
1.25
1.23
1.16
1.15
1.15
1.14
1.12
1.02
1.01
1.00
0.98
0.79
0.77
0.76
O
O
2.00
2.06
3.23
2.64
6.34
1.01
1.29
0.81
9.5 9.0 8.5 8.0 7.5 7.0 6.5 6.0 5.5 5.0 4.5 4.0 3.5 3.0 2.5 2.0 1.5 1.0 0.5
f1 (ppm)

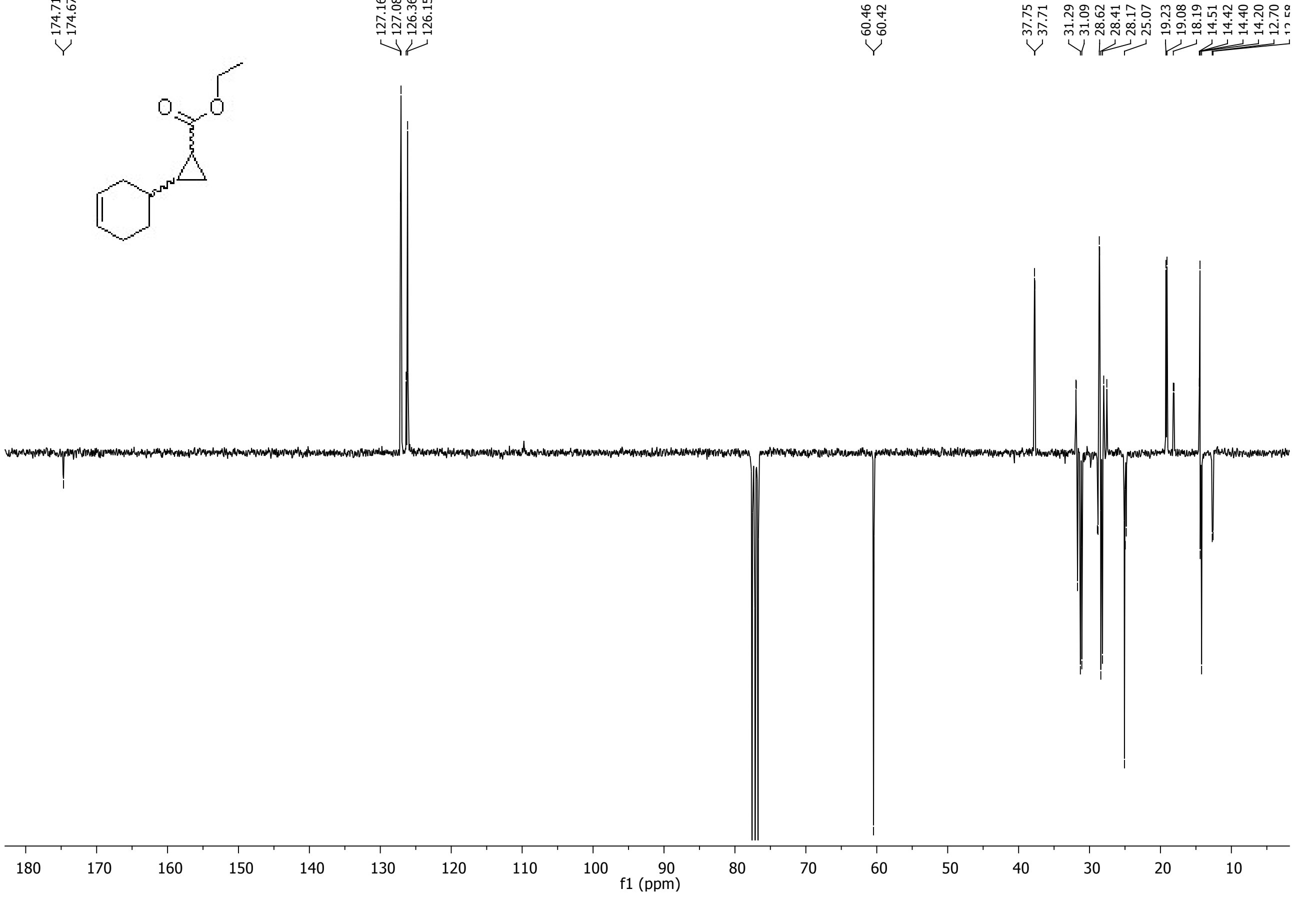
174.71
174.67
127.16
127.08
126.36
126.15
60.46
60.42
37.75
37.71
31.29
31.09
28.62
28.41
28.17
25.07
19.23
19.08
18.19
14.51
14.42
14.40
14.20
12.70
O
O
180 170 160 150 140 130 120 110 100 90 80 70 60 50 40 30 20 10
f1 (ppm)

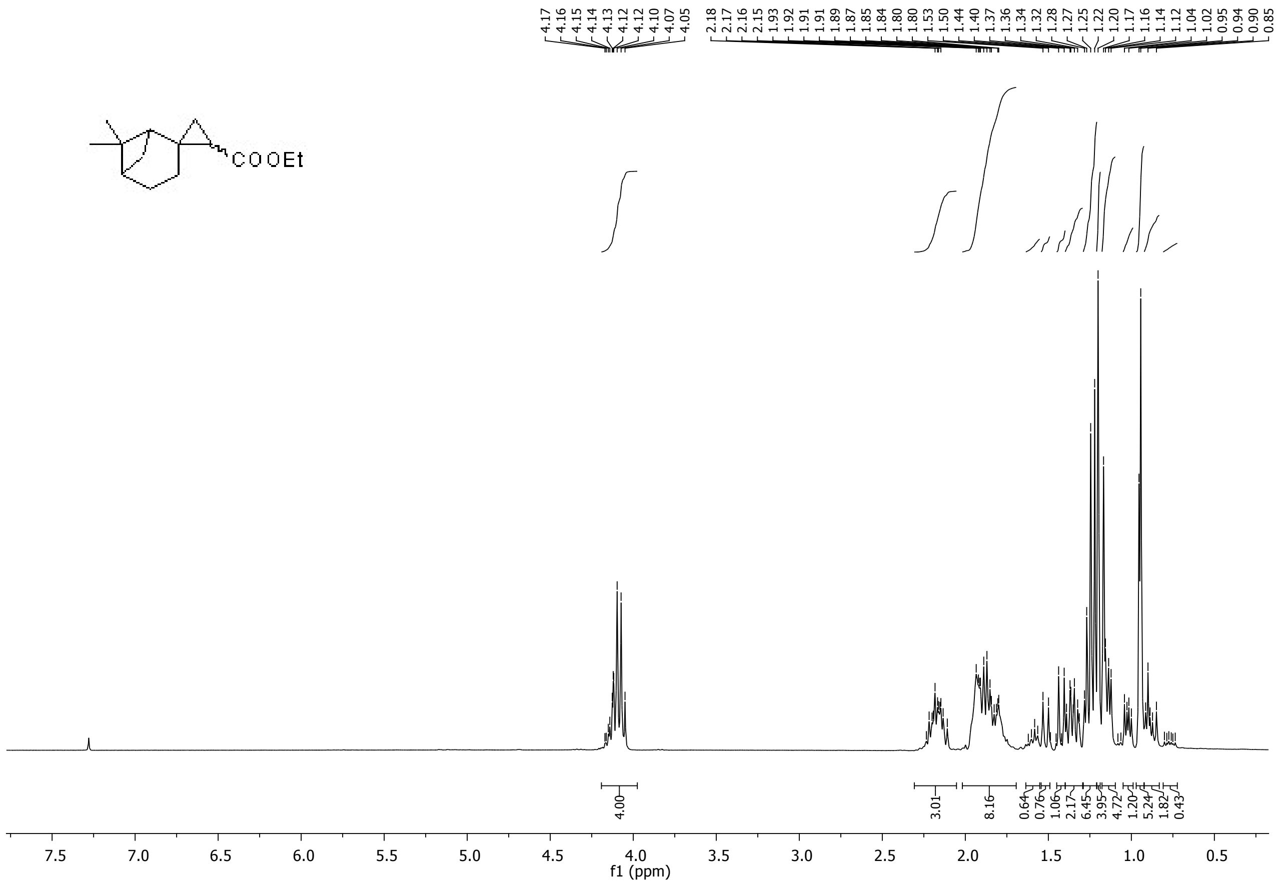
4.17 4.16 4.15 4.14 4.13 4.12 4.12 4.10 4.07 4.05
2.18 2.17 2.16 2.15 1.93 1.92 1.91 1.91 1.89 1.87 1.85 1.84 1.80 1.80 1.80 1.53 1.50 1.44 1.40 1.37 1.36 1.34 1.32 1.28 1.27 1.25 1.22 1.20 1.17 1.16 1.14 1.12 1.04 1.02 0.95 0.94 0.90 0.85
COOEt
4.00
3.01
8.16
0.64 0.76 1.06 2.17 6.45 3.95 4.72 1.20 5.24 1.82 0.43
7.5 7.0 6.5 6.0 5.5 5.0 4.5 4.0 3.5 3.0 2.5 2.0 1.5 1.0 0.5
f1 (ppm)

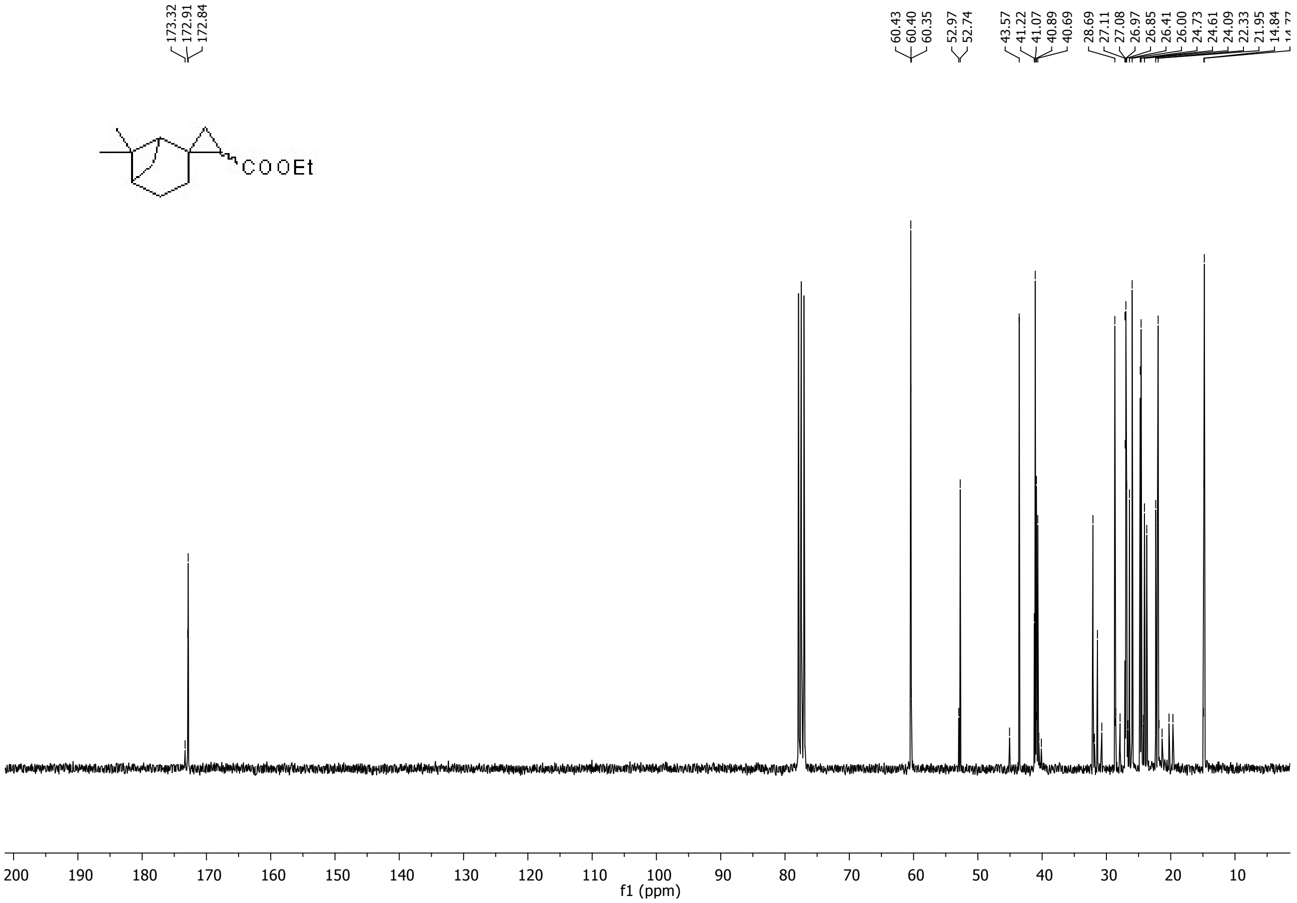
173.32 172.91 172.84
60.43 60.40 60.35
52.97 52.74
43.57 41.22 41.07 40.89 40.69
28.69 27.11 27.08 26.97 26.85 26.41 26.00 24.73 24.61 24.09 22.33 21.95 14.84
COOEt
200 190 180 170 160 150 140 130 120 110 100 90 80 70 60 50 40 30 20 10
f1 (ppm)

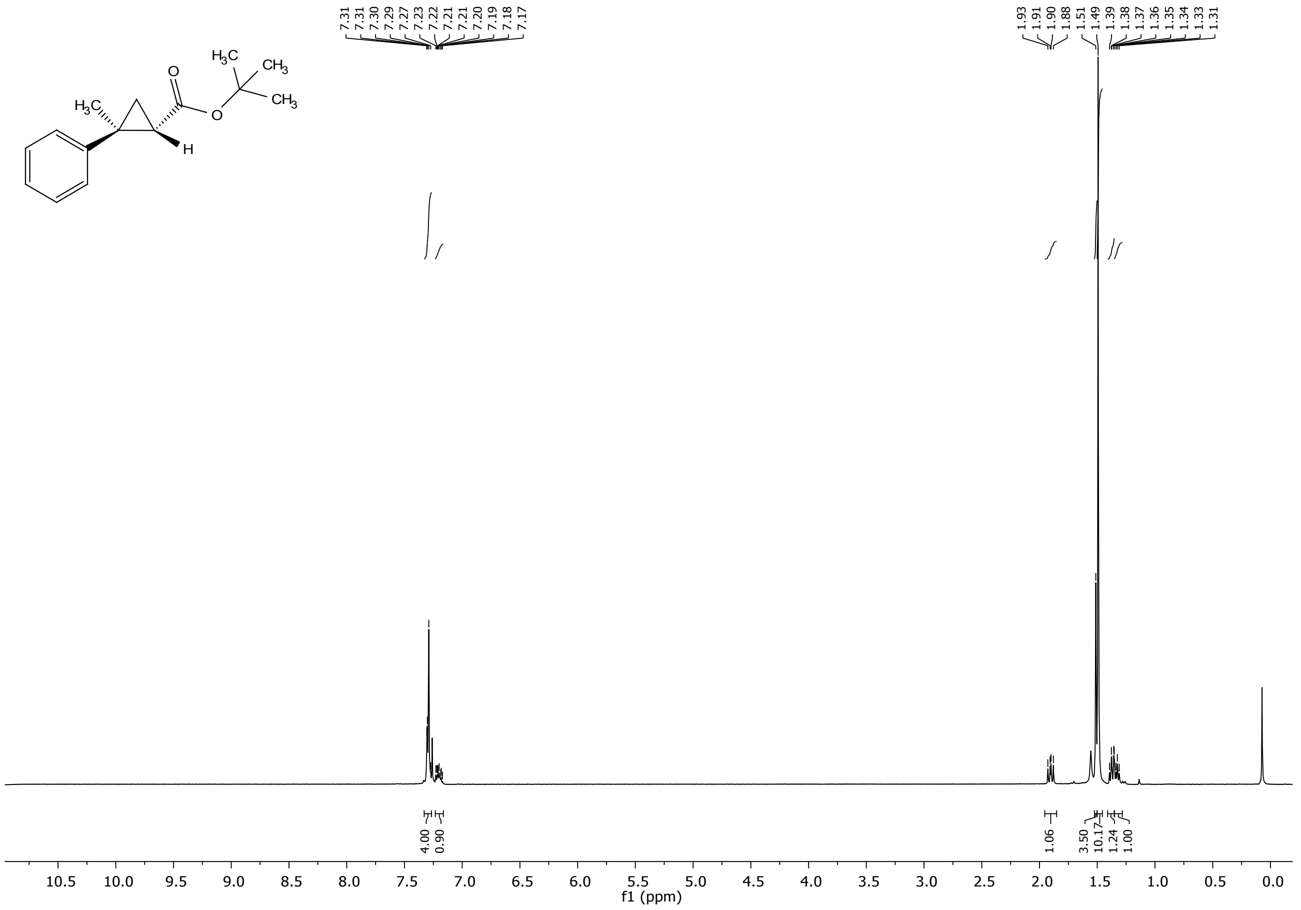
7.31
7.31
7.30
7.29
7.27
7.23
7.22
7.21
7.21
7.20
7.19
7.18
7.17
1.93
1.91
1.90
1.88
1.51
1.49
1.39
1.38
1.37
1.36
1.35
1.34
1.33
1.31
4.00
0.90
1.06
3.50
10.17
1.24
1.00
f1 (ppm)

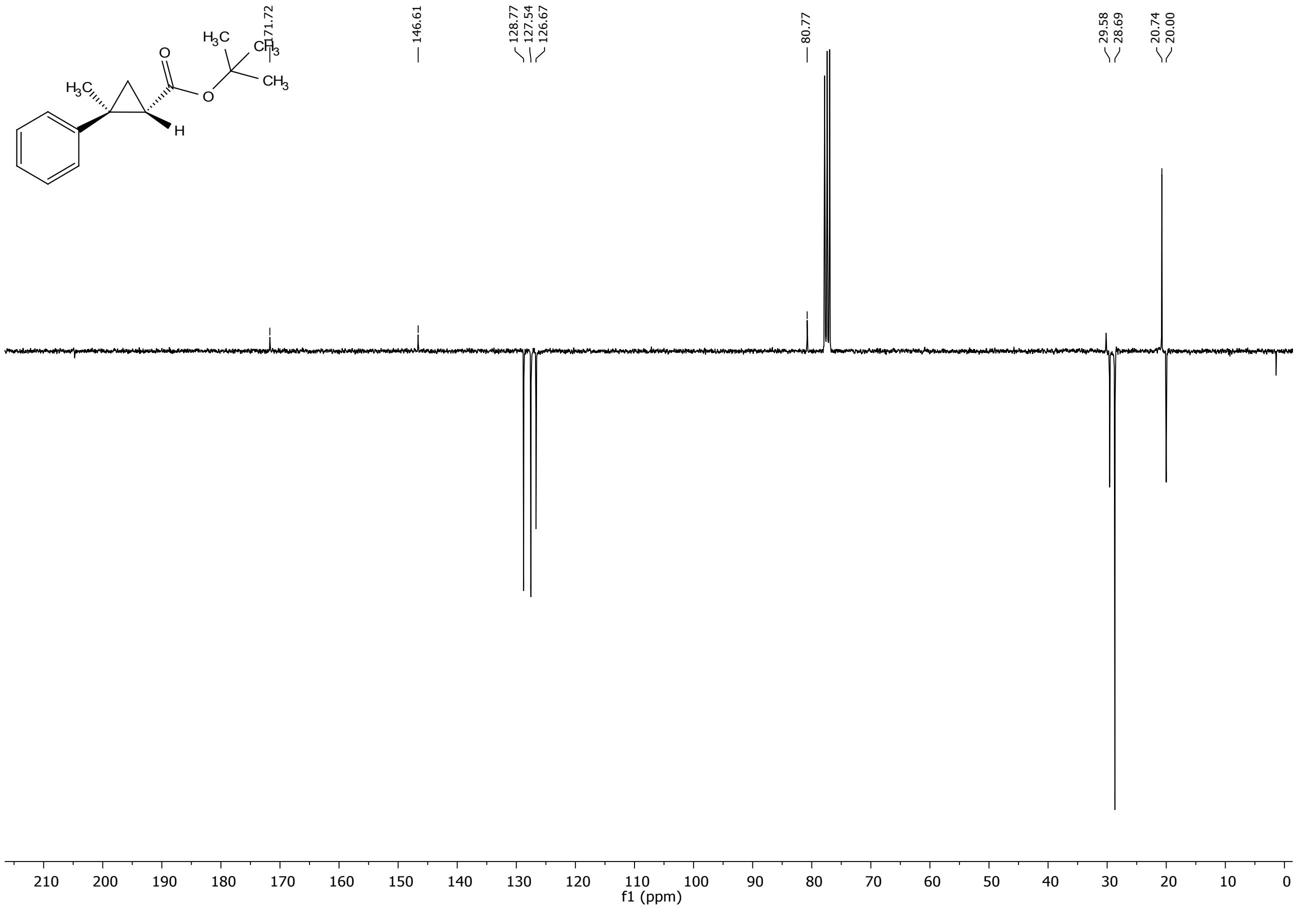

H3C
CH3
CH3
O
O
H3C
H
171.72
146.61
128.77
127.54
126.67
80.77
29.58
28.69
20.74
20.00
210 200 190 180 170 160 150 140 130 120 110 100 90 80 70 60 50 40 30 20 10 0
f1 (ppm)

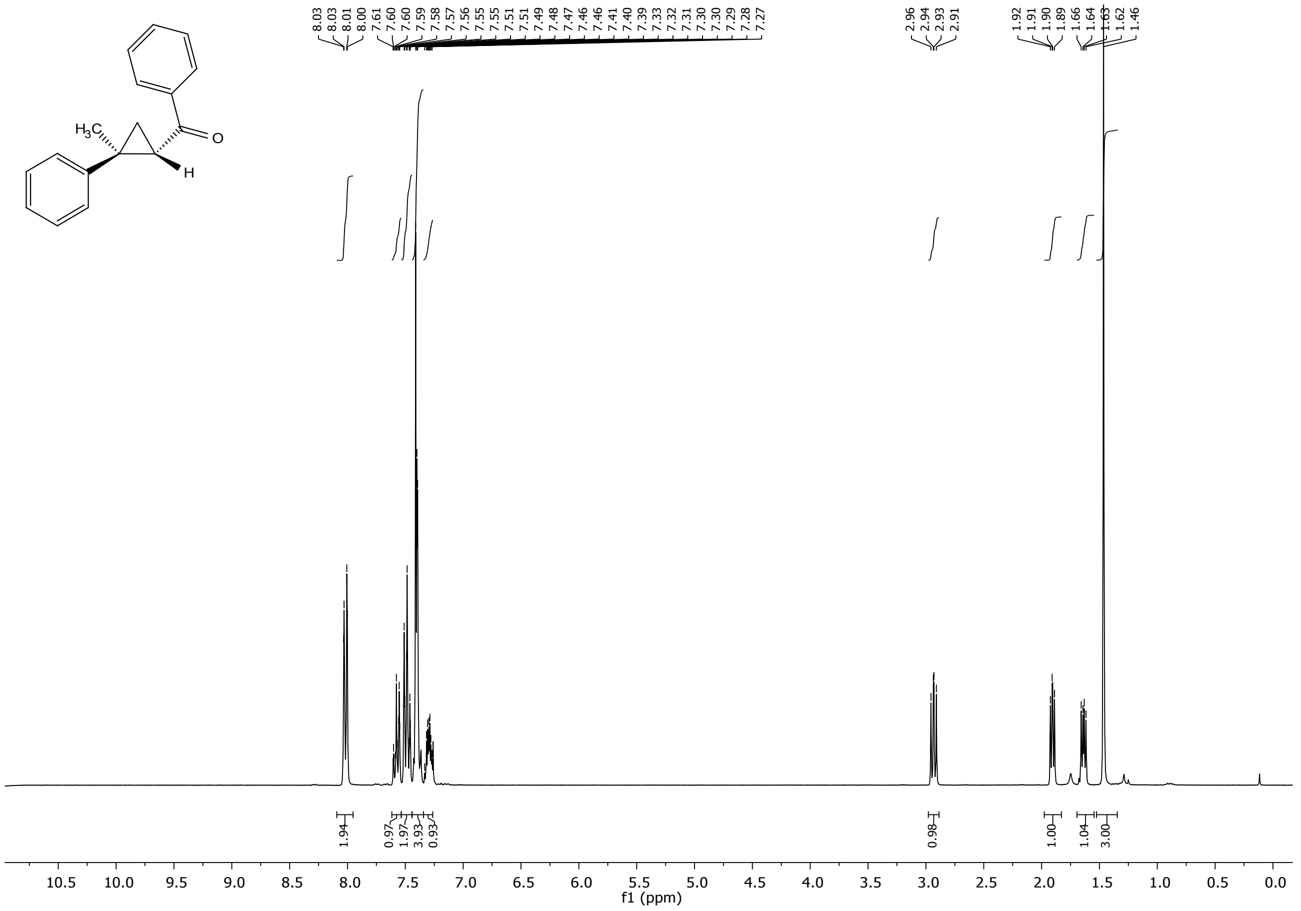
8.03 8.03 8.01 8.00 7.61 7.60 7.60 7.59 7.58 7.57 7.56 7.55 7.55 7.51 7.51 7.49 7.48 7.47 7.46 7.46 7.41 7.40 7.39 7.33 7.32 7.31 7.30 7.30 7.29 7.28 7.27
2.96 2.94 2.93 2.91
1.92 1.91 1.90 1.89 1.66 1.64 1.63 1.62 1.46
H3C
O
H
1.94
0.97 1.97 3.93 0.93
0.98
1.00
1.04 3.00
10.5 10.0 9.5 9.0 8.5 8.0 7.5 7.0 6.5 6.0 5.5 5.0 4.5 4.0 3.5 3.0 2.5 2.0 1.5 1.0 0.5 0.0
f1 (ppm)

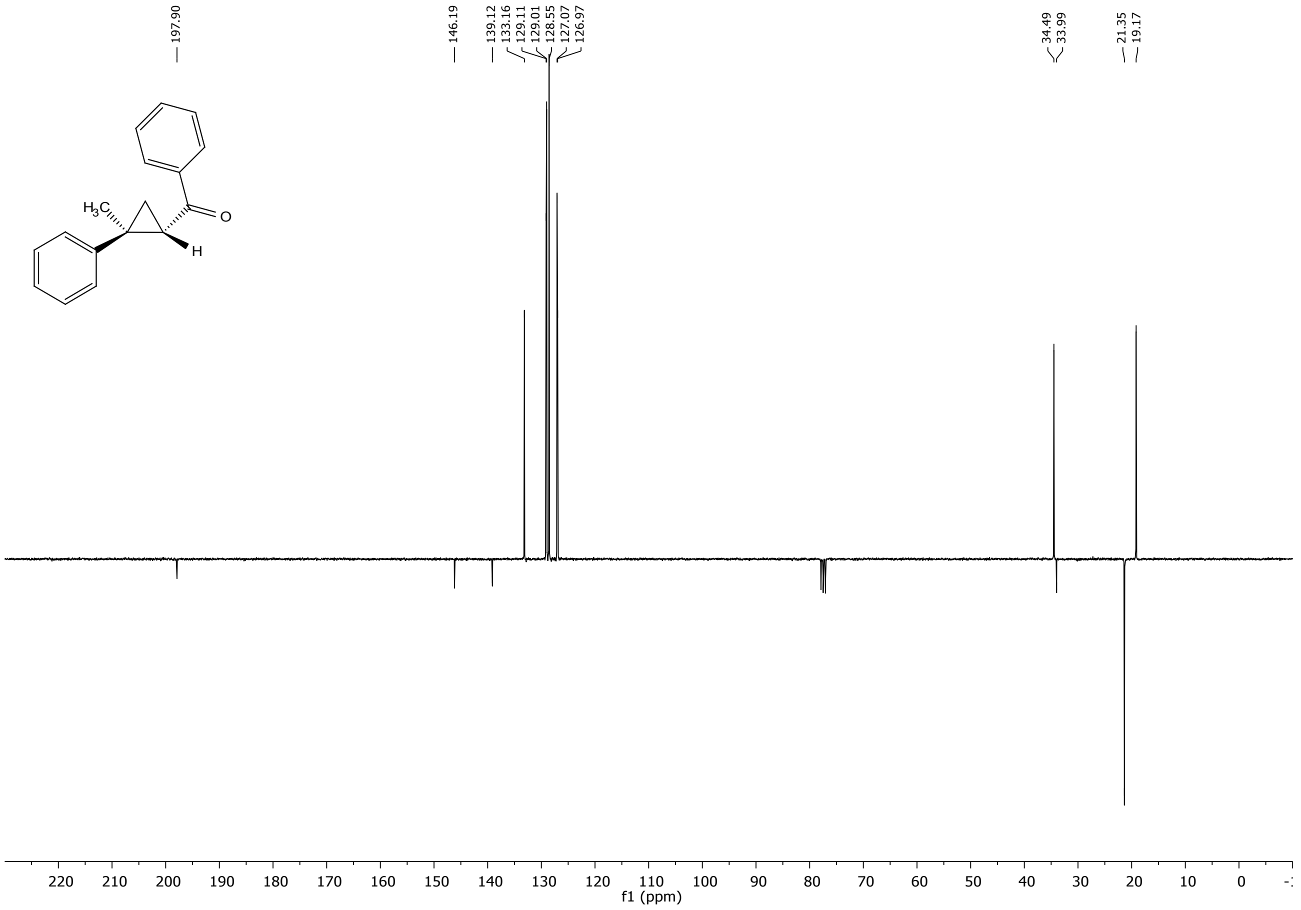
197.90
146.19
139.12
133.16
129.11
129.01
128.55
127.07
126.97
34.49
33.99
21.35
19.17
H3C
O
H
220 210 200 190 180 170 160 150 140 130 120 110 100 90 80 70 60 50 40 30 20 10 0
f1 (ppm)

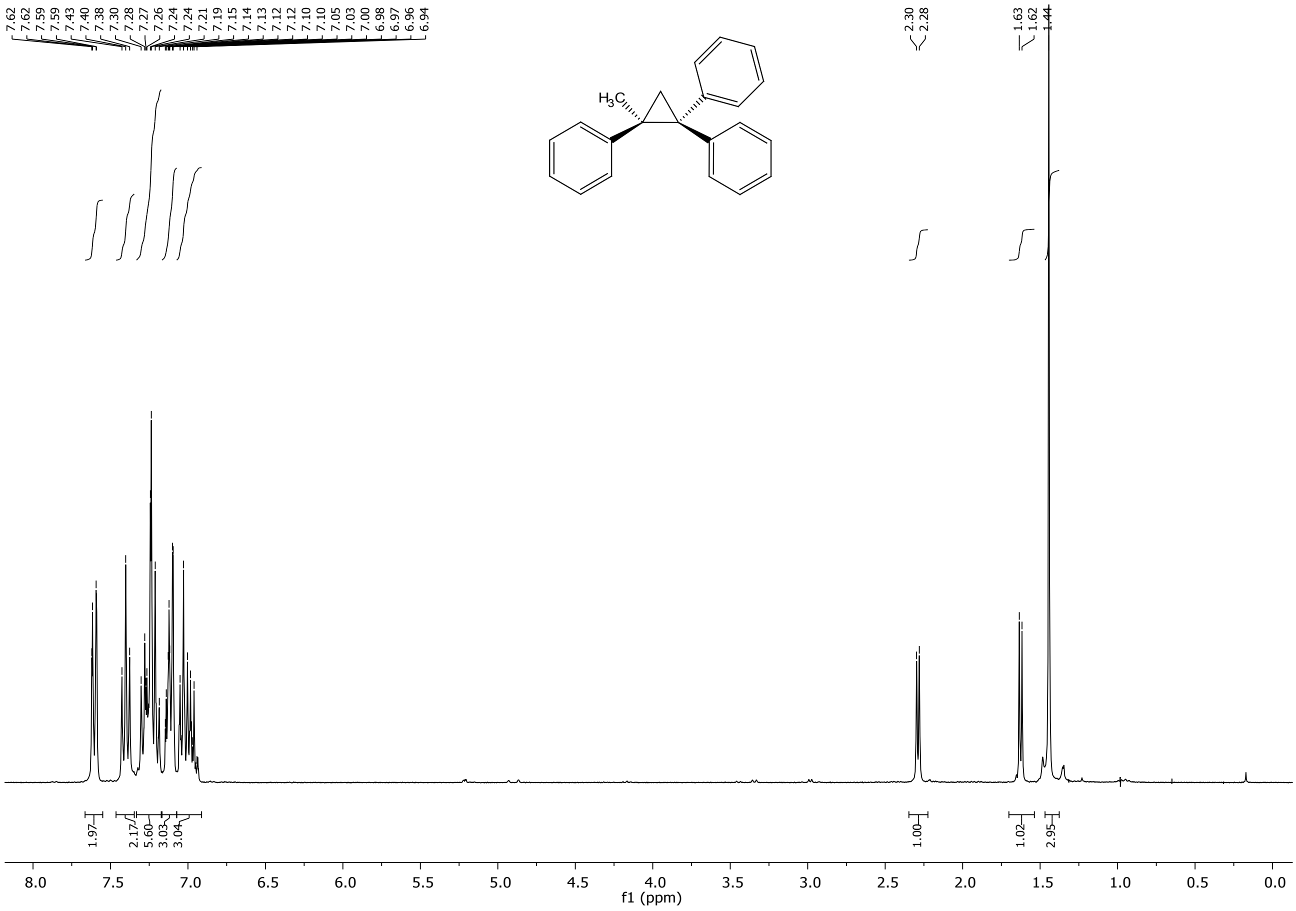

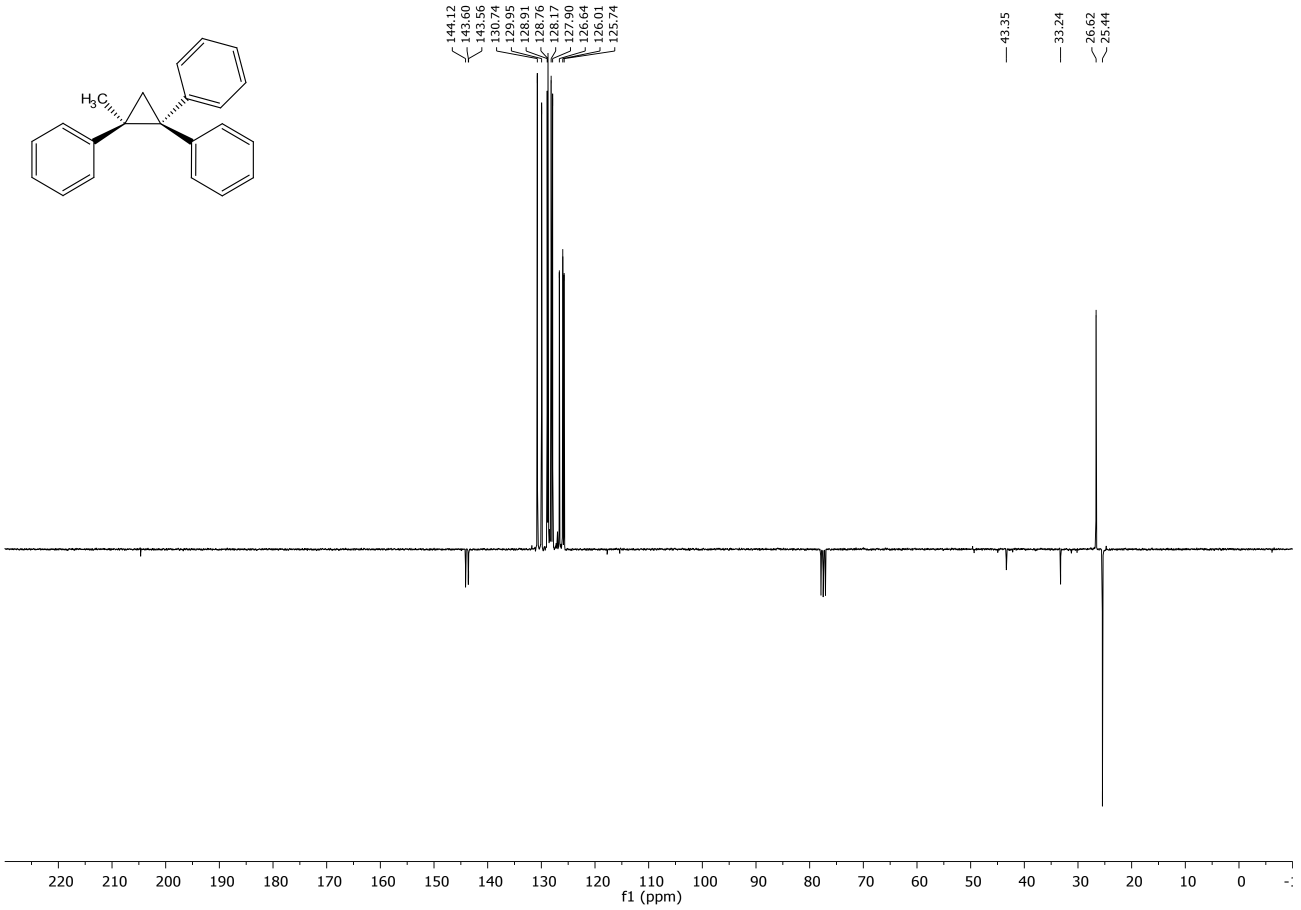

H3C
144.12
143.60
143.56
130.74
129.95
128.91
128.76
128.17
127.90
126.64
126.01
125.74
43.35
33.24
26.62
25.44
220 210 200 190 180 170 160 150 140 130 120 110 100 90 80 70 60 50 40 30 20 10 0
f1 (ppm)

9. References

1. J. Zhang, W. Chen, D. Huang, X. Zeng, X. Wang and Y. Hu, *J. Org. Chem.*, 2017, **82**, 9171-9174.
2. M. I. Javed and M. Brewer, *Org. Lett.*, 2007, **9**, 1789-1792.
3. P. Wyatt, A. Hudson, J. Charmant, A. G. Orpen and H. Phetmung, *Org. Biomol. Chem.*, 2006, **4**, 2218-2232.
4. N. Beatham and A. F. Orchard, *J. Electron. Spectrosc. Relat. Phenom.*, 1976, **9**, 129-148.
5. R. V. Jagadeesh, T. Stemmler, A.-E. Surkus, H. Junge, K. Junge and M. Beller, *Nat. Protoc.*, 2015, **10**, 548-557.
6. M. Bordeaux, V. Tyagi and R. Fasan, *Angew. Chem. Int. Ed.*, 2015, **54**, 1744-1748.
7. Y. Chen and X. P. Zhang, *J. Org. Chem.*, 2007, **72**, 5931-5934.
8. M. L. Rosenberg, A. Krivokapic and M. Tilset, *Org. Lett.*, 2009, **11**, 547-550.
9. S. Bachmann and A. Mezzetti, *Helv. Chim. Acta*, 2001, **84**, 3063-3074.
10. Y. Chen and X. P. Zhang, *J. Org. Chem.*, 2004, **69**, 2431-2435.
11. A. P. Kroitor, L. P. Cailler, A. G. Martynov, Y. G. Gorbunova, A. Y. Tsivadze and A. B. Sorokin, *Dalton Trans.*, 2017, **46**, 15651-15655.
12. D. M. Carminati, D. Intrieri, A. Caselli, S. Le Gac, B. Boitrel, L. Toma, L. Legnani and E. Gallo, *Chem. Eur. J.*, 2016, **22**, 13599-13612.
13. S. Fantauzzi, E. Gallo, E. Rose, N. Raoul, A. Caselli, S. Issa, F. Ragaini and S. Cenini, *Organometallics*, 2008, **27**, 6143-6151.
14. G.-J. Jiang, X.-F. Fu, Q. Li and Z.-X. Yu, *Org. Lett.*, 2012, **14**, 692-695.
15. M. L. Rosenberg, K. Vlašaná, N. S. Gupta, D. Wragg and M. Tilset, *J. Org. Chem.*, 2011, **76**, 2465-2470.
16. A. G. M. Barrett, D. C. Braddock, I. Lenoir and H. Tone, *J. Org. Chem.*, 2001, **66**, 8260-8263.
17. M. Hagar, F. Ragaini, E. Monticelli, A. Caselli, P. Macchi and N. Casati, *Chem. Commun.*, 2010, **46**, 6153-6155.
18. T. Nishimura, K. Ohe and S. Uemura, *J. Org. Chem.*, 2001, **66**, 1455-1465.
19. H. Liu, Y. Wei and C. Cai, *New J. Chem.*, 2016, **40**, 674-678.
